# Intermolecular water attack to alkenylgold carbenes derived from 3-propargylindoles

**DOI:** 10.3762/bjoc.22.98

**Published:** 2026-09-02

**Authors:** Lorena Renedo, Marta Solas, Samuel Suárez-Pantiga, Roberto Sanz

**Affiliations:** 1 Área de Química Orgánica, Departamento de Química, Facultad de Ciencias, Universidad de Burgos, Pza. Misael Bañuelos s/n, 09001 Burgos, Spain

**Keywords:** alcohols, gold catalysis, hydroxylation, indoles, vinyl carbenes

## Abstract

An intermolecular hydroxylation of α,β-unsaturated gold carbene intermediates, generated from readily accessible 3-propargylindoles through a tandem 1,2-indole migration–hydroxylation sequence using water as an external nucleophile, has been developed. Under mild reaction conditions and employing gold(I) catalysts bearing bulky and electron-rich phosphine ligands, a significant range of hydroxy-functionalized indole derivatives was synthesized in high yields. Mechanistic studies support the selective addition of water to the β-position of the electrophilic α,β-unsaturated gold carbene intermediate, whereas competing attack at the carbene center or direct hydration of the activated alkyne was not observed.

## Introduction

Gold-catalyzed reactions involving the generation of alkenyl carbene intermediates have emerged as a powerful synthetic tool due to their diversified transformation sites. Typical precursors for these highly reactive intermediates are stabilized vinyldiazo compounds [1] and cyclopropenes [2] and, although Rh-catalyzed processes are the most well-established, Au catalysts have gained attention more recently [3–4]. However, it is highly desirable to generate alkenylgold carbenes from more easily accessible alkynes. In this sense, propargylic esters [5] and sulfides [6] are the most prevalent reported precursors for these intermediates, through 1,2-acyloxy and 1,2-sulfur migrations, respectively. In addition, more specific substrates such as 7-alkenyl-1,3,5-cycloheptatrienes [7], 1,6-enynes with propargylic alkoxy groups [8], or terminal diynes [9], have also been reported as suitable precursors.

Alkenylgold carbenes typically present carbene reactivity, such as cyclopropanation, C–H activation, 1,2-hydride shifts, or 1,2-addition reactions [10–13], whereas the so-called vinylogous reactivity at the β-position of the unsaturated gold carbene has been scarcely explored [14–16]. In addition, these intermediates also behave as three-carbon synthons as demonstrated by the different and useful (3 + *n*) carbo- and heterocycloaddition reactions described over the last few years [17].

**Scheme 1:**
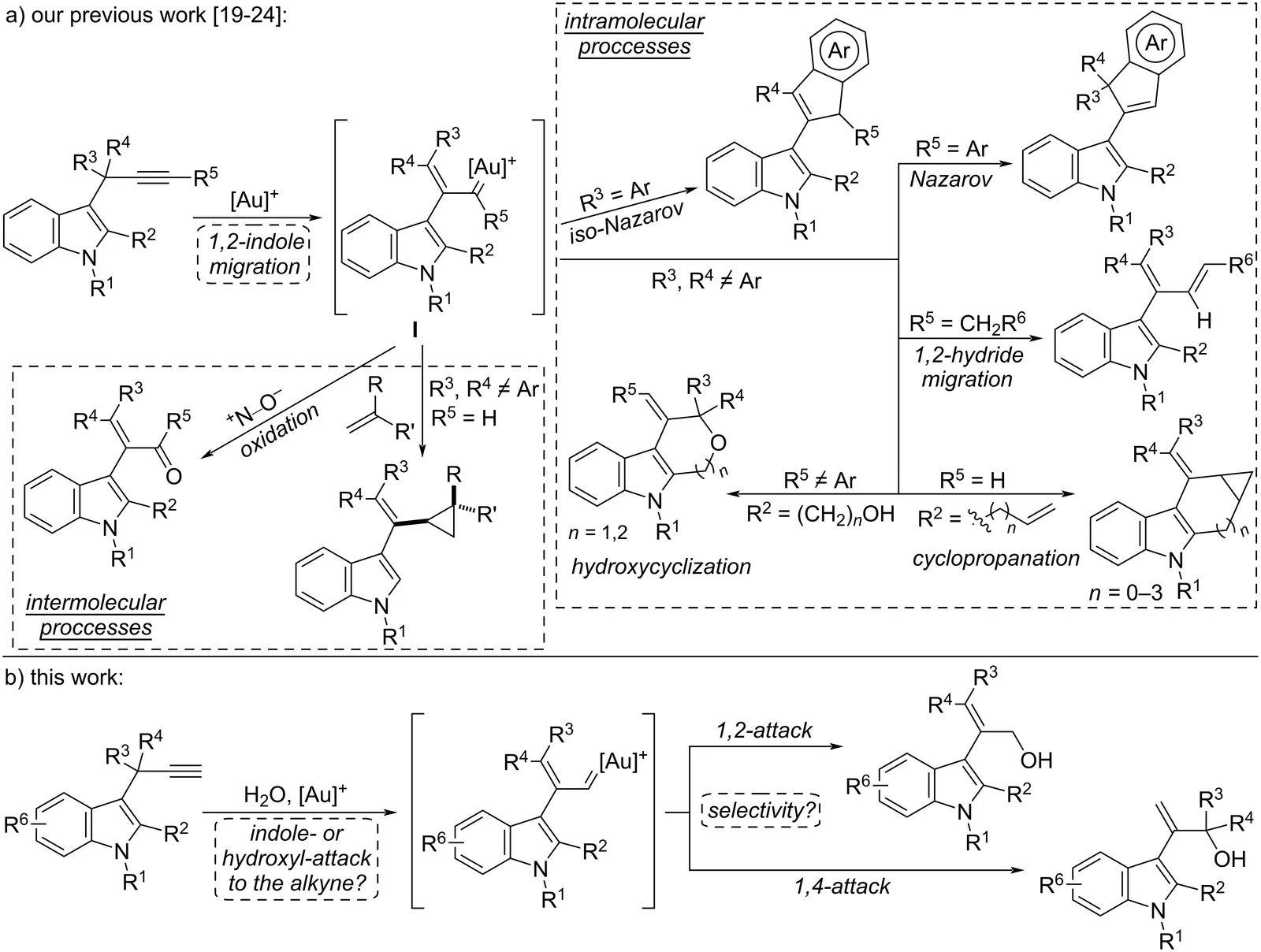
a) Intra- and intermolecular reactivity of α-indolylalkenylgold carbenes from 3-propargylindoles. b) Proposed work.

In this field, we have found that 3-propargylindoles, which are readily available through the direct nucleophilic substitution of indoles with propargylic alcohols [18], undergo a 1,2-indole migration under gold(I) catalysis to generate α,β-unsaturated gold carbenes **I** (Scheme 1a) [19]. Interestingly, these reactions involve the cleavage and formation of C–C bonds, instead of C–O or C–S bonds observed in the corresponding propargylic esters and sulfides, respectively. Electrophilic intermediates **I** exhibit rich reactivity through both intramolecular and intermolecular pathways. In the intramolecular manifold, highly favored aura-(iso)-Nazarov cyclizations take place when aromatic groups are present at either the propargylic (R^3^) or the terminal (R^5^) positions of the propagylindole, leading to 3-(inden-2-yl)indoles. In contrast, when no aromatic substituents are present at any of these positions (R^3^–R^5^ ≠ Ar), an alternative 1,2-hydride migration occurs, affording 2-indolyl-1,3-butadienes [20–21]. Furthermore, when an alkenyl group is located at C2 of the starting propargylindole, an intramolecular cyclopropanation takes place, providing access to 5–8-membered polycyclic indole derivatives [22]. More recently, we have found that 3-propargylindoles bearing a hydroxyalkyl substituent at C2 enable access to indole-fused cyclic ethers through addition of the hydroxy group to the β-position of gold intermediate **I** (vinylogous reactivity), which was further supported by DFT studies [23]. On the other hand, these alkenyl gold carbenes can also participate in intermolecular reactions. For example, their reactions with *N*-oxides enable carbene oxidation, leading to α-indolyl α,β-unsaturated carbonyls [24]. Moreover, treatment of 3-propargylindoles with external alkenes under gold catalysis results in intermolecular cyclopropanation reactions with high *cis*-diastereoselectivity, providing access to 3-(1-cyclopropylvinyl)indoles [22]. At this point, considering our recent report on the tandem 1,2-indole migration–hydroxycyclization reactions of 2-hydroxyalkyl-3-propargylindoles [23], we wondered whether hydroxyl attack on vinylgold carbenes **I** could also occur in an intermolecular fashion, analogous to the reactivity of alkenes, which can react with **I** through both intra- and intermolecular pathways. However, hydroxyl attack on α,β-unsaturated carbene **I** could potentially proceed through either attack at the carbene center or addition to the β-position (vinylogous reactivity). In addition, OH-attack could also occur directly on the gold-activated alkyne, without participation of the indole nucleus. We herein report our results on the gold(I)-catalyzed reactions of 3-propargylindoles with water.

## Results and Discussion

**Scheme 2:**
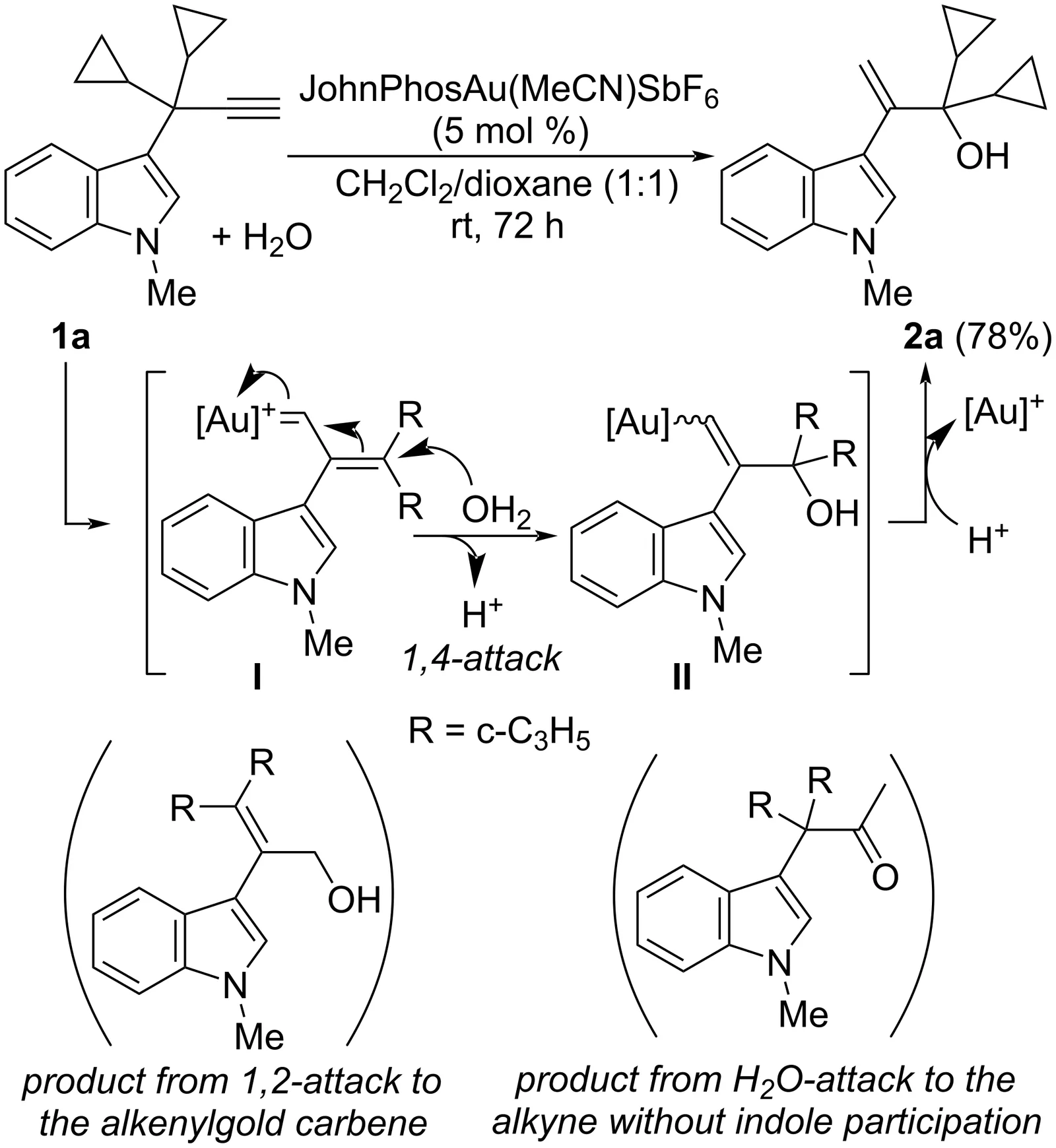
Proof of concept.

3-Propargylindole **1a**, readily accessible in gram quantities through the Brønsted acid-catalyzed direct nucleophilic substitution of the corresponding propargylic alcohol with *N*-methylindole [18], was selected as the model substrate for its reaction with H_2_O as an external nucleophile under gold catalysis. Considering our recent work on the tandem Au-catalyzed 1,2-indole migration–intramolecular hydroxycyclization reaction [23], JohnPhosAu(MeCN)SbF_6_ was chosen as the catalyst for a preliminary study (Scheme 2). A mixture of CH_2_Cl_2_ and dioxane was employed to enhance the solubility of H_2_O and promote its interaction with the reactive intermediates [25–26]. Under these conditions, and at room temperature, alcohol **2a** was selectively obtained in high yield, although prolonged reaction times were required to achieve complete conversion. This result supports a selective water attack onto intermediate α,β-unsaturated gold carbene **I** through a formal β-addition pathway, leading to a new vinylgold complex **II**, which subsequently undergoes protodemetalation to regenerate the catalyst and furnish **2a**. Interestingly, neither the competing addition to the carbene center pathway nor direct hydration of the activated alkyne to form the corresponding methyl ketone, without involvement of the indole moiety, was observed. In addition, the resulting compound **2a** belongs to the class of 3-alkenylindoles, a family of functionalized indoles that exhibit rich reactivity, primarily owing to their nucleophilic character [27–28].

At this stage, we decided to evaluate the reactivity of **1a** with H_2_O under catalysis by different gold(I) complexes (Table 1). Although a gold complex bearing a JohnPhos ligand was initially selected (see Scheme 2), approximately 3 days were required for complete conversion, with only ca. 30% conversion observed after 24 h (Table 1, entry 1). Gold catalysts bearing phosphine ligands such as MorDalPhos or DavePhos proved unsuitable for this transformation (Table 1, entries 2 and 3), as did IPrAuNTf_2_ bearing an NHC ligand (Table 1, entry 4). In contrast, gold complexes containing other phosphine ligands, such as *t*-Bu_3_P, Ph_3_P, and JackiePhos, afforded alcohol **2a**, although complete conversion was not achieved after 24 h (Table 1, entries 5–7). Gratifyingly, gold catalysts bearing bulkier phosphine ligands such as SPhos, XPhos and BrettPhos (Table 1, entries 8–10) provided **2a** in high yields with complete conversion, the latter requiring only 3 h (Table 1, entry 10). Further increasing the steric hindrance around the metal center by using bulkier ligands such as di-Ad-BrettPhos and Me_4_*t*-BuXPhos led to even higher yields of **2a** (Table 1, entries 11 and 12) [29]. The enhanced performance observed with these ligands likely arises from suppression of alternative side reactions of intermediate gold carbene **I** due to the steric congestion around the metal center imposed by these ligands. Other cosolvents, such as acetone (Table 1, entry 13), could also be employed, whereas in the absence of the oxygenated polar cosolvent, the reaction was not observed. Using Me_4_*t*-BuXPhos as ligand, the effect of the presence of silver in the reaction medium was also examined, revealing slightly lower efficiency (Table 1, entry 14).

**Table 1:** Optimization of catalyst for the Au-catalyzed tandem 1,2-indole migration–hydroxylation of **1a**.^a^

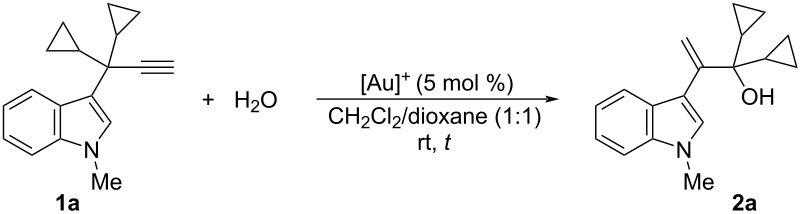				

Entry	Catalyst	Time (h)	Conversion (%)^b^	Yield (%)^b^

1	JohnPhosAu(MeCN)SbF_6_	24	30	25
2	MorDalPhosAu(MeCN)SbF_6_	24	0	–
3	DavePhosAuNTf_2_	24	0	–
4	IPrAuNTf_2_	24	0	–
5	*t*-Bu_3_PAuNTf_2_	24	35	28
6	Ph_3_PAuNTf_2_	24	72	38
7	JackiePhosAuNTf_2_	24	81	73
8	SPhosAuNTf_2_	24	100	64
9	XPhosAuNTf_2_	24	100	75
10	BrettPhosAuNTf_2_	3	100	75
11	di-Ad-BrettPhosAuNTf_2_	3	100	83
12	(Me_4_*t*-BuXPhos)AuNTf_2_	3	100	89
13^c^	(Me_4_*t*-BuXPhos)AuNTf_2_	3	100	79
14	(Me_4_*t*-BuXPhos)AuCl/AgNTf_2_	3	100	78

^a^Reactions were carried out by treatment of **1a** (0.1 mmol) with H_2_O (0.7 mmol, 13 μL) in 1 mL of CH_2_Cl_2_/1,4-dioxane (1:1) for the referred time at room temperature. ^b^Determined by ^1^H NMR analysis using CH_2_Br_2_ as internal standard. ^c^Carried out in 1 mL of CH_2_Cl_2_/acetone (1:1).

**Scheme 3:**
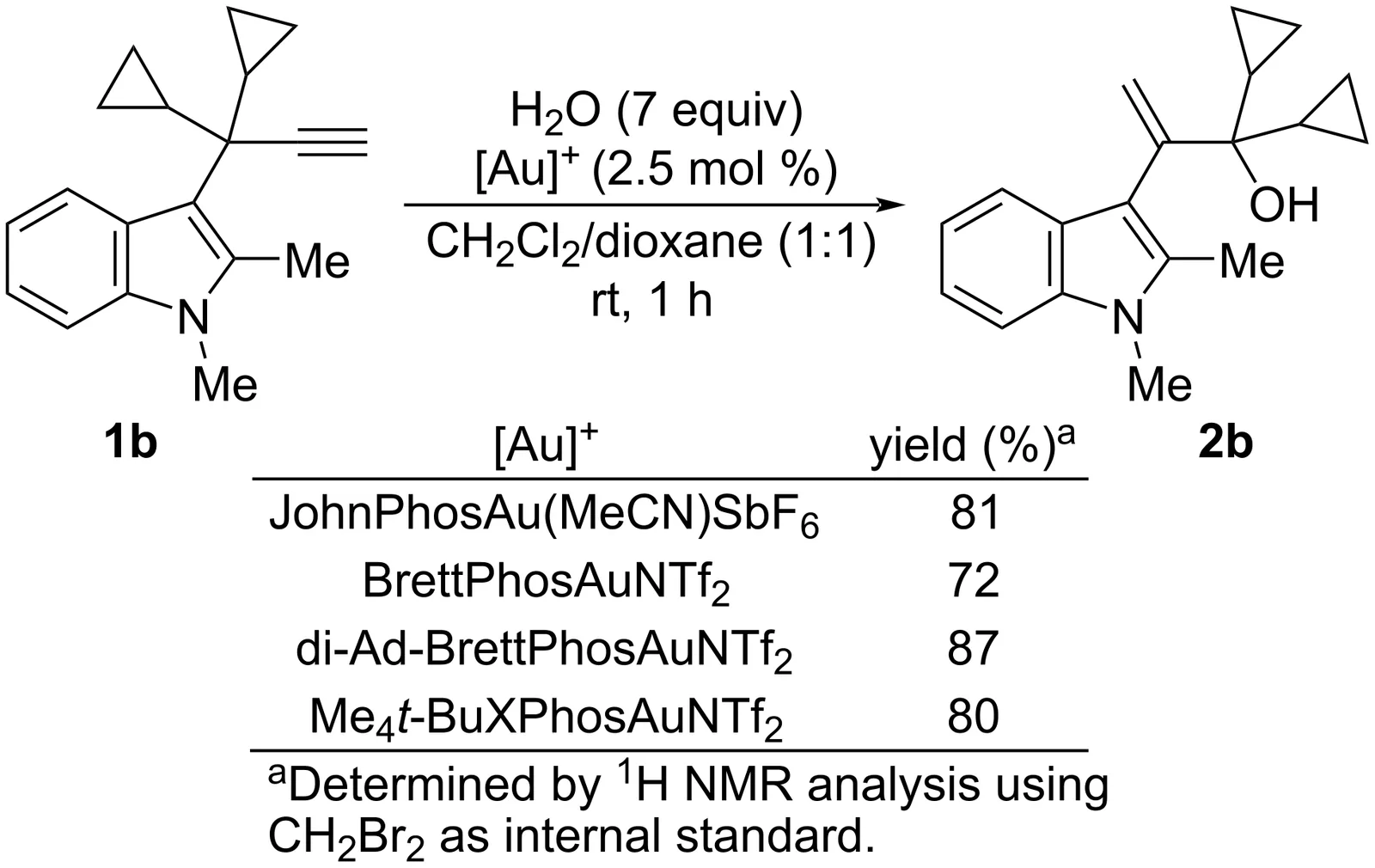
Further catalyst screening with **1b**.

As demonstrated in our previous studies, increased nucleophilicity of the indole nucleus favors the initial indole attack onto the activated alkyne and, consequently, the subsequent 1,2-indole migration leading to the corresponding alkenylgold carbene intermediate **I**. Accordingly, we decided to briefly investigate 3-propargylindole **1b**, bearing a more nucleophilic 1,2-dimethylindole moiety, using the most effective catalysts previously identified for substrate **1a** (Scheme 3). These experiments confirmed that the tandem 1,2-indole migration–hydroxyl attack process is more favorable for **1b**, allowing the use of a broader range of gold complexes as catalysts with lower catalyst loadings, while still providing similarly high yields of the expected alcohol **2b**.

Consequently, di-Ad-BrettPhosAuNTf_2_ (**B**) and Me_4_*t*-BuXPhosAuNTf_2_ (**C**), both bearing bulky ligands, were selected as the most active catalysts. In addition, the more readily available and less sterically hindered JohnPhosAu(MeCN)SbF_6_ (**A**) was also chosen for subsequent studies. We first explored the substrate scope of 3-propargylindoles **1a**–**w** featuring different substitution patterns on the indole nucleus (Table 2). Reactions of substrates bearing hydrogen, alkyl, or phenyl substituents at either the nitrogen atom or C2-position (**1a**–**i**) proceeded efficiently, yielding the corresponding tertiary alcohols **2a**–**i** in high yields (Table 2, entries 1–9). Next, substitution on the benzenoid ring was investigated using substrates derived from *N*–H, *N*-methyl and 1,2-dimethylindole cores. Halogenated starting materials (F, Cl, and Br) at the benzenoid ring reacted smoothly under the optimized conditions (Table 2, entries 10–14, 19–21, and 23). Similarly, electron-rich alkoxy-substituted starting indoles **1o**,**p** furnished the corresponding alcohols **2o**,**p** in high yields (Table 2, entries 15 and 16). Moreover, electron-withdrawing substituents, such as ester, at C5 and C6 positions were also well tolerated, as shown by the formation of indole derivatives **2q**,**r**,**v** (Table 2, entries 17, 18, and 22).

**Table 2:** Synthesis of hydroxy-functionalized indoles **2** from functionalized 3-propargylindoles **1a**–**w**.^a^

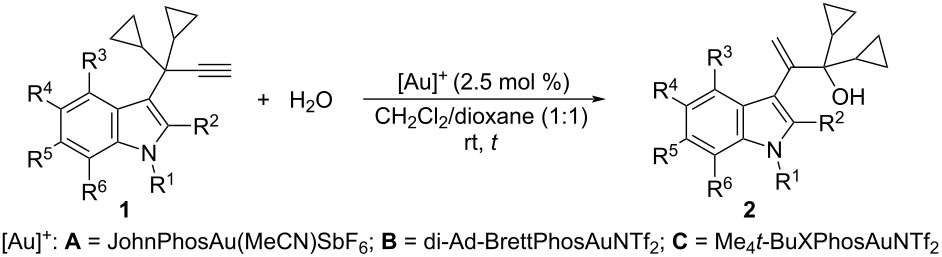											

Entry	**1**	R^1^	R^2^	R^3^	R^4^	R^5^	R^6^	[Au]^+^	Time (h)	**2**	Yield (%)^b^

1	**1a**	Me	H	H	H	H	H	**B**	3	**2a**	78^c^
2	**1b**	Me	Me	H	H	H	H	**B**	1	**2b**	83
3	**1c**	Me	Et	H	H	H	H	**B**	3	**2c**	91
4	**1d**	Me	Ph	H	H	H	H	**A**	5	**2d**	70
5	**1e**	H	H	H	H	H	H	**C**	1	**2e**	76^d^
6	**1f**	H	Me	H	H	H	H	**C**	4	**2f**	79^e^
7	**1g**	H	Et	H	H	H	H	**B**	6	**2g**	88
8	**1h**	H	Ph	H	H	H	H	**B**	24	**2h**	82
9	**1i**	Ph	H	H	H	H	H	**C**	3	**2i**	68
10	**1j**	Me	H	Br	H	H	H	**C**	3	**2j**	74
11	**1k**	Me	Me	H	F	H	H	**A**	1	**2k**	73
12	**1l**	Me	H	H	Cl	H	H	**B**	3	**2l**	78
13	**1m**	Me	H	H	Br	H	H	**B**	1	**2m**	81
14	**1n**	Me	Me	H	Br	H	H	**A**	1	**2n**	75
15	**1o**	Me	H	H	OMe	H	H	**B**	24	**2o**	86
16	**1p**	Me	Me	H	OMe	H	H	**B**	8	**2p**	71
17	**1q**	Me	H	H	CO_2_Me	H	H	**B**	1	**2q**	81
18	**1r**	H	H	H	CO_2_Me	H	H	**C**	3	**2r**	80
19	**1s**	H	H	H	H	Cl	H	**B**	24	**2s**	80
20	**1t**	Me	H	H	H	Cl	H	**B**	2	**2t**	83
21	**1u**	Me	H	H	H	Br	H	**B**	1	**2u**	79
22	**1v**	Me	H	H	H	CO_2_Me	H	**C**	3	**2v**	70
23	**1w**	H	H	H	H	H	Br	**C**	16	**2w**	75

^a^Reaction conditions: **1** (0.3 mmol), H_2_O (2.1 mmol, 38 μL), gold catalyst (0.0075 mmol) in 1:1 CH_2_Cl_2_/dioxane (3 mL) at rt. ^b^Isolated yield of the corresponding alcohol **2** referred to starting indole **1** after column chromatography. ^c^78% yield using catalyst **A** (*t* = 72 h). ^d^72% yield using catalyst **A** (*t* = 72 h). ^e^63% yield using catalyst **A** (*t* = 24 h).

**Scheme 4:**
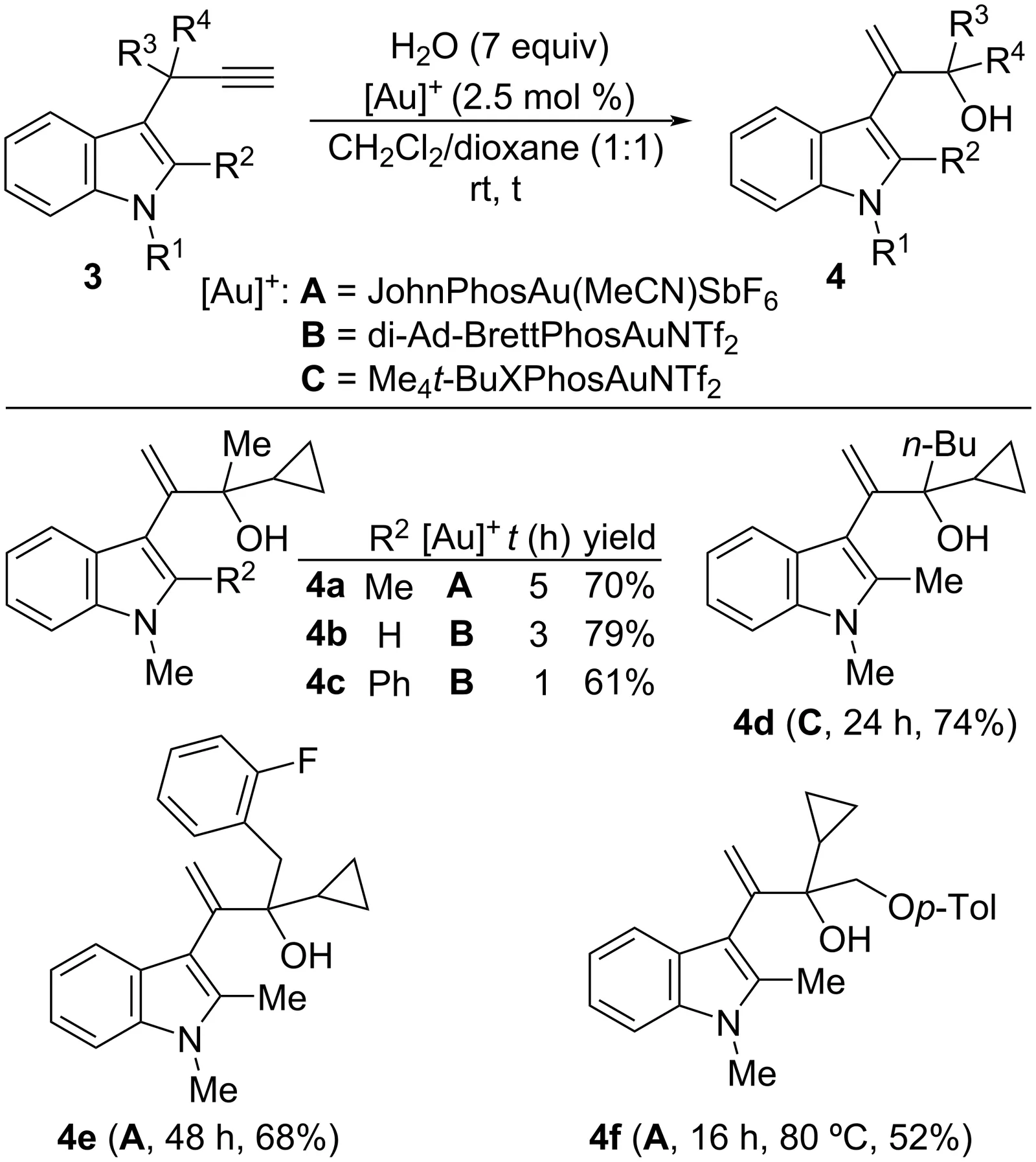
Substrate scope: variation of the propargylic position.

Additionally, we explored the reactivity of other 3-propargylindoles bearing different substituents at the propargylic position, considering that aryl substituents are not suitable due to the competing and highly favored iso-Nazarov reaction. First, substrates **3a**–**c**, possessing a methyl group at the propargylic position, reacted analogously to propargylindoles **1**, leading to the corresponding hydroxy-functionalized indole derivatives **4a**–**c** (Scheme 4). Similarly, substrates with butyl, benzyl, or aryloxymethyl substituents at the propargylic position (**3d**–**f**) also underwent the transformation smoothly, providing access to the corresponding alcohols **4d**–**f**. The presence of at least one cyclopropyl group at the propargylic position is primarily related to the preparation of the starting substrates rather than to a limitation of the gold-catalyzed transformation itself. Our synthetic route to the required 3-propargylindoles relies on a Brønsted acid-catalyzed substitution of indoles with propargylic alcohols. This process proceeds through a S_N_1-type mechanism and therefore requires suitably activated alcohols [30].

**Scheme 5:**
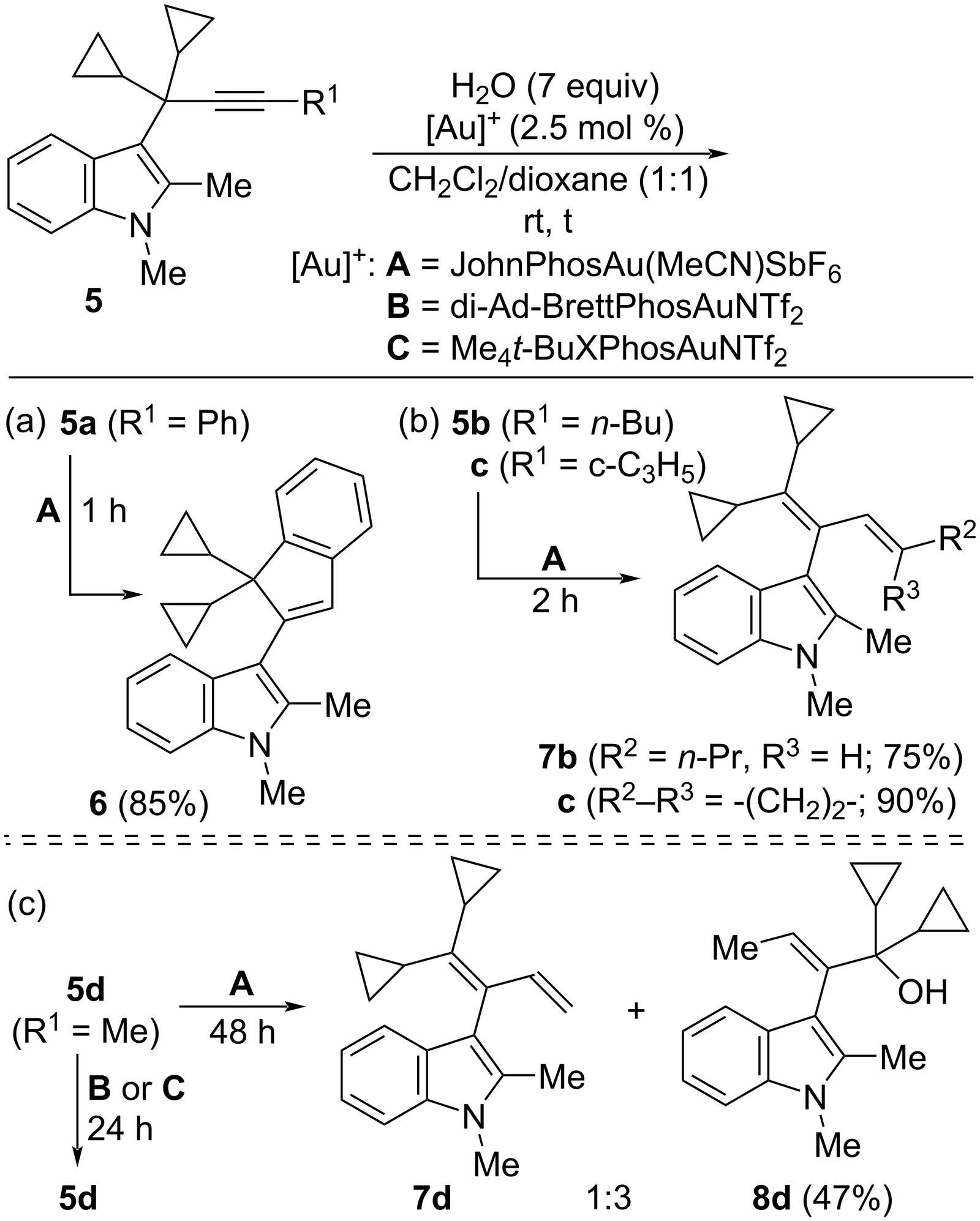
Limitations and reactivity of methyl-substituted alkyne **5d**.

After evaluating the reactivity of terminal alkynes **1** and **3**, we next synthesized a variety of substrates **5** based on internal alkynes (Scheme 5). As an expected limitation, the reaction of phenyl-substituted 3-propargylindole **5a** led exclusively to the formation of indenylindole **6**, derived from a tandem 1,2-indole migration–Nazarov cyclization sequence (Scheme 5a). Next, alkyl-substituted alkynes **5b**,**c** were evaluated under the optimized conditions; however, no incorporation of water was observed, and dienylindoles **7b,c** were exclusively obtained in high yields. In these cases, the 1,2-hydride shift pathway is favored over intermolecular hydroxyl attack (Scheme 5b). In contrast, when methyl-substituted substrate **5d** was subjected to the reaction conditions using JohnPhosAu(MeCN)SbF_6_ as catalyst, a ca 1:3 mixture of the corresponding dienylindole **7d** and the alcohol **8d** was obtained, allowing isolation of the hydroxylated indole in moderate yield as a single geometrical isomer. Surprisingly, the starting material was recovered upon using catalysts **B** or **C** with bulkier ligands, showing the unsuitability of these complexes for substrates bearing internal alkynes. Other gold catalysts tested, such as BrettPhosAuNTf_2_ and IPrAuNTf_2_, resulted in incomplete conversion together with lower yields and selectivity, whereas XPhosAuNTf_2_ led to appreciable decomposition.

**Scheme 6:**
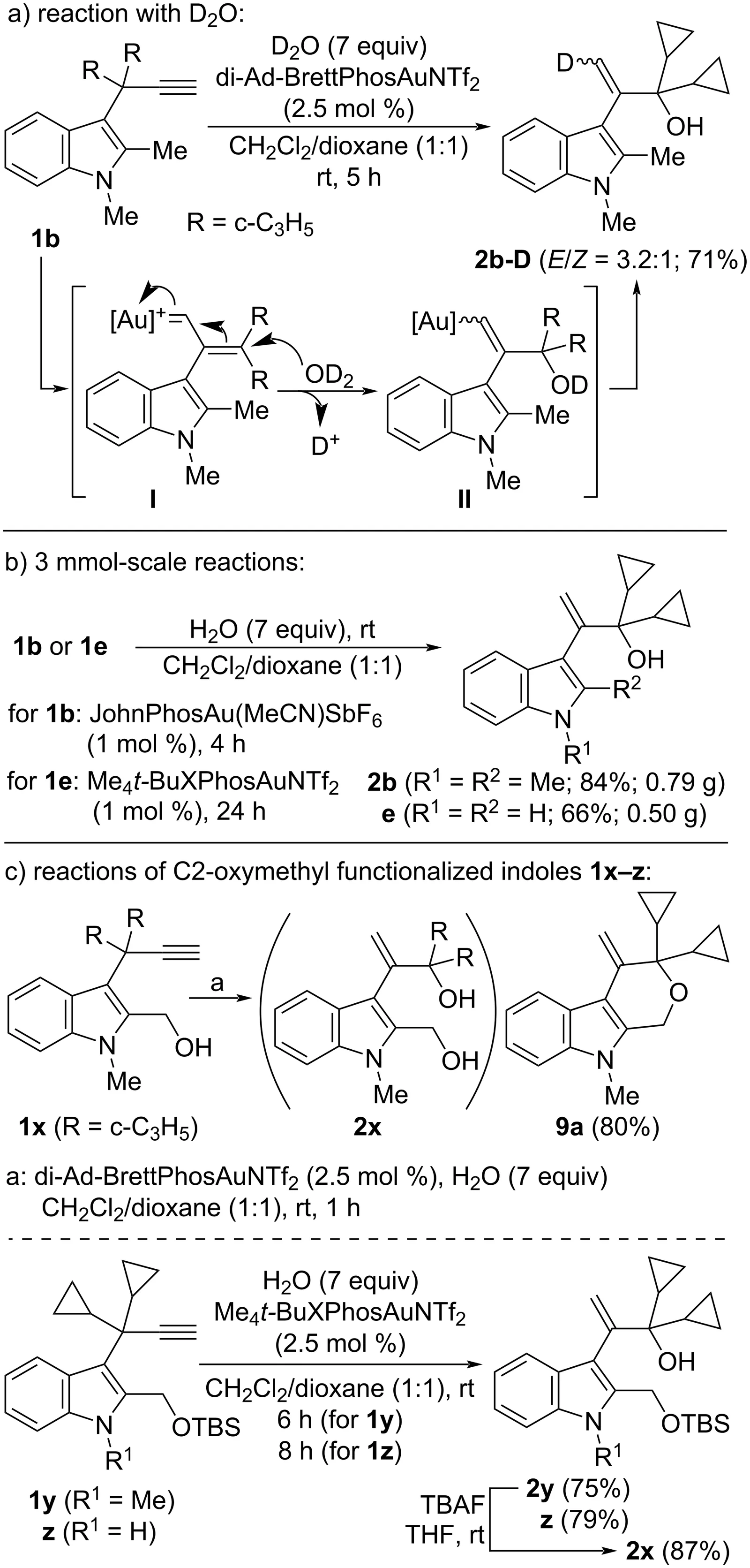
Further experiments: deuteration, gram-scale and competitive inter- vs intramolecular reaction.

At this point, we performed the reaction of **1b** with D_2_O, obtaining the corresponding deuterated indole derivative **2b-D** as a ca. 3:1 mixture of *E*/*Z* diastereoisomers, thus supporting our initial mechanistic proposal involving intermediates **I** and **II** (Scheme 6a). In addition, the scalability of the developed process was demonstrated through the synthesis of **2b** and **2e** on a 3 mmol scale. Notably, the catalyst loading could be reduced to 1 mol % while maintaining good efficiency (Scheme 6b). In addition, we decided to investigate whether hydroxyl attack onto intermediate carbene **I** is favored through an inter- or intramolecular pathway. To this end, C2-hydroxymethyl-substituted indole **1x** was subjected to the Au-catalyzed reaction in the presence of H_2_O. Under these conditions, the reaction led exclusively to the tetrahydropyrano[3,4-*b*]indole **9a**, with no traces of diol **2x**, the product expected from the intermolecular reaction with water (Scheme 6c). As anticipated, protection of the C2 hydroxy group in indoles **1y**,**z** enabled the formation of the corresponding alcohols **2y**,**z** via water incorporation. Subsequent TBAF-mediated desilylation of **2y** furnished diol **2x**, which was not accessible directly from **1x**.

**Scheme 7:**
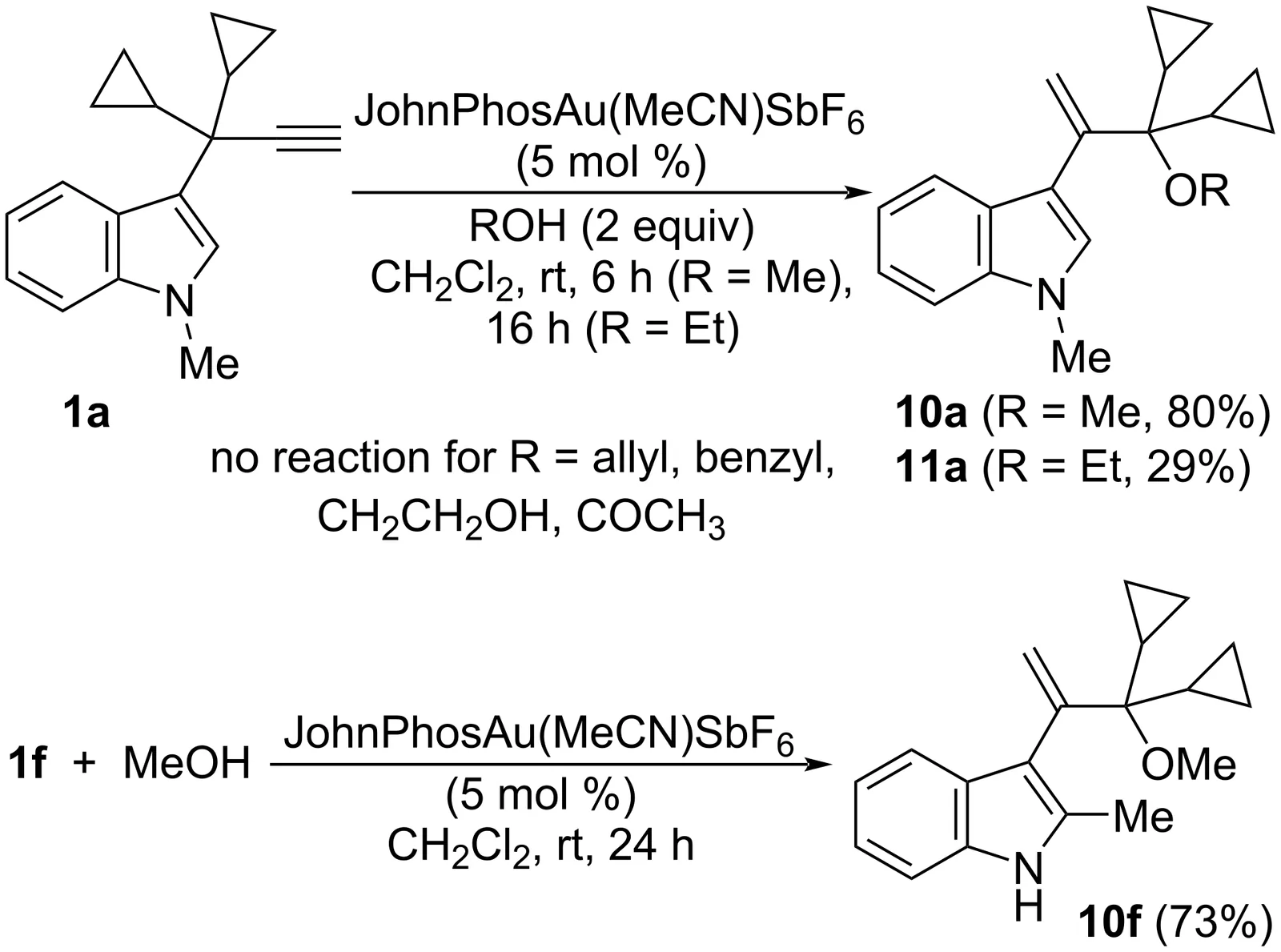
Alcohols as external nucleophiles. Synthesis of methoxy-functionalized indoles **10**.

Finally, other external nucleophiles, such as alcohols or amines and amides, were evaluated to test if they exhibit analogous behaviour to that of water. Gratifyingly, treatment of **1a** with MeOH, using JohnPhosAu(MeCN)SbF_6_ as the catalyst, afforded the methoxy-functionalized indole derivative **10a** in high yield. Among the other nucleophiles examined, EtOH provided the corresponding ethoxy-functionalized indole **11a**, albeit in low yield, whereas allyl alcohol, benzyl alcohol, ethyleneglycol, acetic acid, as well as *p*-toluidine and *p*-toluenesulfonamide, did not afford the corresponding functionalized products under the standard reaction conditions. Analogously, *N–*H indole **1f** afforded the corresponding indole derivative **10f** (Scheme 7).

## Conclusion

To sum up, we have developed a gold(I)-catalyzed tandem 1,2-indole migration–water attack reaction of 3-propargylindoles that exploits water as an external nucleophile. This transformation provides straightforward access to a synthetically useful range of hydroxy-functionalized indole derivatives through the selective intermolecular addition of water to the β-position of α,β-unsaturated gold carbene intermediates. Gold(I) complexes bearing bulky and electron-rich phosphine ligands proved to be excellent catalysts for this transformation. Notably, the reaction proceeds under mild conditions and is compatible with a variety of substitution patterns on both the indole core and the propargylic moiety. Mechanistic studies, including D_2_O-labeling and intramolecular competition experiments, strongly support the intermediacy of α,β-unsaturated gold carbene species and the preferential vinylogous attack of water. Furthermore, the practicality of the methodology was demonstrated through gram-scale reactions.

## Supporting Information

Experimental procedures and spectroscopic data for all new compounds. Copies of ^1^H NMR and ^13^C NMR spectra for new compounds.

File 1 Experimental and analytical data.

File 2 NMR spectra.

## Data Availability

Data generated and analyzed during this study will be openly available in Zenodo at https://doi.org/10.5281/zenodo.21673176  following an embargo period of two months from the date of publication.
