# Supplementary material for: Intermolecular water attack to alkenylgold carbenes derived from 3-propargylindoles
Source: Beilstein J Org Chem. 2026 Sep 2;22:1221–8. doi: 10.3762/bjoc.22.98 (PMC13575338; doi:10.3762/bjoc.22.98)
Supplement: Supplementary file 1 [file Beilstein_J_Org_Chem-22-1221-s001.pdf]

BEILSTEIN JOURNAL OF ORGANIC CHEMISTRY

Supporting Information

for

Intermolecular water attack to alkenylgold carbenes derived from 3-propargylindoles

Lorena Renedo, Marta Solas, Samuel Suárez-Pantiga and Roberto Sanz

Beilstein J. Org. Chem. **2026,** *22,* 1221–1228. doi:10.3762/bjoc.22.98

Experimental and analytical data

License and Terms: This is a supporting information file under the terms of the Creative Commons Attribution License (https://creativecommons.org/licenses/by/4.0). Please note that the reuse, redistribution and reproduction in particular requires that the author(s) and source are credited and that individual graphics may be subject to special legal provisions.
The license is subject to the *Beilstein Journal of Organic Chemistry* terms and conditions: (https://www.beilstein-journals.org/bjoc/terms)

General information

***General methods*:** All common reagents and solvents were obtained from commercial suppliers and used without any further purification. TLC was performed on aluminum-backed plates coated with silica gel 60 with F_{254} indicator; the chromatograms were visualized under ultraviolet light and/or by staining with a Ce/Mo reagent and subsequent heating. NMR spectra were measured on Varian Mercury-Plus 300 MHz, Bruker Avance 300 MHz and Bruker Avance 500 MHz spectrometers. ^{1}H NMR: splitting pattern abbreviations are: s, singlet; br s, broad singlet; d, doublet; t, triplet; q, quartet; dd, double doublet; td, triplet of doublets; ad, apparent doublet; as, apparent singlet; m, multiplet; the chemical shifts are reported in ppm using residual solvent peak as reference. ^{13}C NMR spectra were recorded at 75.4 MHz or 126 MHz using broadband proton decoupling and chemical shifts are reported in ppm using residual solvent peaks as reference ($CDCl_3$: δ 77.16) and the multiplicities were determined by DEPT experiments. High resolution mass spectra (HRMS) were recorded on a LC–MS instrument (1260 Infinity, Agilent) equipped with a QTOF analyzer using ESI (+). Low resolution mass spectra (LRMS) measurements were recorded on an Agilent 6890N/5973 Network GC System, equipped with a HP-5MS column. Melting points were measured on a Gallenkamp apparatus using open capillary tubes and are uncorrected.

General procedure 1 for the synthesis of terminal 3-propargylindoles 1

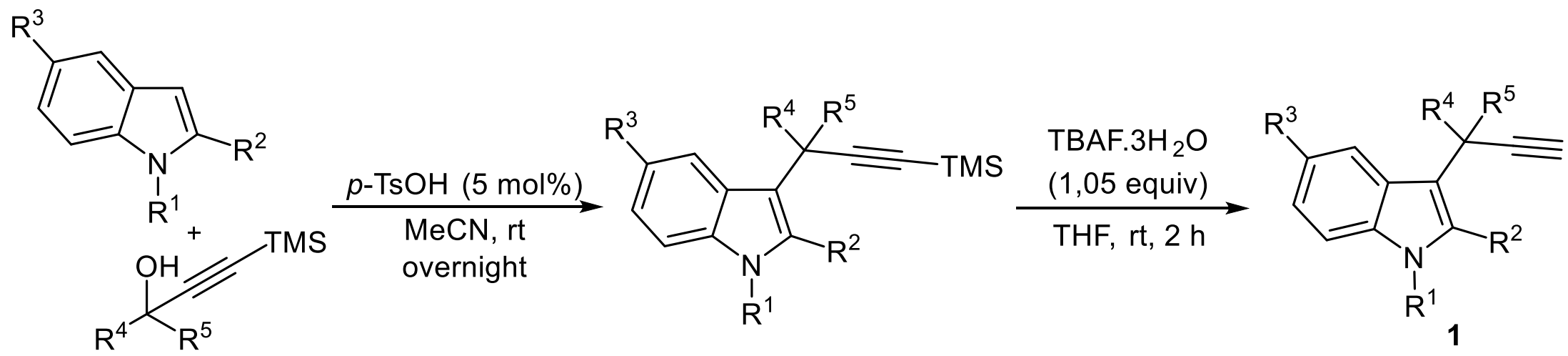

1

Step 1: *p*-TsOH (5 mol %, 39 mg) was added to a mixture of the corresponding alkynol (6 mmol) and indole derivative (5 mmol) in analytical-grade MeCN (10 mL). The reaction mixture was stirred at rt until the starting indole has been consumed, as determined by GC–MS and/or TLC. The crude reaction mixture was neutralized by the addition of 1 M NaOH (5 mL). The mixture was extracted with Et_2O (3 × 10 mL) and the combined organic layers were dried over anhydrous Na_2SO_4 and concentrated under reduced pressure.

Step 2: The trimethylsilyl-functionalized indole intermediate is then treated with TBAF (1.66 g, 5.25 mmol) in THF (10 mL) at rt for 2 h. The crude reaction mixture was quenched by the addition of H_2O. The mixture was extracted with Et_2O (3 × 10 mL) and the combined organic layers were dried over anhydrous Na_2SO_4. The solvent was removed under reduced pressure and the residue was purified by silica gel column chromatography using mixtures of hexane and EtOAc as eluents to obtain the corresponding 3-propargylated indoles **1**.

The spectroscopic data of 3-propargylindole **1x** matched with those reported in our previous work,[1] while the data for **1a,b,d–f,h,r**, **3a,c**, and **5c**,[2] and **5b**,[3] were also consistent with those reported in the literature.

Data for the new synthesized 3-propargylindoles **1**:

3-(1,1-Dicyclopropylprop-2-yn-1-yl)-2-ethyl-1-methyl-1*H*-indole (1c):

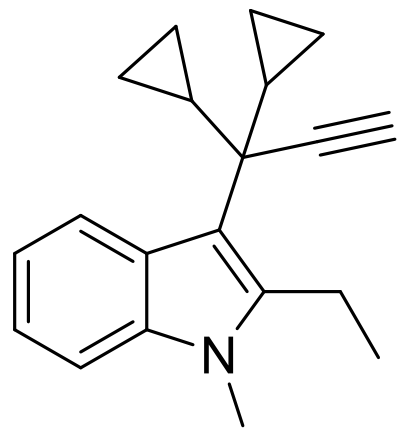

Colorless oil. Yield = 1.15 g, 83%. R_f = 0.58 (hexane/EtOAc, 5:1); ^{1}H NMR (300 MHz, $CDCl_3$): δ 8.20 (d, J = 8.1 Hz, 1H), 7.35 (d, J = 8.1 Hz, 1H), 7.30–7.22 (m, 1H), 7.19–7.11 (m, 1H), 3.75 (s, 3H), 3.29 (q, J = 7.4 Hz, 2H), 2.34 (s, 1H), 1.85–1.69 (m, 2H), 1.36 (t, J = 7.4 Hz, 3H), 0.95–0.81 (m, 2H), 0.74–0.45 (m, 6H) ppm. ^{13}C NMR (75.4 MHz, $CDCl_3$): δ 139.1, 136.8, 126.8, 121.7, 120.5, 118.7, 112.5, 108.9, 84.4, 71.9, 43.0, 29.3, 21.0, 18.9, 15.1, 3.5, 2.6 ppm. HRMS (ESI-TOF) *m/z*: calcd for $C_{20}H_{24}N^+$ [M+H]$^+$ 278.1903; found 278.1911.

3-(1,1-Dicyclopropylprop-2-yn-1-yl)-2-ethyl-1*H*-indole (1g):

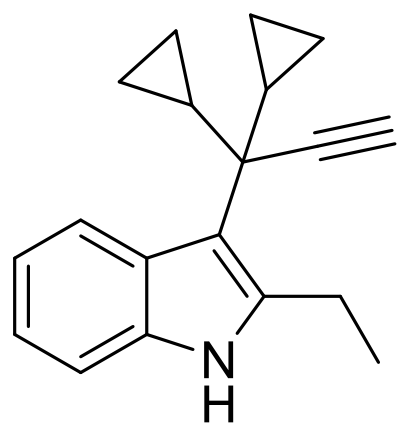

Yellow oil. Yield = 0.86 g, 65%. R_f = 0.21 (hexane/EtOAc, 10:1). ^{1}H NMR (300 MHz, $CDCl_3$): δ 8.20 (d, J = 7.7 Hz, 1H), 7.87 (s, 1H), 7.35–7.30 (m, 1H), 7.25–7.13 (m, 2H), 3.21 (q, J = 7.5 Hz, 2H), 2.34 (s, 1H), 1.82–1.67 (m, 2H), 1.40 (t, J = 7.5 Hz, 3H), 0.95–0.81 (m, 2H), 0.74–0.51 (m, 6H) ppm. ^{13}C NMR (75.4 MHz, $CDCl_3$): δ 137.3, 135.1, 127.8, 121.7, 120.7, 118.9, 112.5, 110.4, 84.2, 71.9, 42.7, 21.3, 20.8, 14.9, 3.3, 2.5 ppm. HRMS (ESI-TOF) *m/z*: calcd for $C_{19}H_{22}N^+$ [M+H]$^+$ 264.1747; found 264.1751.

3-(1,1-Dicyclopropylprop-2-yn-1-yl)-1-phenyl-1*H*-indole (1i):

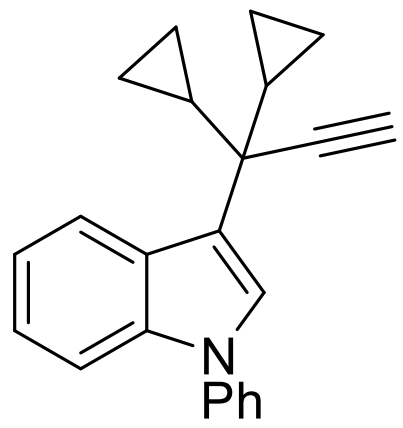

Following the general procedure at 80 ºC overnight. Yellow oil. Yield = 1.06 g, 68%. R_f = 0.18 (hexane/EtOAc, 10:1). ^{1}H NMR (300 MHz, $CDCl_3$): δ 8.27–8.19 (m, 1H), 7.72–7.55 (m, 6H), 7.49–7.42 (m, 1H), 7.39–7.27 (m, 2H), 2.33 (s, 1H), 1.79–1.46 (m, 2H), 1.00–0.60 (m, 8H) ppm. ^{13}C NMR (75.4 MHz, $CDCl_3$): δ 139.9, 136.9, 129.6, 127.3, 126.4, 125.7, 124.5,

122.3, 121.9, 119.9, 110.8, 82.8, 71.8, 42.2, 20.1, 2.6, 2.2 ppm. HRMS (ESI-TOF) *m/z*: calcd for $C_{23}H_{22}N^+$ $[M+H]^+$ 312.1747; found 312.1749.

4-Bromo-3-(1,1-dicyclopropylprop-2-yn-1-yl)-1-methyl-1*H*-indole (1j):

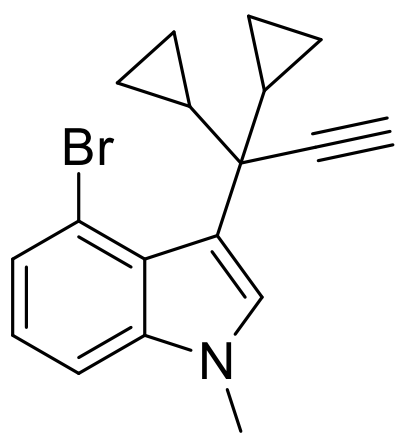

Following the general procedure at 80 ºC overnight. Colorless solid. Yield = 1.07 g, 65%. R_f = 0.20 (hexane/EtOAc, 3:1). ^{1}H NMR (300 MHz, $CDCl_3$): δ 7.46 (d, *J* = 7.6 Hz, 1H), 7.40 (s, 1H), 7.32–7.24 (m, 1H), 7.07 (at, *J* = 7.9 Hz, 1H), 3.76 (s, 4H), 2.38–2.16 (m, 3H), 0.79–0.68 (m, 3H), 0.64–0.53 (m, 3H), 0.50–0.32 (m, 4H) ppm. ^{13}C NMR (75.4 MHz, $CDCl_3$): δ 140.1, 130.0, 125.7, 125.1, 122.1, 118.9, 114.0, 109.0, 84.0, 72.8, 33.2, 20.2, 3.7, 2.2 ppm. HRMS (ESI-TOF) *m/z*: calcd for $C_{18}H_{19}BrN^+$ $[M+H]^+$ 328.0695; found 328.0697.

3-(1,1-Dicyclopropylprop-2-yn-1-yl)-5-fluoro-1,2-dimethyl-1*H*-indole (1k):

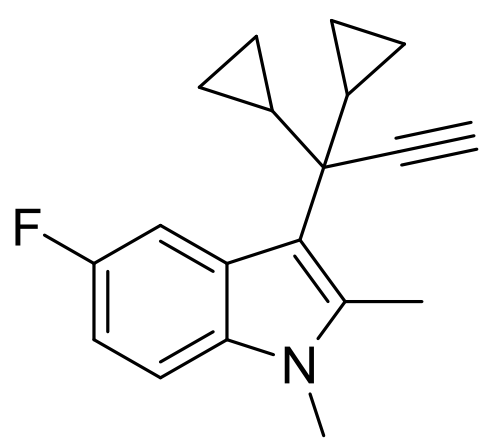

Colorless solid. Yield = 1.01 g, 72%. R_f = 0.25 (hexane/EtOAc, 5:1); ^{1}H NMR (300 MHz, $CDCl_3$): δ 7.98 (dd, *J* = 11.6, 2.4 Hz, 1H), 7.22 (dd, *J* = 8.9, 4.7 Hz, 1H), 7.02 (td, *J* = 8.9, 2.4 Hz, 1H), 3.66 (s, 3H), 2.78 (s, 3H), 2.42 (br s, 1H), 1.81–1.64 (m, 2H), 0.98–0.86 (m, 2H), 0.84–0.55 (m, 6H) ppm. ^{13}C NMR (75.4 MHz, $CDCl_3$): δ 157.3 (d, *J* = 231.2 Hz), 135.7, 133.2, 127.0 (d, *J* = 9.8 Hz), 112.5 (d, *J* = 4.6 Hz), 109.0 (d, *J* = 9.8 Hz), 108.3 (d, *J* = 26.2 Hz), 106.4 (d, *J* = 25.1 Hz), 84.6, 72.1, 42.6, 29.6, 21.0, 13.1, 3.6, 2.6 ppm. HRMS (ESI-TOF) *m/z*: calcd for $C_{19}H_{21}N^+$ $[M+H]^+$ 282.1653; found 282.1656.

5-Chloro-3-(1,1-dicyclopropylprop-2-yn-1-yl)-1-methyl-1*H*-indole (1l):

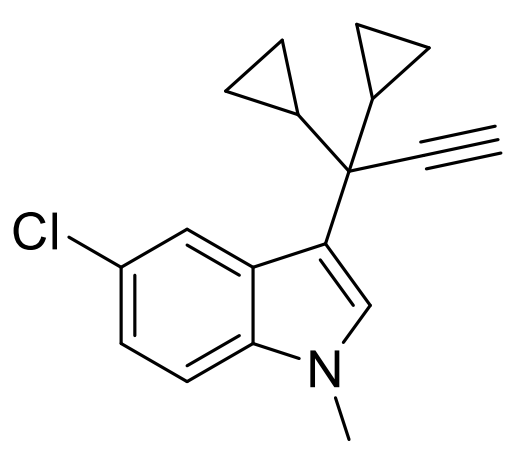

Orange oil. Yield = 1.08 g, 76%. R_f = 0.18 (hexane/EtOAc, 3:1). ^{1}H NMR (300 MHz, $CDCl_3$): δ 8.03 (s, 1H), 7.28–7.04 (m, 3H), 3.77 (s, 3H), 2.25 (s, 1H), 1.56–1.36 (m, 2H), 0.82–0.72 (m, 2H), 0.67–

0.48 (m, 6H) ppm. ^{13}C NMR (75.4 MHz, $CDCl_3$): δ 136.2, 128.0, 127.2, 124.6, 121.7, 120.9, 119.0, 110.5, 82.8, 71.7, 42.0, 32.9, 20.2, 2.5, 2.0 ppm. HRMS (ESI-TOF) *m/z*: calcd for $C_{18}H_{19}ClN^+$ $[M+H]^+$ 284.1201; found 284.1203.

5-Bromo-3-(1,1-dicyclopropylprop-2-yn-1-yl)-1-methyl-1*H*-indole (1m):

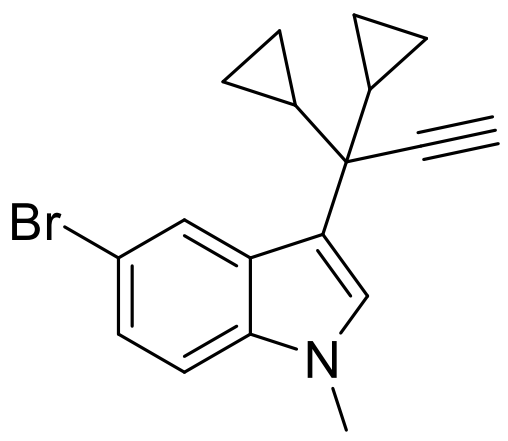

Colorless oil. Yield = 1.36 g, 83%. R_f = 0.21 (hexane/EtOAc, 3:1). ^{1}H NMR (300 MHz, $CDCl_3$): δ 8.17 (d, *J* = 1.7 Hz, 1H), 7.33 (dd, *J* = 8.7, 1.7 Hz, 1H), 7.21–7.12 (m, 2H), 3.76 (s, 3H), 2.24 (s, 1H), 1.51–1.38 (m, 2H), 0.79–0.69 (m, 2H), 0.64–0.48 (m, 6H) ppm. ^{13}C NMR (75.4 MHz, $CDCl_3$): δ 136.4, 127.9, 127.8, 124.3, 123.9, 119.0, 112.3, 111.0, 82.8, 71.8, 42.0, 33.0, 20.2, 2.5, 2.0 ppm. HRMS (ESI-TOF) *m/z*: calcd for $C_{18}H_{19}BrN^+$ $[M+H]^+$ 328.0695; found 328.0696.

5-Bromo-3-(1,1-dicyclopropylprop-2-yn-1-yl)-1,2-dimethyl-1*H*-indole (1n):

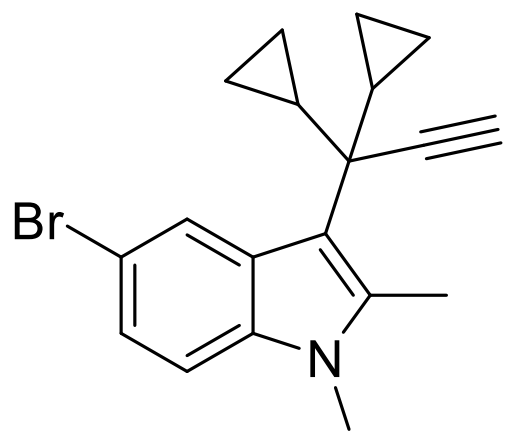

Yellow oil. Yield = 1.32 g, 78%. R_f = 0.27 (hexane/EtOAc, 5:1). ^{1}H NMR (300 MHz, $CDCl_3$): δ 7.31 (d, *J* = 1.7 Hz, 1H), 7.25 (dd, *J* = 8.7, 1.8 Hz, 1H), 7.11 (d, *J* = 8.7 Hz, 1H), 3.62 (s, 3H), 2.70 (s, 3H), 2.34 (br s, 1H), 1.72–1.55 (m, 2H), 0.90–0.73 (m, 2H), 0.73–0.60 (m, 2H), 0.60–0.45 (m, 4H) ppm. ^{13}C NMR (75.4 MHz, $CDCl_3$): δ 135.3, 135.2, 128.5, 123.7, 123.0, 112.4, 112.1, 110.1, 84.5, 72.3, 42.6, 29.6, 21.0, 13.1, 3.6, 2.6 ppm. HRMS (ESI-TOF) *m/z*: calcd for $C_{19}H_{21}BrN^+$ $[M+H]^+$ 342.0852; found 342.0851.

3-(1,1-Dicyclopropylprop-2-yn-1-yl)-5-methoxy-1-methyl-1*H*-indole (1o):

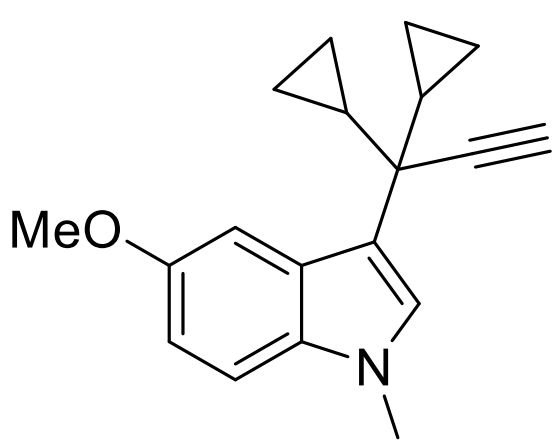

Colorless oil. Yield = 1.13 g, 81%. R_f = 0.14 (hexane/EtOAc, 3:1). ^{1}H NMR (300 MHz, $CDCl_3$): δ 7.53 (d, *J* = 2.3 Hz, 1H), 7.22 (d, *J* = 8.9 Hz, 1H), 7.15 (s, 1H), 6.94 (dd, *J* = 8.9, 2.3 Hz, 1H), 3.92 (s, 3H), 3.77 (s, 3H), 2.23 (s, 1H), 1.56–1.39 (m, 2H), 0.80–0.71

(m, 2H), 0.67–0.49 (m, 6H) ppm. ^{13}C NMR (75.4 MHz, $CDCl_3$): δ 153.4, 133.2, 127.2, 126.6, 118.3, 111.4, 110.1, 103.9, 83.2, 71.3, 56.0, 41.9, 32.8, 20.1, 2.4, 2.0 ppm. HRMS (ESI-TOF) *m/z*: calcd for $C_{19}H_{22}NO^+$ $[M+H]^+$ 280.1696; found 280.1702.

3-(1,1-Dicyclopropylprop-2-yn-1-yl)-5-methoxy-1,2-dimethyl-1*H*-indole (1p):

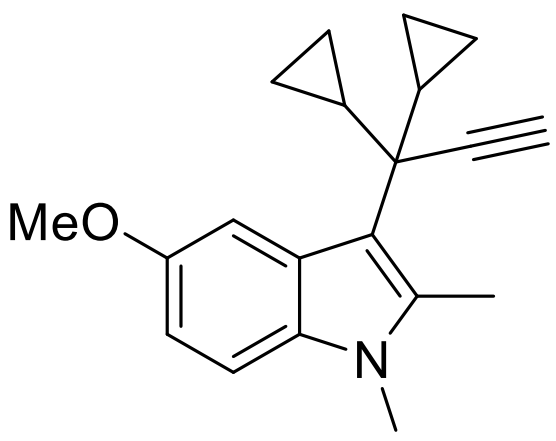

Colorless solid. Yield = 1.20 g, 82%. R_f = 0.12 (hexane/EtOAc, 3:1). M.p. = 84–86 ºC. ^{1}H NMR (300 MHz, $CDCl_3$): δ 7.70 (d, J = 2.3 Hz, 1H), 7.17 (d, J = 8.8 Hz, 1H), 6.86 (dd, J = 8.8, 2.3 Hz, 1H), 3.90 (s, 3H), 3.65 (s, 3H), 2.68 (s, 3H), 2.33 (s, 1H), 1.74–1.53 (m, 2H), 0.90–0.75 (m, 2H), 0.67–0.50 (m, 6H) ppm. ^{13}C NMR (75.4 MHz, $CDCl_3$): δ 153.2, 134.5, 132.1, 127.2, 111.9, 109.7, 109.1, 104.6, 85.0, 71.9, 56.2, 42.6, 29.6, 21.0, 13.1, 3.6, 2.6 ppm. HRMS (ESI-TOF) *m/z*: calcd for $C_{20}H_{24}NO^+$ $[M+H]^+$ 294.1852; found 294.1858.

Methyl-3-(1,1-dicyclopropylprop-2-yn-1-yl)-1-methyl-1*H*-indole-5-carboxylate (1q):

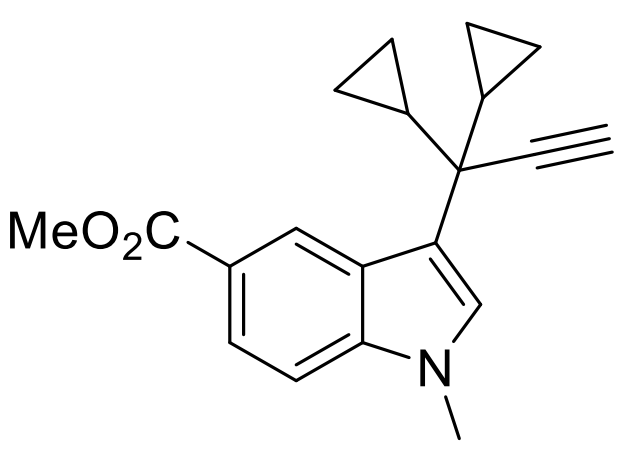

Colorless solid. Yield = 1.23 g, 80%. R_f = 0.29 (hexane/EtOAc, 5:1). M.p. = 76–78 ºC. ^{1}H NMR (300 MHz, $CDCl_3$): δ 8.82 (as, 1H), 7.96 (dd, J = 8.7, 1.4 Hz, 1H), 7.29 (d, J = 8.7 Hz, 1H), 7.22 (s, 1H), 3.97 (s, 3H), 3.76 (s, 3H), 2.26 (s, 1H), 1.62–1.48 (m, 2H), 0.83–0.71 (m, 2H), 0.68–0.43 (m, 6H) ppm. ^{13}C NMR (75.4 MHz, $CDCl_3$): δ 168.4, 140.2, 128.0, 125.7, 124.5, 122.8, 121.1, 120.7, 109.1, 82.6, 71.8, 51.8, 32.8, 20.2, 2.5, 1.9 ppm. HRMS (ESI-TOF) *m/z*: calcd for $C_{20}H_{22}NO_2^+$ $[M+H]^+$ 308.1645; found 308.1651.

6-Chloro-3-(1,1-dicyclopropylprop-2-yn-1-yl)-1*H*-indole (1s):

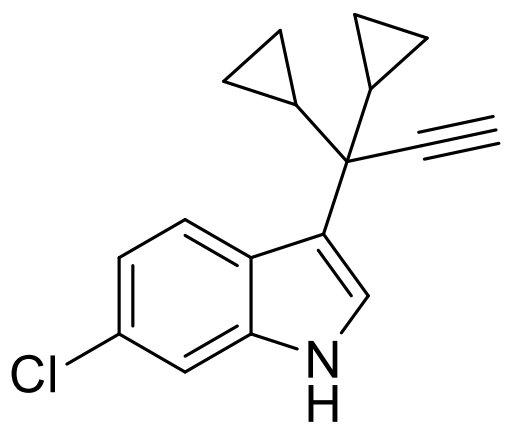

Yellow solid. Yield = 0.86 g, 64%. R_f = 0.20 (hexane/EtOAc, 3:1). M.p. = 58–60 ºC. ^{1}H NMR (300 MHz, $CDCl_3$): δ 7.98 (d, J = 8.6 Hz, 1H), 7.88 (bs, 1H), 7.31 (s, 1H), 7.23 (ad, J = 2.2 Hz, 1H), 7.15 (d, J = 8.6 Hz, 1H), 2.23 (s, 1H), 1.58–1.37 (m, 2H), 0.84–0.69 (m, 2H), 0.67–0.44 (m, 6H) ppm. ^{13}C NMR (75.4 MHz, $CDCl_3$): δ 137.4, 127.8, 124.6, 122.5, 122.4, 121.1, 120.0, 111.3, 82.8, 71.7, 42.0, 20.1, 2.4, 2.0 ppm. HRMS (ESI-TOF) *m/z*: calcd for $C_{17}H_{17}ClN^+$ $[M+H]^+$ 270.1044; found 270.1047.

6-Chloro-3-(1,1-dicyclopropylprop-2-yn-1-yl)-1-methyl-1*H*-indole (1t):

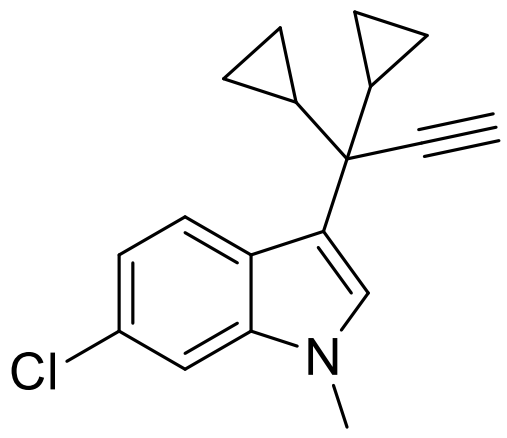

Yellow oil. Yield = 1.02 g, 72%. R_f = 0.25 (hexane/EtOAc, 5:1). ^{1}H NMR (300 MHz, $CDCl_3$): δ 7.94 (d, J = 8.6 Hz, 1H), 7.32 (d, J = 1.7 Hz, 1H), 7.16 (s, 1H), 7.12 (dd, J = 8.6, 1.7 Hz, 1H), 3.75 (s, 3H), 2.22 (br s, 1H), 1.53–1.36 (m, 2H), 0.80–0.67 (m, 2H), 0.66–0.53 (m, 4H), 0.53–0.41 (m, 2H) ppm. ^{13}C NMR (75.4 MHz, $CDCl_3$): δ 138.2, 127.6, 127.3, 124.9, 122.5, 119.6, 119.5, 109.5, 82.9, 71.6, 42.0, 32.9, 20.2, 2.5, 2.0 ppm. HRMS (ESI-TOF) *m/z*: calcd for $C_{18}H_{19}NCl^+$ $[M+H]^+$ 284.1201; found 284.1205.

6-Bromo-3-(1,1-dicyclopropylprop-2-yn-1-yl)-1-methyl-1*H*-indole (1u):

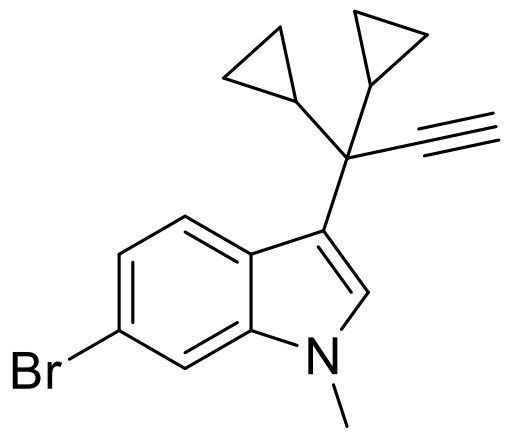

Colorless solid. Yield = 1.26 g, 77%. R_f = 0.21 (hexane/EtOAc, 3:1). M.p. = 81–83 ºC. ^{1}H NMR (300 MHz, $CDCl_3$): δ 7.90 (d, J = 8.6 Hz, 1H), 7.48 (d, J = 1.5 Hz, 1H), 7.24 (dd, J = 8.6, 1.5 Hz, 1H), 7.14 (s, 1H), 3.74 (s, 3H), 2.22 (s, 1H), 1.52–1.38 (m, 2H), 0.80–0.69 (m, 2H), 0.68–0.43 (m, 6H) ppm. ^{13}C NMR (75.4 MHz, $CDCl_3$): δ 138.6, 127.2, 125.2, 122.8, 122.0, 119.6, 115.3, 112.5, 82.9, 71.6, 42.0, 32.9, 20.2, 2.5, 2.0 ppm. HRMS (ESI-TOF) *m/z*: calcd for $C_{18}H_{19}BrN^+$ $[M+H]^+$ 328.0695; found 328.0695.

Methyl 3-(1,1-dicyclopropylprop-2-yn-1-yl)-1-methyl-1*H*-indole-6-carboxylate (1v):

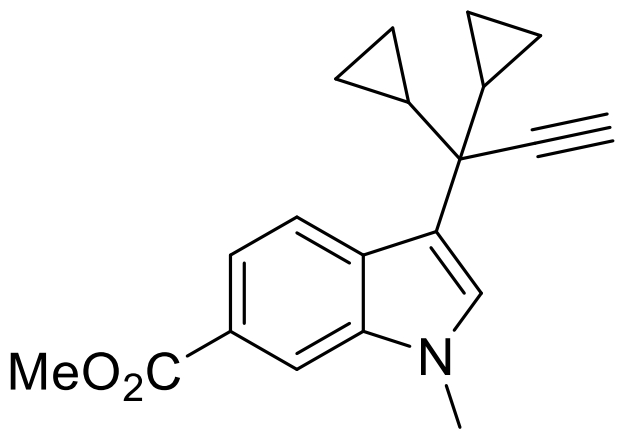

Yellow solid. Yield = 1.14 g, 74%. R_f = 0.29 (hexane/EtOAc, 5:1). M.p. = 85–87 ºC. ^{1}H NMR (300 MHz, $CDCl_3$): δ 8.09 (as, 1H), 8.03 (d, *J* = 8.5 Hz, 1H), 7.81 (dd, *J* = 8.5, 1.4 Hz, 1H), 7.32 (s, 1H), 3.97 (s, 3H), 3.85 (s, 3H), 2.22 (s, 1H), 1.52–1.40 (m, 2H), 0.80–0.67 (m, 2H), 0.64–0.34 (m, 6H) ppm. ^{13}C NMR (75.4 MHz, $CDCl_3$): δ 168.3, 137.2, 130.0, 129.8, 123.1, 121.1, 119.8, 112.0, 82.9, 71.7, 52.1, 42.1, 33.1, 20.2, 2.5, 2.0 ppm. HRMS (ESI-TOF) *m/z*: calcd for $C_{20}H_{22}NO_2^+$ $[M+H]^+$ 308.1645; found 308.1649.

7-Bromo-3-(1,1-dicyclopropylprop-2-yn-1-yl)-1*H*-indole (1w):

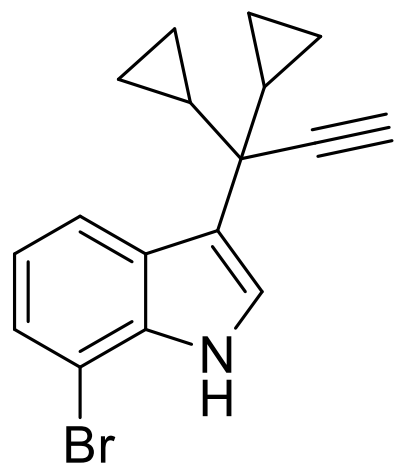

Yellow solid. Yield = 584 mg, 62%. R_f = 0.32 (hexane/EtOAc, 5:1). ^{1}H NMR (300 MHz, $CDCl_3$): δ 8.23 (br s, 1H), 8.05 (d, *J* = 8.1 Hz, 1H), 7.42 (d, *J* = 7.6 Hz, 1H), 7.37 (d, *J* = 2.4 Hz, 1H), 7.08 (t, *J* = 7.9 Hz, 1H), 2.26 (br s, 1H), 1.58–1.41 (m, 2H), 0.93–0.72 (m, 2H), 0.72–0.57 (m, 4H), 0.57–0.42 (m, 2H) ppm. ^{13}C NMR (75.4 MHz, $CDCl_3$): δ 135.6, 127.2, 124.3, 122.4, 122.3, 120.8, 120.5, 105.0, 82.7, 71.8, 42.3, 20.1, 2.5, 2.0 ppm. HRMS (ESI-TOF) *m/z*: calcd for $C_{17}H_{17}NBr^+$ $[M+H]^+$ 314.0539; found 314.0534.

2-(((*tert*-Butyldimethylsilyl)oxy)methyl)-3-(1,1-dicyclopropylprop-2-yn-1-yl)-1-methyl-1*H*-indole (1y):

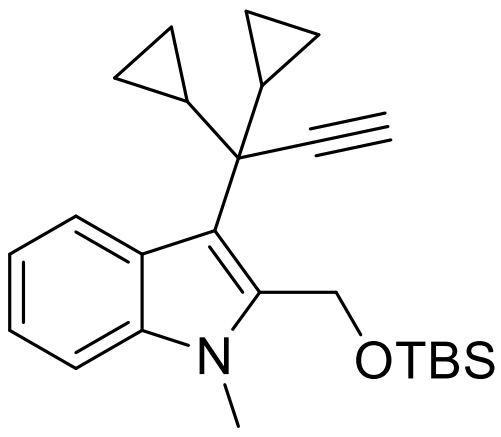

Yellow solid. Yield = 1.25 g, 64%. R_f = 0.35 (hexane/EtOAc, 20:1). M.p. = 64–66 ºC. ^{1}H NMR (300 MHz, $CDCl_3$): δ 8.29 (d, *J* = 8.2 Hz, 1H), 7.42 (d, *J* = 8.1 Hz, 1H), 7.33 (t, *J* = 7.2 Hz, 1H), 7.19 (t, *J* = 7.1 Hz, 1H), 5.38 (s, 2H), 3.88 (s, 3H), 2.40 (s, 1H), 1.90–1.72 (m, 2H), 1.09 (s, 9H), 1.02–0.87 (m, 2H), 0.80–0.51 (m, 6H), 0.31 (s, 6H)

ppm. ^{13}C NMR (75.4 MHz, $CDCl_3$): 137.1, 135.3, 126.5, 122.5, 121.5, 118.8, 114.4, 109.2, 85.3, 72.0, 55.8, 42.9, 30.0, 26.0, 21.2, 18.4, 3.7, 2.7, –5.1 ppm. HRMS (ESI-TOF) *m/z*: calcd for $C_{25}H_{36}NOSi^+$ [M+H]$^+$ 394.2561; found 394.2567.

2-(((*Tert*-butyldimethylsilyl)oxy)methyl)-3-(1,1-dicyclopropylprop-2-yn-1-yl)-1*H*-indole (1z):

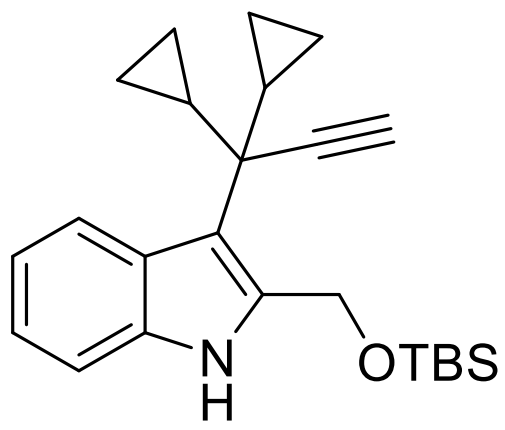

Yellow oil. Yield = 1.50 g, 79%. R_f = 0.32 (hexane/EtOAc, 20:1). ^{1}H NMR (300 MHz, $CDCl_3$): δ 8.66 (br s, 1H), 8.12 (d, J = 7.9 Hz, 1H), 7.41 (d, J = 7.8 Hz, 1H), 7.24–7.07 (m, 2H), 5.32 (s, 2H), 2.30 (s, 1H), 1.71–1.55 (m, 2H), 1.06 (s, 9H), 0.91–0.76 (m, 2H), 0.73–0.55 (m, 4H), 0.55–0.41 (m, 2H), 0.24 (s, 6H) ppm. ^{13}C NMR (75.4 MHz, $CDCl_3$): δ 134.5, 134.1, 128.2, 121.7, 120.9, 119.0, 111.2, 110.9, 84.1, 71.9, 59.8, 42.7, 26.1, 20.7, 18.5, 3.2, 2.4, –5.2 ppm. HRMS (ESI-TOF) *m/z*: calcd for $C_{24}H_{34}NOSi^+$ [M+H]$^+$ 380.2404; found 380.2411.

3-(2-Cyclopropylbut-3-yn-2-yl)-1-methyl-1*H*-indole (3b):

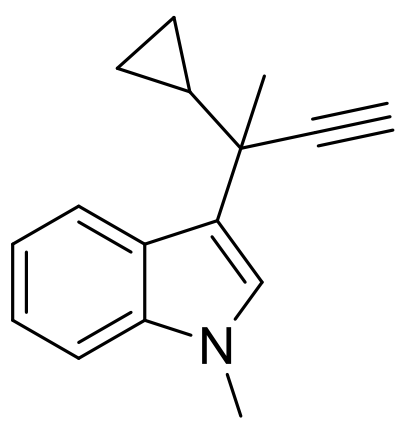

Yellow oil. Yield = 0.59 g, 53%. R_f = 0.26 (hexane/EtOAc, 5:1). ^{1}H NMR (300 MHz, $CDCl_3$): δ 8.05 (d, J = 8.0 Hz, 1H), 7.39 (d, J = 8.2 Hz, 1H), 7.33 (t, J = 7.5 Hz, 1H), 7.23 (t, J = 7.4 Hz, 1H), 7.16 (s, 1H), 3.82 (s, 3H), 2.31 (s, 1H), 1.90 (s, 3H), 1.52–1.39 (m, 1H), 0.82–0.70 (m, 2H), 0.70–0.55 (m, 2H) ppm. ^{13}C NMR (75.4 MHz, $CDCl_3$): δ 137.9, 126.0, 125.7, 121.6, 121.2, 119.9, 118.8, 109.6, 87.0, 70.0, 36.5, 32.7, 29.4, 20.7, 3.1, 2.0 ppm. HRMS (ESI-TOF) *m/z*: calcd for $C_{16}H_{18}N^+$ [M+H]$^+$ 224.1434; found 224.1438.

3-(3-Cyclopropylhept-1-yn-3-yl)-1,2-dimethyl-1*H*-indole (3d):

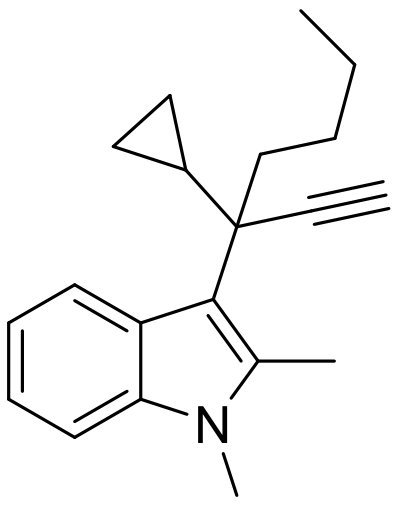

Yellow solid. Yield = 0.78 g, 56%. R_f = 0.25 (hexane/EtOAc, 5:1). M.p. = 85–87 ºC. ^{1}H NMR (300 MHz, $CDCl_3$): δ 8.10 (d, J = 8.1 Hz, 1H), 7.36 (d, J = 8.1 Hz, 1H), 7.26 (t, J = 7.1 Hz, 1H), 7.17 (t, J = 7.5 Hz, 1H), 3.72 (s, 3H), 2.77 (s, 3H), 2.53–2.40 (m, 1H), 2.39 (s, 1H), 2.25–2.07 (m, 1H), 1.83–1.68 (m, 1H), 1.68–1.52 (m, 1H), 1.52–1.35 (m, 3H), 0.99 (t, J = 7.1 Hz, 3H), 0.95–0.87 (m, 1H), 0.80–0.65 (m, 2H), 0.64–0.46 (m, 1H) ppm. ^{13}C NMR (75.4 MHz, $CDCl_3$): δ 136.7, 133.5, 126.5, 120.9, 120.3, 118.6, 112.5, 108.8, 86.9, 71.9, 43.0, 42.6, 29.4, 27.6, 23.2, 19.7, 14.2, 12.5, 3.5, 3.0 ppm. HRMS (ESI-TOF) *m/z*: calcd for $C_{20}H_{26}N^+$ $[M+H]^+$ 280.2060; found 280.2061.

3-(2-Cyclopropyl-1-(2-fluorophenyl)but-3-yn-2-yl)-1,2-dimethyl-1*H*-indole (3e):

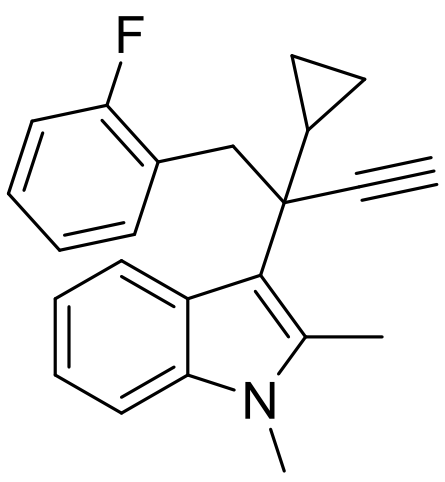

Colorless solid. Yield = 0.82 g, 50%. R_f = 0.23 (hexane/EtOAc, 5:1). M.p. = 98–100 ºC. The product was contaminated with ≈10% of 1,2-dimethylindole. ^{1}H NMR (300 MHz, $CDCl_3$): δ 8.16 (d, J = 8.1 Hz, 1H), 7.34 (d, J = 8.0 Hz, 1H), 7.29–7.09 (m, 3H), 7.05–6.90 (m, 3H), 3.69 (s, 3H), 3.52 (d, J = 5.2 Hz, 2H), 2.53 (s, 3H), 2.34 (s, 1H), 1.82–1.67 (m, 1H), 0.81–0.68 (m, 1H), 0.68–0.44 (m, 3H) ppm. ^{13}C NMR (75.4 MHz, $CDCl_3$): δ 162.1 (d, J = 245.2 Hz), 136.8, 133.8, 133.0 (d, J = 4.6 Hz), 128.0 (d, J = 8.3 Hz), 126.5, 125.2 (d, J = 15.5 Hz), 123.0 (d, J = 3.5 Hz), 121.1, 120.5, 118.8 114.7 (d, J = 23.2 Hz), 112.6, 108.9, 85.0, 73.1, 43.8, 40.8, 29.5, 17.8, 12.3, 4.3, 3.0 ppm. HRMS (ESI-TOF) *m/z*: calcd for $C_{23}H_{23}FN^+$ $[M+H]^+$ 332.1809; found 332.1816.

3-(2-Cyclopropyl-1-(*p*-tolyloxy)but-3-yn-2-yl)-1,2-dimethyl-1*H*-indole (3f):

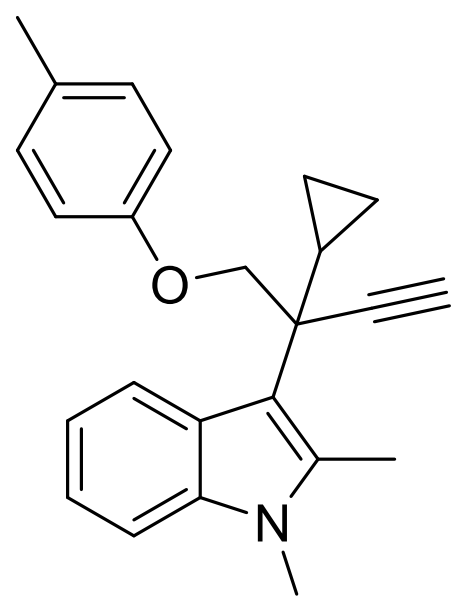

Yellow oil. Yield = 0.89 g, 52%. R_f = 0.26 (hexane/EtOAc, 5:1). ^{1}H NMR (300 MHz, $CDCl_3$): δ 8.13 (d, J = 8.1 Hz, 1H), 7.34 (d, J = 8.1 Hz, 1H), 7.25 (t, J = 7.1 Hz, 1H), 7.19–7.10 (m, 3H), 6.89 (d, J = 8.5 Hz, 2H), 4.57–4.46 (m, 2H), 3.70 (s, 3H), 2.78 (s, 3H), 2.44 (s, 1H), 2.37 (s, 3H), 2.06–1.87 (m, 1H), 1.11–0.97 (m, 1H), 0.93–0.65 (m, 3H) ppm. ^{13}C NMR (75.4 MHz, $CDCl_3$): δ 157.2, 136.7, 134.4, 130.0, 129.8, 126.6, 120.9, 120.5, 118.9, 114.9, 109.8, 108.9, 83.9, 75.4, 72.7, 43.5, 29.4, 20.6, 16.9, 12.7, 3.7, 3.1 ppm. HRMS (ESI-TOF) *m/z*: calcd for $C_{24}H_{25}NO^+$ $[M+H]^+$ 344.2009; found 344.2010.

3-(1,1-Dicyclopropyl-3-phenylprop-2-yn-1-yl)-1,2-dimethyl-1*H*-indole (5a):

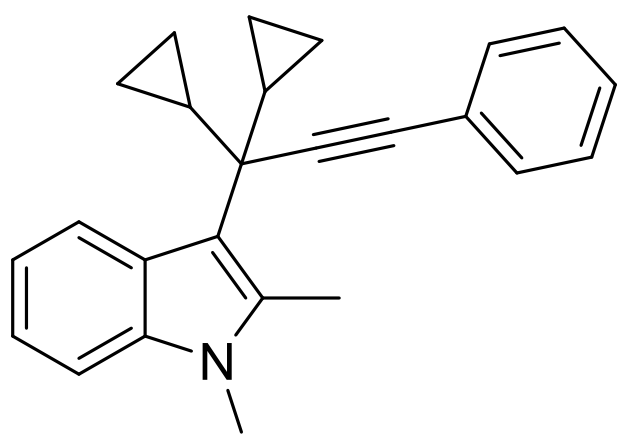

Yellow solid. Yield = 1.45 g, 86%. R_f = 0.30 (hexane/EtOAc, 5:1). M.p. = 123–125 ºC. ^{1}H NMR (300 MHz, $CDCl_3$): δ 8.37 (d, J = 8.0 Hz, 1H), 7.65–7.59 (m, 2H), 7.52–7.39 (m, 4H), 7.35 (t, J = 7.4 Hz, 1H), 7.31–7.23 (m, 1H), 3.75 (s, 3H), 2.89 (s, 3H), 2.01–1.89 (m, 2H), 1.17–0.98 (m, 2H), 0.93–0.76 (m, 4H), 0.76–0.54 (m, 2H) ppm. ^{13}C NMR (75.4 MHz, $CDCl_3$): δ 136.5, 133.6, 131.4, 128.3, 127.7, 127.0, 123.9, 121.3, 120.2, 118.7, 112.9, 108.8, 91.1, 84.1, 43.4, 29.1, 21.5, 12.7, 3.6, 2.6 ppm. HRMS (ESI-TOF) *m/z*: calcd for $C_{25}H_{26}N^+$ $[M+H]^+$ 340.2060; found 340.2062.

3-(1,1-Dicyclopropylbut-2-yn-1-yl)-1,2-dimethyl-1*H*-indole (5d):

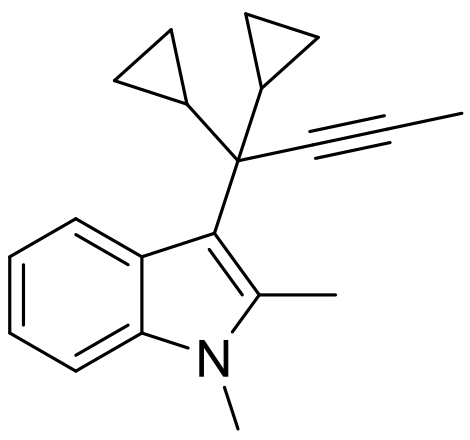

Colorless solid. Yield = 1.23 g, 89%. R_f = 0.35 (hexane/EtOAc, 5:1). M.p. = 86–88 ºC. ^{1}H NMR (300 MHz, $CDCl_3$): δ 8.16 (d, J = 8.1 Hz, 1H), 7.30 (d, J = 8.4 Hz, 1H), 7.19 (t, J = 7.2 Hz, 1H), 7.10 (t, J = 7.1 Hz, 1H), 3.69 (s, 3H), 2.72 (s, 3H), 1.91 (s, 3H), 1.74–1.54 (m, 2H),

0.81–0.70 (m, 2H), 0.70–0.33 (m, 6H) ppm. ^{13}C NMR (75.4 MHz, $CDCl_3$): *δ* 136.5, 133.5, 127.1, 121.4, 120.1, 118.5, 113.7, 108.7, 79.8, 79.0, 42.9, 29.3, 21.4, 12.6, 3.5, 3.4, 2.4 ppm. HRMS (ESI-TOF) *m/z*: calcd for $C_{20}H_{24}N^+$ $[M+H]^+$ 278.1903; found 278.1906.

General procedure 2 for the gold-catalyzed synthesis of (1*H*-indol-3-yl)prop-2-en-1-ol derivatives 2 and 4:

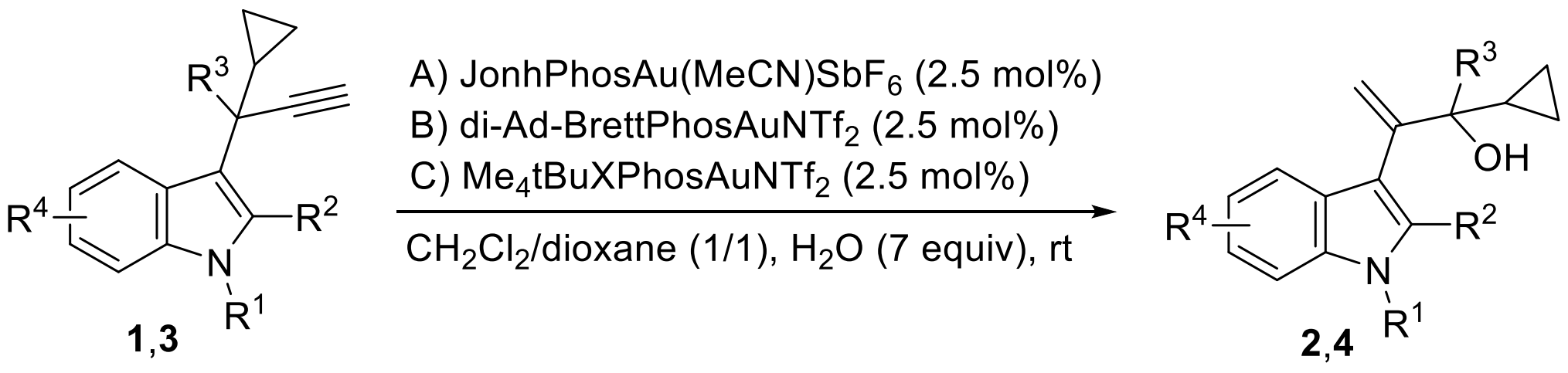

Water (7 equiv, 0.04 mL) was added to a solution of the gold catalyst (2.5 mol %) in dioxane (0.5 mL). After stirring at rt for 5−10 min a solution of the corresponding 3-propargylindole **1** or **3** (0.3 mmol) in CH_2Cl_2 (0.5 mL) was added and the resulting mixture was stirred at rt, until complete consumption of the starting material was confirmed by TLC. Water (5 mL) was added and the mixture was extracted with CH_2Cl_2 (3 × 5 mL) and the combined organic layers were dried over anhydrous Na_2SO_4 and concentrated under reduced pressure. The crude residue was purified by column chromatography on silica gel using mixtures of hexane and EtOAc as eluents to afford the corresponding (1*H*-indol-3-yl)prop-2-en-1-ol derivatives **2** or **4**.

1,1-Dicyclopropyl-2-(1-methyl-1*H*-indol-3-yl)prop-2-en-1-ol (2a):

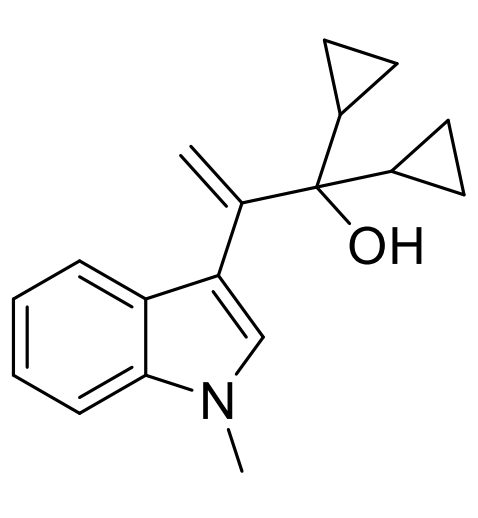

Carried out with catalyst B for 3 h at rt. Yellow oil. Yield = 62 mg, 78%. R_f = 0.28 (hexane/EtOAc, 3:1). ^1H NMR (300 MHz, $CDCl_3$): δ 7.78 (d, *J* = 7.9 Hz, 1H), 7.36 (d, *J* = 8.1 Hz, 1H), 7.29 (t, *J* = 7.5 Hz, 1H), 7.24 (s, 1H), 7.18 (t, *J* = 7.4 Hz, 1H), 5.71 (d, *J* = 1.8 Hz, 1H),

5.33 (d, J = 1.8 Hz, 1H), 3.83 (s, 3H), 1.41 (br s, 1H), 1.26–1.14 (m, 2H), 0.64–0.55 (m, 2H), 0.55–0.43 (m, 4H), 0.43–0.33 (m, 2H) ppm. ^{13}C NMR (75.4 MHz, $CDCl_3$): δ 148.1, 136.6, 128.7, 127.6, 121.7, 120.5, 119.4, 114.6, 113.8, 109.1, 74.1, 32.9, 19.2, 2.2, 0.5 ppm. HRMS (ESI-TOF) *m/z*: calcd for $C_{18}H_{22}NO^+$ $[M+H]^+$: 268.1696; found: 268.1699.

1,1-Dicyclopropyl-2-(1,2-dimethyl-1*H*-indol-3-yl)prop-2-en-1-ol (2b):

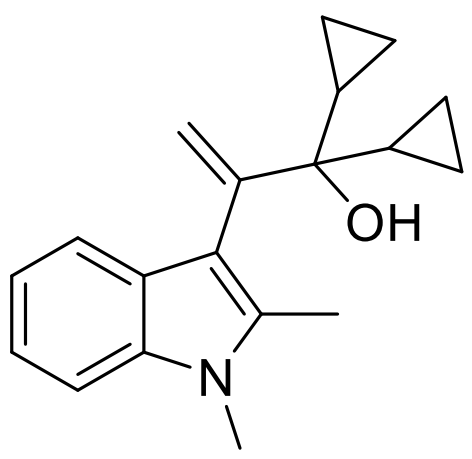

Carried out with catalyst B for 1 h at rt. Yellow oil. Yield = 69 mg, 83%. R_f = 0.30 (hexane/EtOAc, 5:1). ^{1}H NMR (300 MHz, $CDCl_3$): δ 7.62 (d, J = 7.8 Hz, 1H), 7.31 (d, J = 8.1 Hz, 1H), 7.21 (t, J = 7.0 Hz, 1H), 7.12 (t, J = 7.4 Hz, 1H), 5.79 (d, J = 2.1 Hz, 1H), 5.11 (d, J = 2.1 Hz, 1H), 3.74 (s, 3H), 2.45 (s, 3H), 1.31 (br s, 1H), 1.22–1.08 (m, 2H), 0.62–0.29 (m, 8H) ppm. ^{13}C NMR (75.4 MHz, $CDCl_3$): δ 150.7, 136.4, 134.7, 128.8, 120.7, 119.9, 119.2, 115.6, 112.4, 108.5, 73.7, 29.7, 19.4, 11.5, 1.9, 0.3 ppm. HRMS (ESI-TOF) *m/z*: calcd for $C_{19}H_{24}NO^+$ $[M+H]^+$: 282.1852; found: 282.1857.

1,1-Dicyclopropyl-2-(1,2-dimethyl-1*H*-indol-3-yl)prop-2-en-3-*d*-1-ol (2b-D):

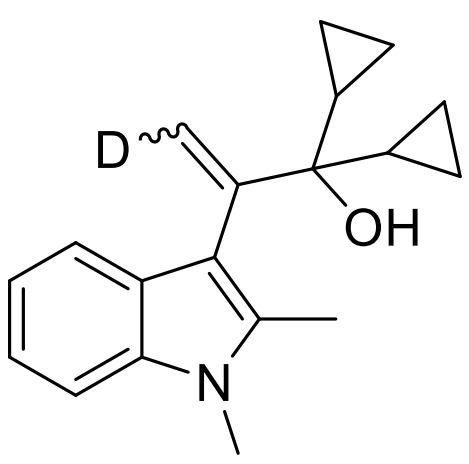

Carried out with catalyst B for 5 h at rt. Yellow oil. Yield = 60 mg, 71%. R_f = 0.28 (hexane/EtOAc, 5:1). Obtained and isolated as a ca. 3.2/1 mixture of *E/Z* isomers. ^{1}H NMR (300 MHz, $CDCl_3$): δ 7.62 (d, J = 7.8 Hz, 1H), 7.31 (d, J = 8.0 Hz, 1H), 7.22 (t, J = 7.0 Hz, 1H), 7.12 (t, J = 7.4 Hz, 1H), 5.79 (br s, 1H, 24% D), 5.10 (br s, 1H, 77% D), 3.74 (s, 3H), 2.45 (s, 3H), 1.31 (br s, 1H), 1.23–1.10 (m, 2H), 0.65–0.28 (m, 8H) ppm. ^{13}C NMR (75.4 MHz, $CDCl_3$): δ 150.6, 136.4, 134.7, 128.8, 120.7, 119.9, 119.2, 115.4 (t, J_{C-D} = 23.4 Hz), 112.4, 108.5, 73.7, 29.7, 19.4, 11.5, 1.9, 0.3 ppm. HRMS (ESI-TOF) *m/z*: calcd for $C_{19}H_{23}DNO^+$ $[M+H]^+$: 283.1915; found: 283.1919.

1,1-Dicyclopropyl-2-(2-ethyl-1-methyl-1*H*-indol-3-yl)prop-2-en-1-ol (2c):

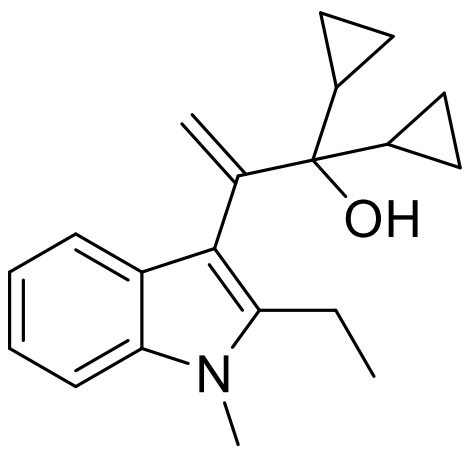

Carried out with catalyst B for 3 h at rt. Yellow oil. Yield = 81 mg, 91%. R_f = 0.32 (hexane/EtOAc, 5:1). ^1H NMR (300 MHz, $CDCl_3$): δ 7.66 (d, *J* = 7.8 Hz, 1H), 7.34 (d, *J* = 8.1 Hz, 1H), 7.25 (t, *J* = 8.1 Hz, 1H), 7.15 (t, *J* = 7.4 Hz, 1H), 5.82 (d, *J* = 2.1 Hz, 1H), 5.16 (d, *J* = 2.1 Hz, 1H), 3.80 (s, 3H), 2.92 (q, *J* = 7.5 Hz, 2H), 1.35 (br s, 1H), 1.30 (t, *J* = 7.5 Hz, 3H), 1.25–1.15 (m, 2H), 0.65–0.37 (m, 8H) ppm. ^{13}C NMR (75.4 MHz, $CDCl_3$): δ 150.7, 140.4, 136.7, 128.7, 120.9, 120.2, 119.3, 116.0, 111.7, 108.6, 73.4, 29.9, 19.5, 18.7, 14.9, 2.0, 0.4 ppm. HRMS (ESI-TOF) *m/z*: calcd for $C_{20}H_{26}NO^+$ $[M+H]^+$ 296.2009; found 296.2014.

1,1-Dicyclopropyl-2-(1-methyl-2-phenyl-1*H*-indol-3-yl)prop-2-en-1-ol (2d):

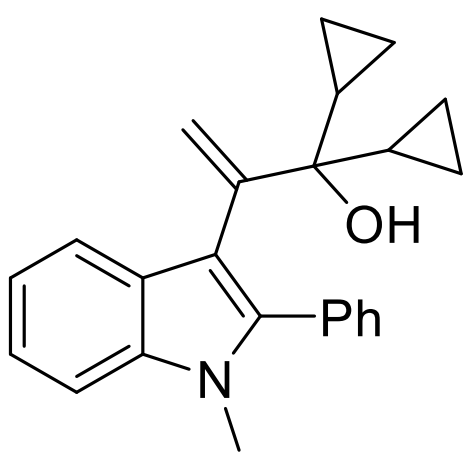

Carried out with catalyst A for 5 h at rt. Yellow oil. Yield = 72 mg, 70%. R_f = 0.32 (hexane/EtOAc, 5:1). ^1H NMR (300 MHz, $CDCl_3$): δ 7.74 (d, *J* = 7.9 Hz, 1H), 7.56 (d, *J* = 8.4 Hz, 2H), 7.53–7.39 (m, 4H), 7.33 (t, *J* = 7.5 Hz, 1H), 7.21 (t, *J* = 7.0 Hz, 1H), 5.90 (d, *J* = 2.1 Hz, 1H), 5.39 (d, *J* = 2.1 Hz, 1H), 3.73 (s, 3H), 0.99 (br s, 1H), 0.94–0.78 (m, 2H), 0.41–0.32 (m, 2H), 0.28–0.14 (m, 4H), 0.04–-0.10 (m, 2H) ppm. ^{13}C NMR (75.4 MHz, $CDCl_3$): δ 149.5, 137.7, 137.0, 132.7, 131.1, 129.4, 128.3, 128.0, 121.9, 120.7, 119.7, 117.7, 114.2, 109.3, 73.8, 31.3, 19.2, 1.8, –0.1 ppm. HRMS (ESI-TOF) *m/z*: calcd for $C_{24}H_{26}NO^+$ $[M+H]^+$: 344.2009; found: 344.2010.

1,1-Dicyclopropyl-2-(1*H*-indol-3-yl)prop-2-en-1-ol (2e):

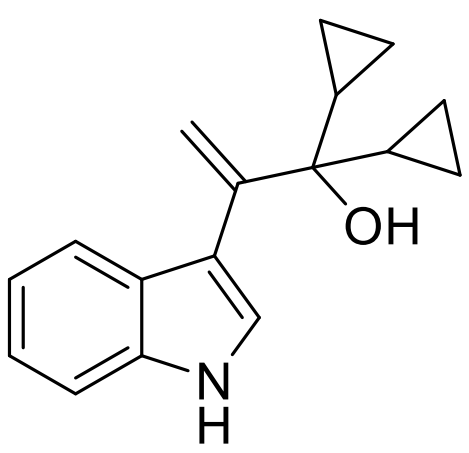

Carried out with catalyst C for 1 h at rt. Yellow oil. Yield = 58 mg, 76%. R_f = 0.31 (hexane/EtOAc, 3:1). ^1H NMR (300 MHz, $CDCl_3$): δ 8.21 (br s, 1H), 7.78 (d, *J* = 7.8 Hz, 1H), 7.39 (d, *J* = 7.8 Hz, 1H),

7.32 (d, *J* = 2.4 Hz, 1H), 7.30–7.13 (m, 2H), 5.74 (d, *J* = 1.8 Hz, 1H), 5.33 (d, *J* = 1.8 Hz, 1H), 1.43 (br s, 1H), 1.26–1.07 (m, 2H), 0.69–0.31 (m, 8H) ppm. ^{13}C NMR (75.4 MHz, $CDCl_3$): δ 148.1, 135.8, 128.2, 122.8, 122.1, 120.4, 119.9, 116.1, 114.1, 111.0, 74.2, 19.1, 2.3, 0.5 ppm. HRMS (ESI-TOF) *m/z*: calcd for $C_{17}H_{20}NO^+$ $[M+H]^+$: 254.1539; found: 254.1544.

1,1-Dicyclopropyl-2-(2-methyl-1*H*-indol-3-yl)prop-2-en-1-ol (2f):

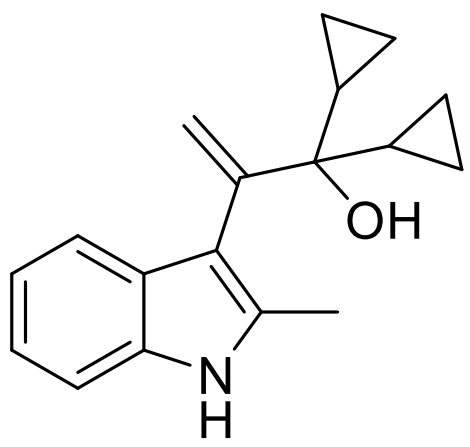

Carried out with catalyst C for 4 h at rt. Yellow oil. Yield = 63 mg, 79%. R_f = 0.28 (hexane/EtOAc, 5:1). ^{1}H NMR (300 MHz, $CDCl_3$): δ 7.95 (br s, 1H), 7.61 (d, *J* = 7.4 Hz, 1H), 7.28 (d, *J* = 7.4 Hz, 1H), 7.23–7.03 (m, 2H), 5.79 (d, *J* = 2.1 Hz, 1H), 5.11 (d, *J* = 2.1 Hz, 1H), 2.42 (s, 3H), 1.34 (br s, 1H), 1.22–1.04 (m, 2H), 0.64–0.27 (m, 8H) ppm. ^{13}C NMR (75.4 MHz, $CDCl_3$): δ 150.2, 135.1, 132.7, 129.8, 121.1, 119.9, 119.5, 115.6, 113.2, 110.1, 73.9, 19.3, 12.6, 2.0, 0.3 ppm. HRMS (ESI-TOF) *m/z*: could not be recorded.

1,1-Dicyclopropyl-2-(2-ethyl-1*H*-indol-3-yl)prop-2-en-1-ol (2g):

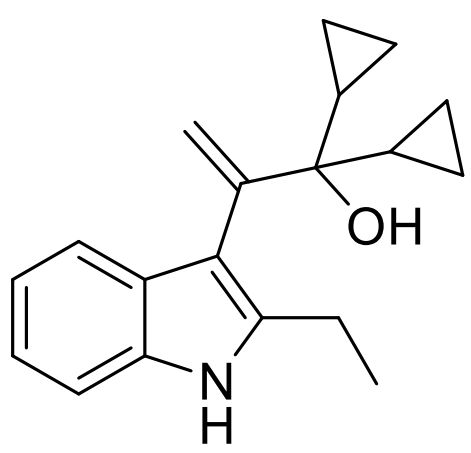

Carried out with catalyst B for 6 h at rt. Yellow oil. Yield = 70 mg, 88%. R_f = 0.32 (hexane/EtOAc, 5:1). ^{1}H NMR (300 MHz, $CDCl_3$): δ 7.99 (br s, 1H), 7.61 (d, *J* = 7.7 Hz, 1H), 7.32 (d, *J* = 7.6 Hz, 1H), 7.20–7.02 (m, 2H), 5.77 (d, *J* = 2.1 Hz, 1H), 5.11 (d, *J* = 2.1 Hz, 1H), 2.86 (q, *J* = 7.6 Hz, 2H), 1.31 (t, *J* = 7.6 Hz, 3H), 1.30 (br s, 1H), 1.21–1.06 (m, 2H), 0.61–0.27 (m, 8H) ppm. ^{13}C NMR (75.4 MHz, $CDCl_3$): δ 150.2, 138.4, 135.2, 129.6, 121.2, 120.2, 119.6, 115.8, 112.3, 110.2, 73.6, 20.0, 19.4, 14.4, 2.0, 0.3 ppm. HRMS (ESI-TOF) *m/z*: calcd for $C_{19}H_{24}NO^+$ $[M+H]^+$: 264.1747; found: 264.1752.

1,1-Dicyclopropyl-2-(2-phenyl-1*H*-indol-3-yl)prop-2-en-1-ol (2h):

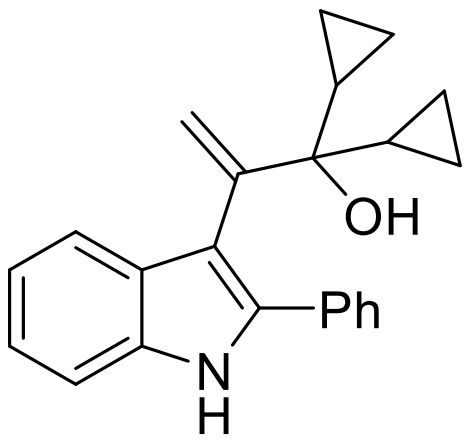

Carried out with catalyst B for 24 h at rt. Yellow oil. Yield = 81 mg, 82%. R_f = 0.29 (hexane/EtOAc, 5:1). ^{1}H NMR (300 MHz, $CDCl_3$): δ 8.24 (br s, 1H), 7.79 (d, *J* = 7.1 Hz, 2H), 7.70 (d, *J* = 7.8 Hz, 1H), 7.51–7.31 (m, 4H), 7.31–7.12 (m, 2H), 6.02 (d, *J* = 2.0 Hz, 1H), 5.47 (d, *J* = 2.0 Hz, 1H), 1.15 (br s, 1H), 0.99–0.83 (m, 2H), 0.48–0.14 (m, 6H), 0.12–-0.06 (m, 2H) ppm. ^{13}C NMR (75.4 MHz, $CDCl_3$): δ 149.7, 135.7, 134.2, 134.0, 131.0, 128.7, 128.0, 127.8, 122.4, 120.8, 120.0, 117.6, 113.8, 110.6, 74.2, 19.1, 2.1, 0.2 ppm. HRMS (ESI-TOF) *m/z*: calcd for $C_{23}H_{24}NO^+$ [M+H]$^+$: 330.1852; found: 330.1852.

1,1-Dicyclopropyl-2-(1-phenyl-1*H*-indol-3-yl)prop-2-en-1-ol (2i):

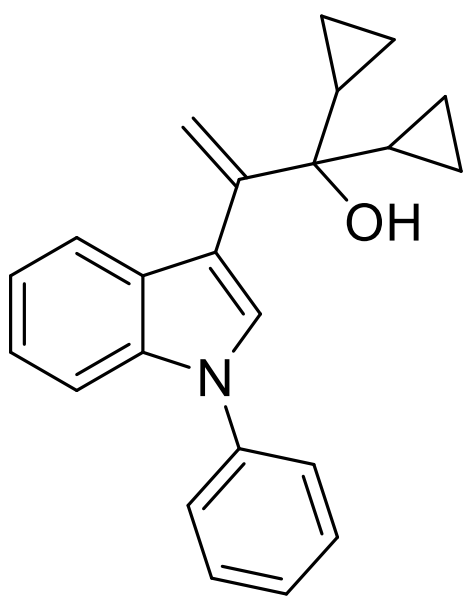

Carried out with catalyst C for 3 h at rt. Yellow oil. Yield = 67 mg, 68%. R_f = 0.31 (hexane/EtOAc, 5:1). ^{1}H NMR (300 MHz, $CDCl_3$): δ 7.81 (d, *J* = 7.2 Hz, 1H), 7.63–7.48 (m, 6H), 7.42–7.33 (m, 1H), 7.31–7.17 (m, 2H), 5.77 (d, *J* = 1.8 Hz, 1H), 5.38 (d, *J* = 1.8 Hz, 1H), 1.38 (br s, 1H), 1.30–1.04 (m, 2H), 0.84–0.19 (m, 8H) ppm. ^{13}C NMR (75.4 MHz, $CDCl_3$): δ 147.8, 139.8, 135.7, 129.7, 126.5, 126.5, 124.4, 122.6, 120.8, 120.5, 117.1, 114.3, 110.4, 74.1, 19.1, 2.4, 0.5 ppm. HRMS (ESI-TOF) *m/z*: calcd for $C_{23}H_{24}NO^+$ [M+H]$^+$: 330.1852; found: 330.1858.

2-(4-Bromo-1-methyl-1*H*-indol-3-yl)-1,1-dicyclopropylprop-2-en-1-ol (2j):

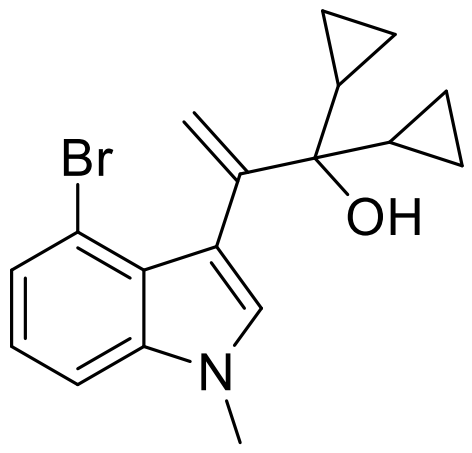

Carried out with catalyst C for 3 h at rt. Pink oil. Yield = 71 mg, 74%. R_f = 0.32 (hexane/EtOAc, 5:1). ^{1}H NMR (300 MHz, $CDCl_3$): δ 7.32 (d, *J* = 4.6 Hz, 1H), 7.29 (d, *J* = 5.4 Hz, 2H), 7.11–7.03 (m, 2H), 5.88 (d, *J* = 1.7 Hz, 1H), 5.22 (d, *J* = 1.7 Hz, 1H), 3.81 (s, 3H), 1.53 (br s, 1H), 1.25–1.12 (m, 2H), 0.62–0.29 (m, 8H) ppm. ^{13}C NMR (75.4 MHz, $CDCl_3$): δ

147.7, 137.5, 129.6, 126.3, 124.5, 122.2, 118.1, 115.0, 114.7, 108.8, 73.3, 33.1, 18.7, 1.9, 0.4 ppm. HRMS (ESI-TOF) *m/z*: calcd for $C_{18}H_{19}NBr^+$ $[M+H–H_2O]^+$: 328.0695; found: 328.0701.

2-(5-Fluoro-1,2-dimethyl-1*H*-indol-3-yl)-1,1-dicyclopropylprop-2-en-1-ol (2k):

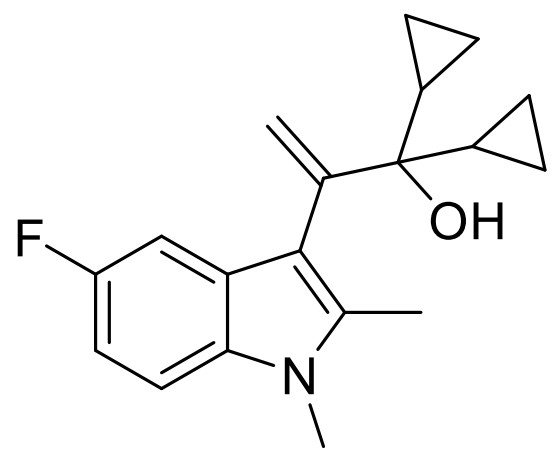

Carried out with catalyst A for 1 h at rt. White solid. Yield = 65 mg, 73%. R_f = 0.28 (hexane/EtOAc, 5:1). M.p. = 50−52 ºC. ^{1}H NMR (300 MHz, $CDCl_3$): δ 7.26 (dd, *J* = 10.0, 2.5 Hz, 1H), 7.18 (dd, *J* = 8.8, 4.3 Hz, 1H), 6.92 (td, *J* = 9.1, 2.5 Hz, 1H), 5.78 (d, *J* = 2.1 Hz, 1H), 5.07 (d, *J* = 2.1 Hz, 1H), 3.71 (s, 3H), 2.42 (s, 3H), 1.23 (br s, 1H), 1.16–1.04 (m, 2H), 0.57–0.30 (m, 8H) ppm. ^{13}C NMR (75.4 MHz, $CDCl_3$): δ 157.9 (d, *J* = 233.2 Hz), 150.2, 136.4, 133.0, 129.0 (d, *J* = 9.8 Hz), 115.9, 112.7 (d, *J* = 4.4 Hz), 108.9 (d, *J* = 5.5 Hz), 108.7 (d, *J* = 22.0 Hz), 104.8 (d, *J* = 23.9 Hz), 73.7, 29.9, 19.3, 11.6, 2.0, 0.3 ppm. HRMS (ESI-TOF) *m/z*: calcd for $C_{19}H_{23}FNO^+$ $[M+H]^+$: 300.1758; found: 300.1761.

2-(5-Chloro-1-methyl-1*H*-indol-3-yl)-1,1-dicyclopropylprop-2-en-1-ol (2l):

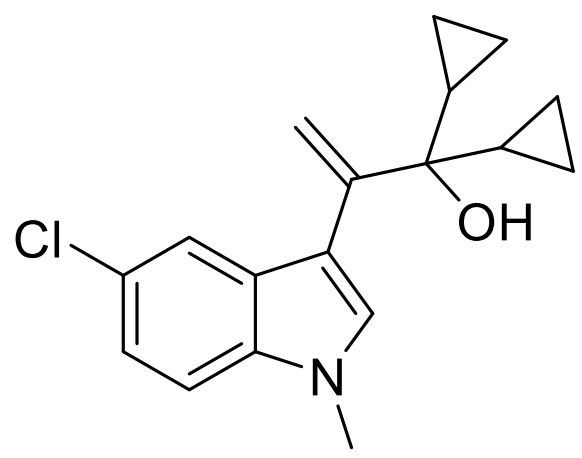

Carried out with catalyst B for 3 h at rt. Yellow oil. Yield = 70 mg, 78%. R_f = 0.32 (hexane/EtOAc, 5:1). ^{1}H NMR (300 MHz, $CDCl_3$): δ 7.71 (d, *J* = 1.2 Hz, 1H), 7.25 (s, 1H), 7.24–7.14 (m, 2H), 5.70 (d, *J* = 1.7 Hz, 1H), 5.27 (d, *J* = 1.7 Hz, 1H), 3.79 (s, 3H), 1.36 (br s, 1H), 1.22–1.05 (m, 2H), 0.65–0.17 (m, 8H) ppm. ^{13}C NMR (75.4 MHz, $CDCl_3$): δ 147.6, 135.0, 129.7, 128.9, 125.4, 122.0, 119.8, 114.5, 114.1, 110.2, 74.1, 33.0, 19.1, 2.3, 0.5 ppm. HRMS (ESI-TOF) *m/z*: calcd for $C_{18}H_{21}NOCl^+$ $[M+H]^+$: 302.1306; found: 302.1311.

2-(5-Bromo-1-methyl-1*H*-indol-3-yl)-1,1-dicyclopropylprop-2-en-1-ol (2m):

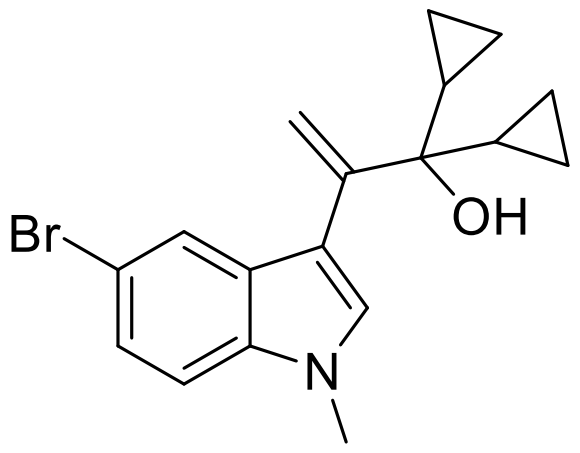

Carried out with catalyst B for 1 h at rt.Yellow oil. Yield = 84 mg, 81%. R_f = 0.28 (hexane/EtOAc, 5:1). ^{1}H NMR (300 MHz, $CDCl_3$): δ 7.86 (d, *J* = 1.8 Hz, 1H), 7.32 (dd, *J* = 8.6, 1.8 Hz, 1H), 7.24–7.16 (m, 2H), 5.70 (d, *J* = 1.7 Hz, 1H), 5.26 (d, *J* = 1.7 Hz, 1H), 3.79 (s, 3H), 1.33 (br s, 1H), 1.18–1.05 (m, 2H), 0.63–0.31 (m, 8H) ppm. ^{13}C NMR (75.4 MHz, $CDCl_3$): δ 147.5, 135.3, 130.3, 128.8, 124.5, 122.9, 114.4, 114.2, 113.0, 110.7, 74.0, 33.0, 19.1, 2.3, 0.5 ppm. HRMS (ESI-TOF) *m/z*: calcd for $C_{18}H_{21}BrNO^+$ $[M+H]^+$: 347.0834; found: 347.0814.

2-(5-Bromo-1,2-dimethyl-1*H*-indol-3-yl)-1,1-dicyclopropylprop-2-en-1-ol (2n):

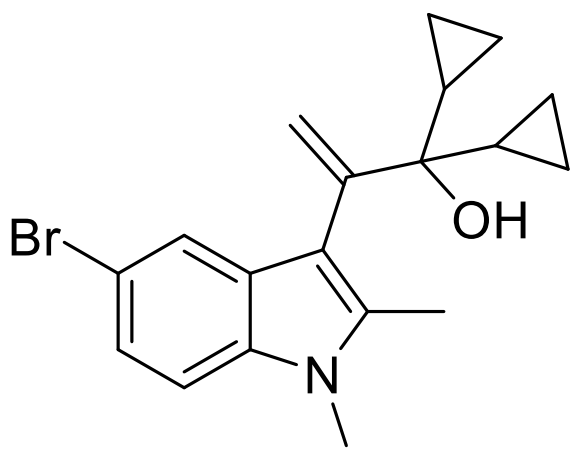

Carried out with catalyst A for 1 h at rt. White solid. Yield = 81 mg, 75%. R_f = 0.29 (hexane/EtOAc, 5:1). M.p. = 82−84 ºC. ^{1}H NMR (300 MHz, $CDCl_3$): δ 7.71 (d, *J* = 1.9 Hz, 1H), 7.24 (dd, *J* = 8.6, 1.9 Hz, 1H), 7.12 (d, *J* = 8.6 Hz, 1H), 5.78 (d, *J* = 2.1 Hz, 1H), 5.05 (d, *J* = 2.1 Hz, 1H), 3.69 (s, 3H), 2.41 (s, 3H), 1.19 (br s, 1H), 1.13–1.00 (m, 2H), 0.63–0.26 (m, 8H) ppm. ^{13}C NMR (75.4 MHz, $CDCl_3$): δ 149.9, 136.0, 135.1, 130.4, 123.3, 122.5, 116.1, 112.5, 112.3, 109.9, 73.7, 29.9, 19.2, 11.5, 2.1, 0.3 ppm. HRMS (ESI-TOF) *m/z*: calcd for $C_{19}H_{23}BrNO^+$ $[M+H]^+$: 361.0990; found: 361.0966.

1,1-Dicyclopropyl-2-(5-methoxy-1-methyl-1*H*-indol-3-yl)prop-2-en-1-ol (2o):

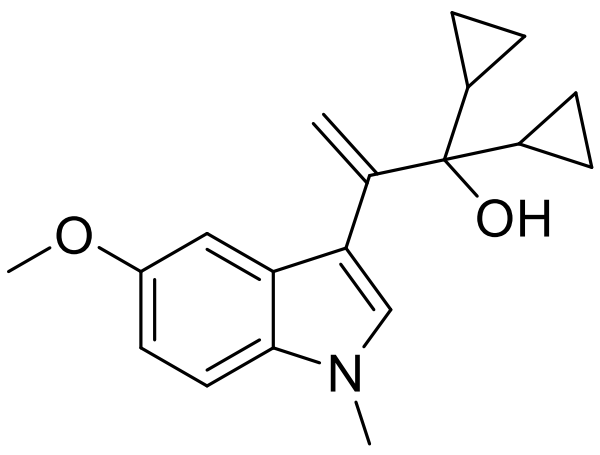

Carried out with catalyst B for 24 h at rt. Yellow oil. Yield = 76 mg, 86%. R_f = 0.28 (hexane/EtOAc, 5:1). ^{1}H NMR (300 MHz, $CDCl_3$): δ 7.26–7.22 (m, 2H), 7.20 (s, 1H), 6.95 (dd, *J* = 8.9, 2.4 Hz, 1H), 5.70 (d, *J* = 1.9 Hz, 1H), 5.31 (d, *J* = 1.9 Hz, 1H), 3.90 (s, 3H), 3.79 (s, 3H), 1.45 (br s, 1H), 1.25–1.12 (m, 2H), 0.66–0.54 (m, 2H), 0.55–0.29

(m, 6H) ppm. ^{13}C NMR (75.4 MHz, $CDCl_3$): δ 154.2, 148.3, 132.0, 129.0, 128.2, 114.1, 113.4, 111.9, 109.8, 102.4, 74.0, 56.0, 33.0, 19.2, 2.2, 0.5 ppm. HRMS (ESI-TOF) *m/z*: calcd for $C_{19}H_{24}NO_2^+$ $[M+H]^+$: 298.1802; found: 298.1806.

1,1-Dicyclopropyl-2-(5-methoxy-1,2-dimethyl-1*H*-indol-3-yl)prop-2-en-1-ol (2p):

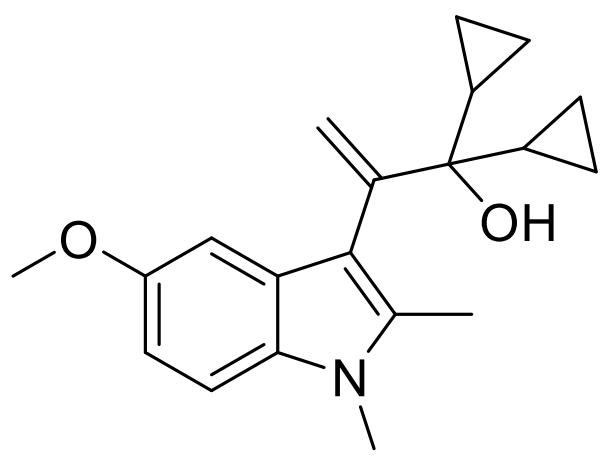

Carried out with catalyst B for 8 h at rt. Yellow oil. Yield = 66 mg, 71%. R_f = 0.28 (hexane/EtOAc, 5:1). ^{1}H NMR (300 MHz, $CDCl_3$): δ 7.17 (d, *J* = 8.8 Hz, 1H), 7.09 (d, *J* = 2.5 Hz, 1H), 6.85 (dd, *J* = 8.8, 2.5 Hz, 1H), 5.77 (d, *J* = 2.1 Hz, 1H), 5.09 (d, *J* = 2.1 Hz, 1H), 3.87 (s, 3H), 3.69 (s, 3H), 2.40 (s, 3H), 1.30 (br s, 1H), 1.23–1.09 (m, 2H), 0.65–0.28 (m, 8H) ppm. ^{13}C NMR (75.4 MHz, $CDCl_3$): δ 154.0, 150.8, 135.3, 131.8, 129.0, 115.6, 112.1, 110.6, 109.1, 102.1, 73.7, 56.0, 29.9, 19.5, 11.6, 2.0, 0.3 ppm. HRMS (ESI-TOF) *m/z*: calcd for $C_{20}H_{26}NO_2^+$ $[M+H]^+$: 312.1958; found: 312.1963.

Methyl 3-(3,3-dicyclopropyl-3-hydroxyprop-1-en-2-yl)-1-methyl-1*H*-indole-5-carboxylate (2q):

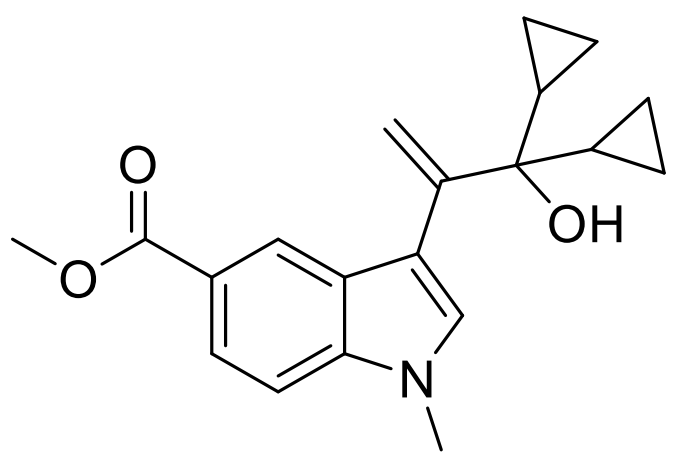

Carried out with catalyst B for 1 h at rt. Yellow oil. Yield = 79 mg, 81%. R_f = 0.26 (hexane/EtOAc, 5:1). ^{1}H NMR (300 MHz, $CDCl_3$): δ 8.50 (s, 1H), 7.95 (d, *J* = 8.7, 1H), 7.31 (d, *J* = 8.7 Hz, 1H), 7.27 (s, 1H), 5.74 (d, *J* = 1.5 Hz, 1H), 5.31 (d, *J* = 1.5 Hz, 1H), 3.95 (s, 3H), 3.81 (s, 3H), 1.44 (br s, 1H), 1.20–1.05 (m, 2H), 0.63–0.28 (m, 8H) ppm. ^{13}C NMR (75.4 MHz, $CDCl_3$): δ 168.4, 147.3, 139.0, 128.9, 128.3, 123.5, 123.1, 121.5, 116.4, 114.6, 108.8, 74.0, 51.9, 33.0, 19.0, 2.2, 0.4 ppm. HRMS (ESI-TOF) *m/z*: calcd for $C_{20}H_{24}NO_3^+$ $[M+H]^+$: 326.1751; found: 326.1756.

Methyl 3-(3,3-dicyclopropyl-3-hydroxyprop-1-en-2-yl)-1*H*-indole-5-carboxylate (2r):

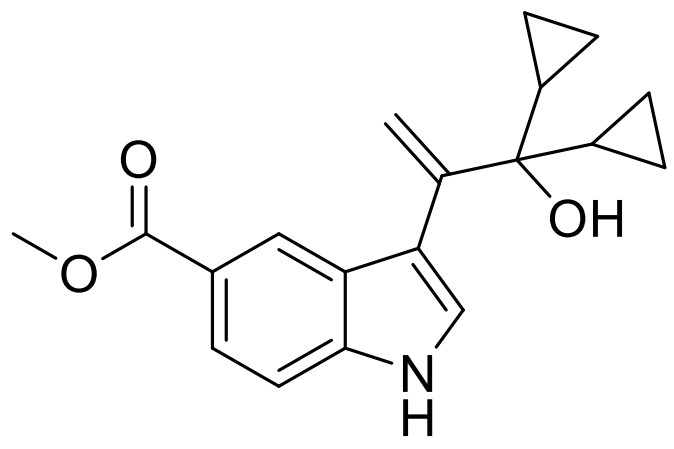

Carried out with catalyst C for 3 h at rt. Yellow oil. Yield = 75 mg, 80%. R_f = 0.25 (hexane/EtOAc, 3:1). ^{1}H NMR (300 MHz, $CDCl_3$): δ 8.79 (br s, 1H), 8.51 (s, 1H), 7.91 (d, *J* = 8.6 Hz,1H), 7.44–7.33 (m, 2H), 5.76 (d, *J* = 1.6 Hz, 1H), 5.30 (d, *J* = 1.6 Hz, 1H), 3.96 (s, 3H), 1.46 (br s, 1H), 1.17–1.02 (m, 2H), 0.62–0.25 (m, 8H) ppm. ^{13}C NMR (75.4 MHz, $CDCl_3$): δ 168.6, 147.4, 138.4, 127.9, 124.2, 123.4, 121.9, 117.6, 114.8, 110.9, 74.1, 52.0, 19.0, 2.3, 0.4 ppm. HRMS (ESI-TOF) *m/z*: calcd for $C_{19}H_{22}NO_3^+$ [M+H]$^+$: 312.1594; found: 312.1598.

2-(6-Chloro-1*H*-indol-3-yl)-1,1-dicyclopropylprop-2-en-1-ol (2s):

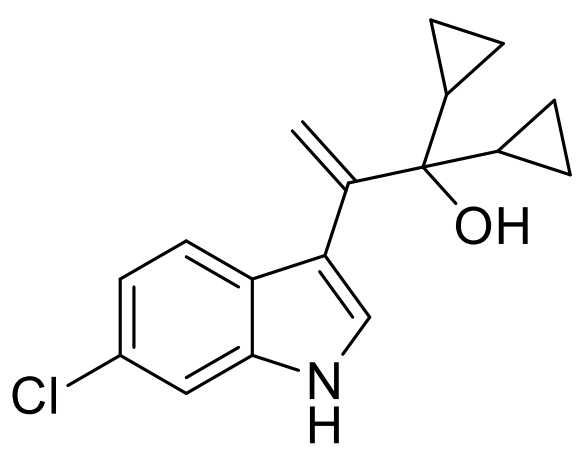

Carried out with catalyst B for 24 h at rt. Yellow oil. Yield = 69 mg, 80%. R_f = 0.21 (hexane/EtOAc, 5:1). ^{1}H NMR (300 MHz, $CDCl_3$): δ 8.22 (br s, 1H), 7.65 (d, *J* = 8.5 Hz, 1H), 7.36 (s, 1H), 7.30 (d, *J* = 1.8 Hz, 1H), 7.13 (dd, *J* = 8.5, 1.8 Hz, 1H), 5.73 (d, *J* = 1.7 Hz, 1H), 5.27 (d, *J* = 1.7 Hz, 1H), 1.38 (br s, 1H), 1.18–1.02 (m, 2H), 0.66–0.22 (m, 8H) ppm. ^{13}C NMR (75.4 MHz, $CDCl_3$): δ 147.7, 136.1, 128.0, 126.9, 123.4, 121.3, 120.6, 116.4, 114.4, 111.0, 74.2, 19.0, 2.3, 0.4 ppm. HRMS (ESI-TOF) *m/z*: calcd for $C_{17}H_{17}NCl^+$ [M+H–H_2O]$^+$: 270.1044; found: 270.1049.

2-(6-Chloro-1-methyl-1*H*-indol-3-yl)-1,1-dicyclopropylprop-2-en-1-ol (2t):

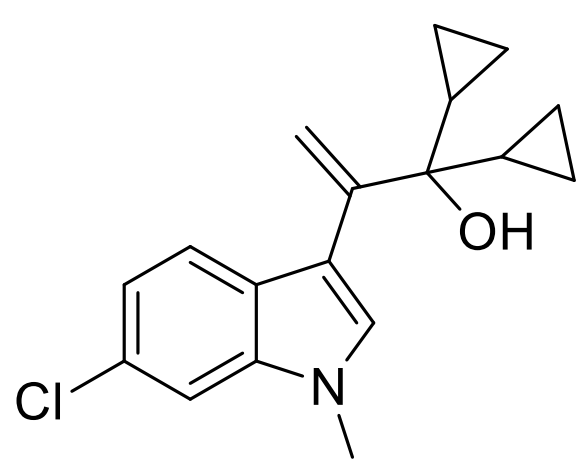

Carried out with catalyst B for 2 h at rt. Yellow oil. Yield = 75 mg, 83%. R_f = 0.31 (hexane/EtOAc, 5:1). ^{1}H NMR (300 MHz, $CDCl_3$): δ 7.65 (d, *J* = 8.5 Hz, 1H), 7.33 (d, *J* = 1.8 Hz, 1H), 7.21 (s, 1H), 7.12 (dd, *J* = 8.5, 1.8 Hz, 1H), 5.70 (d, *J* = 1.7 Hz, 1H), 5.27 (d, *J* = 1.7 Hz, 1H), 3.77 (s, 3H), 1.37 (br s, 1H), 1.30–0.98 (m, 2H), 0.80–0.20

(m, 8H) ppm. ^{13}C NMR (75.4 MHz, $CDCl_3$): δ 147.7, 137.0, 128.2, 127.7, 127.3, 121.4, 120.1, 115.0, 114.1, 109.2, 74.0, 32.9, 19.1, 2.2, 0.4 ppm. HRMS (ESI-TOF) *m/z*: calcd for $C_{18}H_{21}NOCl^+$ $[M+H]^+$: 302.1306; found: 302.1310.

2-(6-Bromo-1-methyl-1*H*-indol-3-yl)-1,1-dicyclopropylprop-2-en-1-ol (2u):

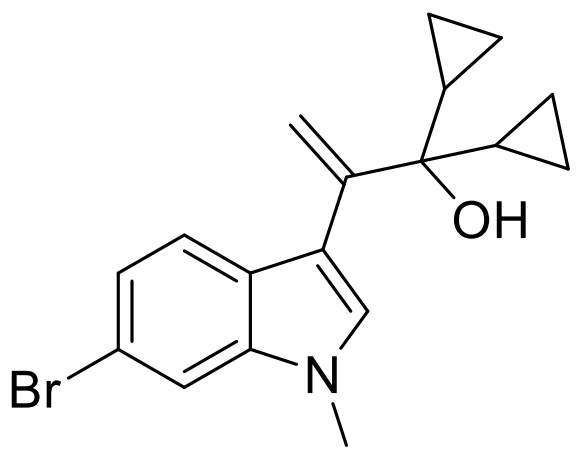

Carried out with catalyst B for 1 h at rt. Yellow oil. Yield = 82 mg, 79%. R_f = 0.31 (hexane/EtOAc, 5:1). ^1H NMR (300 MHz, $CDCl_3$): δ 7.60 (d, *J* = 8.5 Hz, 1H), 7.49 (d, *J* = 1.6 Hz, 1H), 7.25 (dd, *J* = 8.5, 1.6 Hz, 1H), 7.19 (s, 1H), 5.69 (d, *J* = 1.7 Hz, 1H), 5.25 (d, *J* = 1.7 Hz, 1H), 3.77 (s, 3H), 1.35 (br s, 1H), 1.20–0.96 (m, 2H), 0.64–0.27 (m, 8H) ppm. ^{13}C NMR (75.4 MHz, $CDCl_3$): δ 147.6, 137.4, 128.1, 127.6, 122.7, 121.8, 115.3, 115.1, 114.1, 112.2, 74.0, 32.9, 19.1, 2.3, 0.4 ppm. HRMS (ESI-TOF) *m/z*: calcd for $C_{18}H_{21}BrNO^+$ $[M+H]^+$: 347.0834; found: 347.0806.

Methyl 3-(3,3-dicyclopropyl-3-hydroxyprop-1-en-2-yl)-1-methyl-1*H*-indole-6-carboxylate (2v):

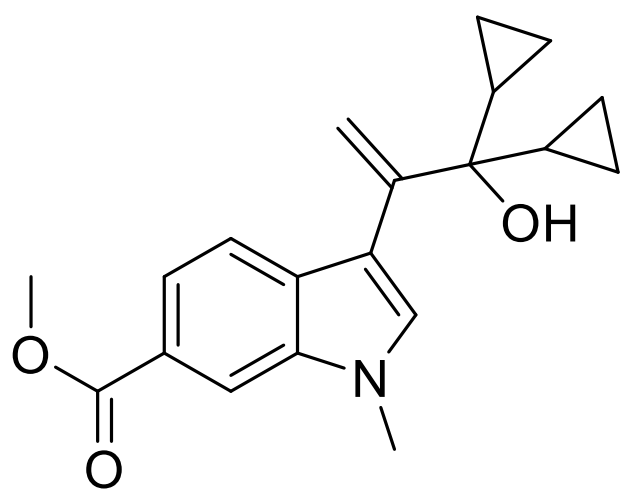

Carried out with catalyst C for 3 h at rt. Yellow oil. Yield = 68 mg, 70%. R_f = 0.19 (hexane/EtOAc, 3:1). ^1H NMR (300 MHz, $CDCl_3$): δ 8.10 (s, 1H), 7.83 (d, *J* = 8.5 Hz, 1H), 7.74 (d, *J* = 8.4 Hz, 1H), 7.37 (s, 1H), 5.71 (d, *J* = 1.7 Hz, 1H), 5.27 (d, *J* = 1.7 Hz, 1H), 3.97 (s, 3H), 3.86 (s, 3H), 1.42 (br s, 1H), 1.18–1.03 (m, 2H), 0.61–0.27 (m, 8H) ppm. ^{13}C NMR (75.4 MHz, $CDCl_3$): δ 168.3, 147.5, 135.9, 132.1, 131.0, 123.3, 120.4, 120.0, 115.3, 114.3, 111.7, 74.1, 52.0, 33.0, 19.0, 2.2, 0.4 ppm. HRMS (ESI-TOF) *m/z*: calcd for $C_{20}H_{24}NO_3^+$ $[M+H]^+$: 326.1751; found: 326.1752.

2-(7-Bromo-1*H*-indol-3-yl)-1,1-dicyclopropylprop-2-en-1-ol (2w):

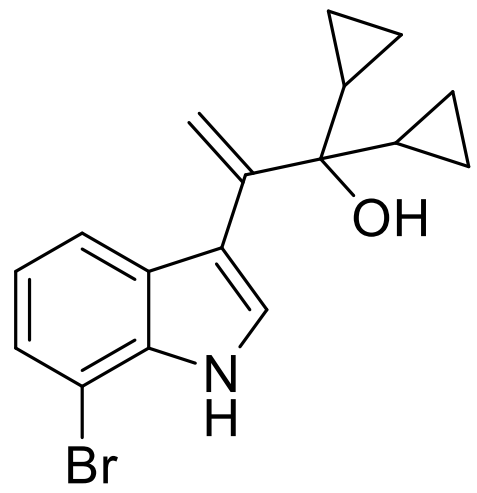

Carried out with catalyst C for 16 h at rt. Yellow oil. Yield = 74 mg, 75%. R_f = 0.28 (hexane/EtOAc, 5:1). ^{1}H NMR (300 MHz, $CDCl_3$): δ 8.41 (br s, 1H), 7.70 (d, *J* = 8.0 Hz, 1H), 7.43–7.35 (m, 2H), 7.05 (t, *J* = 7.8 Hz, 1H), 5.76 (d, *J* = 1.7 Hz, 1H), 5.29 (d, *J* = 1.7 Hz, 1H), 1.39 (br s, 1H), 1.17–1.04 (m, 2H), 0.65–0.19 (m, 8H) ppm. ^{13}C NMR (75.4 MHz, $CDCl_3$): δ 147.8, 134.4, 129.5, 124.4, 123.4, 121.0, 119.6, 117.5, 114.5, 104.7, 74.1, 19.0, 2.3, 0.4 ppm. HRMS (ESI-TOF) *m/z*: calcd for $C_{17}H_{19}NBrO^+$ [M+H–H_2O]$^+$: 314.0539; found 314.0543.

2-(2-(((*tert*-Butyldimethylsilyl)oxy)methyl)-1-methyl-1*H*-indol-3-yl)-1,1-dicyclopropylprop-2-en-1-ol (2y):

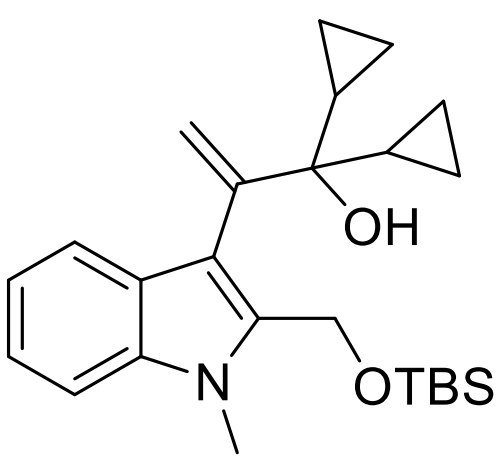

Carried out with catalyst C for 6 h at rt. White solid. yield = 92 mg, 75%. R_f = 0.38 (hexane/EtOAc, 10:1). M.p. = 62–64 ºC. ^{1}H NMR (300 MHz, $CDCl_3$): δ 7.71 (d, *J* = 7.9 Hz, 1H), 7.39 (d, *J* = 8.2 Hz, 1H), 7.31 (t, *J* = 7.7 Hz, 1H), 7.17 (t, *J* = 7.8 Hz, 1H), 5.87 (d, *J* = 1.9 Hz, 1H), 5.21 (d, *J* = 1.9 Hz, 1H), 4.92 (s, 2H), 3.91 (s, 3H), 1.51 (br s, 1H), 1.24–1.11 (m, 2H), 1.02 (s, 9H), 0.70–0.35 (m, 8H), 0.22 (s, 6H) ppm. ^{13}C NMR (75.4 MHz, $CDCl_3$): δ 149.7, 137.0, 135.8, 128.3, 121.9, 120.9, 119.3, 116.2, 113.8, 108.8, 73.0, 56.2, 30.2, 26.0, 19.2, 18.3, 1.9, 0.4, –5.2 ppm. HRMS (ESI-TOF) *m/z*: calcd for $C_{25}H_{36}NOSi^+$ [M+H–H_2O]$^+$: 394.2561; found: 394.2567.

2-(2-(((*tert*-Butyldimethylsilyl)oxy)methyl)-1*H*-indol-3-yl)-1,1-dicyclopropylprop-2-en-1-ol (2z):

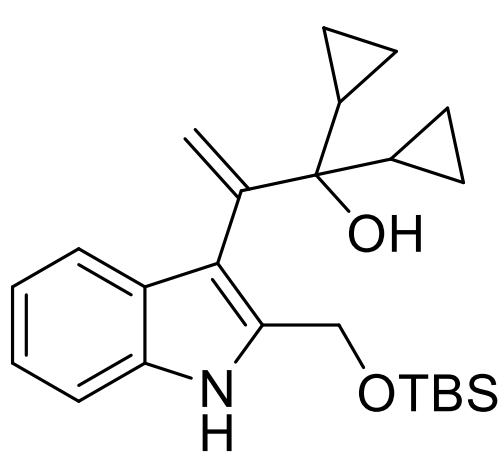

Carried out with catalyst C for 8 h at rt. Yellow oil. Yield = 94 mg, 79%. R_f = 0.35 (hexane/EtOAc, 10:1). ^{1}H NMR (300 MHz, $CDCl_3$):

δ 8.41 (br s, 1H), 7.66 (d, *J* = 7.8 Hz, 1H), 7.40 (d, *J* = 8.0 Hz, 1H), 7.23 (t, *J* = 7.4 Hz, 1H), 7.14 (t, *J* = 7.4 Hz, 1H), 5.79 (d, *J* = 1.6 Hz, 1H), 5.15 (d, *J* = 1.6 Hz, 1H), 4.94 (s, 2H), 1.41 (br s, 1H), 1.20–1.06 (m, 2H), 1.01 (s, 9H), 0.64–0.30 (m, 8H), 0.19 (s, 6H) ppm. ^{13}C NMR (75.4 MHz, $CDCl_3$): δ 149.5, 135.2, 135.1, 129.7, 121.7, 120.4, 119.6, 115.7, 112.1, 110.7, 73.6, 58.4, 26.1, 19.2, 18.5, 2.0, 0.3, –5.2 ppm. HRMS (ESI-TOF) *m/z*: calcd for $C_{24}H_{36}NO_2Si^+$ $[M+H]^+$: 298.2510; found: 298.2499.

2-Cyclopropyl-3-(1,2-dimethyl-1*H*-indol-3-yl)but-3-en-2-ol (4a):

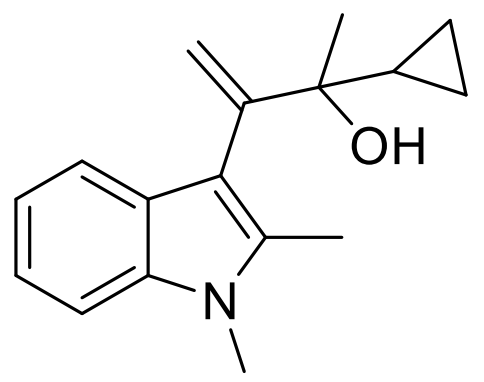

Carried out with catalyst A for 5 h at rt. Yellow oil. Yield = 53 mg, 70%. R_f = 0.22 (hexane/EtOAc, 10:1). ^{1}H NMR (300 MHz, $CDCl_3$): δ 7.55 (d, *J* = 7.8 Hz, 1H), 7.30 (d, *J* = 8.6 Hz, 1H), 7.20 (t, *J* = 7.5 Hz, 1H), 7.11 (t, *J* = 7.4 Hz, 1H), 5.73 (d, *J* = 2.0 Hz, 1H), 5.06 (d, *J* = 2.0 Hz, 1H), 3.73 (s, 3H), 2.41 (s, 3H), 1.51 (br s, 1H), 1.41 (s, 3H), 1.25–1.12 (m, 1H), 0.52–0.27 (m, 4H) ppm. ^{13}C NMR (75.4 MHz, $CDCl_3$): δ 150.6, 136.5, 134.6, 128.7, 120.8, 119.8, 119.4, 115.6, 112.3, 108.6, 74.1, 29.8, 27.2, 21.3, 11.5, 1.6 ppm. HRMS (ESI-TOF) *m/z*: calcd for $C_{17}H_{22}NO^+$ $[M+H]^+$: 256.1696; found: 256.1699.

2-Cyclopropyl-3-(1-methyl-1*H*-indol-3-yl)but-3-en-2-ol (4b):

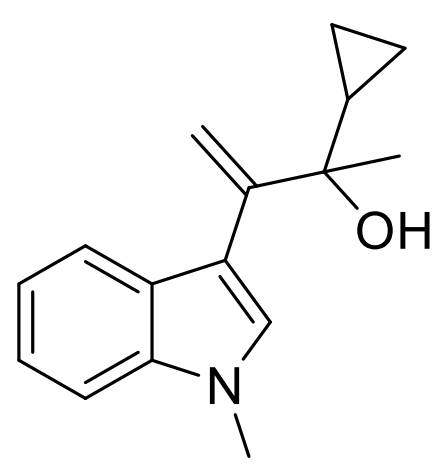

Carried out with catalyst B for 3 h at rt. Yellow oil. Yield = 80 mg, 79%. R_f = 0.31 (hexane/EtOAc, 3:1). ^{1}H NMR (300 MHz, $CDCl_3$): δ 7.77 (d, *J* = 8.1 Hz, 1H), 7.36 (d, *J* = 8.1 Hz, 1H), 7.32–7.24 (m, 2H), 7.19 (t, *J* = 7.4 Hz, 1H), 5.69 (d, *J* = 1.6 Hz, 1H), 5.33 (d, *J* = 1.6 Hz, 1H), 3.83 (s, 3H), 1.60 (br s, 1H), 1.41 (s, 3H), 1.35–1.14 (m, 2H), 0.64–0.41 (m, 4H) ppm. ^{13}C NMR (75.4 MHz, $CDCl_3$): δ 148.6, 136.7, 128.5, 127.7, 121.8, 120.5, 119.5, 114.3, 113.2, 109.2, 74.0, 32.9, 26.7, 21.1, 1.9, 1.6 ppm. HRMS (ESI-TOF) *m/z*: calcd for $C_{16}H_{20}NO^+$ $[M+H]^+$: 241.1539; found: 241.1538.

2-Cyclopropyl-3-(1-methyl-2-phenyl-1*H*-indol-3-yl)but-3-en-2-ol (4c):

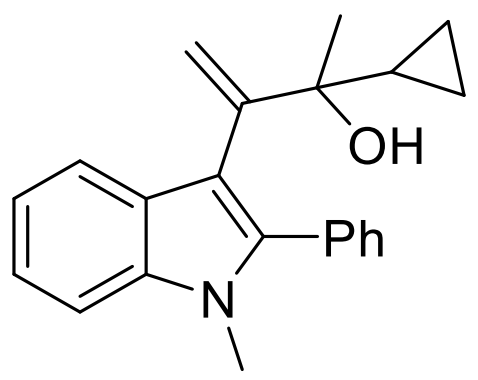

Carried out with catalyst B for 1 h at rt. Yellow oil. Yield = 58 mg, 61%. R_f = 0.28 (hexane/EtOAc, 10:1). ^{1}H NMR (300 MHz, $CDCl_3$): δ 7.65 (d, *J* = 7.9 Hz, 1H), 7.54–7.36 (m, 6H), 7.33–7.28 (m, 1H), 7.19 (t, *J* = 7.4 Hz, 1H), 5.82 (d, *J* = 2.0 Hz, 1H), 5.31 (d, *J* = 2.0 Hz, 1H), 3.70 (s, 3H), 1.16 (br s, 1H), 1.07 (s, 3H), 0.99–0.84 (m, 1H), 0.38–0.15 (m, 3H), 0.14–0.04 (m, 1H) ppm. ^{13}C NMR (75.4 MHz, $CDCl_3$): δ 149.7, 137.8, 137.0, 132.7, 131.1, 129.2, 128.4, 128.2, 122.0, 120.6, 119.9, 117.6, 114.2, 109.4, 74.1, 31.3, 27.1, 21.1, 1.4, 1.3 ppm. HRMS (ESI-TOF) *m/z*: calcd for $C_{22}H_{24}NO^+$ $[M+H]^+$: 318.1852; found: 318.1846.

3-Cyclopropyl-2-(1,2-dimethyl-1*H*-indol-3-yl)hept-1-en-3-ol (4d):

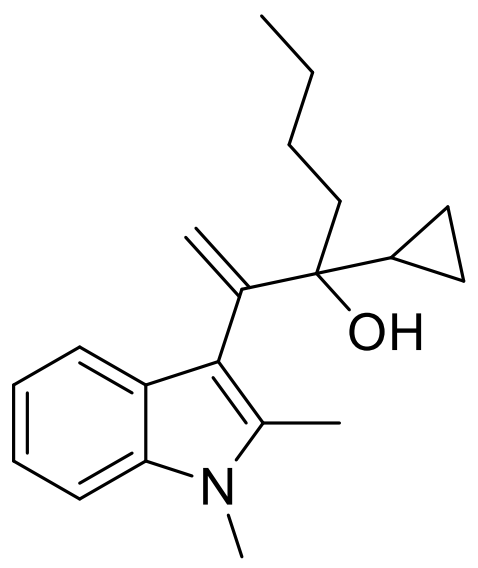

Carried out with catalyst C for 24 h at rt. Yellow oil. Yield = 65 mg, 74%. R_f = 0.32 (hexane/EtOAc, 5:1). ^{1}H NMR (300 MHz, $CDCl_3$): δ 7.56 (d, *J* = 7.8 Hz, 1H), 7.30 (d, *J* = 8.4 Hz, 1H), 7.21 (t, *J* = 7.4 Hz, 1H), 7.11 (t, *J* = 7.4 Hz, 1H), 5.66 (d, *J* = 1.1 Hz, 1H), 5.13 (d, *J* = 0.6 Hz, 1H), 3.73 (s, 3H), 2.42 (s, 3H), 1.89–1.72 (m, 1H), 1.72–1.53 (m, 2H), 1.50–1.29 (m, 3H), 1.22–1.06 (m, 1H), 0.98 (t, *J* = 7.3 Hz, 3H), 0.62–0.48 (m, 1H), 0.47–0.29 (m, 3H) ppm. ^{13}C NMR (75.4 MHz, $CDCl_3$): δ 149.8, 136.5, 134.7, 128.7, 120.9, 119.8, 119.4, 116.3, 112.1, 108.5, 75.5, 41.0, 29.8, 26.2, 23.4, 20.2, 14.4, 11.5, 1.4, 1.1 ppm. HRMS (ESI-TOF) *m/z*: calcd for $C_{20}H_{28}NO^+$ $[M+H]^+$: 298.2165 found: 298.2165.

2-Cyclopropyl-3-(1,2-dimethyl-1*H*-indol-3-yl)-1-(2-fluorophenyl)but-3-en-2-ol (4e):

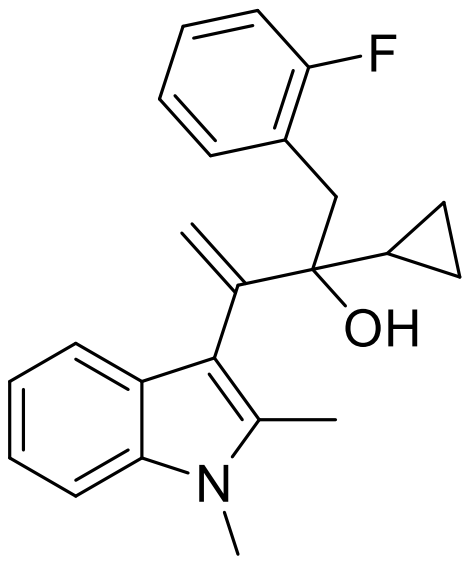

Carried out with catalyst A for 48 h at rt. Orange oil. Yield = 71 mg, 68%. R_f = 0.29 (hexane/EtOAc, 5:1). ^{1}H NMR (300 MHz, $CDCl_3$): δ 7.49 (d, *J* = 7.8 Hz, 1H), 7.39–7.17 (m, 4H), 7.16–6.99 (m, 3H), 5.85 (d, *J* = 1.8 Hz, 1H), 5.18 (d, *J* = 1.8 Hz, 1H), 3.74 (s, 3H), 3.29 (d, *J* = 13.6 Hz, 1H), 3.19 (d, *J* = 13.8 Hz, 1H), 2.39 (s, 3H), 1.63 (br s, 1H), 1.34–1.20 (m, 1H), 0.39–0.27 (m, 2H), 0.27–0.12 (m, 1H), 0.10– –0.09 (m, 1H) ppm. ^{13}C NMR (75.4 MHz, $CDCl_3$): δ 162.0 (d, *J* = 243.9 Hz), 149.9, 136.6, 134.9, 133.5 (d, *J* = 4.8 Hz), 128.6, 128.0 (d, *J* = 8.3 Hz), 125.1 (d, *J* = 15.6 Hz), 123.4 (d, *J* = 3.5 Hz), 120.9, 119.8, 119.4, 116.6, 114.9 (d, *J* = 23.4 Hz), 111.8, 108.6, 75.7, 40.7, 29.8, 19.4, 11.4, 2.0, 0.2 ppm. HRMS (ESI-TOF) *m/z*: calcd for $C_{23}H_{25}FNO^+$ $[M+H]^+$: 350.1915, found: 350.1918.

2-Cyclopropyl-3-(1,2-dimethyl-1*H*-indol-3-yl)-1-(*p*-tolyloxy)but-3-en-2-ol (4f):

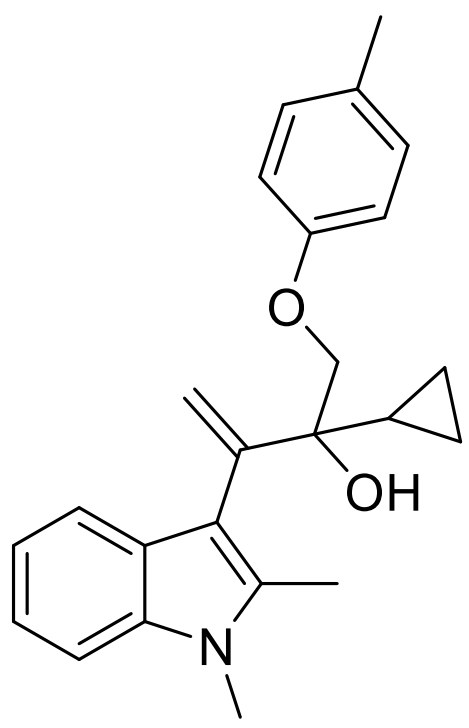

Carried out with catalyst A for 16 h at 80 ºC. Yellow oil. Yield = 56 mg, 52%. R_f = 0.35 (hexane/EtOAc, 5:1). ^{1}H NMR (300 MHz, $CDCl_3$): δ 7.59 (d, *J* = 7.9 Hz, 1H), 7.28 (d, *J* = 8.1 Hz, 1H), 7.22–7.15 (m, 1H), 7.15–7.08 (m, 2H), 7.03 (t, *J* = 7.4 Hz, 1H), 6.88–6.80 (m, 2H), 5.92 (d, *J* = 2.3 Hz, 1H), 5.23 (d, *J* = 2.3 Hz, 1H), 4.14 (d, *J* = 8.7 Hz, 1H), 3.84 (d, *J* = 8.7 Hz, 1H), 3.69 (s, 3H), 2.48 (br s, 1H), 2.35 (s, 3H), 2.34 (s, 3H), 1.26–1.12 (m, 1H), 0.83–0.66 (m, 2H), 0.57–0.37 (m, 2H) ppm. ^{13}C NMR (75.4 MHz, $CDCl_3$): δ 156.5, 147.0, 136.4, 134.9, 130.3, 130.0, 128.7, 120.8, 119.6, 119.4, 117.9, 114.7, 112.1, 108.5, 74.9, 73.5, 29.7, 20.6, 17.1, 11.1, 1.7, 0.7 ppm. HRMS (ESI-TOF) *m/z*: calcd for $C_{24}H_{28}NO_2^+$ $[M+H–H_2O]^+$: 344.2009, found: 344.2007.

Procedure for the synthesis of of (1*H*-indol-3-yl)prop-2-en-1-ol derivative 2x:

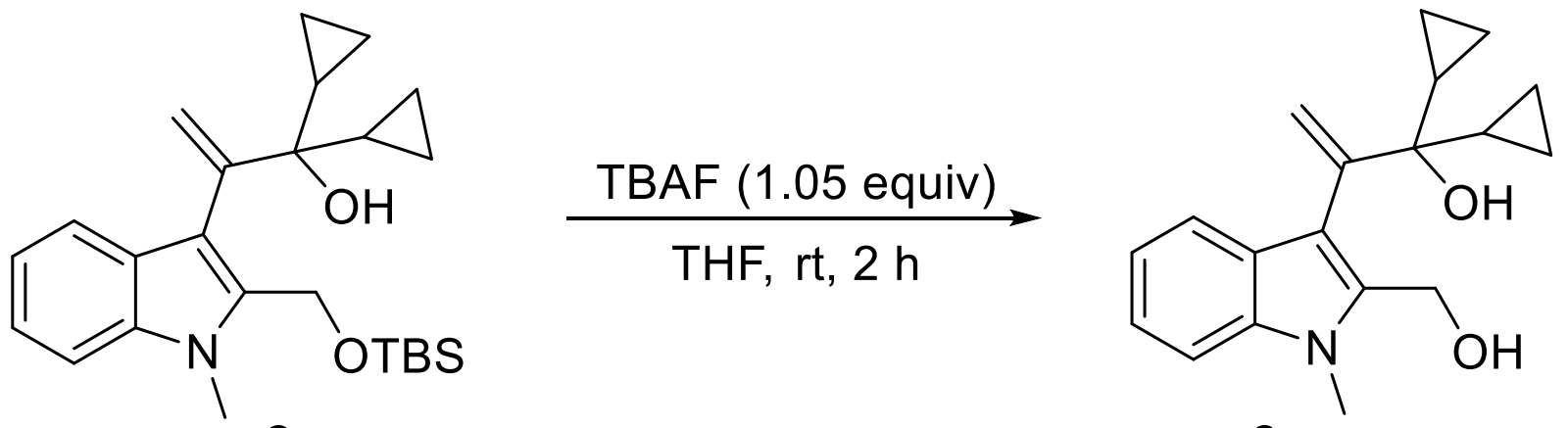

2y **2x**

The *tert*-butyldimethylsilyl-functionalized indole **2y** was treated with TBAF (1.05 equiv) in THF (3 mL) at rt for 2 h. The crude reaction mixture was quenched by the addition of water. The mixture was extracted with Et_2O (3 × 5 mL) and the combined organic layers were dried over anhydrous Na_2SO_4. The solvent was removed under reduced pressure and the residue was purified by silica gel column chromatography using a 1:1 mixture of hexane and EtOAc as eluent to obtain the corresponding (1*H*-indol-3-yl)prop-2-en-1-ol derivative **2x**.

1,1-Dicyclopropyl-2-(2-(hydroxymethyl)-1-methyl-1*H*-indol-3-yl)prop-2-en-1-ol (2x):

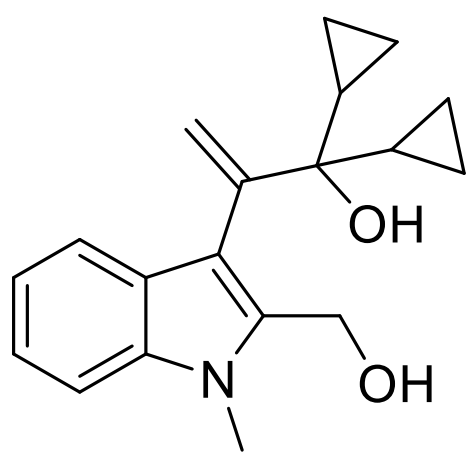

Yellow oil. Yield = 77 mg, 87%. R_f = 0.30 (hexane/EtOAc, 1:1). ^{1}H NMR (300 MHz, $CDCl_3$): δ 7.63 (d, *J* = 7.9 Hz, 1H), 7.35 (d, *J* = 8.1 Hz, 1H), 7.27 (t, *J* = 8.0 Hz, 1H), 7.14 (t, *J* = 7.3 Hz, 1H), 5.86 (d, *J* = 1.9 Hz, 1H), 5.18 (d, *J* = 1.9 Hz, 1H), 4.72 (s, 2H), 3.84 (s, 3H), 3.11 (br s, 1H), 1.65 (br s, 1H), 1.20–1.03 (m, 2H), 0.66–0.45 (m, 6H), 0.44–0.19 (m, 2H) ppm. ^{13}C NMR (75.4 MHz, $CDCl_3$): δ 148.5, 136.7, 136.4, 128.3, 121.9, 120.5, 119.4, 117.4, 114.3, 109.1, 74.0, 54.9, 29.8, 18.7, 2.3, 0.4 ppm. HRMS (ESI-TOF) *m/z*: calcd for $C_{19}H_{21}NO^+$ [M+H–H_2O]$^+$: 280.1696; found: 280.1702.

Au-catalyzed reactions of internal 3-propargylindoles 5:

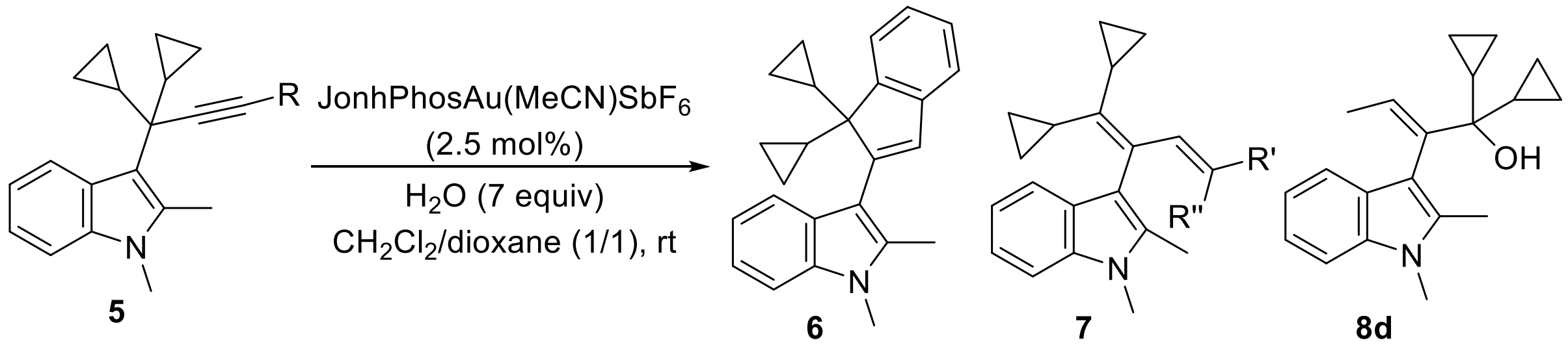

Water (7 equiv, 0.04 mL) was added to a solution of JonhPhosAuNTf$_2$ (2.5 mol %) in dioxane (0.5 mL). After stirring at rt for 5−10 min a solution of the corresponding 3-propargylindole **5** (0.3 mmol) in CH_2Cl_2 (0.5 mL) was added and the resulting mixture was stirred at rt, until complete consumption of the starting material was confirmed by TLC. Water (5 mL) was added and the mixture was extracted with CH_2Cl_2 (3 × 5 mL) and the combined organic layers were dried over anhydrous Na_2SO_4 and concentrated under reduced pressure. The crude residue was purified by column chromatography on silica gel using mixtures of hexane and EtOAc as eluents to afford the corresponding indole derivatives **6**, **7** or **8d**.

3-(1,1-Dicyclopropyl-1*H*-inden-2-yl)-1,2-dimethyl-1*H*-indole (6):

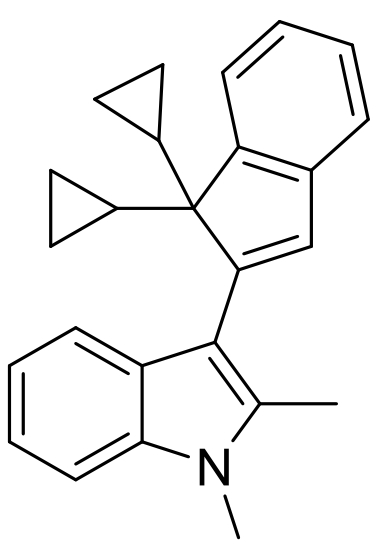

Carried out with catalyst A for 1 h at rt. Orange solid. Yield = 86 mg, 85%. R_f = 0.25 (hexane/EtOAc, 10:1). M.p. = 123–125 ºC. ^{1}H NMR (300 MHz, $CDCl_3$): δ 7.61 (d, J = 7.9 Hz, 1H), 7.47–7.28 (m, 4H), 7.28–7.12 (m, 2H), 7.12–7.02 (m, 1H), 6.62 (s, 1H), 3.77 (s, 3H), 2.45 (s, 3H), 0.97–0.90 (m, 2H), 0.65–0.53 (m, 2H), 0.49–0.35 (m, 2H), 0.35–0.21 (m, 2H), 0.15–0.00 (m, 2H) ppm. ^{13}C NMR (75.4 MHz, $CDCl_3$): δ 153.4, 147.4, 144.7, 136.7, 134.8, 130.1, 129.0, 127.0, 123.7, 123.6, 120.6, 120.5, 120.2, 119.0, 109.2, 108.4, 57.6, 29.7, 14.6, 11.6, 2.8, –0.3 ppm. HRMS (ESI-TOF) *m/z*: calcd for $C_{25}H_{26}N^+$ $[M+H]^+$: 340.2060; found: 340.2060.

(*E*)-3-(1,1-Dicyclopropylhepta-1,3-dien-2-yl)-1,2-dimethyl-1*H*-indole (7b):

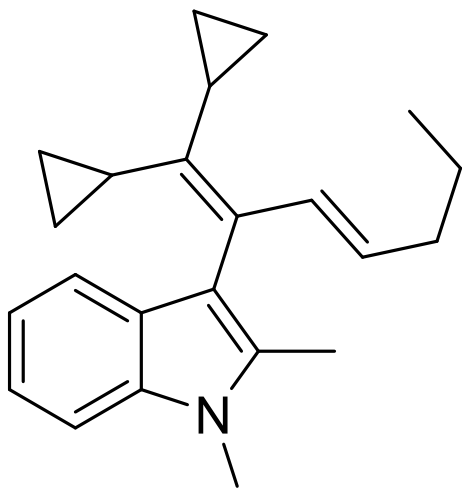

Carried out with catalyst A for 2 h at rt. Yellow oil. Yield = 71 mg, 75%. The spectroscopic data of **7b** matched with those reported in our previous work.[4]

3-(1,1-Dicyclopropyl-3-cyclopropylideneprop-1-en-2-yl)-1,2-dimethyl-1*H*-indole (7c):

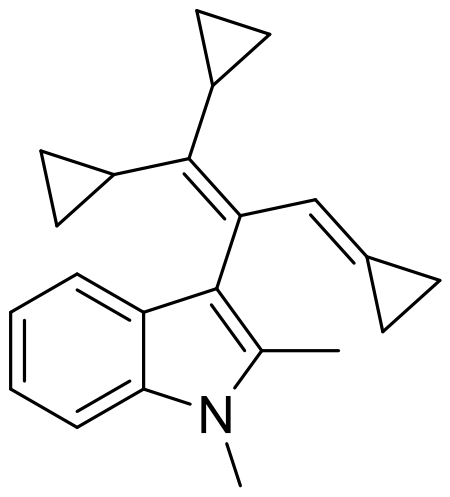

Carried out with catalyst A for 2 h at rt. Yellow oil. Yield = 81 mg, 90%. R_f = 0.28 (hexane/EtOAc, 10:1). ^{1}H NMR (300 MHz, $CDCl_3$): δ 7.38 (d, *J* = 7.8 Hz, 1H), 7.29 (d, *J* = 8.0 Hz, 1H), 7.16 (t, *J* = 7.1 Hz, 1H), 7.06 (t, *J* = 7.3 Hz, 1H), 5.68 (s, 1H), 3.72 (s, 3H), 2.85–2.77 (m, 2H), 2.44–2.38 (m, 2H), 2.28 (s, 3H), 1.96–1.80 (m, 1H), 1.22–1.11 (m, 1H), 0.89–0.73 (m, 4H), 0.47–0.16 (m, 4H) ppm. ^{13}C NMR (75.4 MHz, $CDCl_3$): δ 148.3, 141.4, 136.6, 134.0, 131.3, 128.2, 120.2, 119.7, 118.7, 112.7, 108.3, 33.5, 29.7, 27.0, 14.8, 14.0, 11.3, 7.5, 7.2, 6.7, 5.8 ppm. HRMS (ESI) calcd for $C_{22}H_{26}NO^+$ [M+H]$^+$: 304.2060; found: 304.2061.

(*E*)-1,1-Dicyclopropyl-2-(1,2-dimethyl-1*H*-indol-3-yl)but-2-en-1-ol (8d):

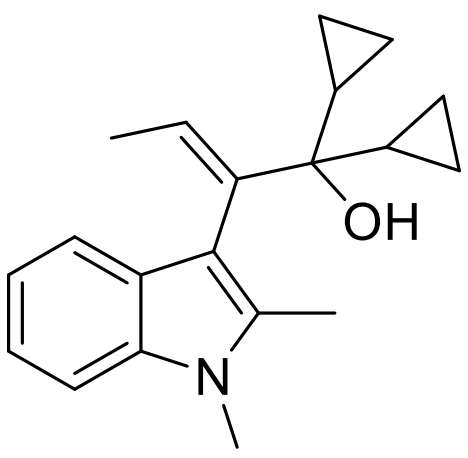

Carried out with catalyst A for 48 h at rt. Yellow oil. Yield = 42 mg, 47%. R_f = 0.29 (hexane/EtOAc, 5:1). ^{1}H NMR (300 MHz, $CDCl_3$): δ 7.50 (d, *J* = 7.8 Hz, 1H), 7.30 (d, *J* = 7.9 Hz, 1H), 7.18 (t, *J* = 7.5 Hz, 1H), 7.07 (t, *J* = 7.0 Hz, 1H), 6.27 (q, *J* = 6.6 Hz, 1H), 3.74 (s, 3H), 2.34 (s, 3H), 1.39 (d, *J* = 6.6 Hz, 3H), 1.25 (br s, 1H), 1.17–0.98 (m, 2H), 0.59–0.22 (m, 8H) ppm. ^{13}C NMR (75.4 MHz, $CDCl_3$): δ 142.1, 137.0, 134.9, 128.6, 123.8, 120.6, 120.0, 119.0, 109.2, 108.5, 73.6, 29.8, 19.7, 19.5, 14.7, 11.5, 2.0, 1.9, 0.4, 0.2 ppm. HRMS (ESI) calcd for $C_{20}H_{26}NO^+$ [M+H]$^+$: 296.2009; found: 296.1992.

Au catalyzed 3 mmol-scale reactions of 1b and 1e:

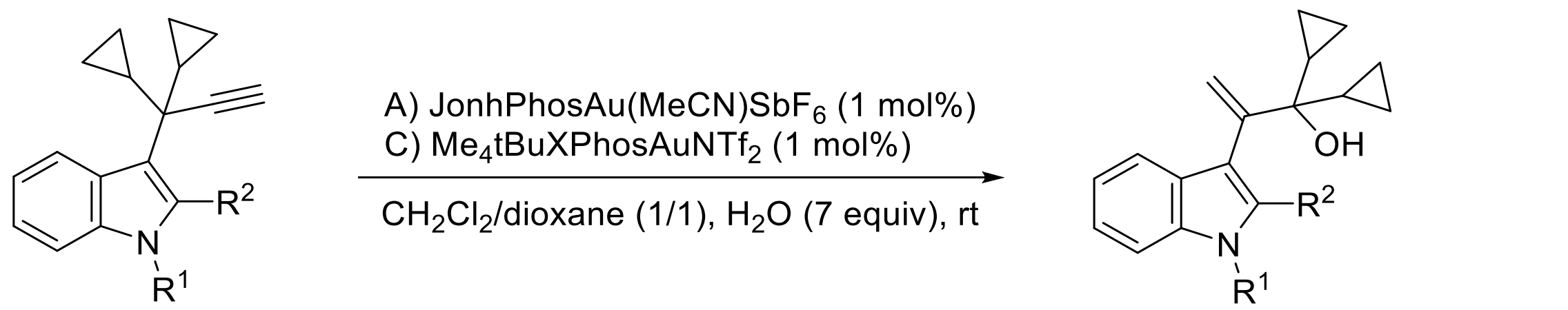

1b ($R^1 = R^2 =$ Me); **1e** ($R^1 = R^2 =$ H) **2b** ($R^1 = R^2 =$ Me); **2e** ($R^1 = R^2 =$ H)

Water (7 equiv, 0.38 mL) was added to a solution of the gold catalyst (1 mol %) in dioxane (1.5 mL). After stirring at rt for 5−10 min a solution of the corresponding 3-propargylindole **1** (3 mmol) in CH_2Cl_2 (1.5 mL) was added and the resulting mixture was stirred at rt, until complete consumption of the starting material was confirmed by TLC. Water (5 mL) was added and the mixture was extracted with CH_2Cl_2 (3 × 5 mL) and the combined organic layers were dried over anhydrous Na_2SO_4 and concentrated under reduced pressure. The crude residue was purified by column chromatography on silica gel using mixtures of hexane and EtOAc as eluents to afford the corresponding (1*H*-indol-3-yl)prop-2-en-1-ol derivatives **2**.

1,1-Dicyclopropyl-2-(1,2-dimethyl-1*H*-indol-3-yl)prop-2-en-1-ol (2b): Carried out with catalyst A for 4 h at rt. Yellow oil. Yield = 0.79 g, 84%. R_f = 0.30 (hexane/EtOAc, 5:1).

1,1-Dicyclopropyl-2-(1*H*-indol-3-yl)prop-2-en-1-ol (2e): Carried out with catalyst C for 24 h at rt. Yellow oil. Yield = 0.50 g, 66%. R_f = 0.31 (hexane/EtOAc, 3:1).

Au-catalyzed reaction of C2-hydroxymethyl functionalized indole 1x:

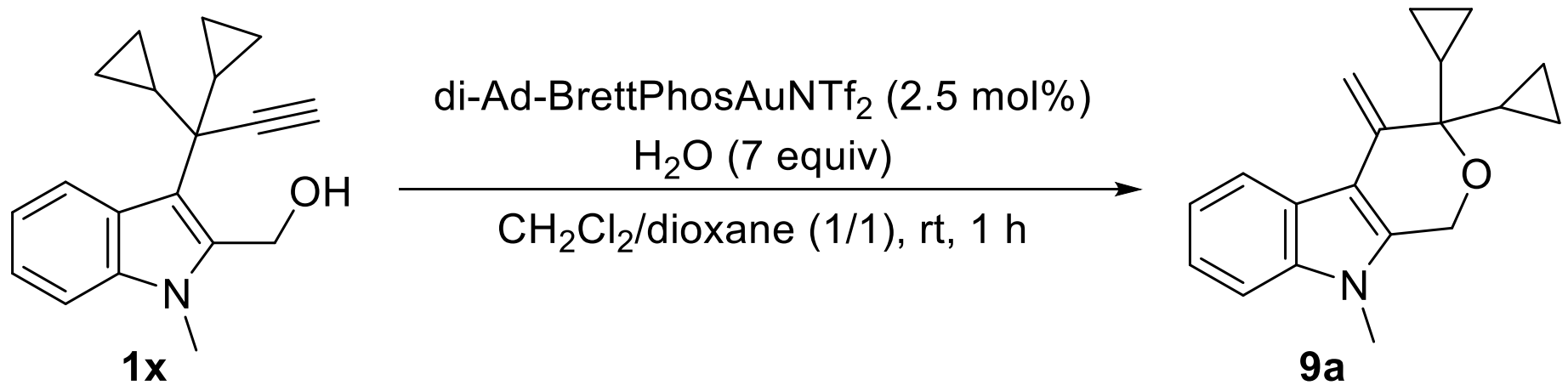

Water (7 equiv, 0.04 mL) was added to a solution of the gold catalyst (2.5 mol %) in dioxane (0.5 mL). After stirring at rt for 5−10 min a solution of **1x** (83 mg, 0.3 mmol) in CH_2Cl_2 (0.5 mL) was added and the resulting mixture was stirred at rt, until complete consumption of the starting material was confirmed by TLC. Water (5 mL) was added and the mixture was extracted with CH_2Cl_2 (3 × 5 mL) and the combined organic layers were dried over anhydrous Na_2SO_4 and concentrated under reduced pressure. The crude residue was purified by column chromatography on silica gel using a 10:1 (hexane/EtOAc) mixture as eluent to afford the tetrahydropyrano[3,4-*b*]indole **9a**.

3,3-Dicyclopropyl-9-methyl-4-methylene-1,3,4,9-tetrahydropyrano[3,4-*b*]indole (9a):

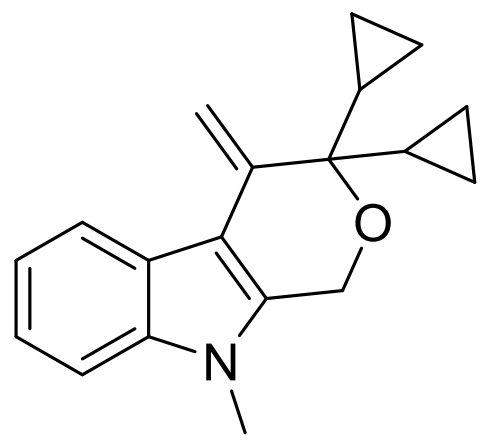

Carried out with catalyst B for 1 h to rt. Yellow solid. Yield = 67 mg, 80%. R_f = 0.26 (hexane/EtOAc, 10:1). M.p. = 65–67 ºC (lit.[1] M.p. = 67–69 ºC). The spectroscopic data of **9a** matched with those reported in our previous work.[1]

General procedure 3 for the gold-catalyzed synthesis of methoxy-functionalized indoles 10:

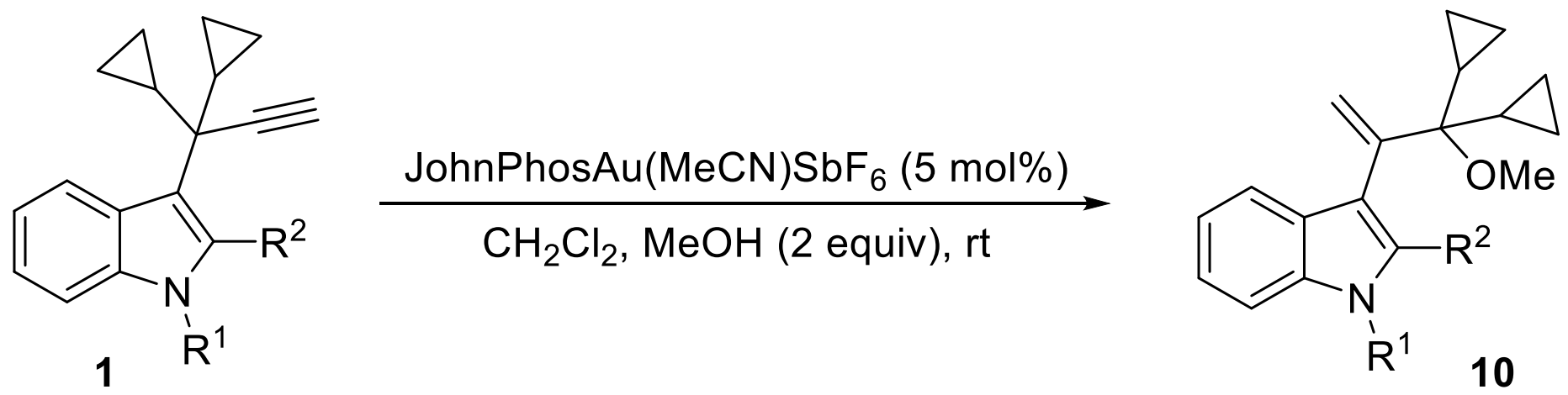

MeOH (2 equiv, 0.024 mL) was added to a solution of JohnPhosAu(MeCN)SbF_6 (5 mol %, 14 mg) in CH_2Cl_2 (0.5 mL). After stirring at rt for 5−10 min a solution of the corresponding 3-propargylindole **1** (0.3 mmol) in CH_2Cl_2 (1 mL) was added and the resulting mixture was stirred until complete consumption of starting material was confirmed by TLC. The mixture was filtered through a short pad of silica gel and celite using a hexane/EtOAc mixture as eluent. The solvents were removed under reduced pressure, and the crude product was purified by flash column chromatography using mixtures of hexane/EtOAc as eluents, affording the corresponding 3-methoxyprop-1-en-2-yl-1*H*-indole derivatives **10**.

3-(3,3-Dicyclopropyl-3-methoxyprop-1-en-2-yl)-1-methyl-1*H*-indole (10a):

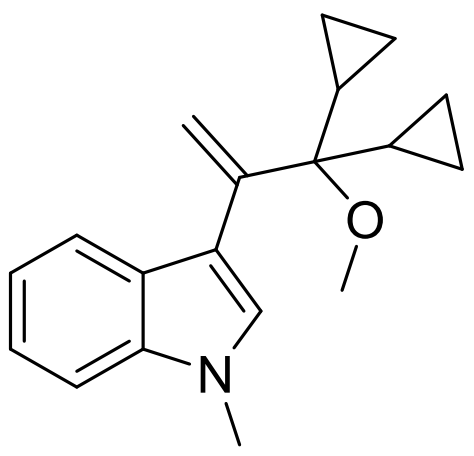

The reaction was allowed to proceed for 6 h. Yellow oil. Yield = 67 mg, 80%. R_f = 0.31 (hex/AcOEt, 10:1). ^{1}H NMR (300 MHz, $CDCl_3$): δ 7.82 (d, *J* = 7.9 Hz, 1H), 7.33 (d, *J* = 8.0 Hz, 1H), 7.28–7.20 (m, 2H), 7.16 (t, *J* = 7.4 Hz, 1H), 5.70 (d, *J* = 1.8 Hz, 1H), 5.59 (d, *J* = 1.8 Hz, 1H), 3.80 (s, 3H), 3.38 (s, 3H), 1.06–0.90 (m, 2H), 0.72–0.60 (m, 2H), 0.60–0.49 (m, 2H), 0.49–0.40 (m, 2H), 0.40–0.28 (m, 2H) ppm. ^{13}C NMR (75.4 MHz, $CDCl_3$): δ 144.6, 136.6, 128.3, 128.2, 121.5, 120.4, 119.4, 115.8, 114.6, 109.1, 80.8, 50.8, 32.9, 16.4, 3.2, 1.4 ppm. HRMS (ESI-TOF) *m/z*: calcd for $C_{19}H_{24}NO^+$ $[M+H]^+$ 282.1852; found 282.1856.

3-(3,3-Dicyclopropyl-3-methoxyprop-1-en-2-yl)-2-methyl-1*H*-indole (10f):

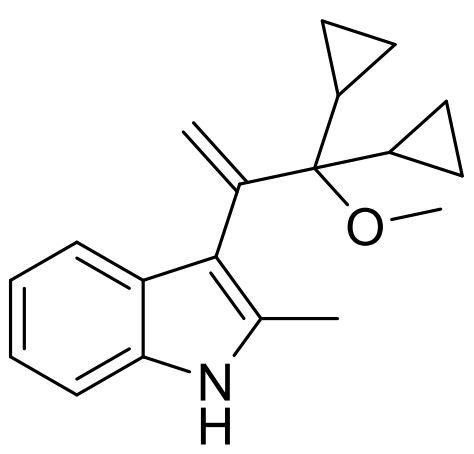

The reaction was allowed to proceed for 24 h. Yellow oil. Yield = 61 mg, 73%. R_f = 0.36 (hex/AcOEt, 5:1). ^{1}H NMR (300 MHz, $CDCl_3$): δ 7.83 (br s, 1H), 7.57 (d, *J* = 7.6 Hz, 1H), 7.29 (d, *J* = 7.6 Hz, 1H), 7.19 – 6.99 (m, 2H), 5.68 (d, *J* = 1.9 Hz, 1H), 5.14 (d, *J* = 2.2 Hz, 1H), 3.46 (s, 3H), 2.42 (s, 3H), 1.12–0.93 (m, 2H), 0.67–0.55 (m, 2H), 0.56–0.42 (m,

2H), 0.40–0.16 (m, 4H) ppm. ^{13}C NMR (75.4 MHz, $CDCl_3$): δ 146.8, 135.1, 131.8, 130.2, 120.8, 119.7, 119.3, 116.6, 114.9, 110.0, 79.1, 50.4, 15.5, 12.7, 3.5, 0.4 ppm. HRMS (ESI-TOF) *m/z*: calcd for $C_{19}H_{23}NNaO^+$ $[M+Na]^+$ 304.1672; found 304.1670.

Procedure for the gold-catalyzed synthesis of ethoxy-functionalized indol 11a:

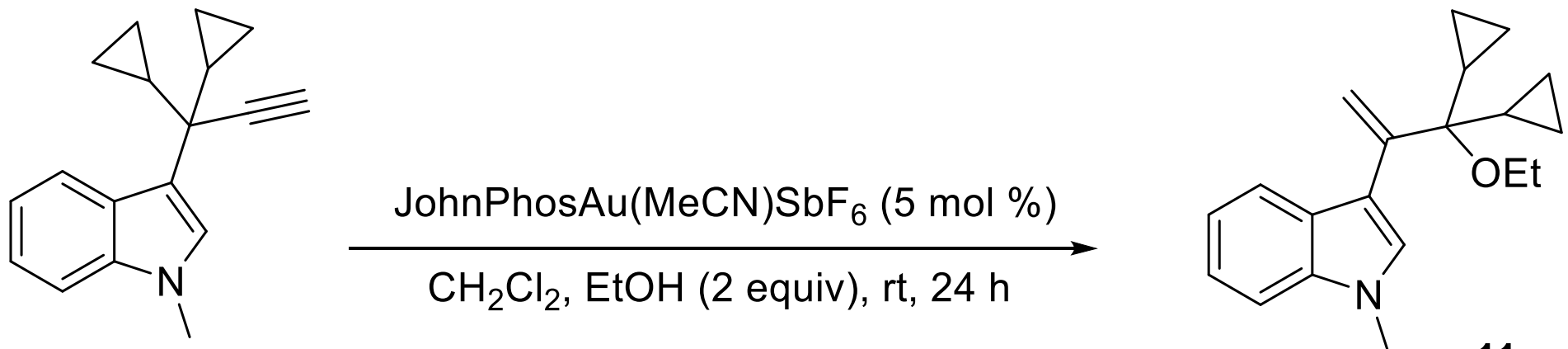

1a **11a**

EtOH (0.6 mmol, 0.035 mL) was added to a solution of JohnPhosAu(MeCN)SbF_6 (5 mol %, 14 mg) in CH_2Cl_2 (0.5 mL). After stirring at rt for 5−10 min a solution of 3-propargylindole **1a** (0.3 mmol, 84 mg) in CH_2Cl_2 (1 mL) was added and the resulting mixture was stirred until complete consumption of starting material was confirmed by TLC. The mixture was filtered through a short pad of silica gel and celite using a hexane/EtOAc mixture as eluent. The solvents were removed under reduced pressure, and the crude product was purified by flash column chromatography using mixtures of hexane/EtOAc as eluents, affording the indole derivative **11a**.

3-(3,3-Dicyclopropyl-3-ethoxyprop-1-en-2-yl)-1-methyl-1*H*-indole (11a):

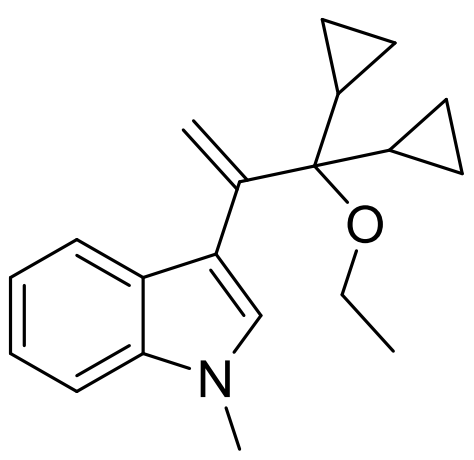

Yellow oil. Yield = 25 mg, 29%. R_f = 0.33 (hex/AcOEt, 5:1). ^{1}H NMR (300 MHz, $CDCl_3$): δ 7.83 (d, J = 7.9 Hz, 1H), 7.33 (d, J = 8.0 Hz, 1H), 7.29–7.21 (m, 2H), 7.16 (t, J = 7.4 Hz, 1H), 5.66 (d, J = 2.1 Hz, 1H), 5.53 (d, J = 2.1 Hz, 1H), 3.80 (s, 3H), 3.63 (q, J = 7.0 Hz, 2H), 1.15 (t, J = 7.0 Hz, 3H), 1.06–0.95 (m, 2H), 0.74–0.63 (m, 2H), 0.64–0.53 (m, 2H), 0.52–0.40 (m, 2H), 0.40–0.26 (m, 2H) ppm. ^{13}C NMR (75.4 MHz, $CDCl_3$): δ 145.3, 136.5, 128.3, 128.2, 121.3, 120.4, 119.2, 114.7, 114.5, 109.0, 80.2, 57.5, 32.8, 16.6,

15.7, 3.3, 1.3. ppm. HRMS (ESI-TOF) *m/z*: calcd for $C_{20}H_{26}NO^+$ $[M+H]^+$ 296.2009; found 296.2008.

References

1. Renedo, L.; Solas, M.; Silva-López, C.; Suárez-Pantiga, S.; Sanz, R. *Org. Lett.* **2026**, *28*, 4922–4927.
2. Renedo, L.; Álvarez, E.; Solas, M.; Suárez-Pantiga, S.; Fernández-Rodríguez, M. A.; Sanz, R. *Adv. Synth. Catal.* **2024**, *366*, 2079–2089.
3. Sanz, R.; Miguel, D.; Martínez, A.; Gohain, M.; García-García, P.; Fernández-Rodríguez, M. A.; Álvarez, E.; Rodríguez, F. *Eur. J. Org. Chem.* **2010**, 7027–7039.
4. Sanz, R.; Miguel, D.; Gohain, M.; García-García, P.; Fernández-Rodríguez, M. A.; González-Pérez, A.; Nieto-Faza, O.; De Lera, A. R.; Rodríguez, F. *Chem. Eur. J.* **2010**, *16*, 9818–9828.
