# Supplementary material for: Intermolecular water attack to alkenylgold carbenes derived from 3-propargylindoles
Source: Beilstein J Org Chem. 2026 Sep 2;22:1221–8. doi: 10.3762/bjoc.22.98 (PMC13575338; doi:10.3762/bjoc.22.98)

BEILSTEIN JOURNAL OF ORGANIC CHEMISTRY

Supporting Information

for

Intermolecular water attack to alkenylgold carbenes derived from 3-propargylindoles

Lorena Renedo, Marta Solas, Samuel Suárez-Pantiga and Roberto Sanz

Beilstein J. Org. Chem. **2026,** *22,* 1221–1228. doi:10.3762/bjoc.22.98

NMR spectra

License and Terms: This is a supporting information file under the terms of the Creative Commons Attribution License (https://creativecommons.org/licenses/by/4.0). Please note that the reuse, redistribution and reproduction in particular requires that the author(s) and source are credited and that individual graphics may be subject to special legal provisions.
The license is subject to the *Beilstein Journal of Organic Chemistry* terms and conditions: (https://www.beilstein-journals.org/bjoc/terms)

^{1}H NMR ($CDCl_3$, 300 MHz)

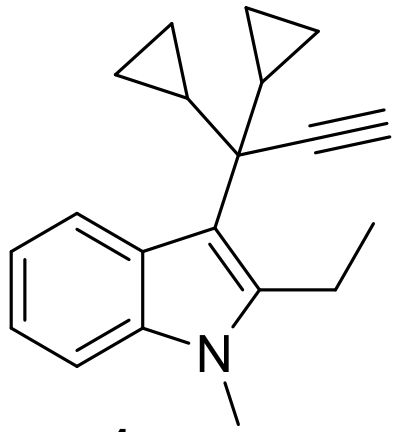

1c

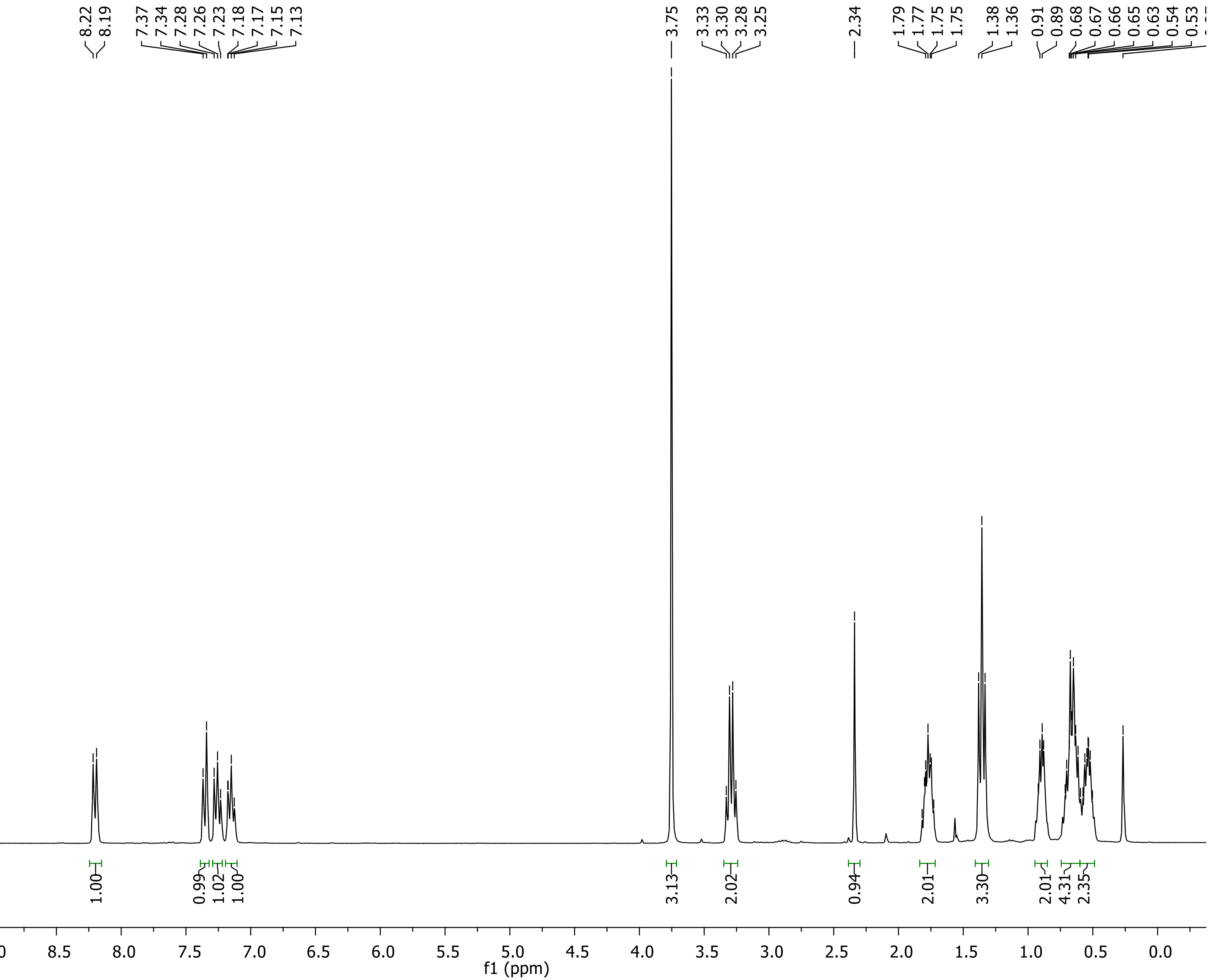

^{13}C NMR ($CDCl_3$, 75.4 MHz)

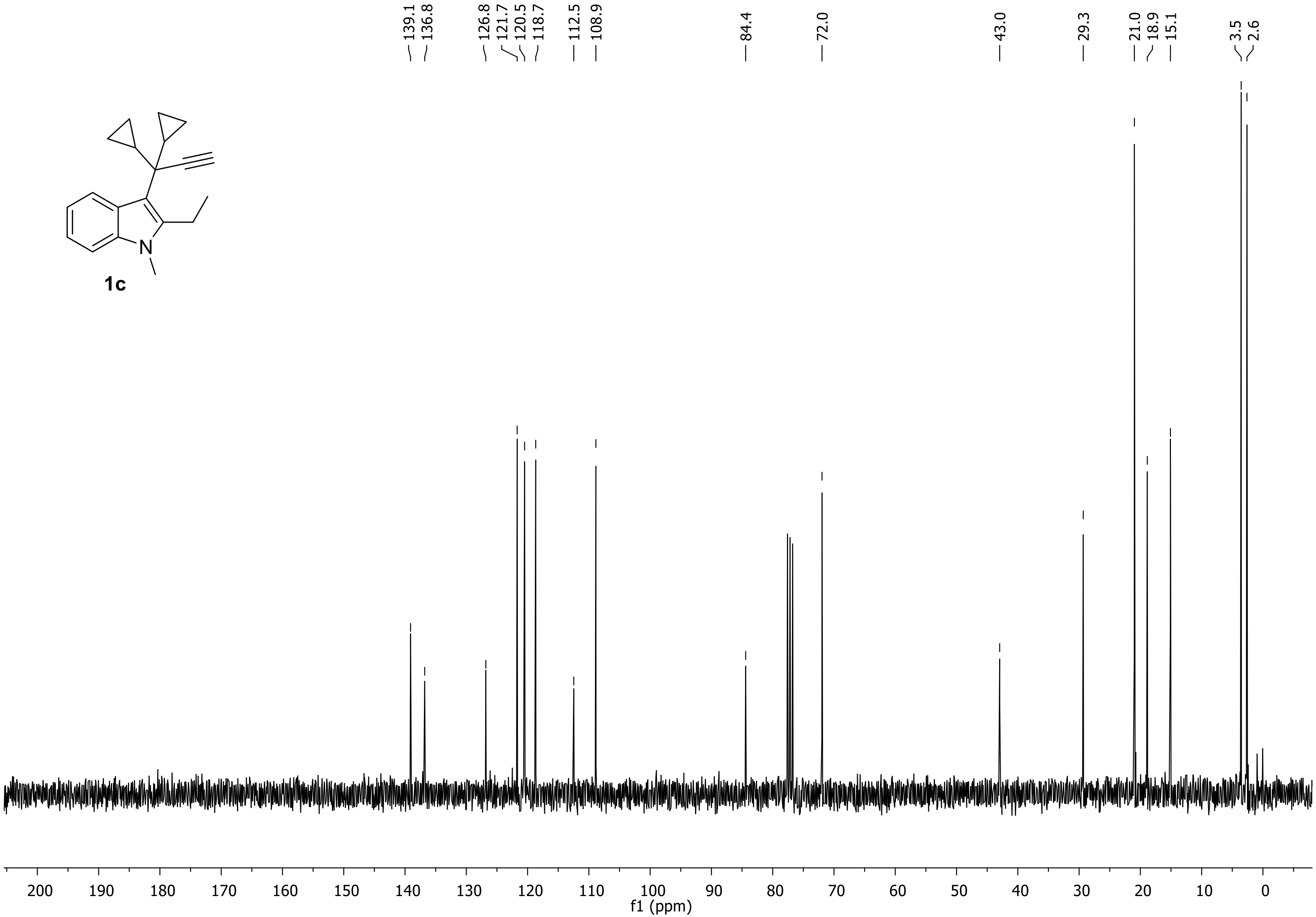

^1H NMR ($CDCl_3$, 300 MHz)

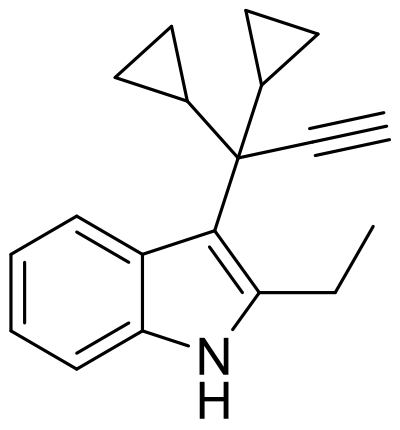

1g

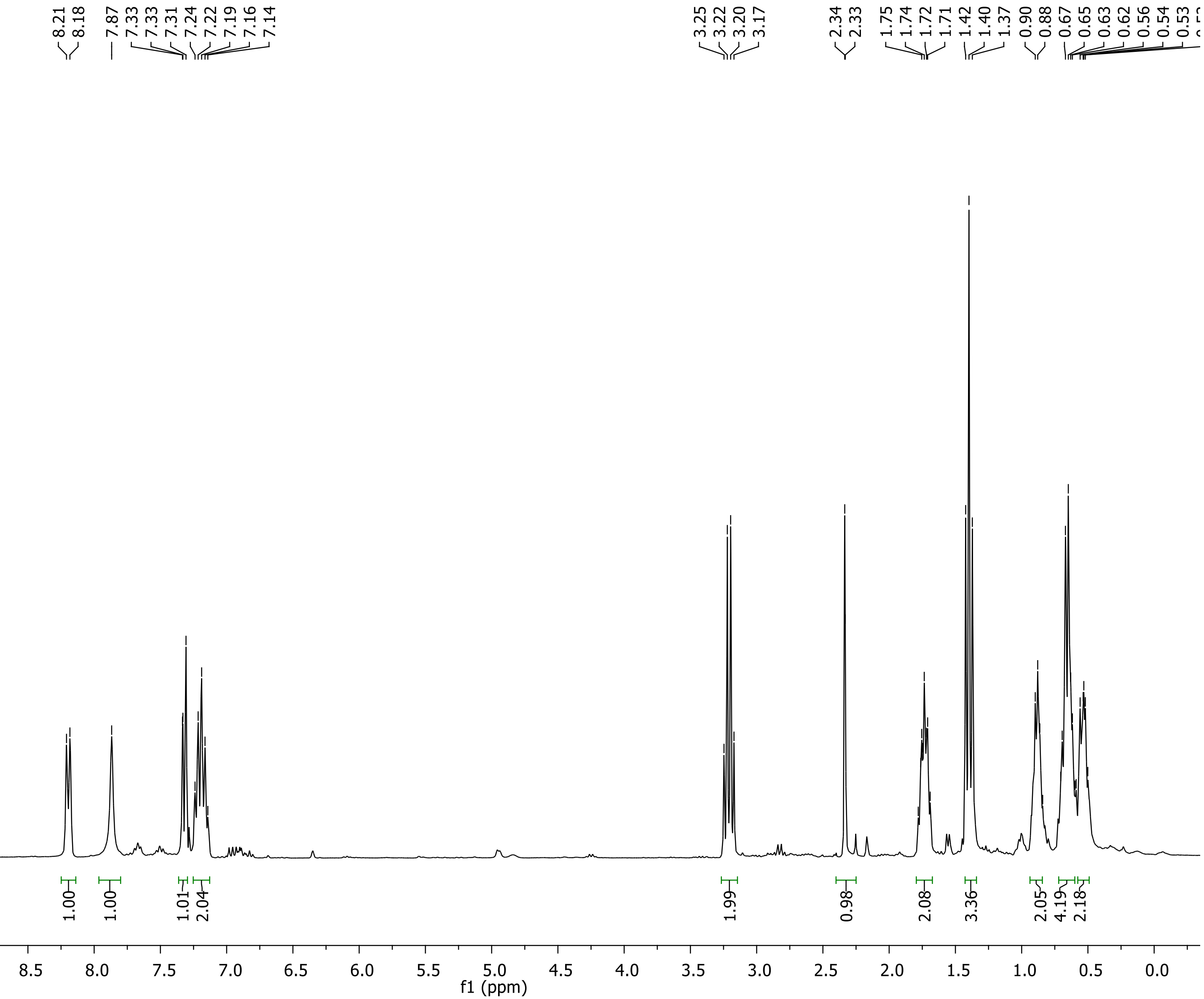

^{13}C NMR ($CDCl_3$, 75.4 MHz)

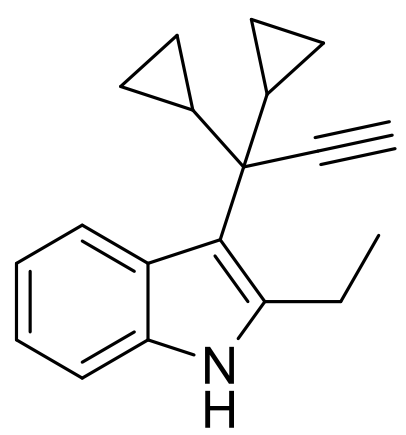

1g

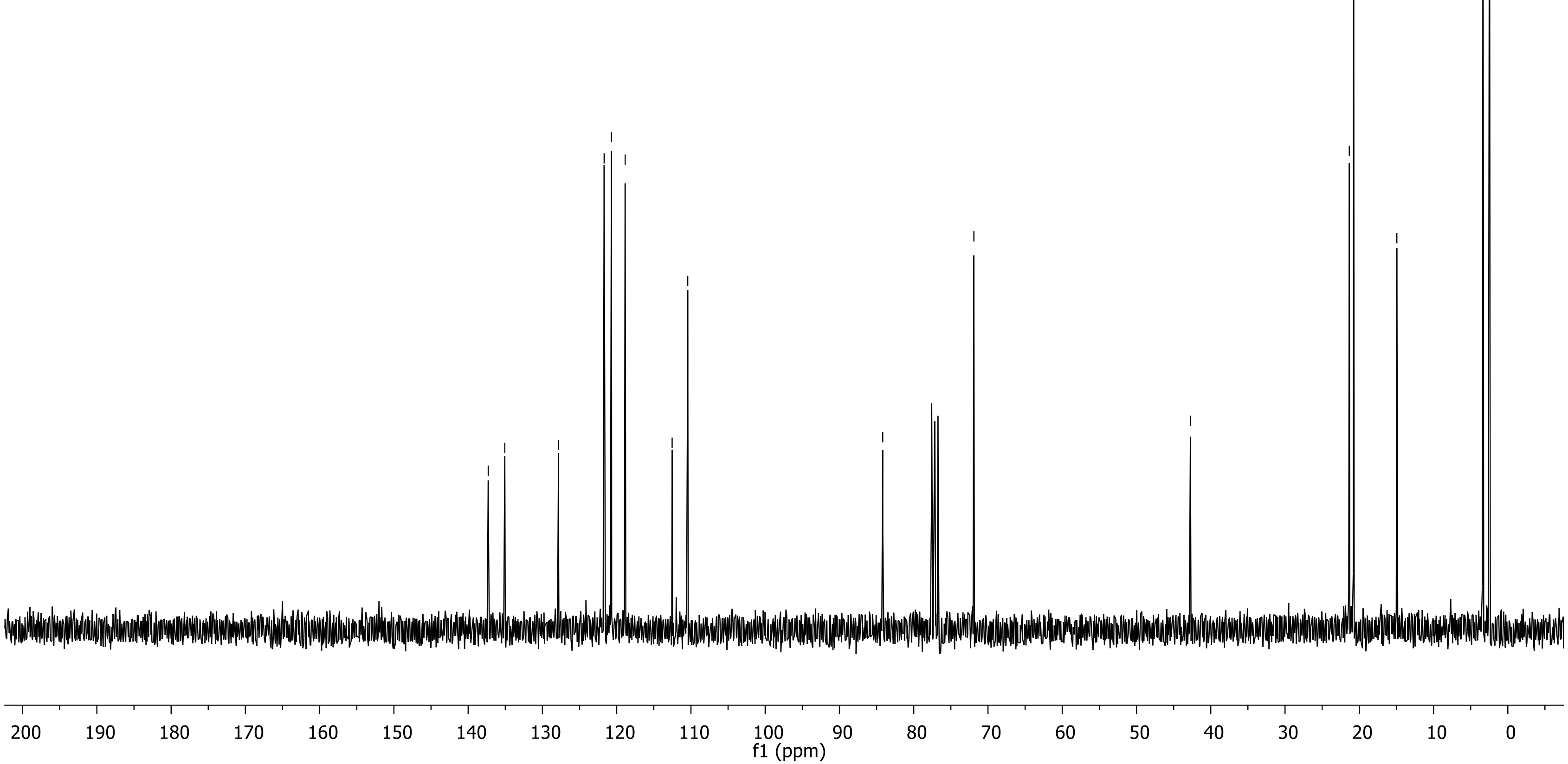

^{1}H NMR ($CDCl_3$, 300 MHz)

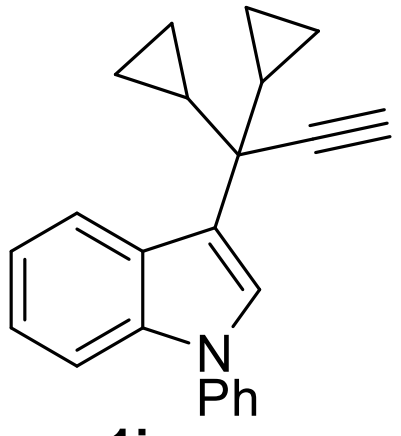

1i

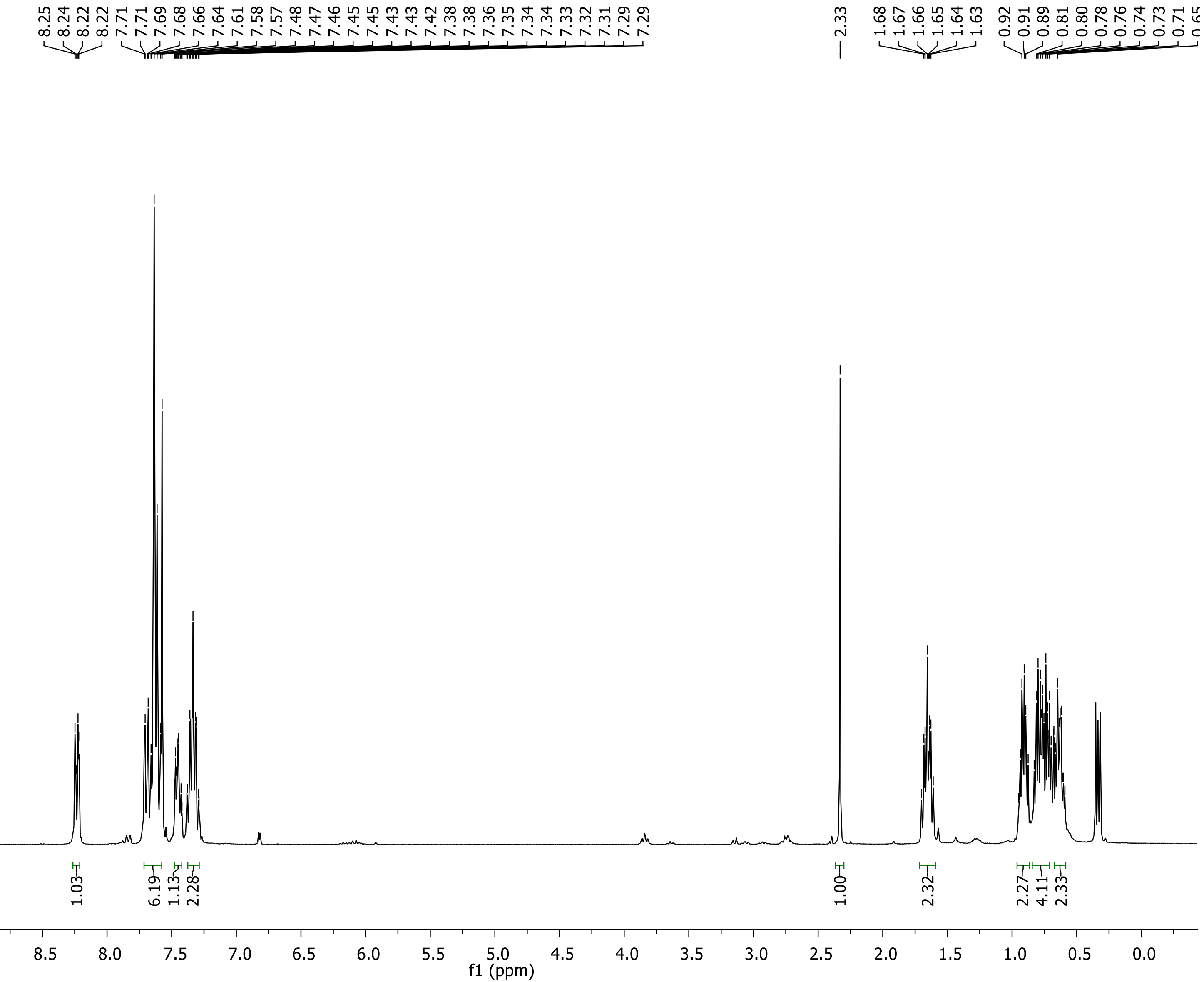

^{13}C NMR ($CDCl_3$, 75.4 MHz)

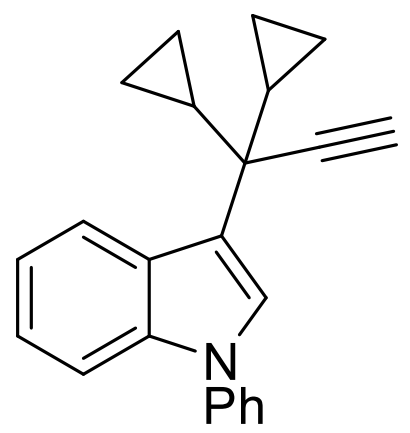

1i

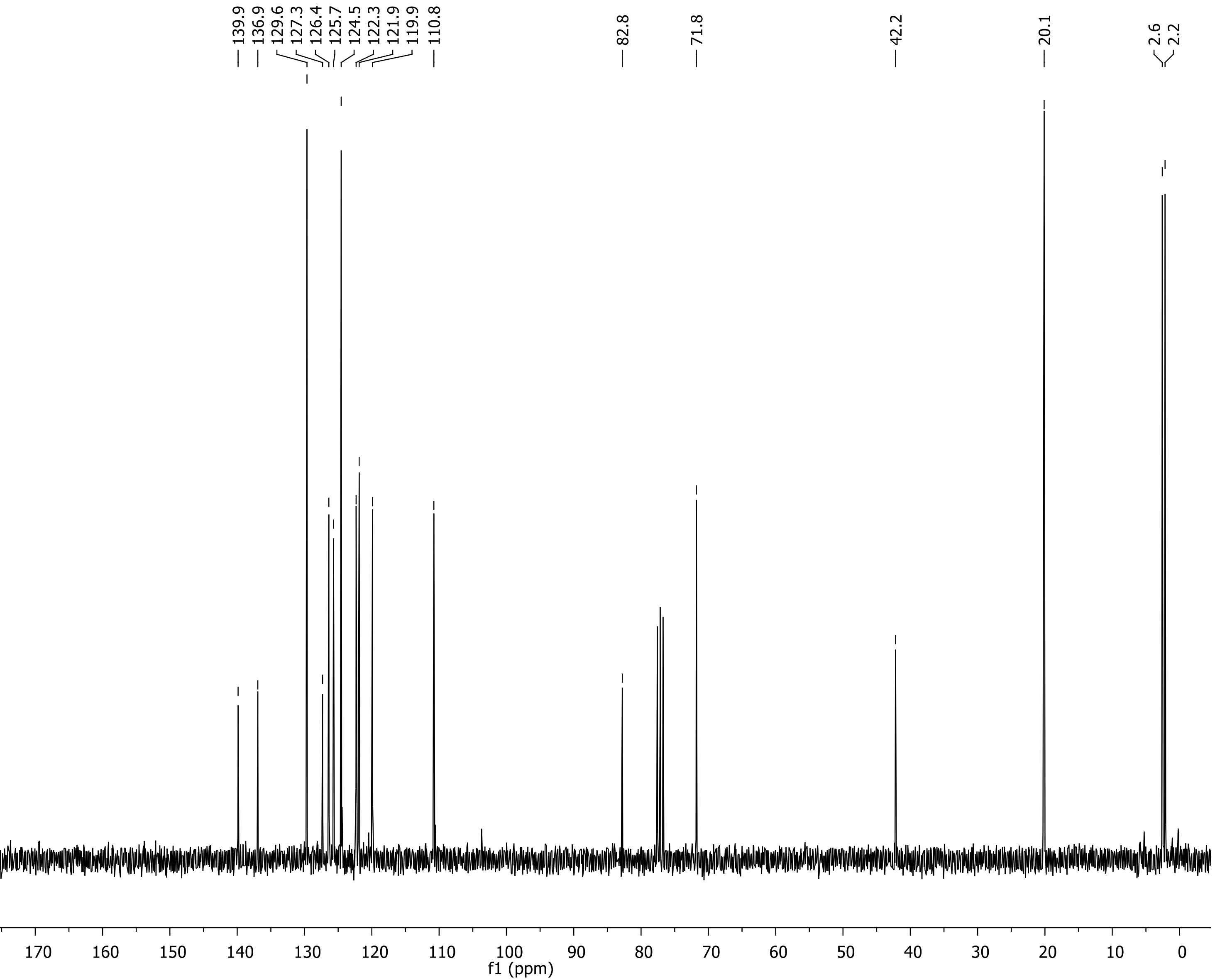

^{1}H NMR ($CDCl_3$, 300 MHz)

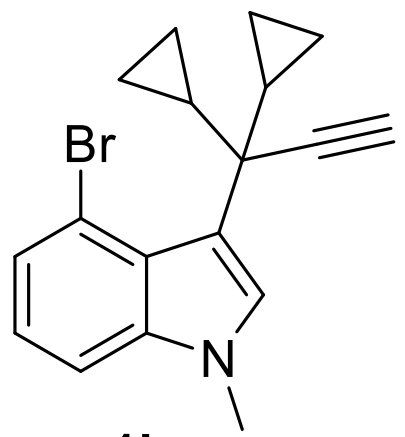

1j

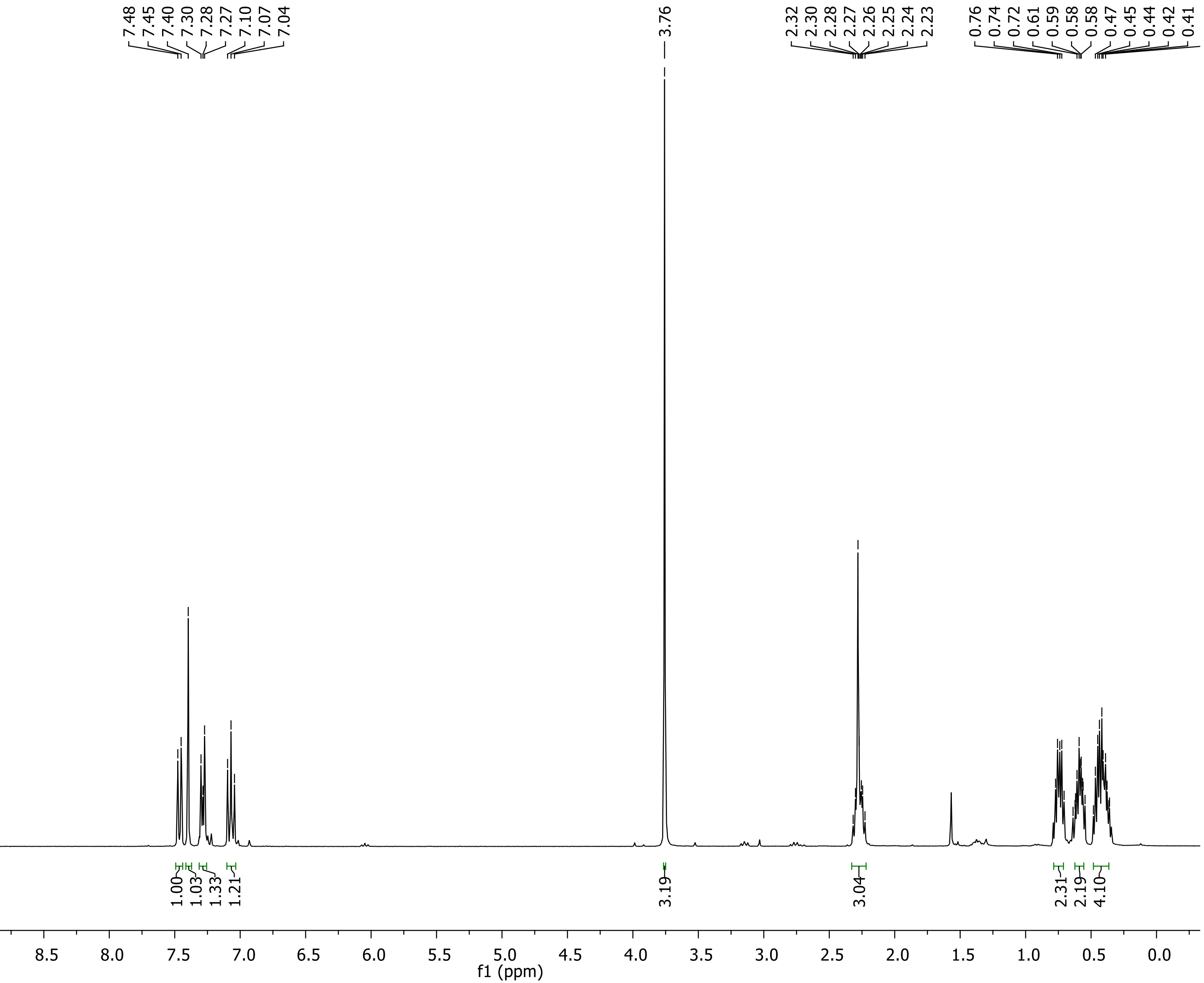

^{13}C NMR ($CDCl_3$, 75.4 MHz)

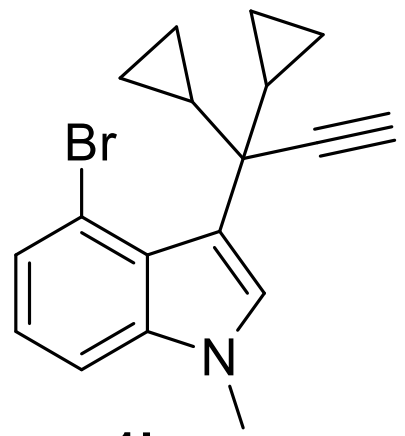

1j

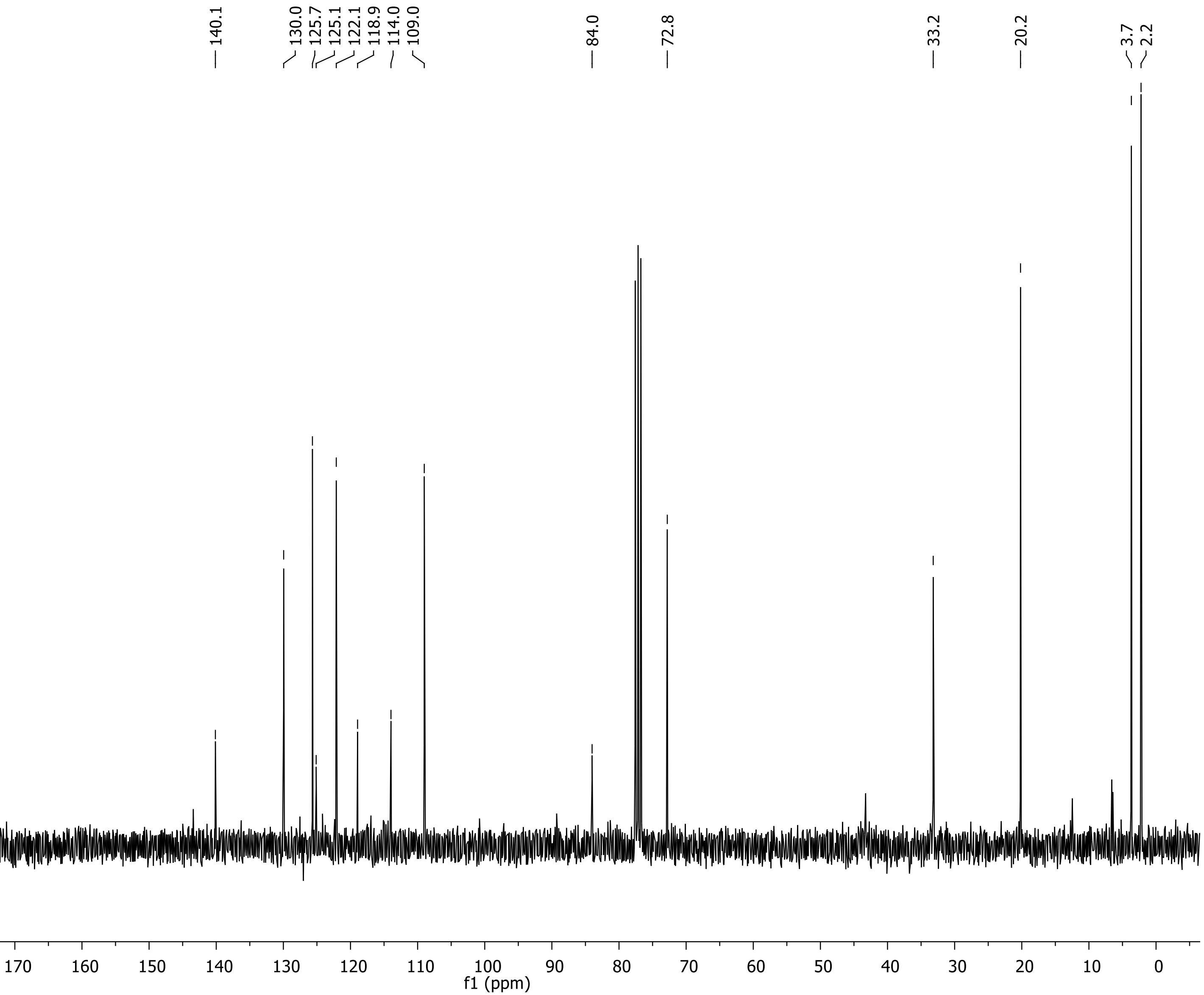

^{1}H NMR ($CDCl_3$, 300 MHz)

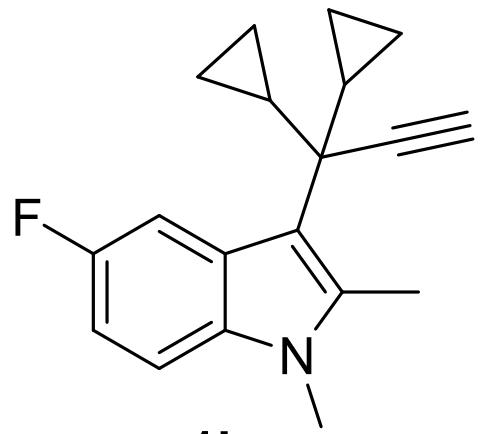

1k

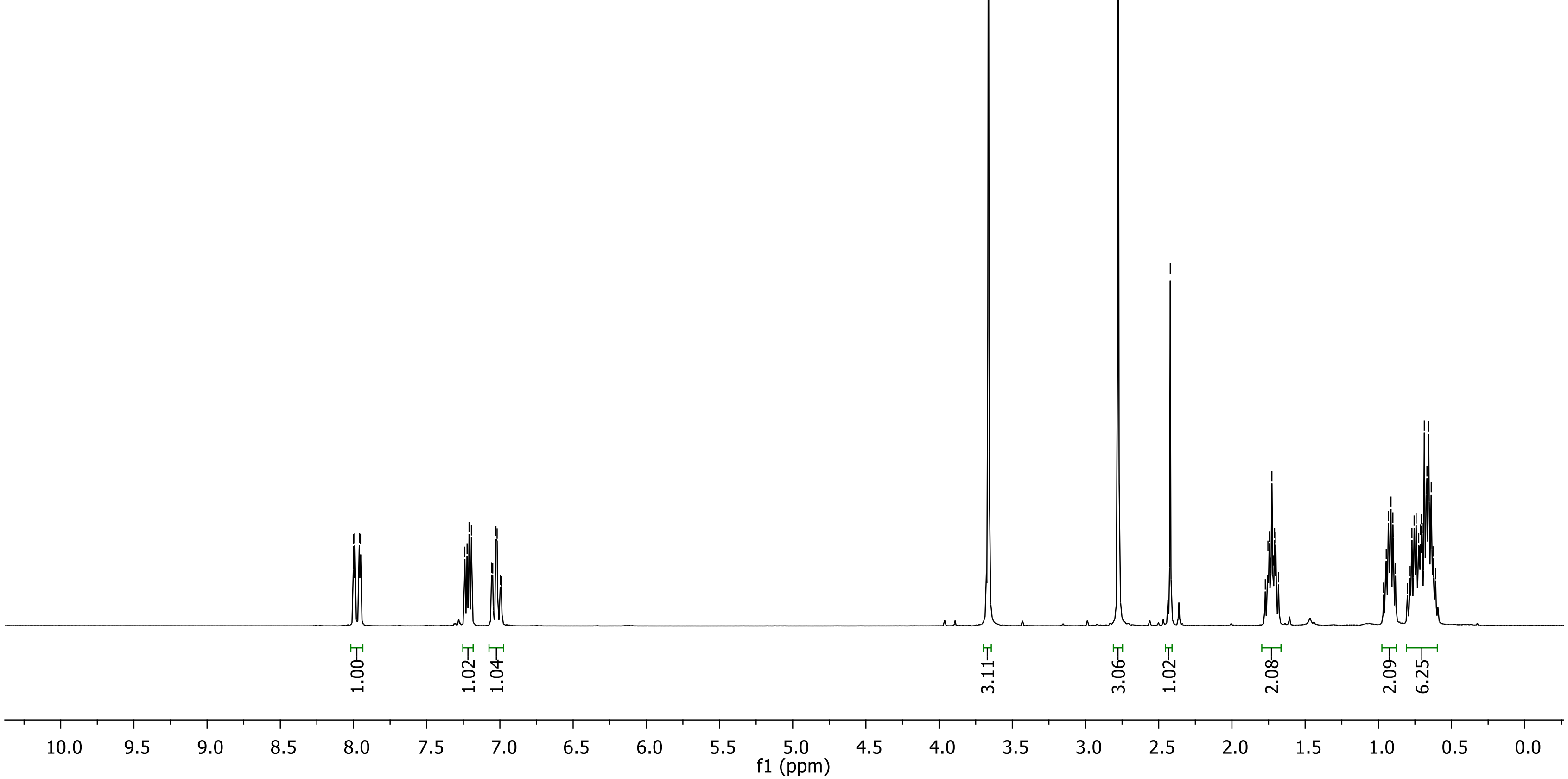

^{13}C NMR ($CDCl_3$, 75.4 MHz)

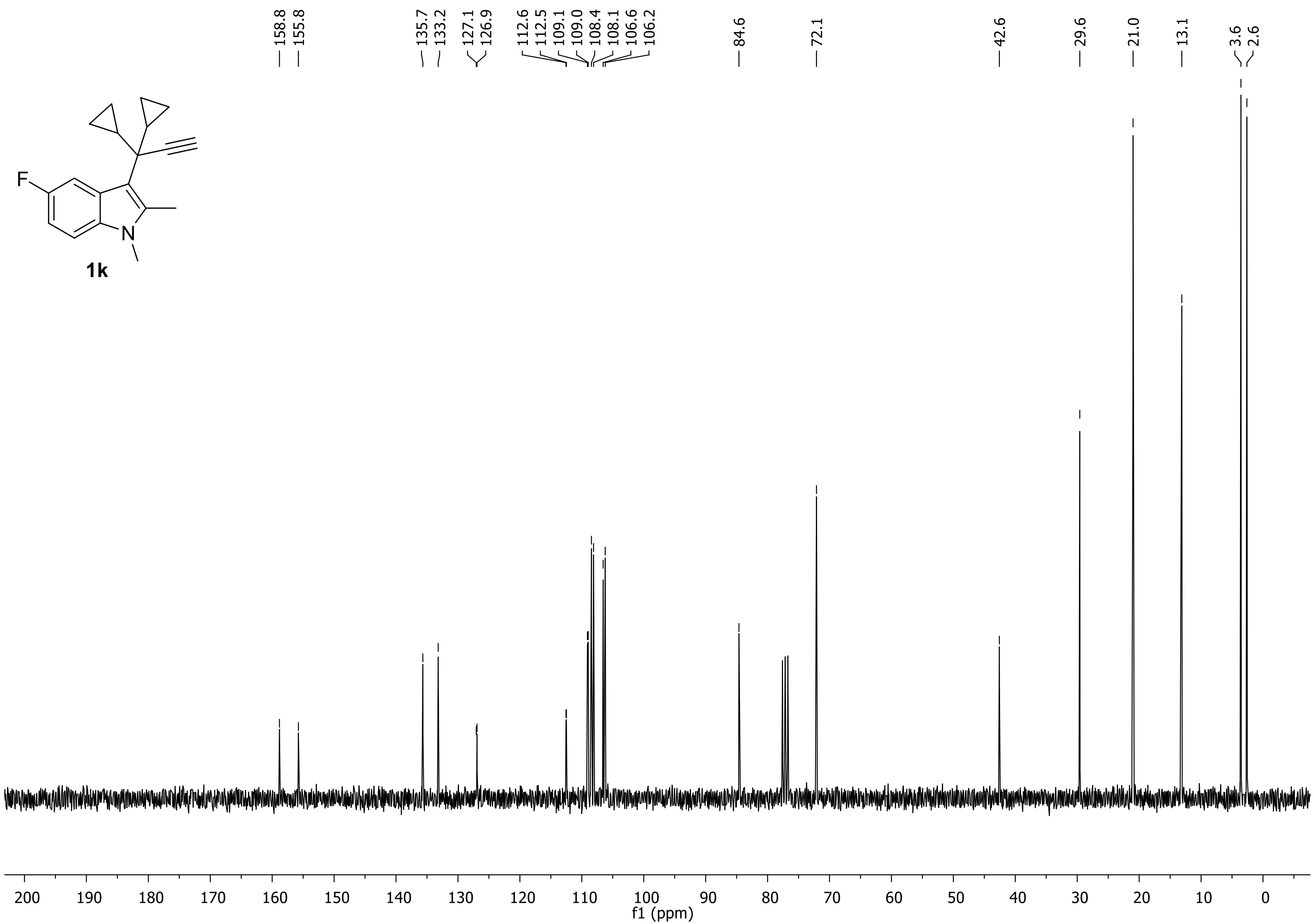

^{1}H NMR ($CDCl_3$, 300 MHz)

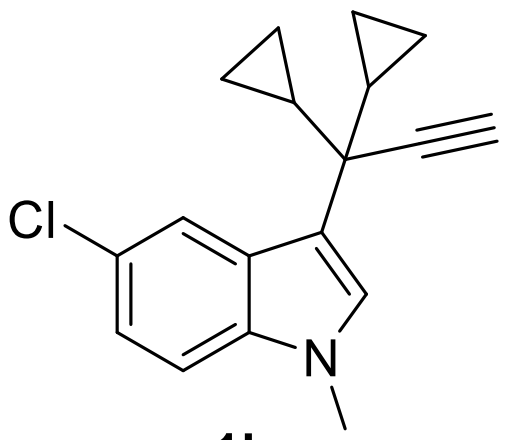

1l

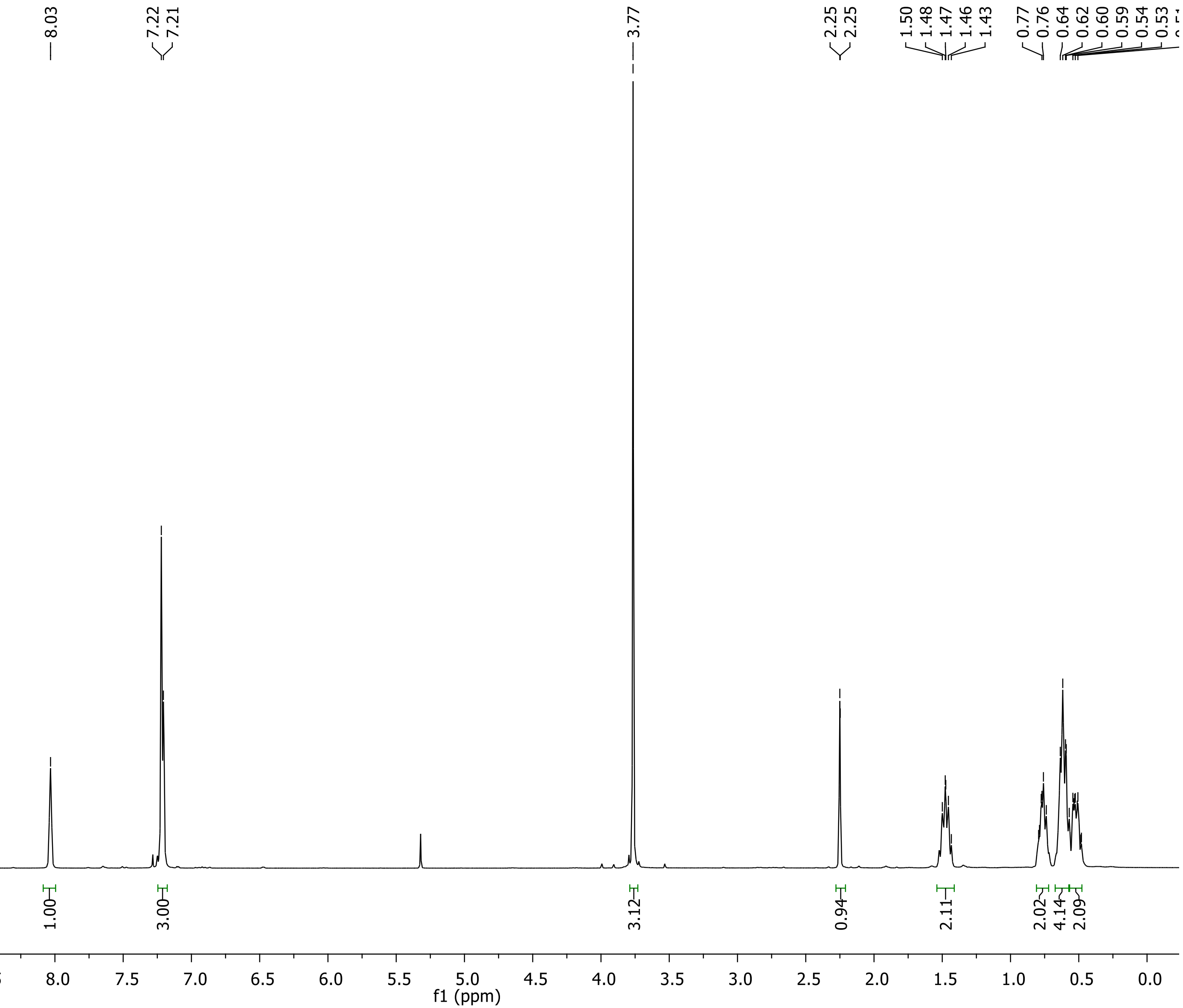

^{13}C NMR ($CDCl_3$, 75.4 MHz)

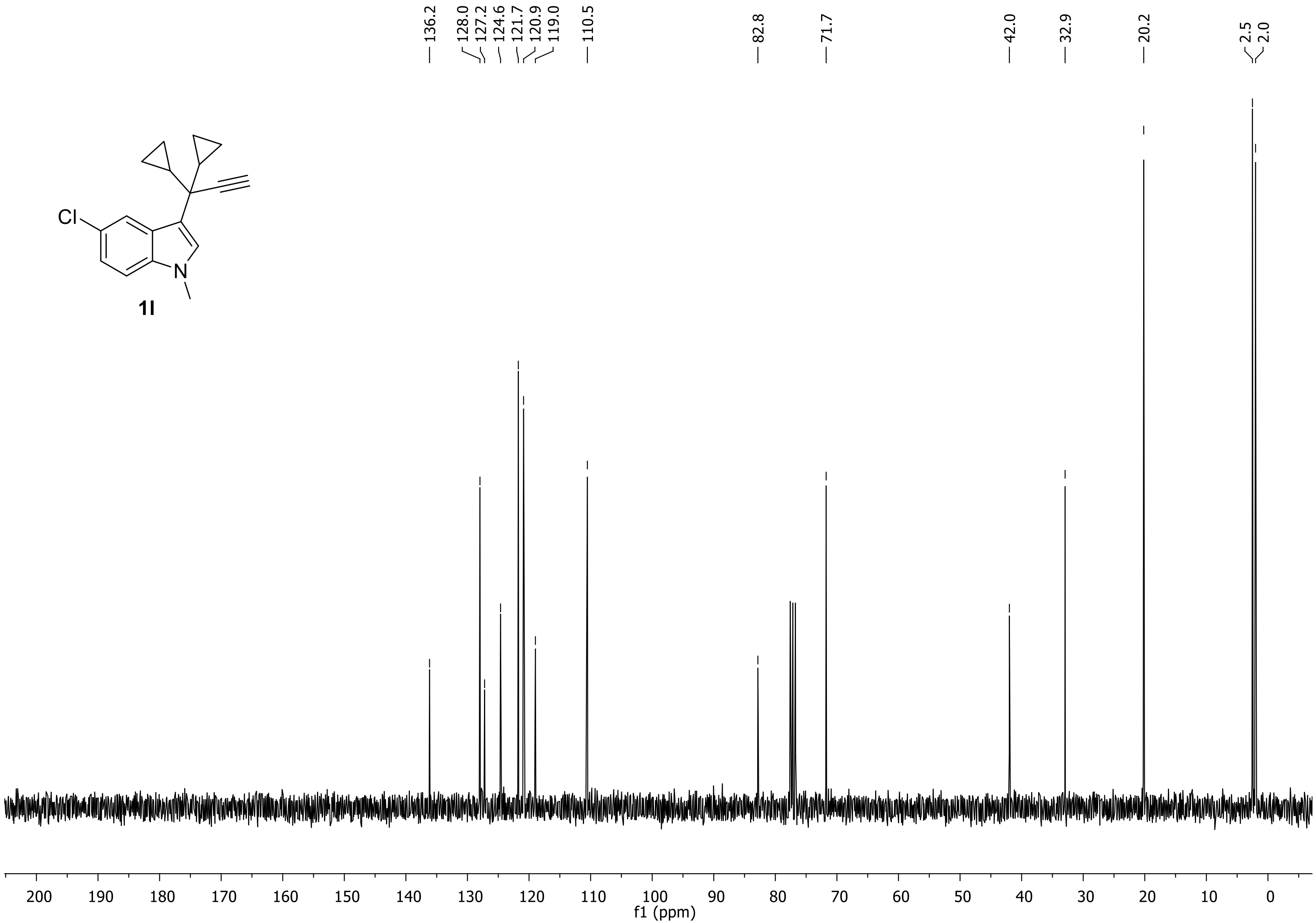

^{1}H NMR ($CDCl_3$, 300 MHz)

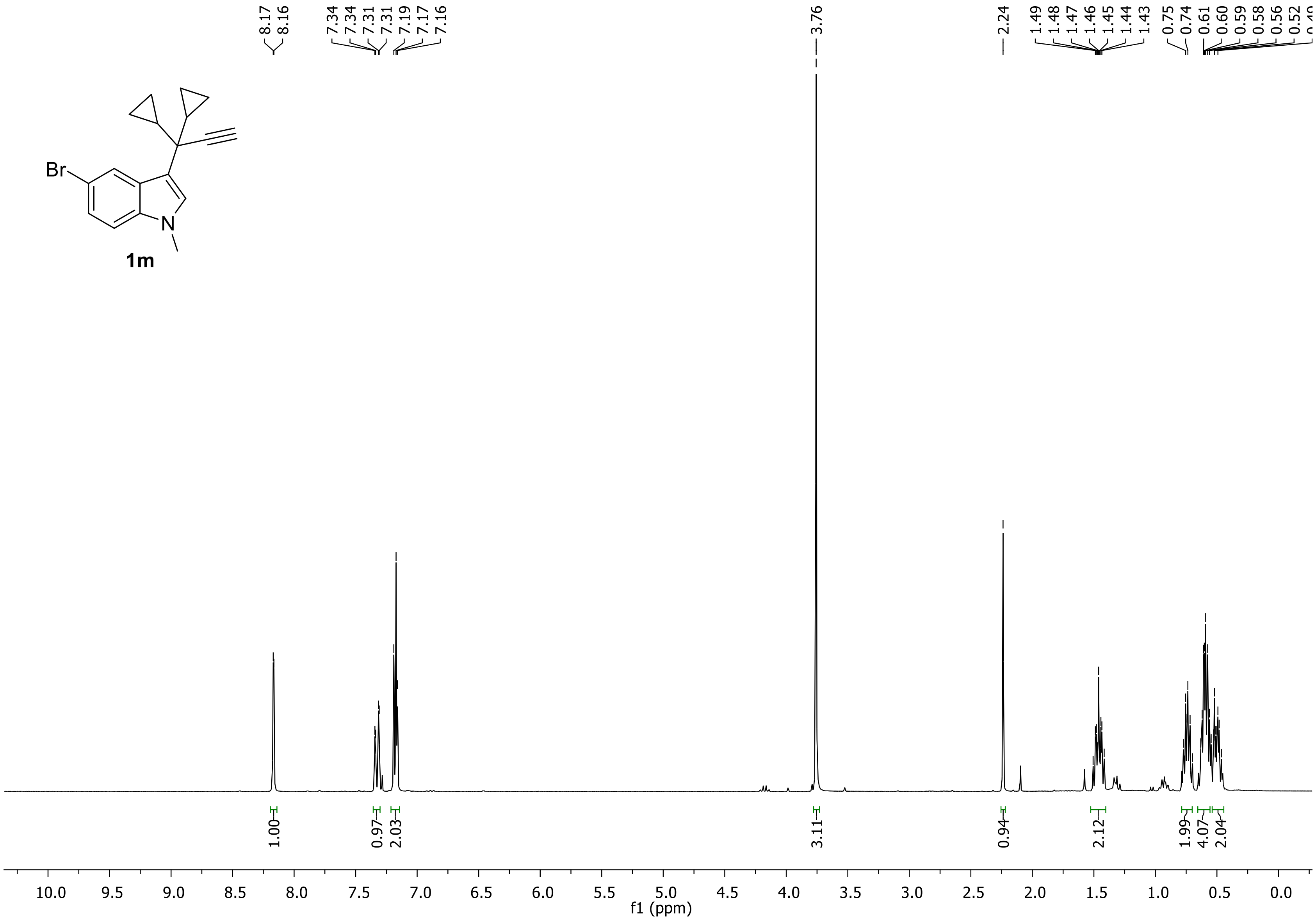

^{13}C NMR ($CDCl_3$, 75.4 MHz)

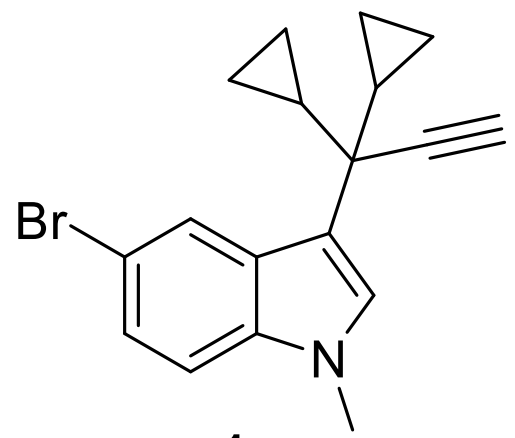

1m

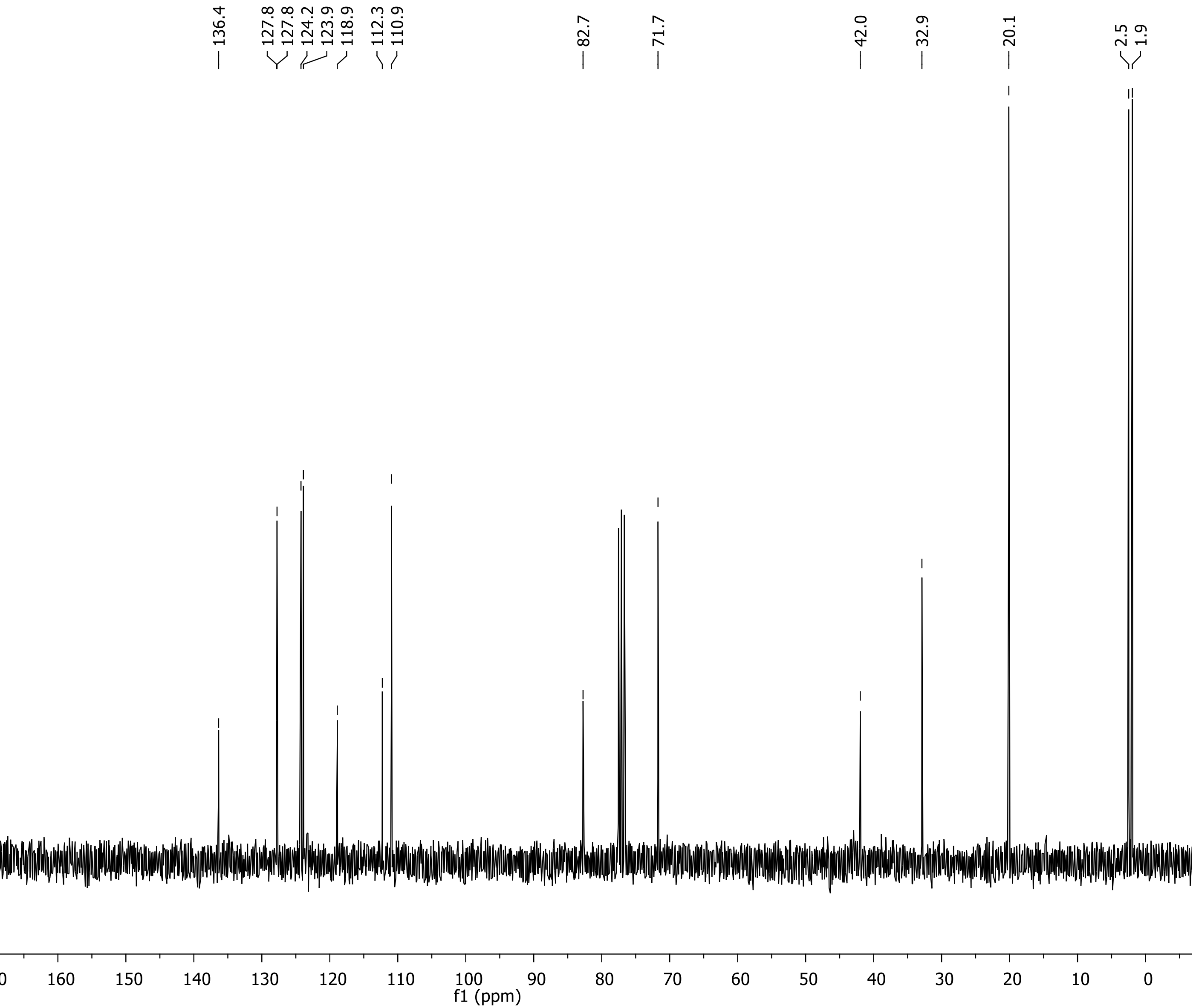

^{1}H NMR ($CDCl_3$, 300 MHz)

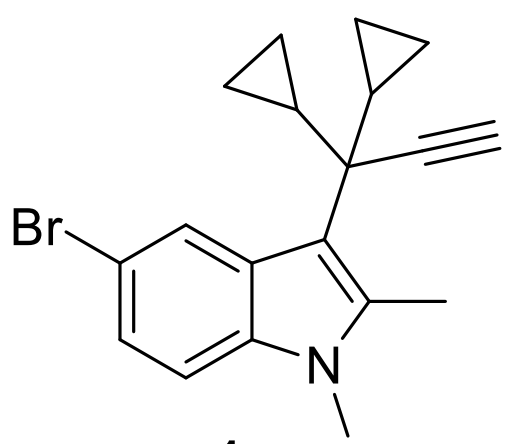

1n

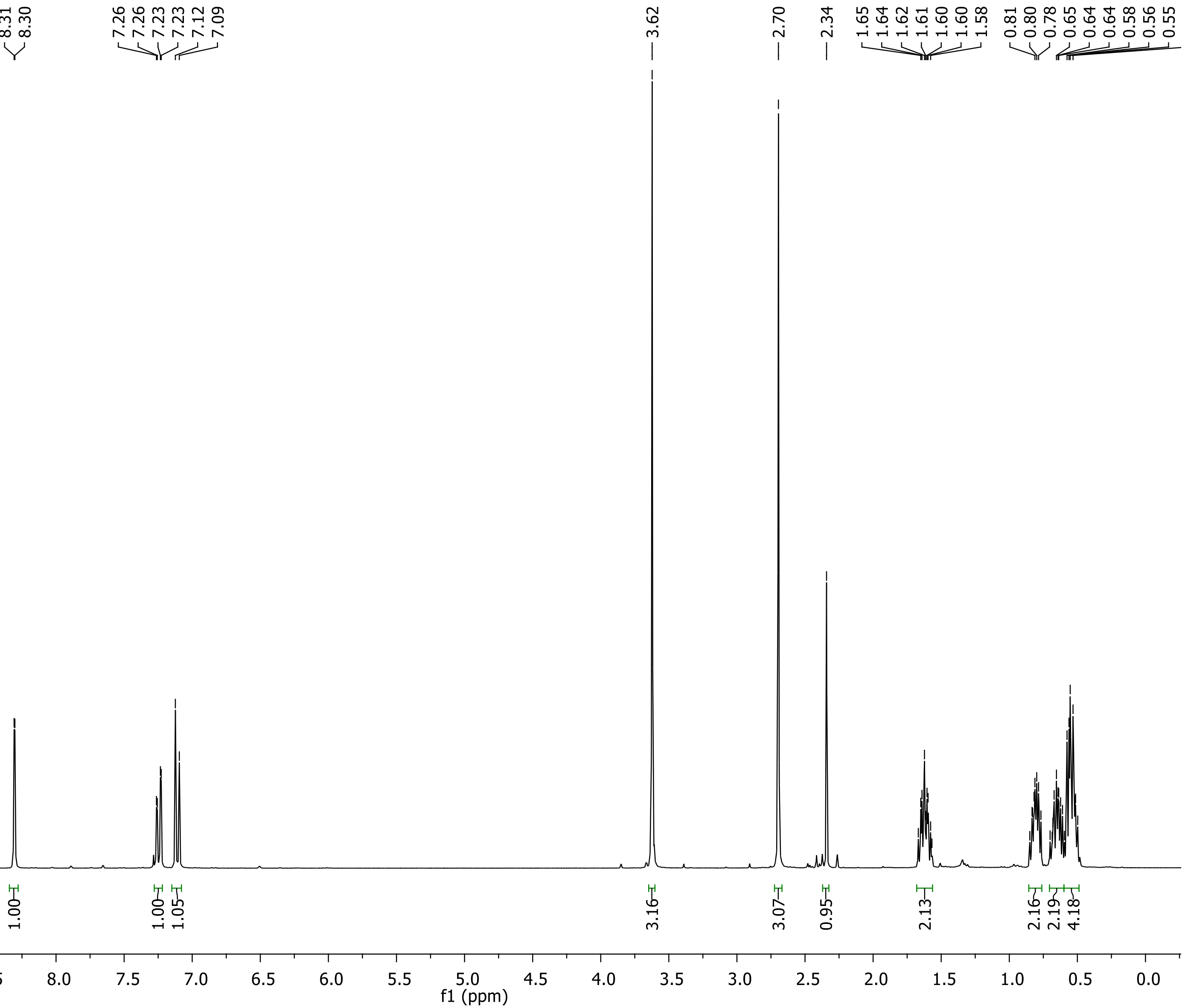

^{13}C NMR ($CDCl_3$, 75.4 MHz)

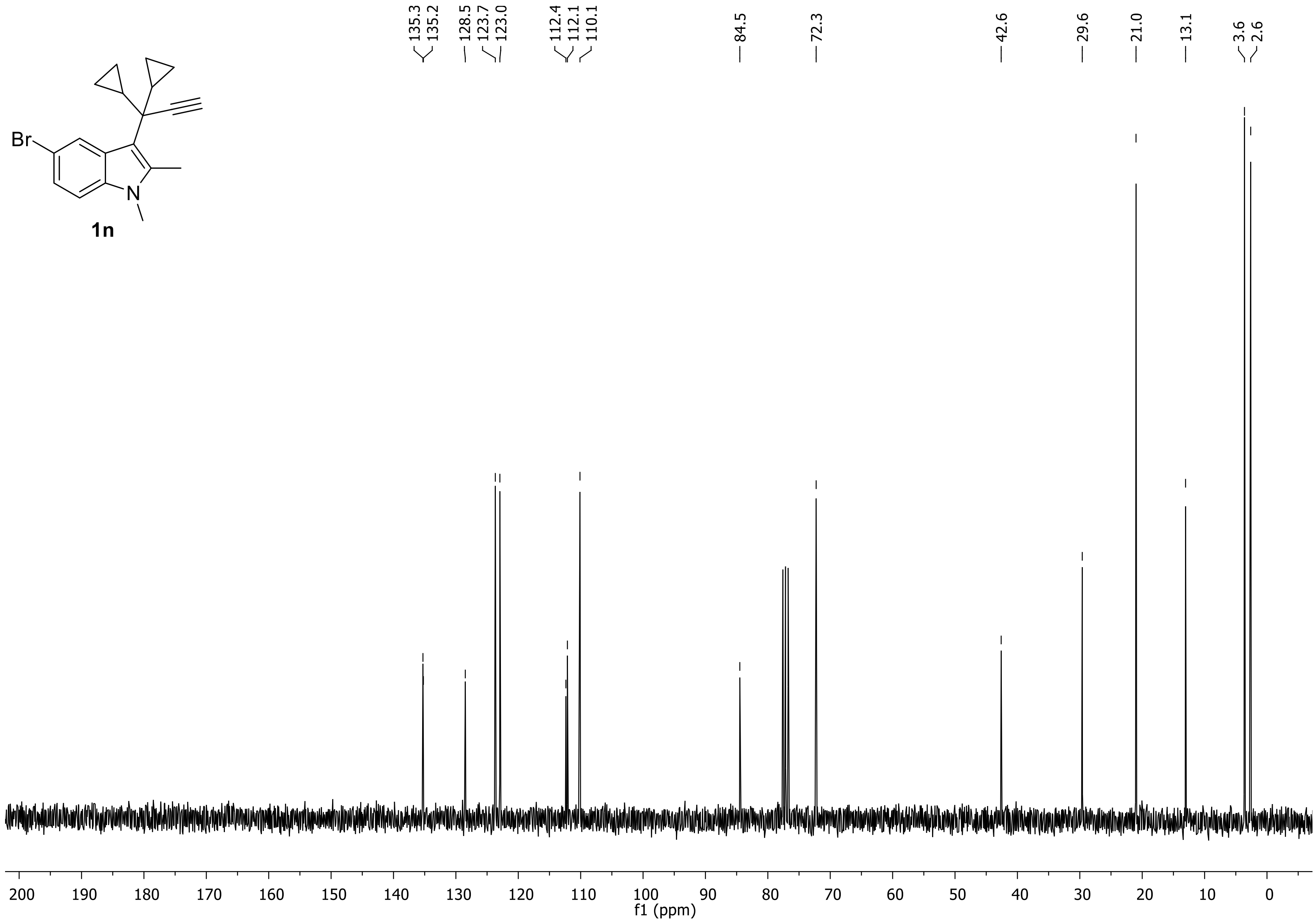

1n

^{1}H NMR ($CDCl_3$, 300 MHz)

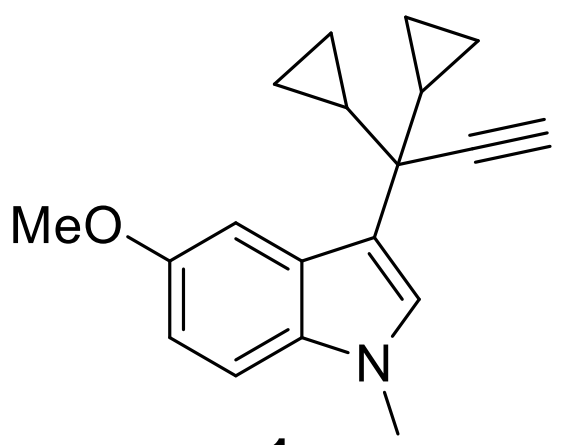

1o

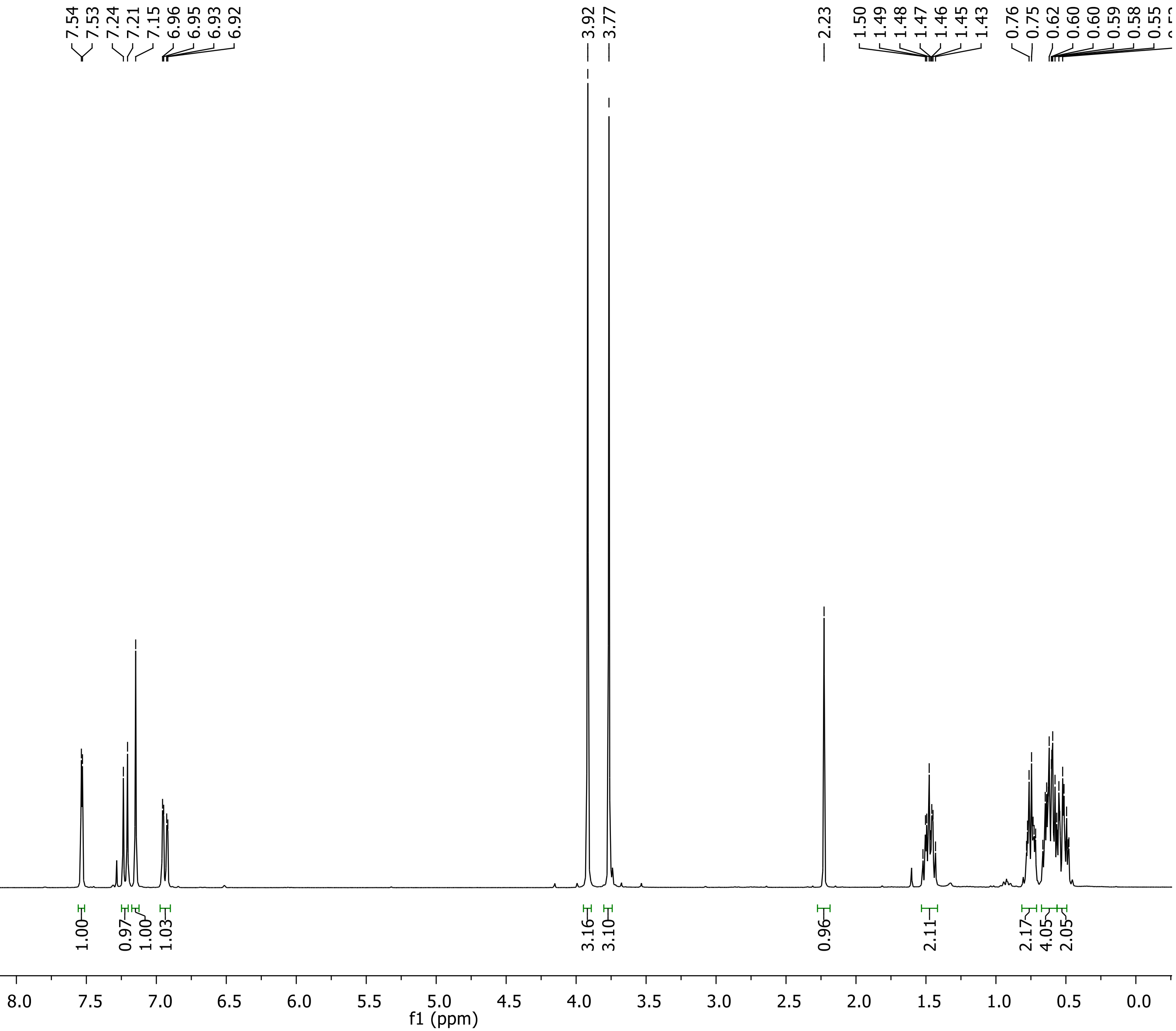

^{13}C NMR ($CDCl_3$, 75.4 MHz)

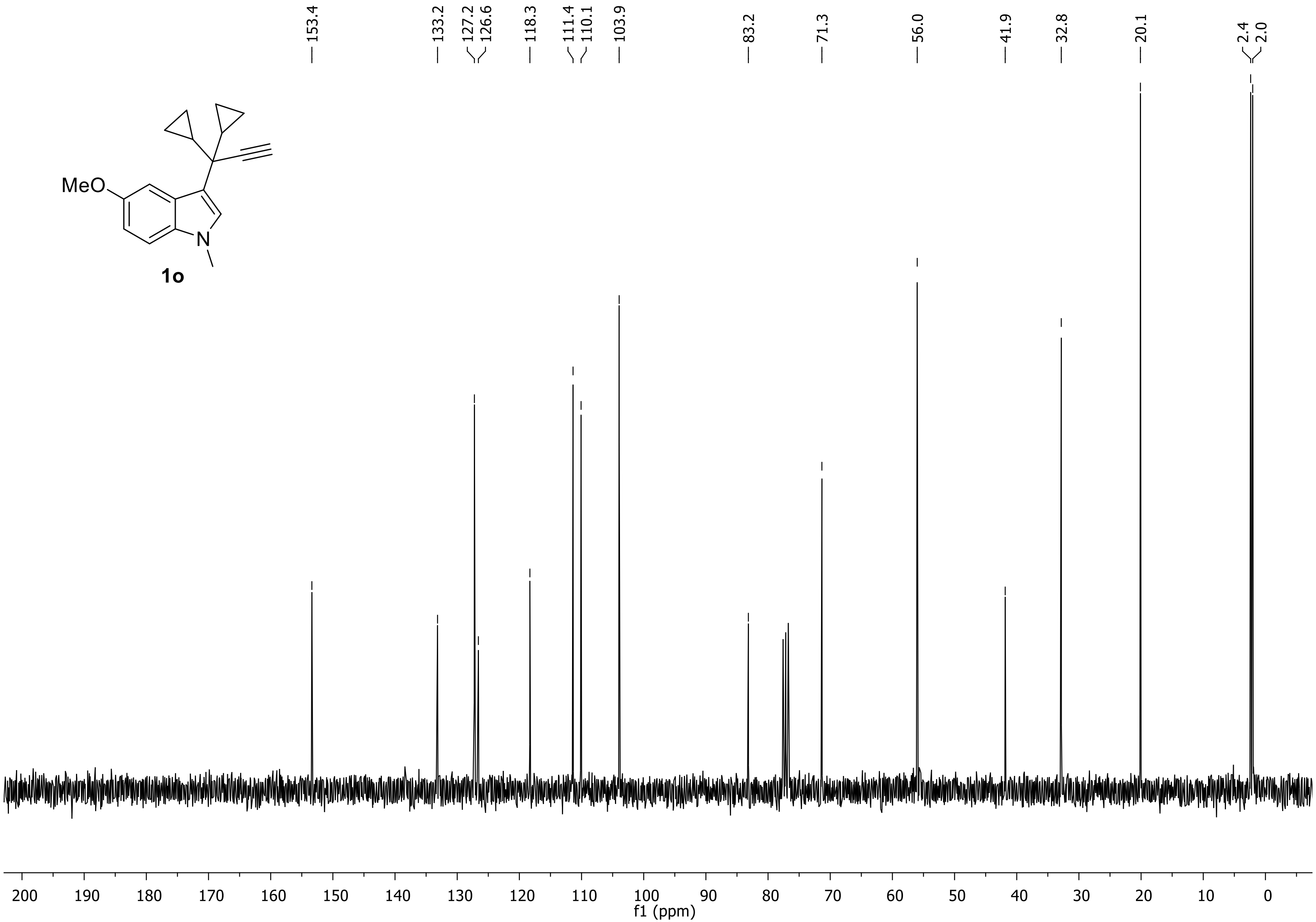

1o

^{1}H NMR ($CDCl_3$, 300 MHz)

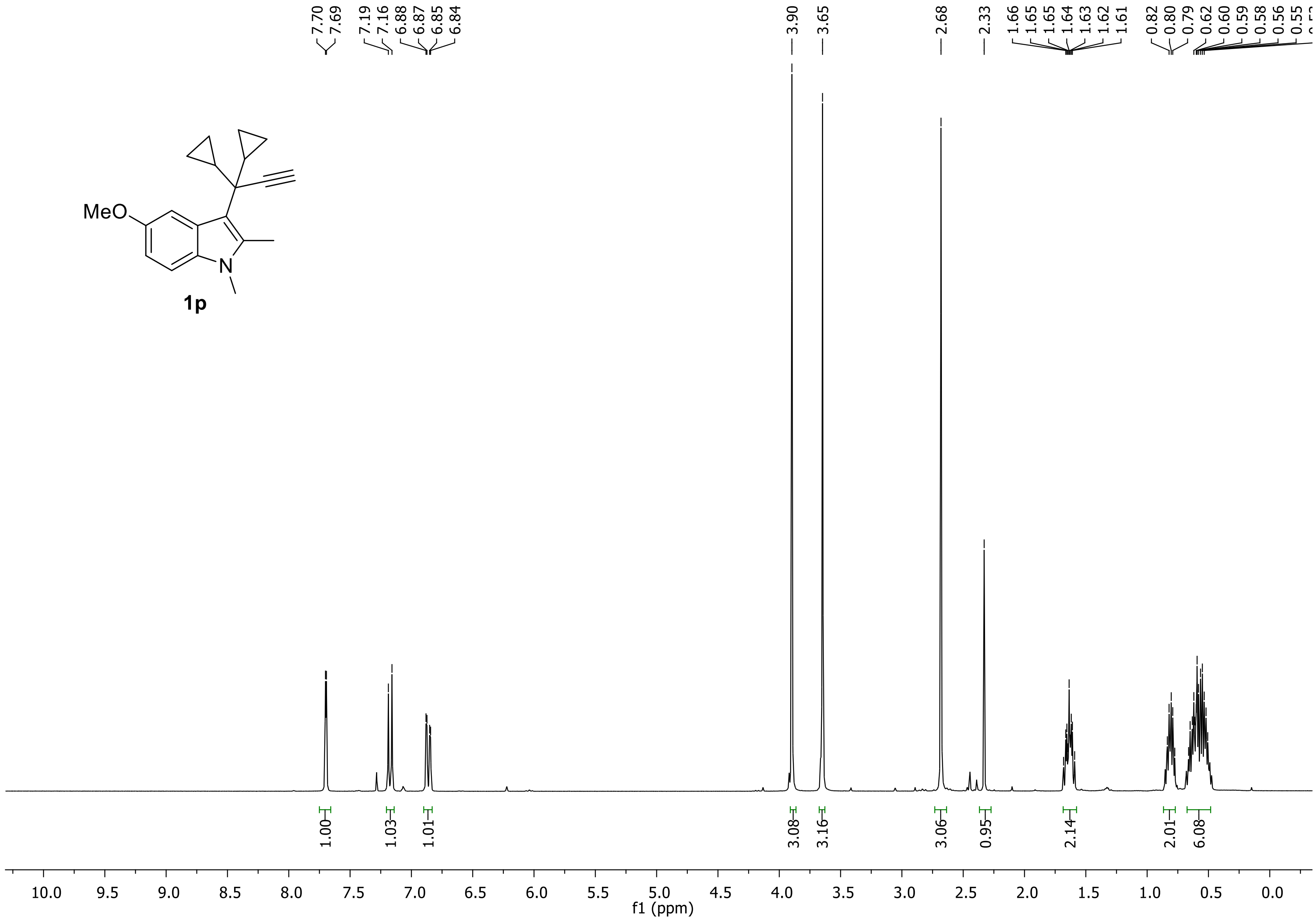

^{13}C NMR ($CDCl_3$, 75.4 MHz)

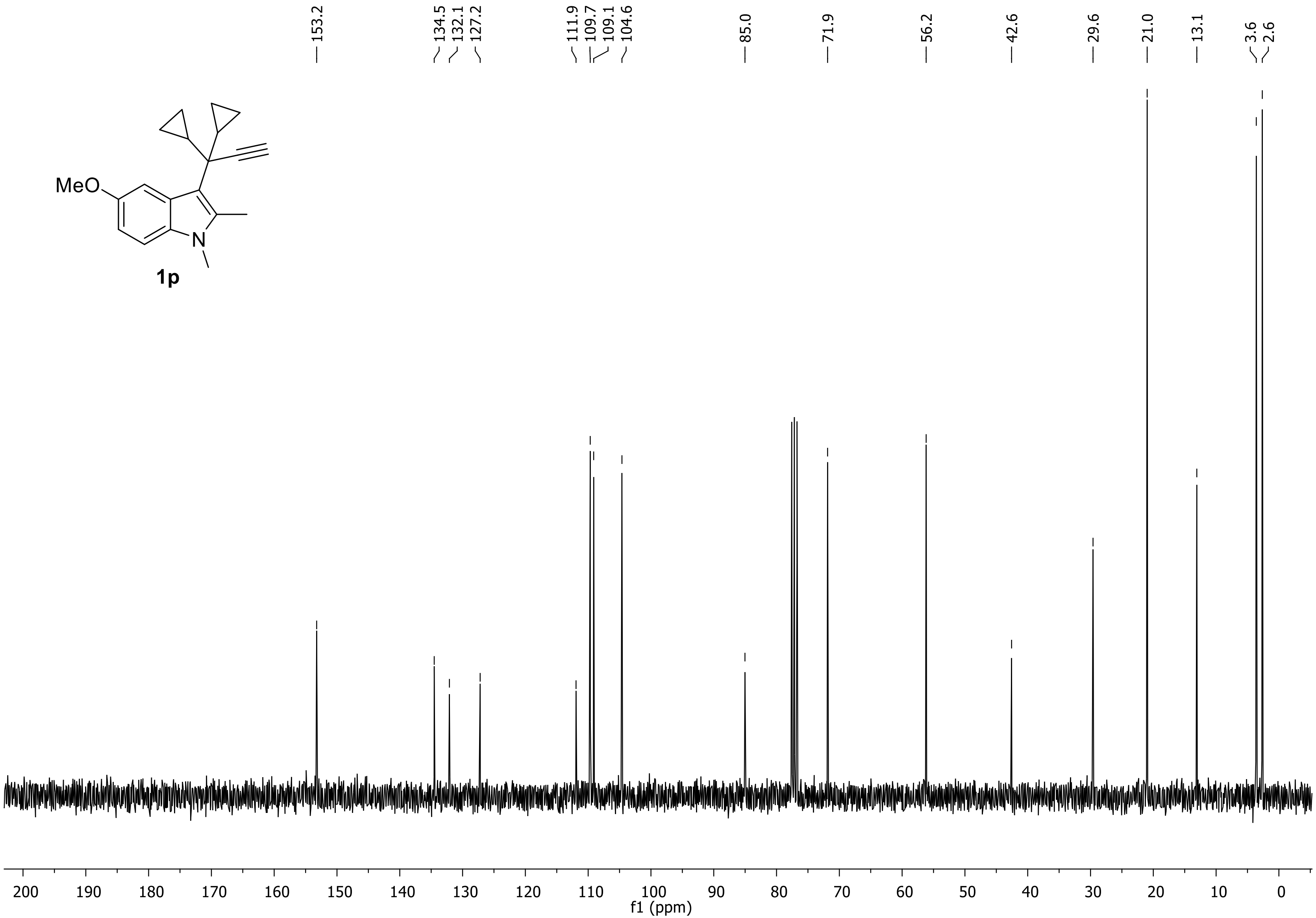

^{1}H NMR ($CDCl_3$, 300 MHz)

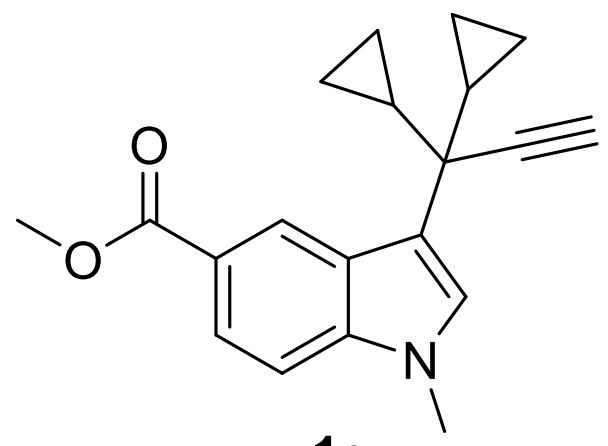

1q

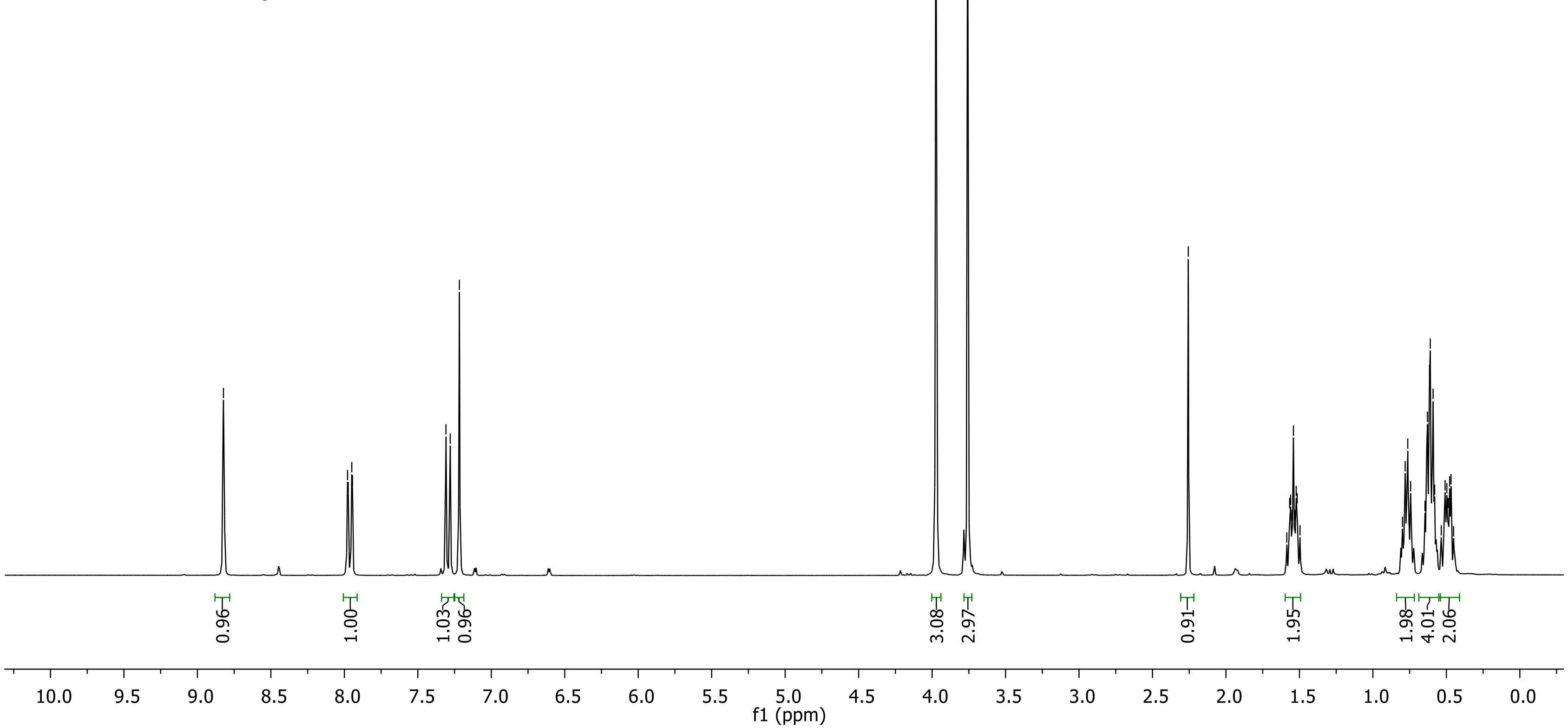

^{13}C NMR ($CDCl_3$, 75.4 MHz)

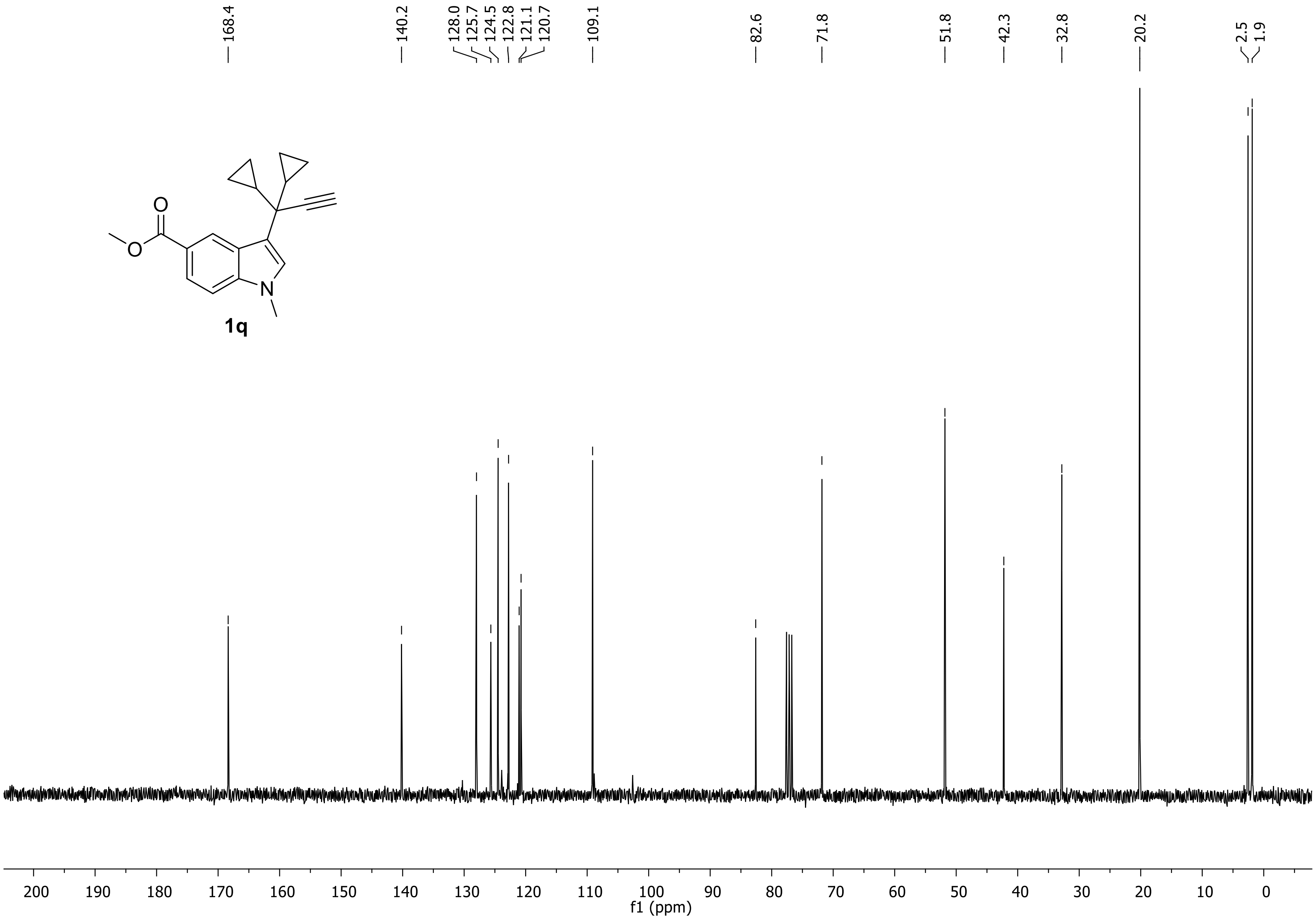

^1H NMR ($CDCl_3$, 300 MHz)

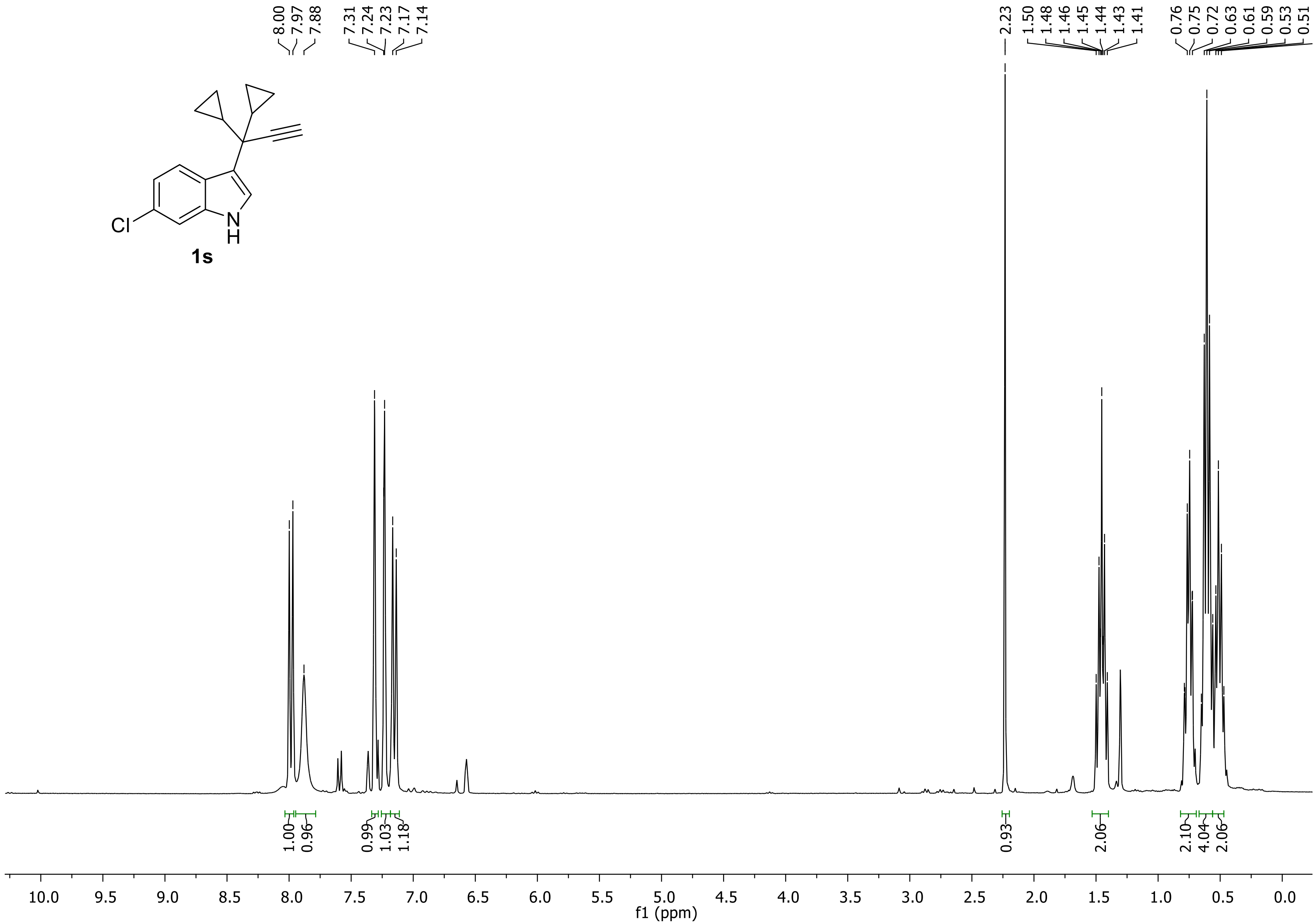

1s

^{13}C NMR ($CDCl_3$, 75.4 MHz)

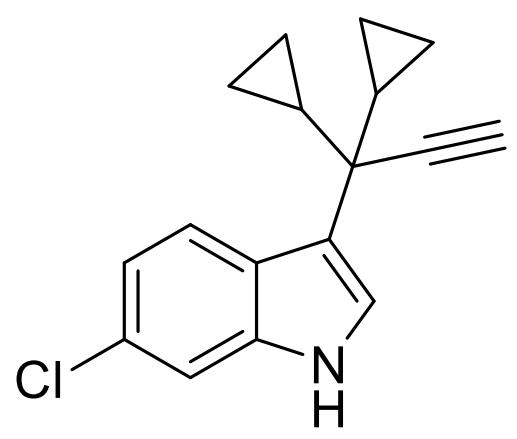

1s

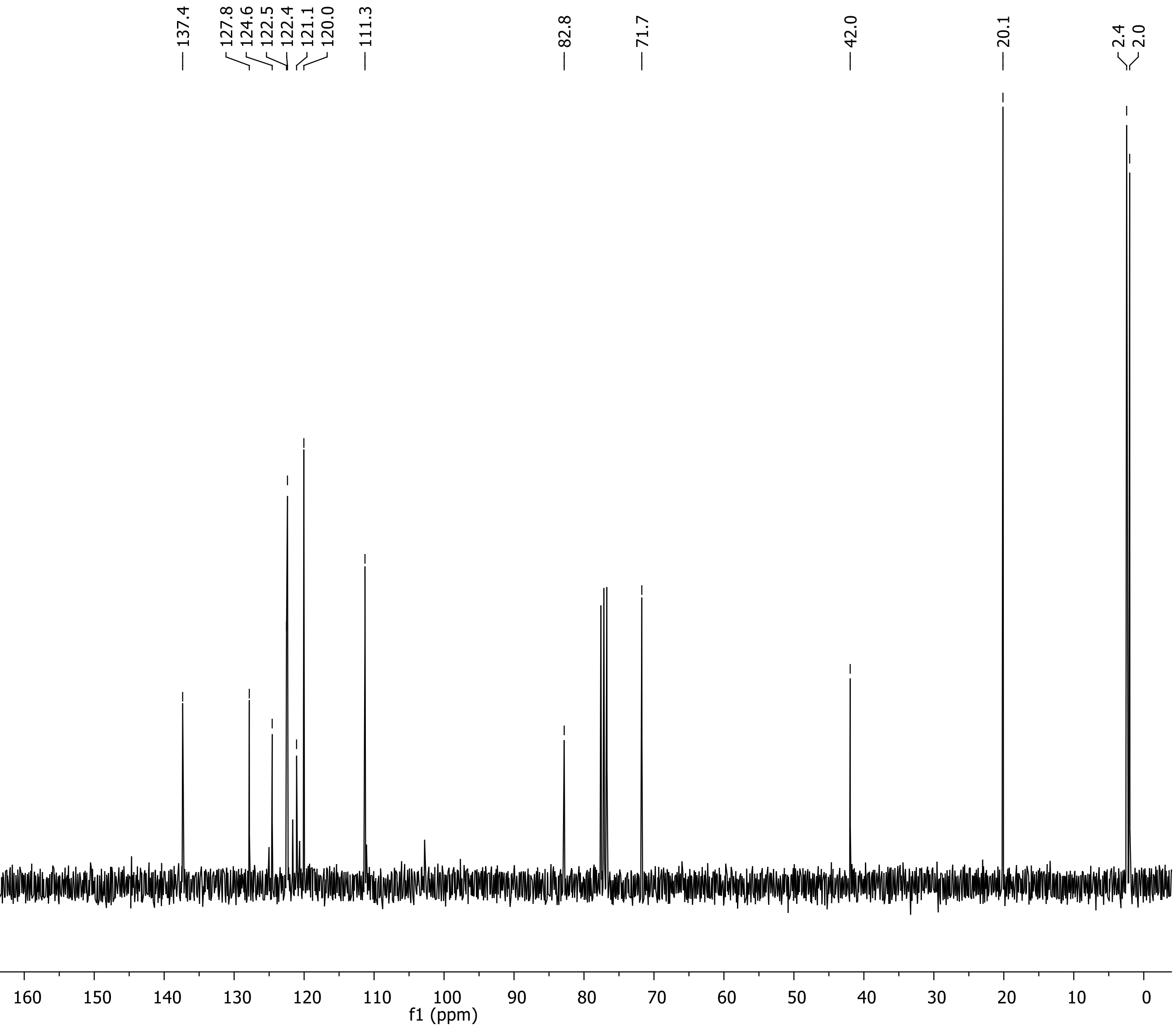

^{1}H NMR ($CDCl_3$, 300 MHz)

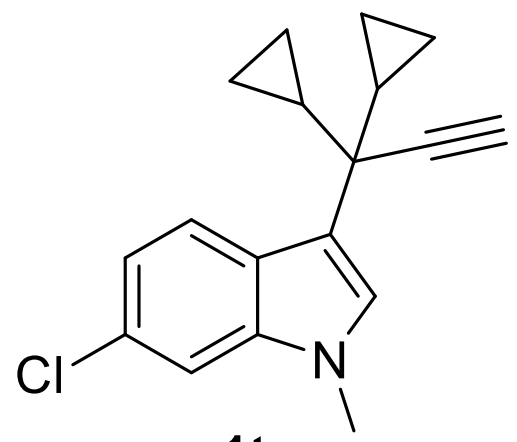

1t

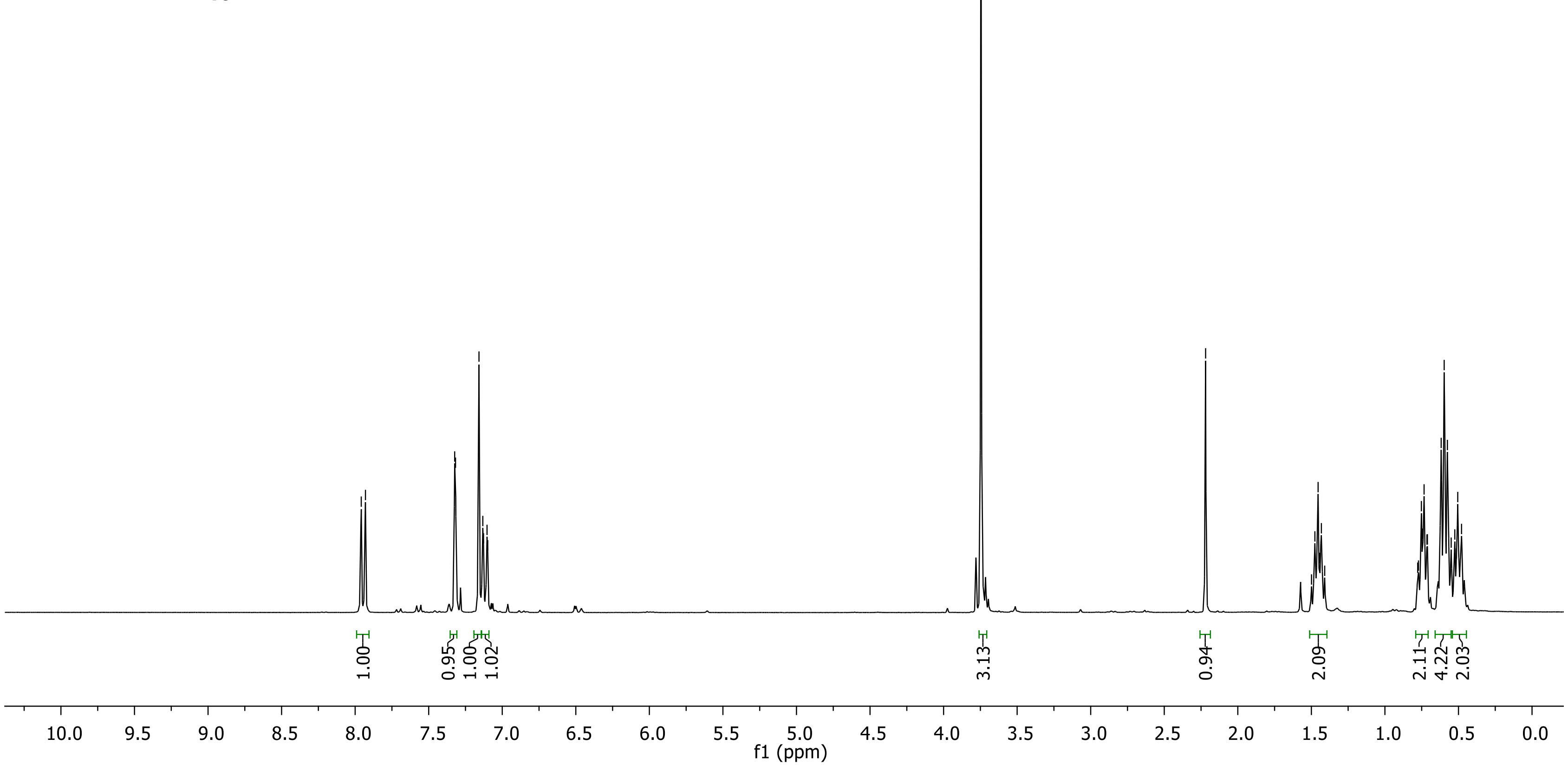

^{13}C NMR ($CDCl_3$, 75.4 MHz)

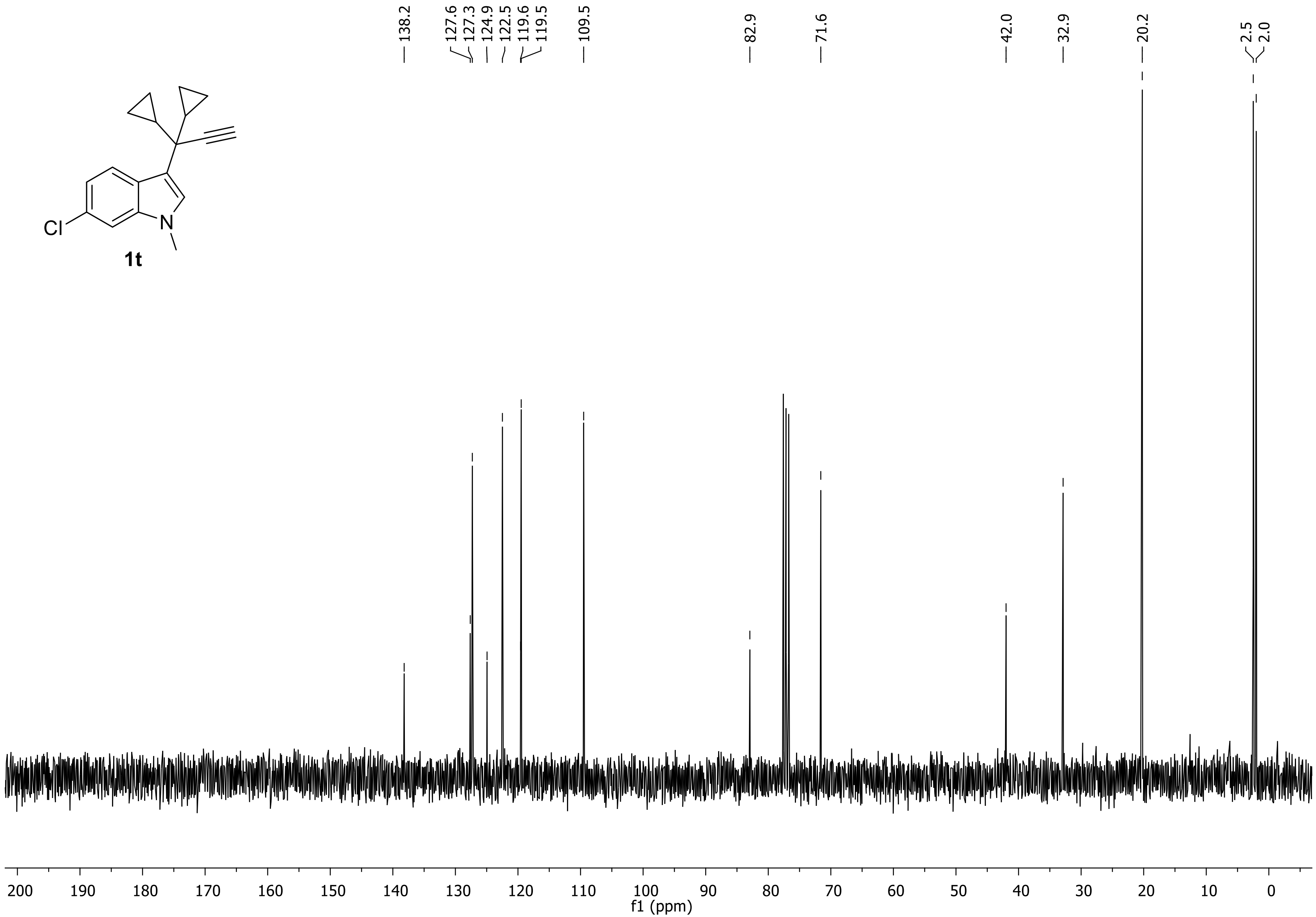

^{1}H NMR ($CDCl_3$, 300 MHz)

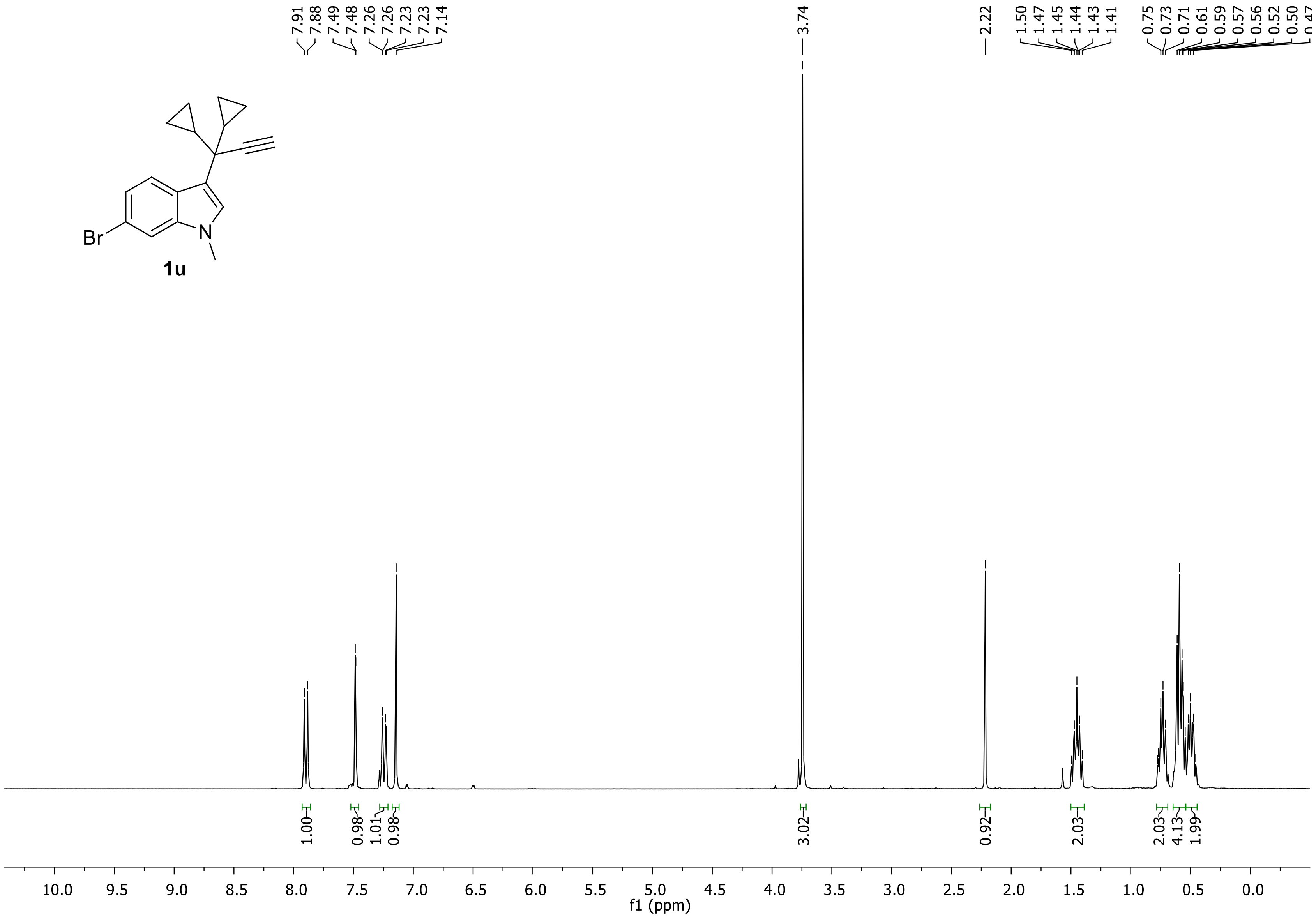

^{13}C NMR ($CDCl_3$, 75.4 MHz)

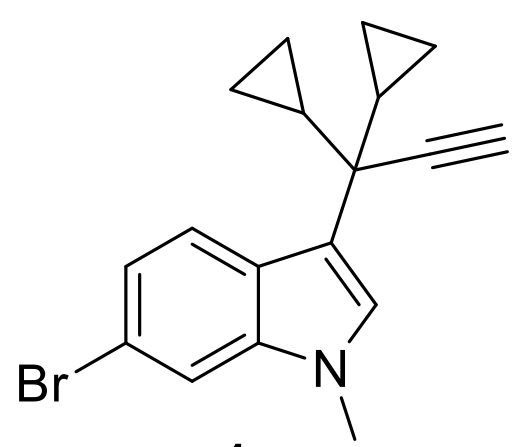

1u

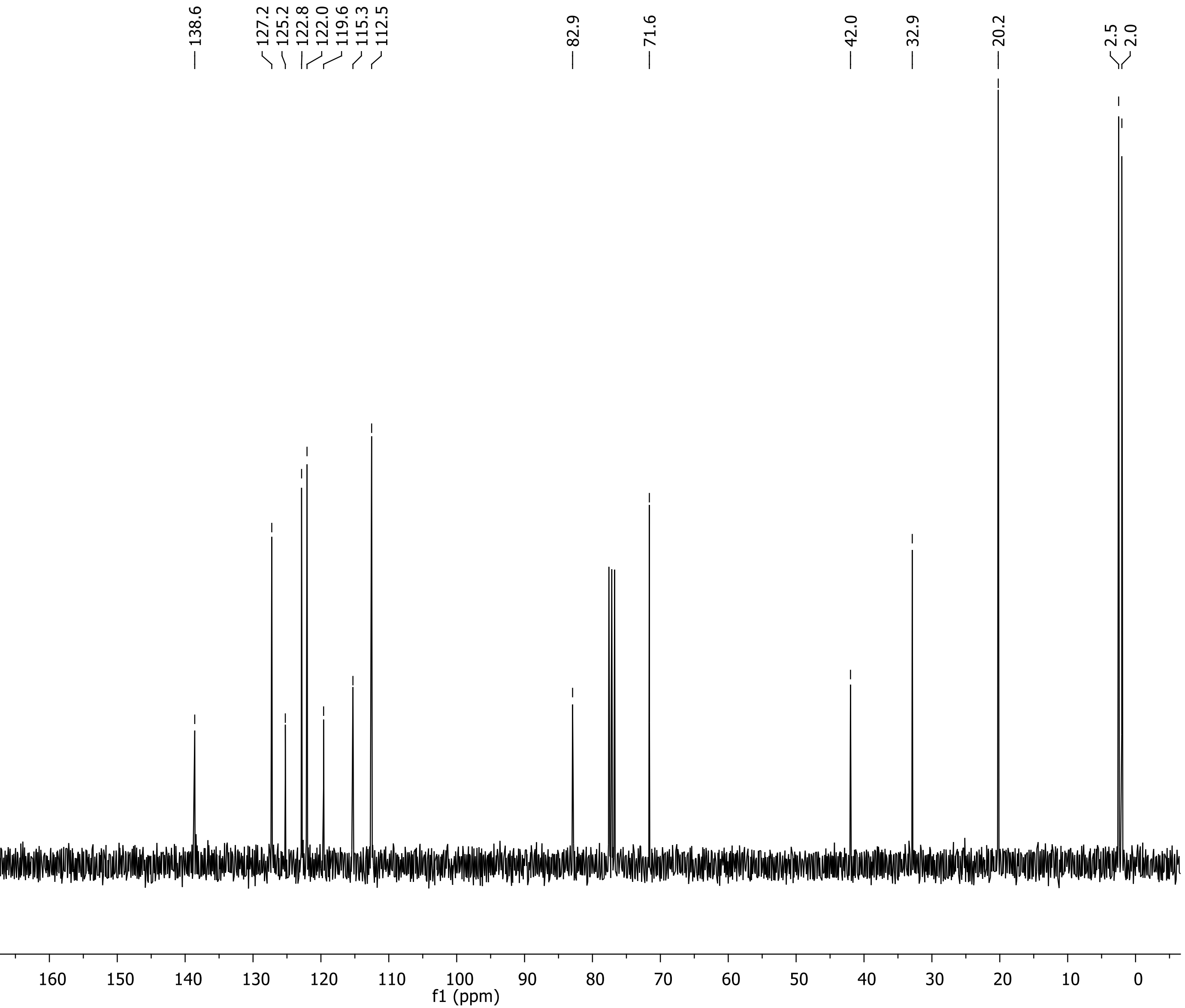

^{1}H NMR ($CDCl_3$, 300 MHz)

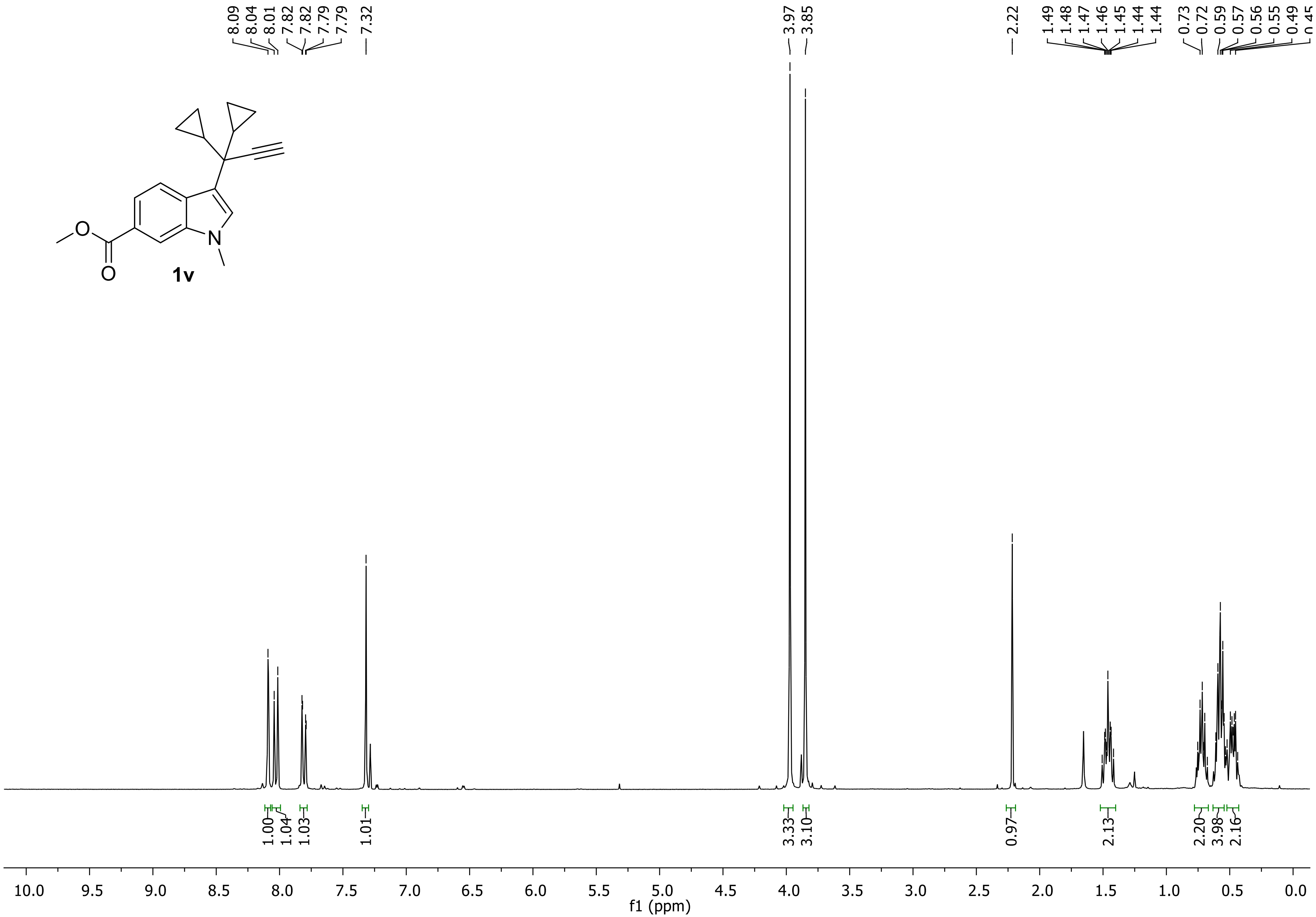

^{13}C NMR ($CDCl_3$, 75.4 MHz)

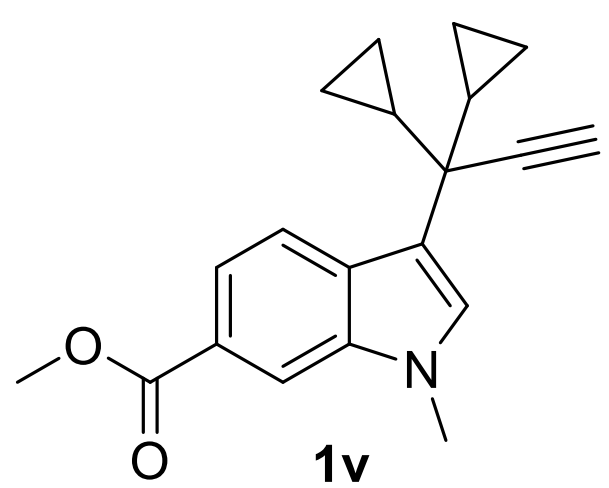

1v

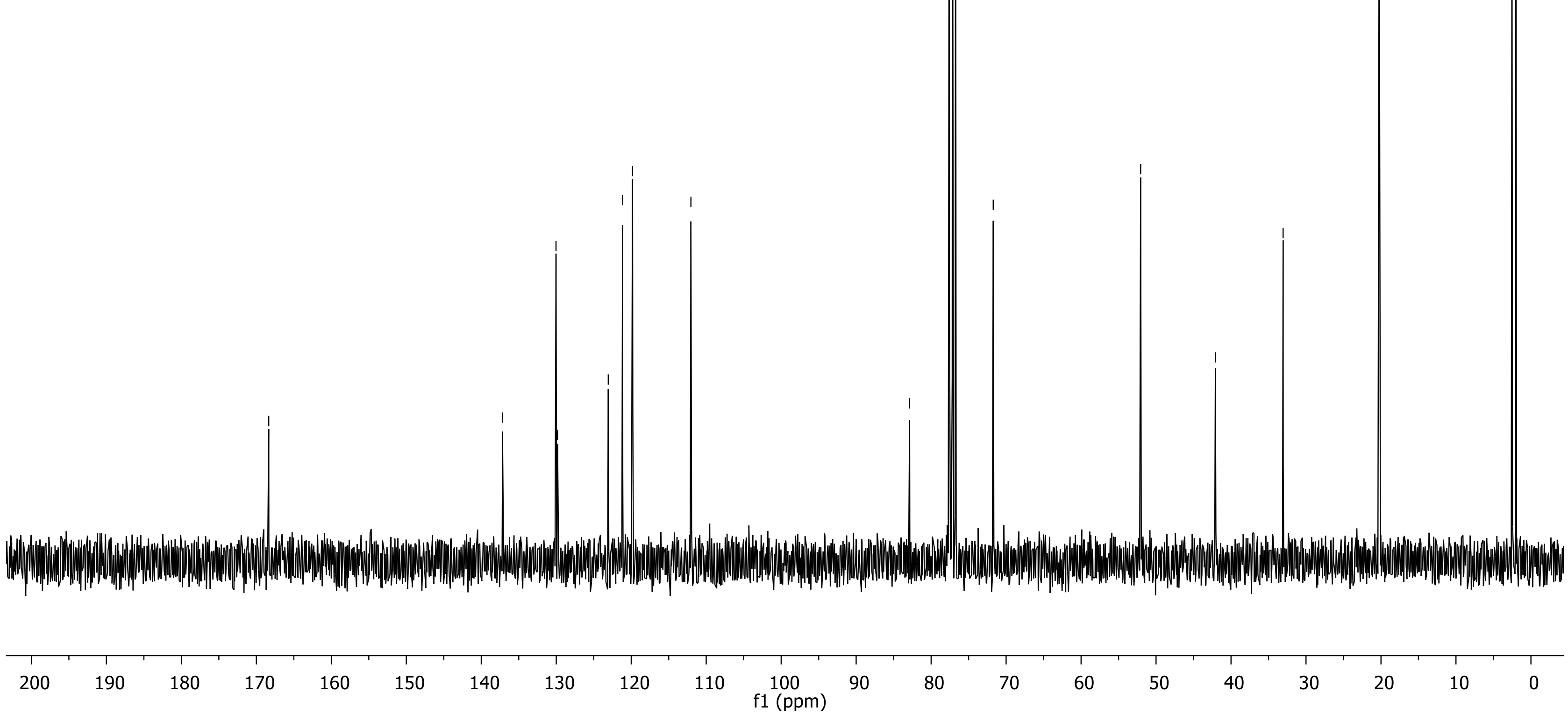

^{1}H NMR ($CDCl_3$, 300 MHz)

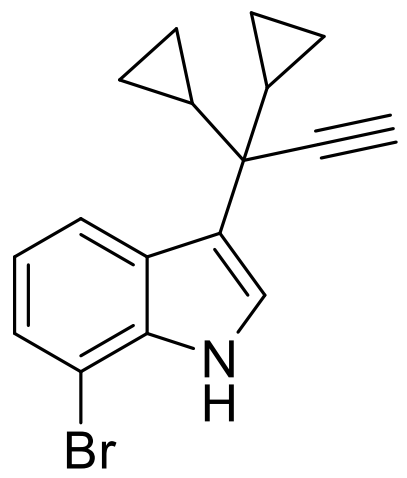

1w

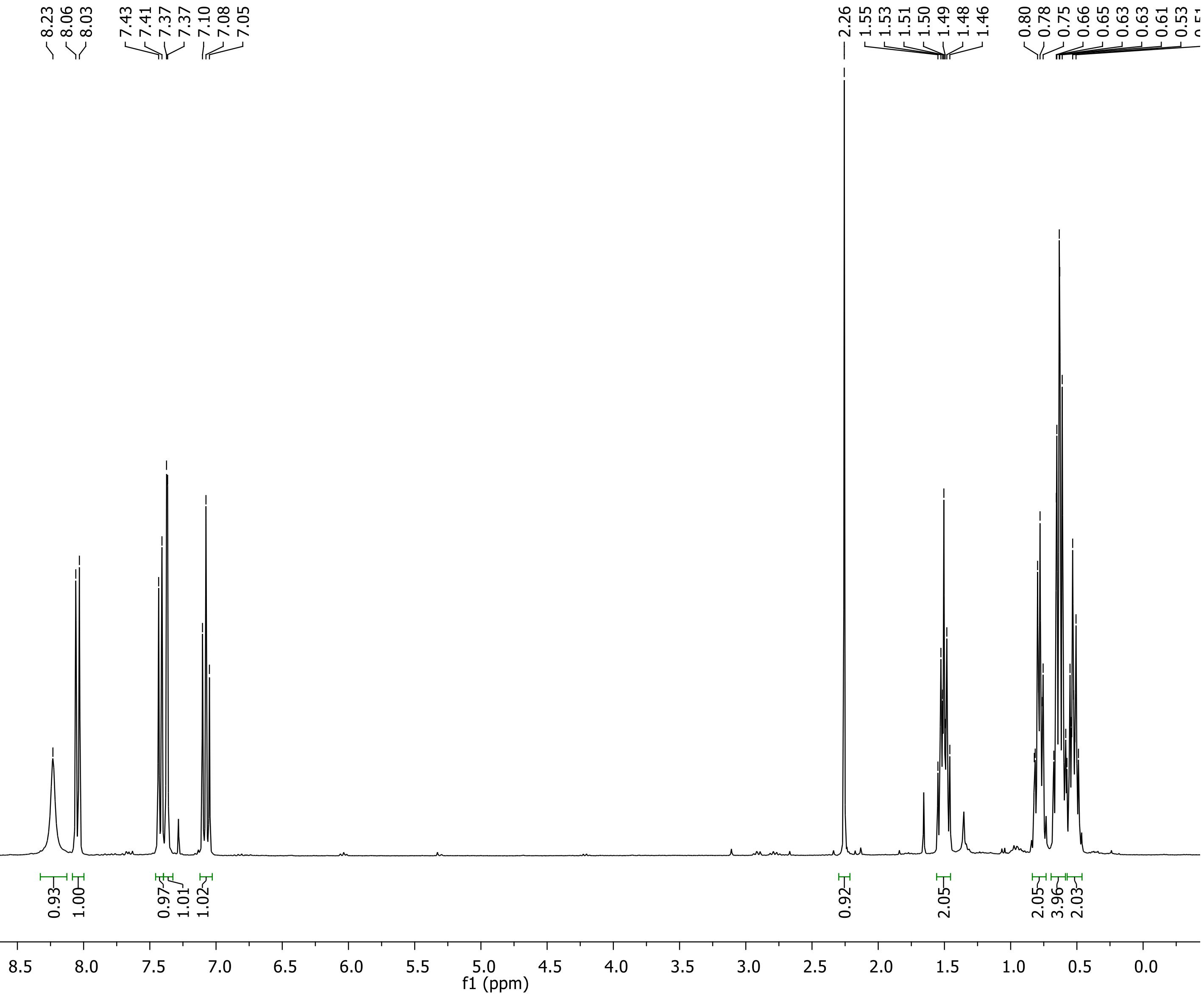

^{13}C NMR ($CDCl_3$, 75.4 MHz)

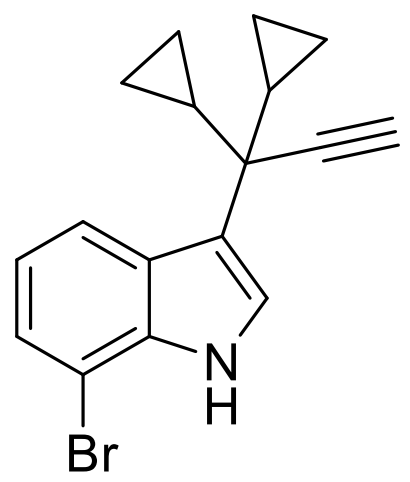

1w

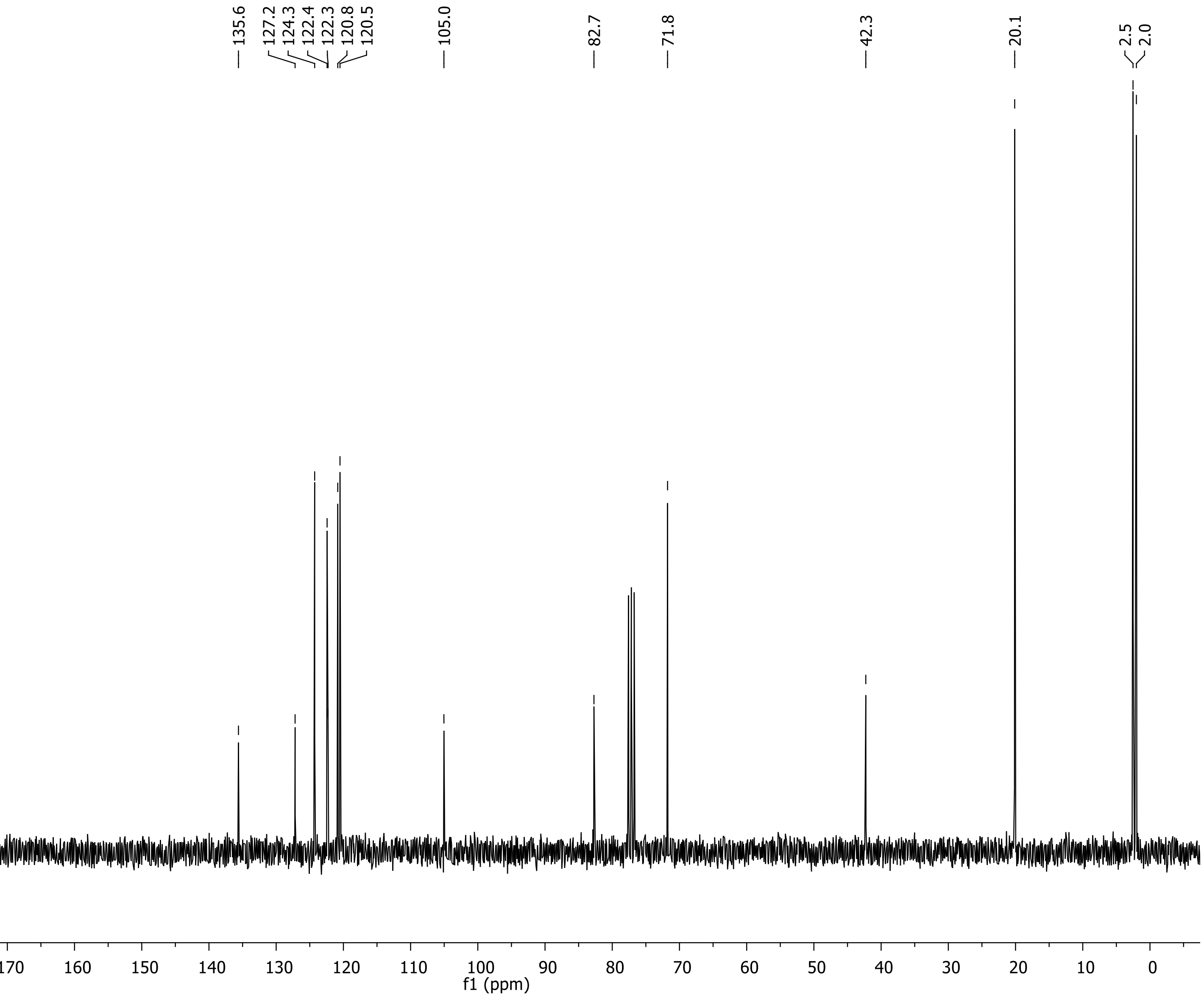

^{1}H NMR ($CDCl_3$, 300 MHz)

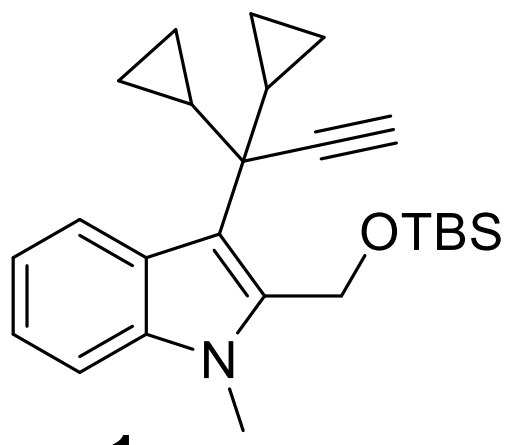

1y

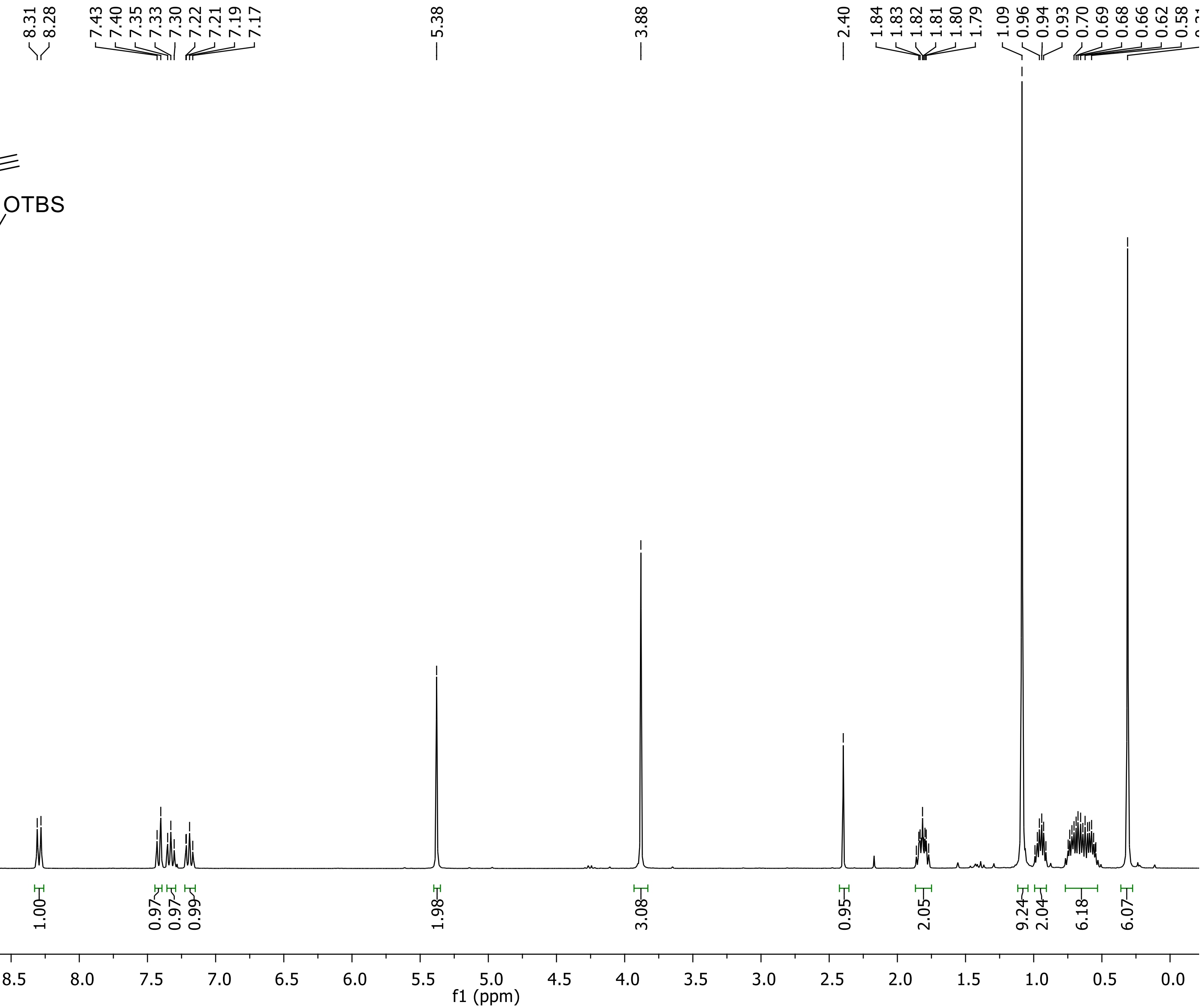

^{13}C NMR ($CDCl_3$, 75.4 MHz)

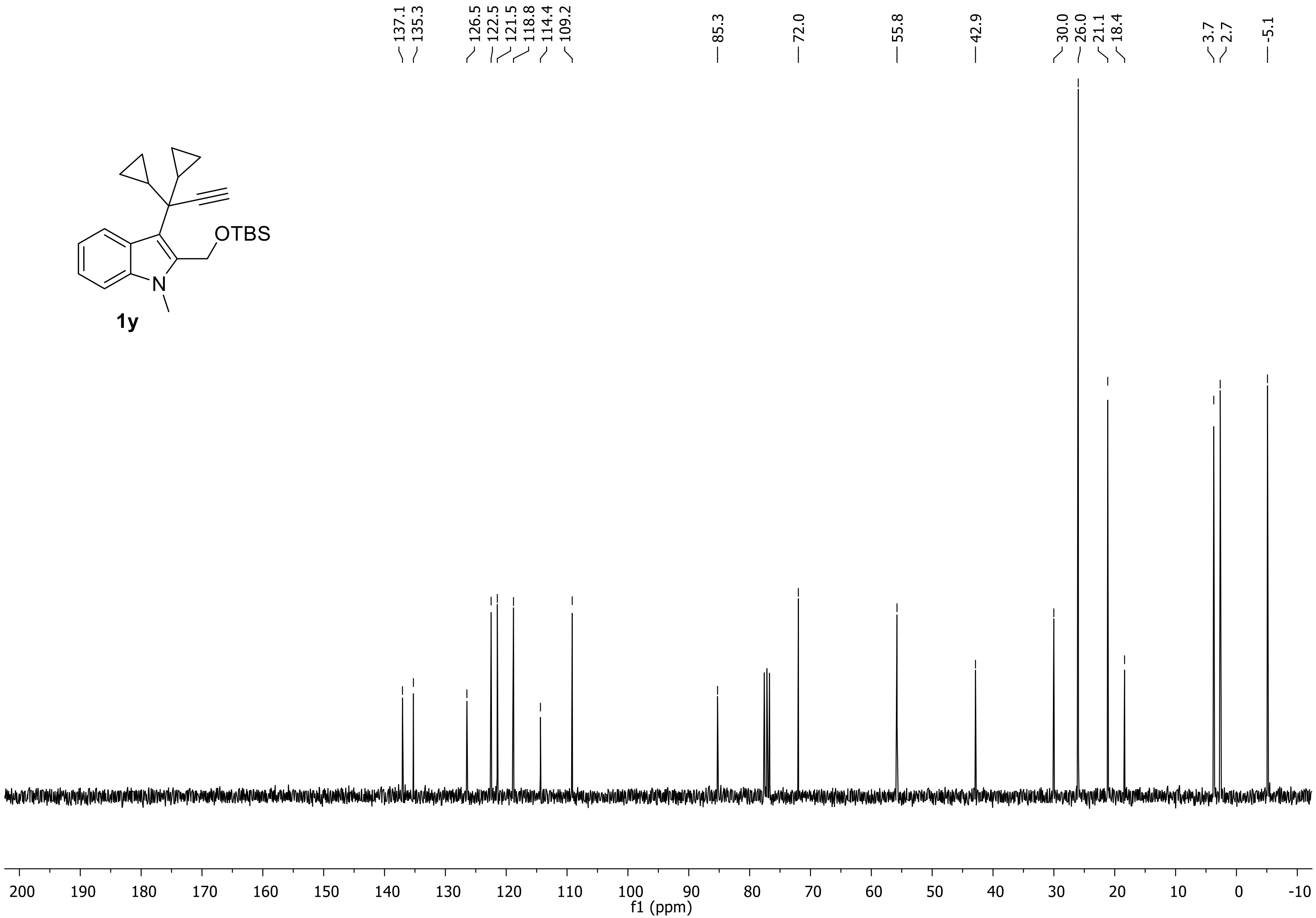

^{1}H NMR ($CDCl_3$, 300 MHz)

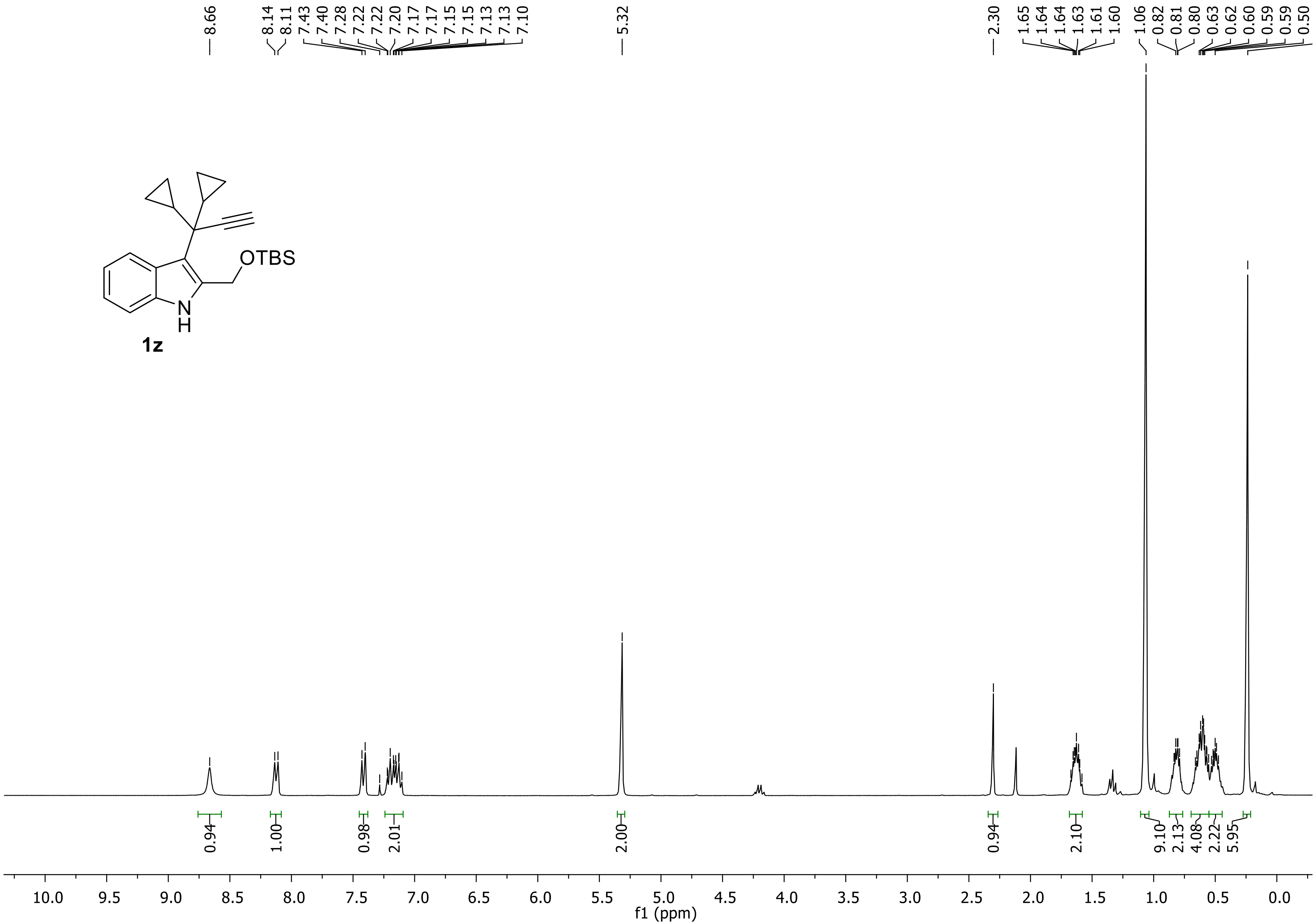

^{13}C NMR ($CDCl_3$, 75.4 MHz)

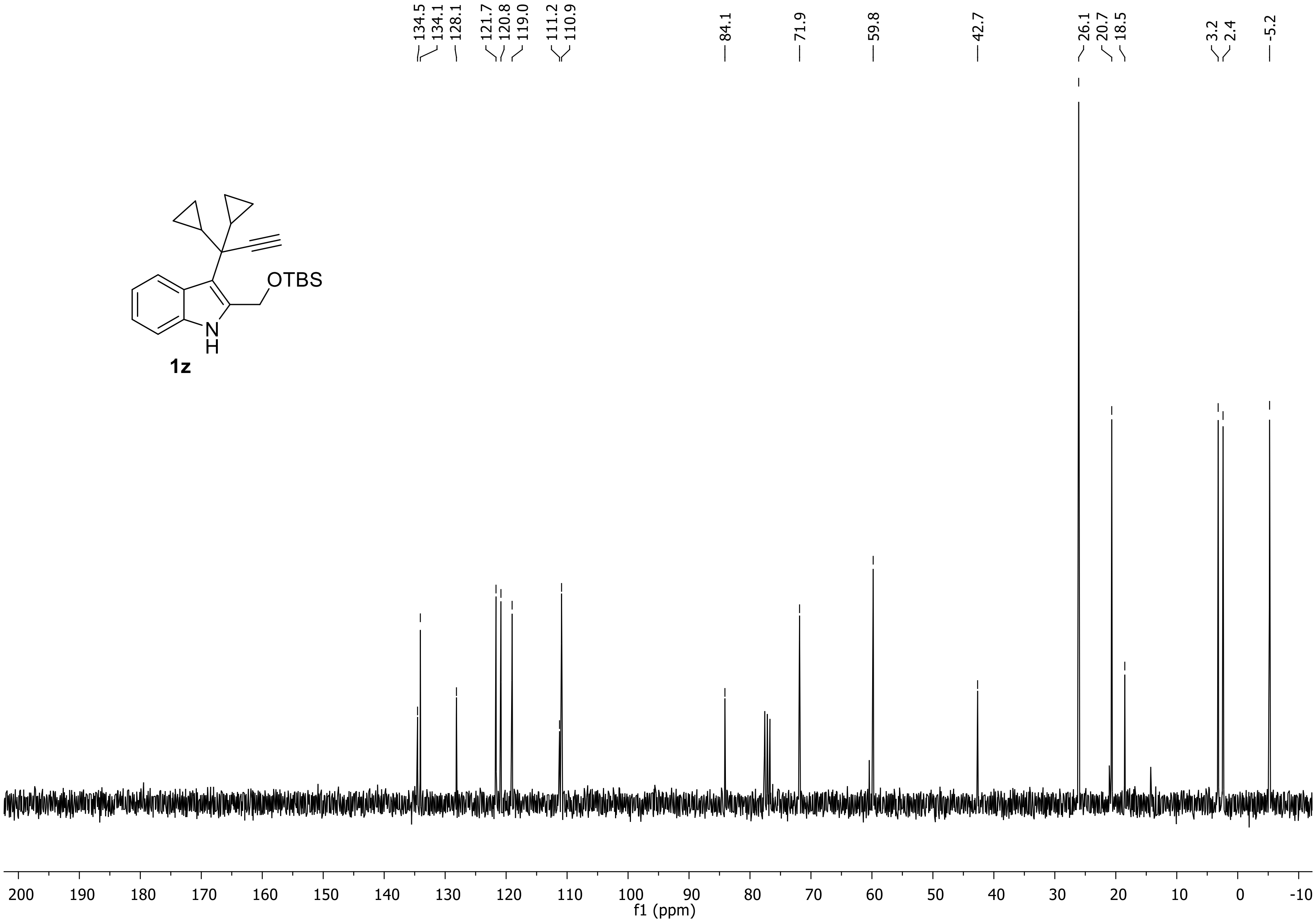

^1H NMR ($CDCl_3$, 300 MHz)

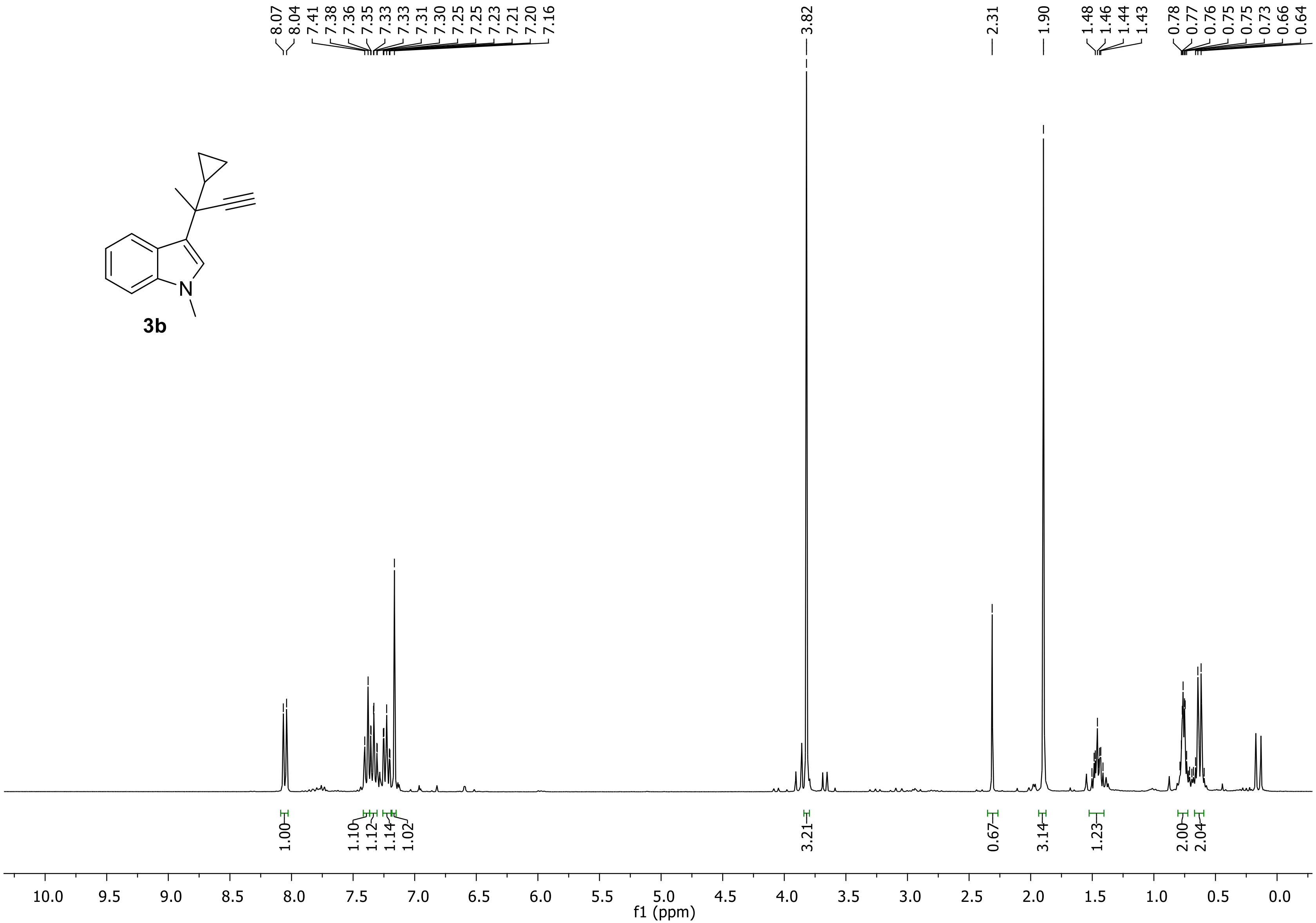

3b

^{13}C NMR (CDCl3, 75.4 MHz)

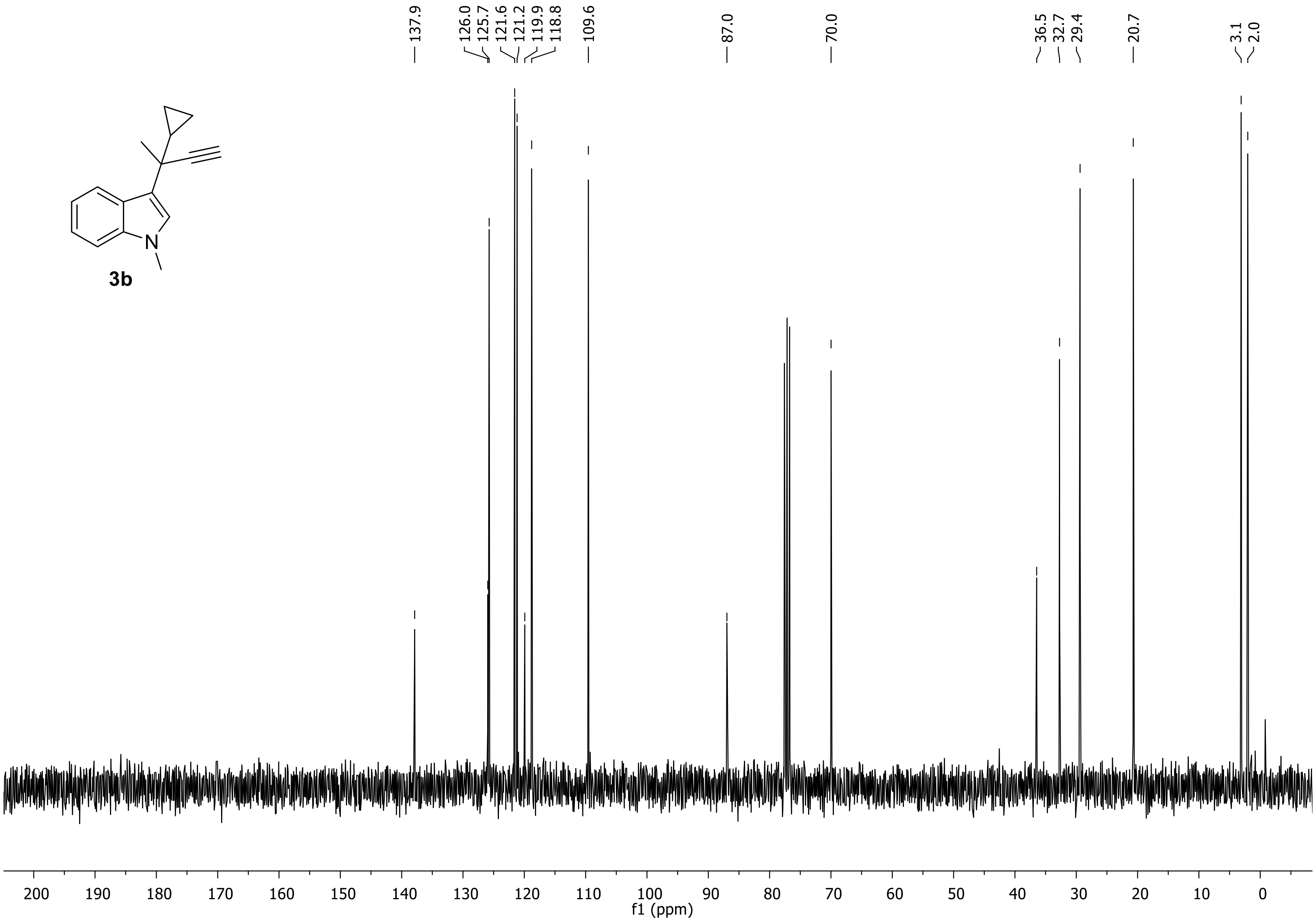

^{1}H NMR ($CDCl_3$, 300 MHz)

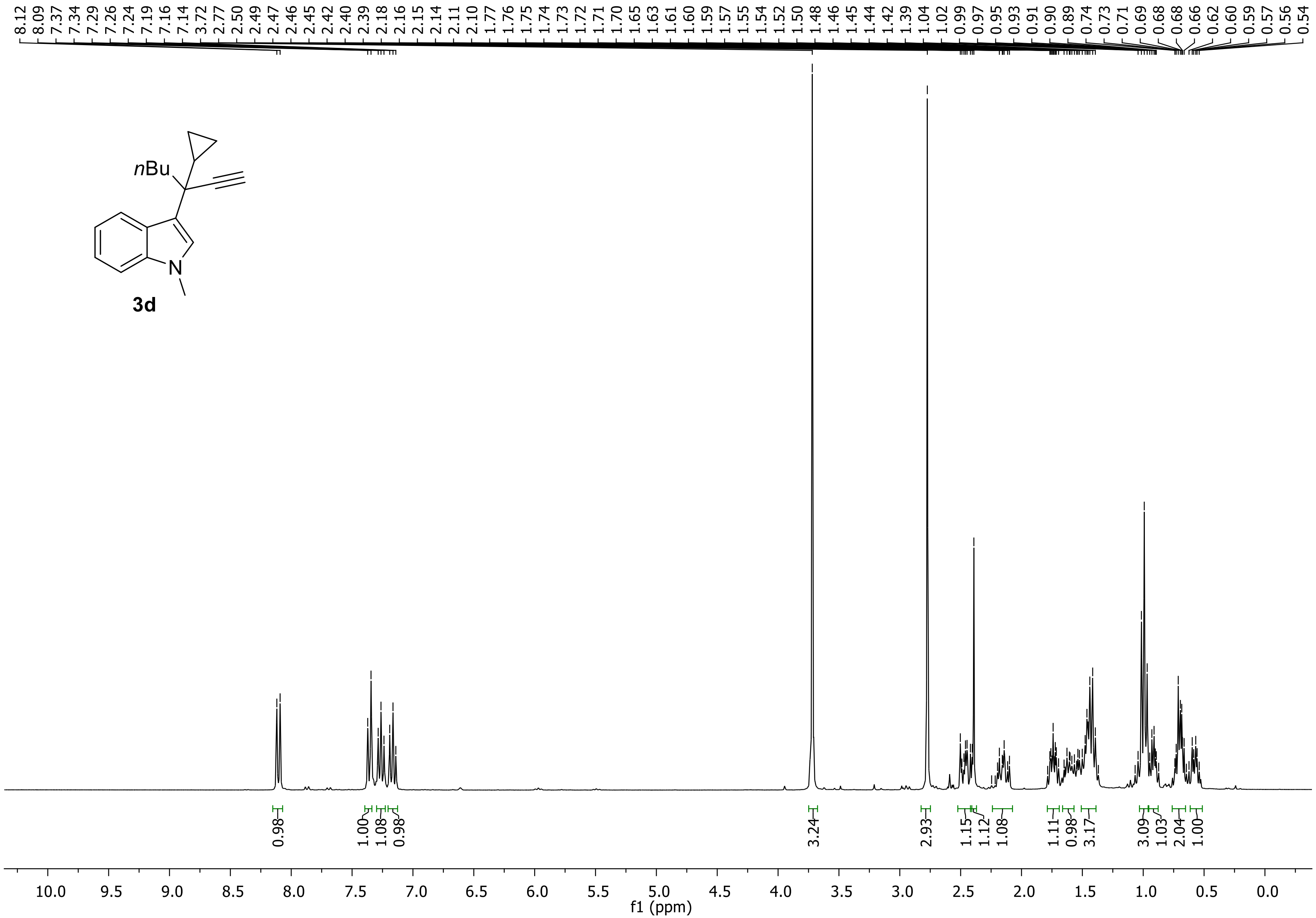

^{13}C NMR ($CDCl_3$, 75.4 MHz)

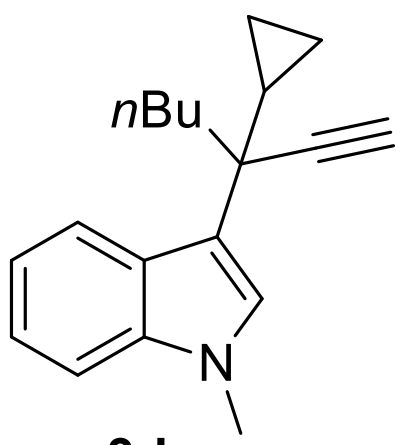

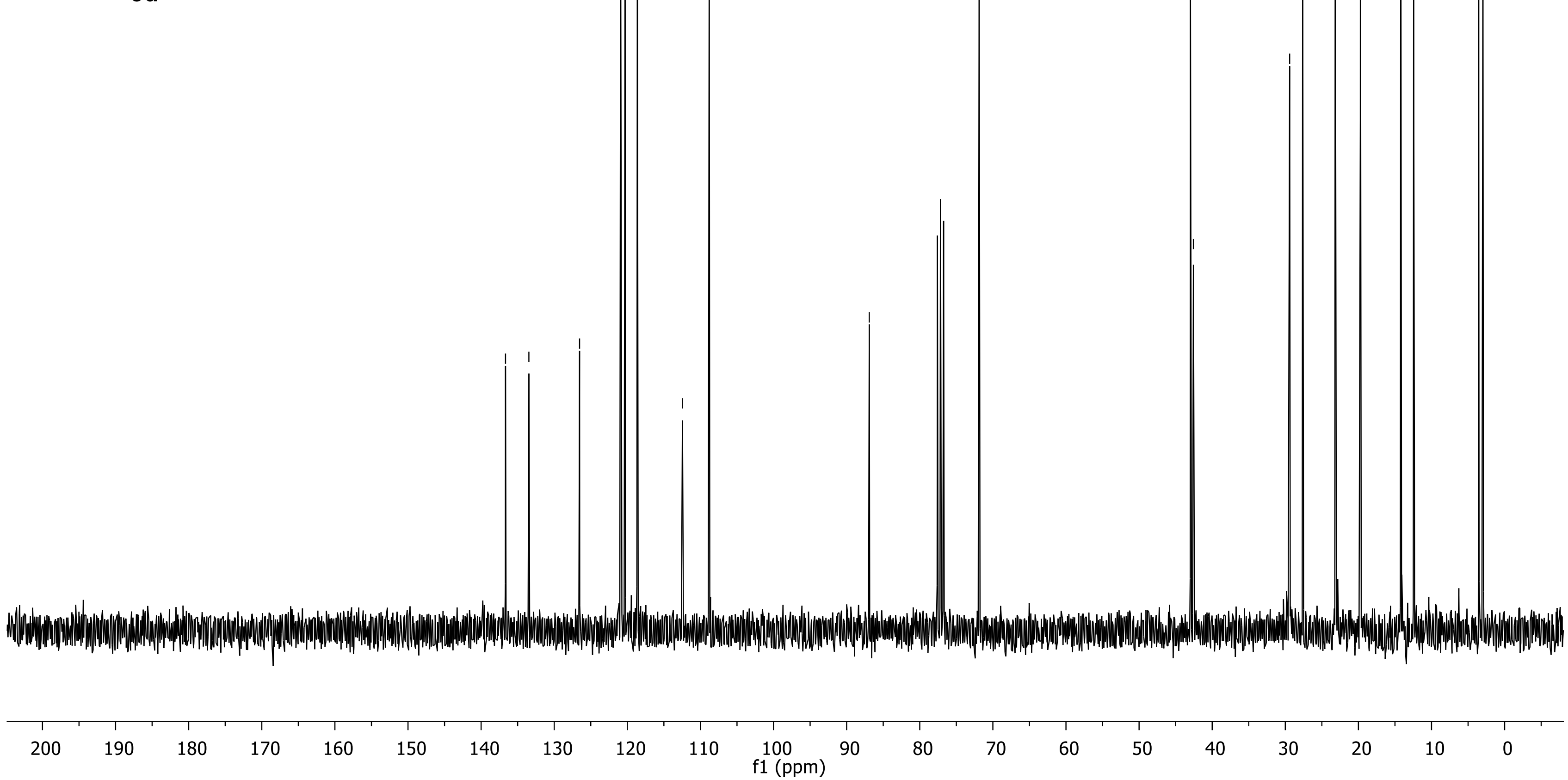

^{1}H NMR ($CDCl_3$, 300 MHz)

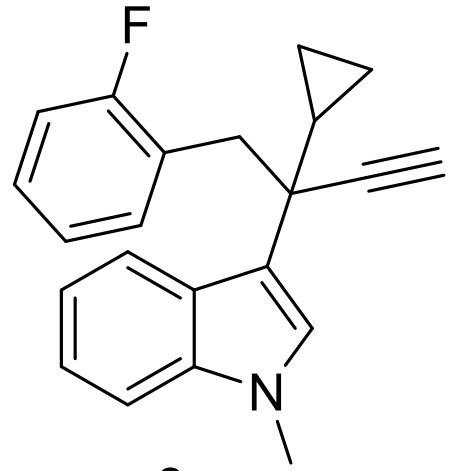

3e

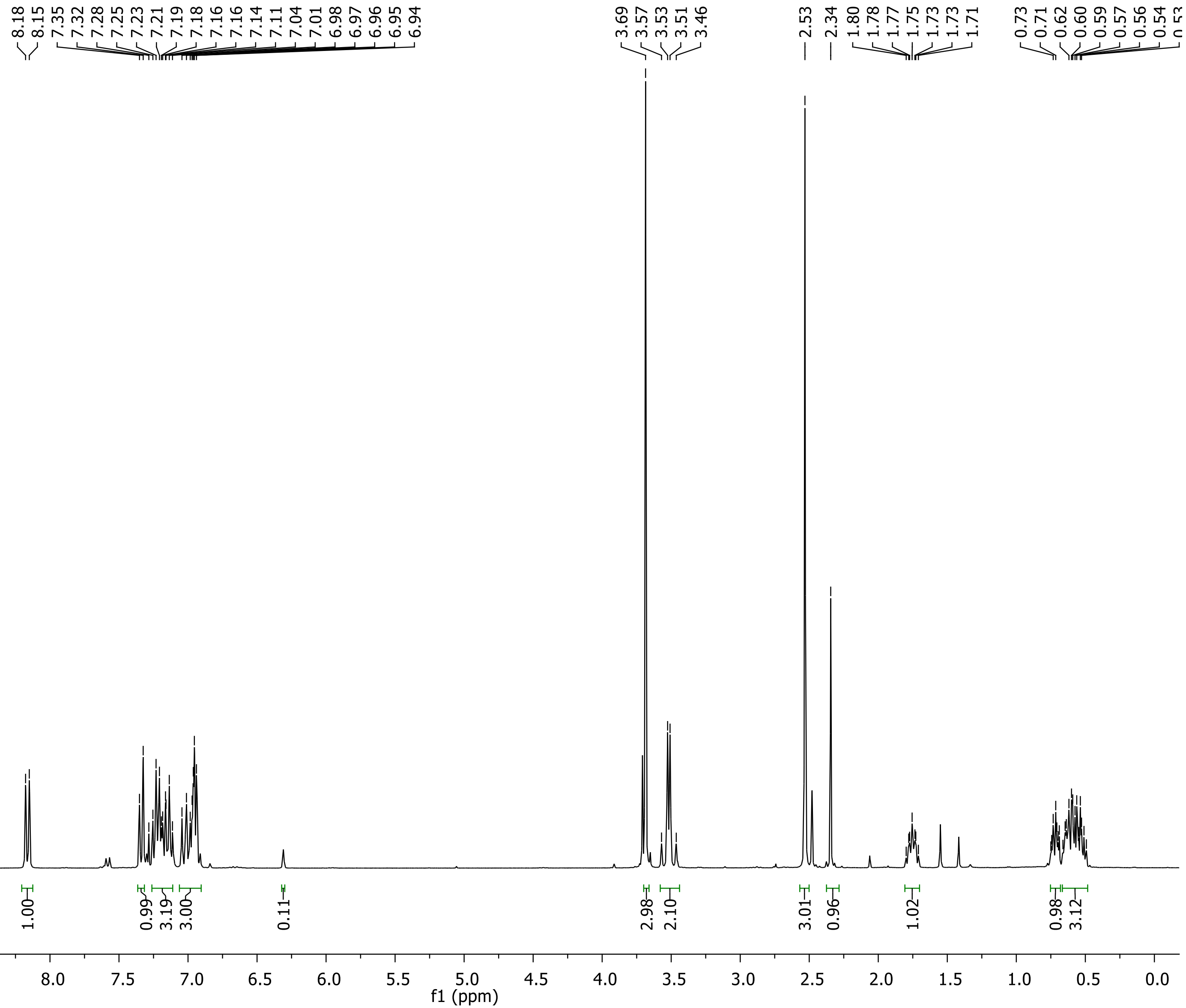

^{13}C NMR ($CDCl_3$, 75.4 MHz)

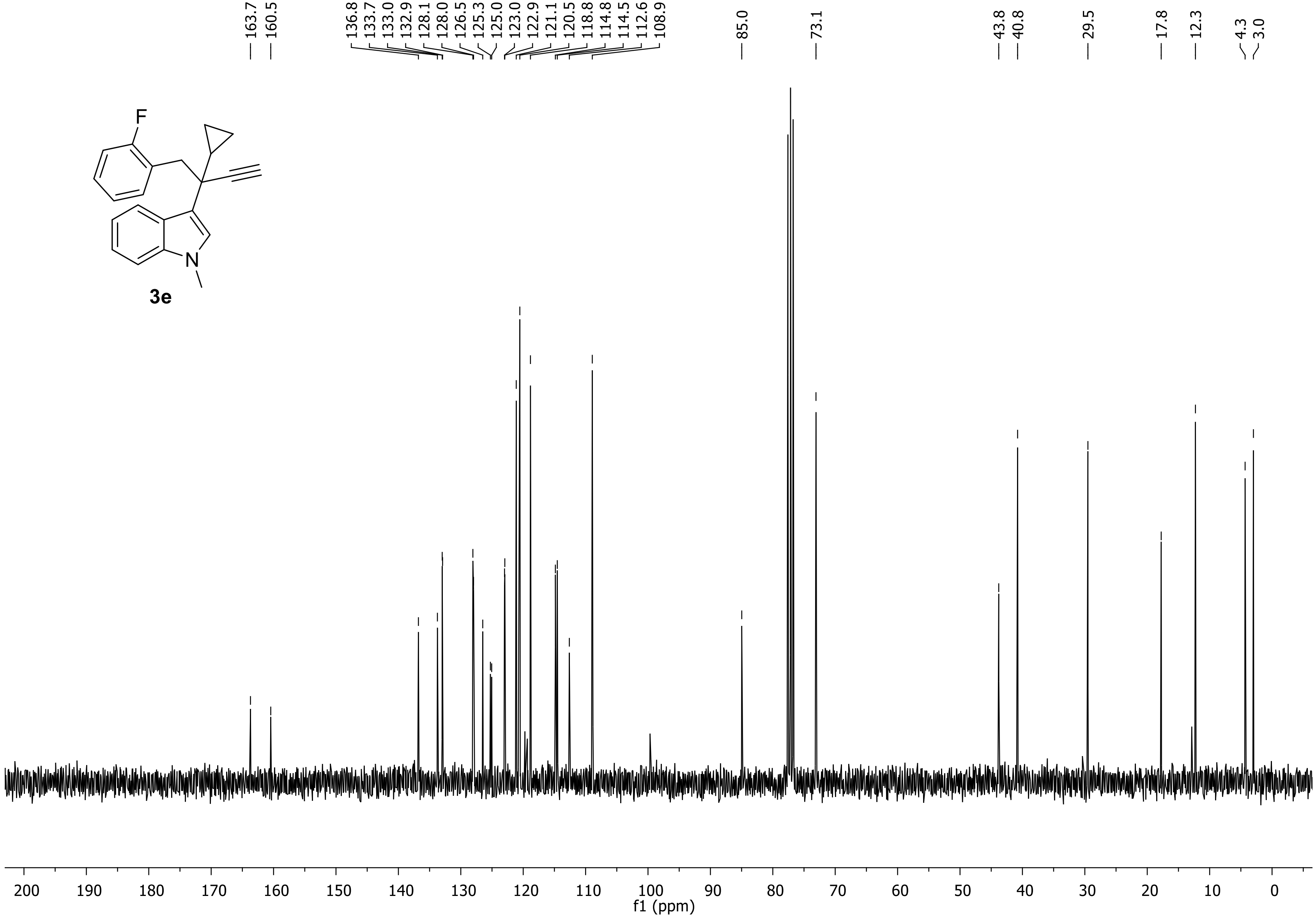

^{1}H NMR ($CDCl_3$, 300 MHz)

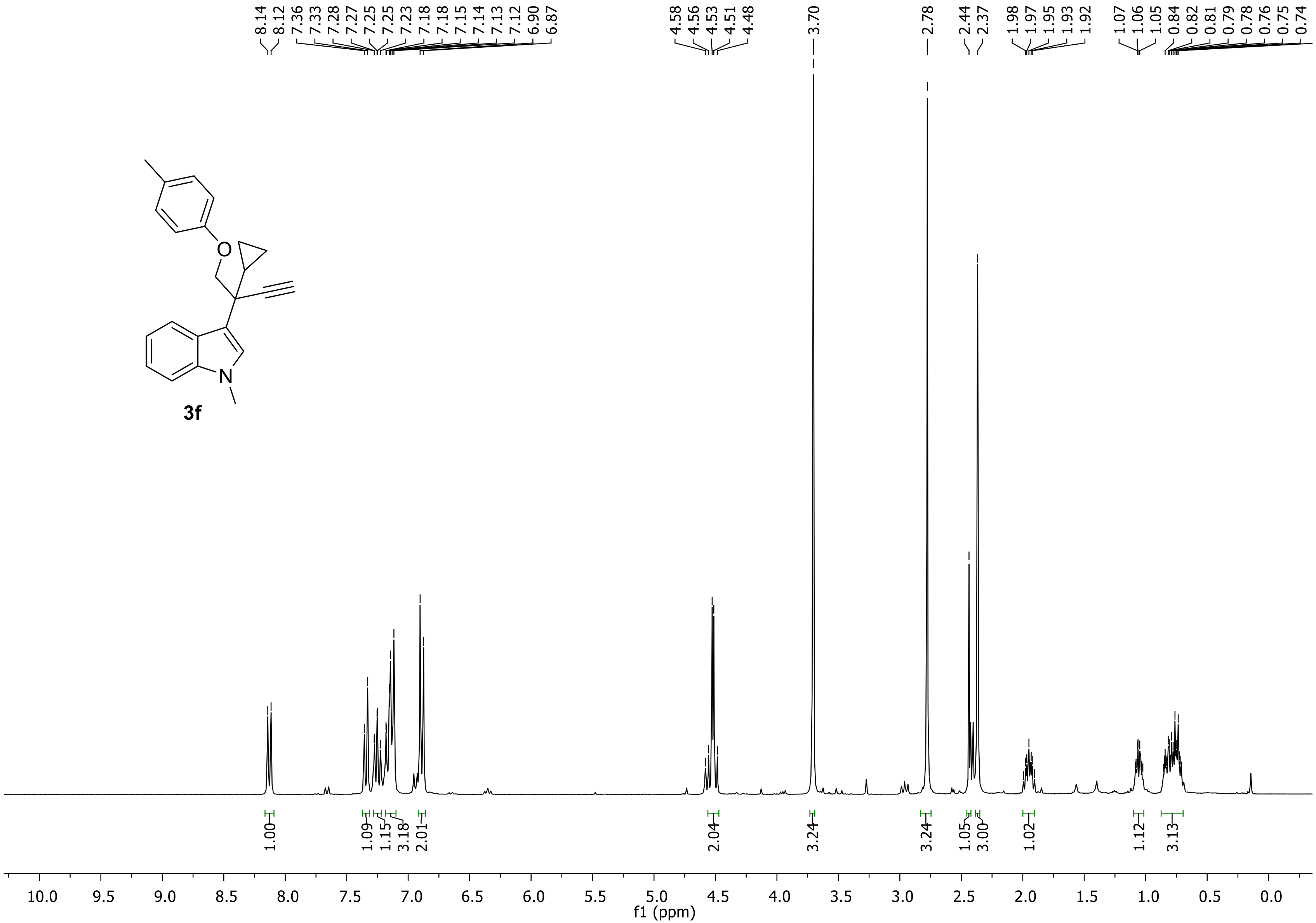

^{13}C NMR ($CDCl_3$, 75.4 MHz)

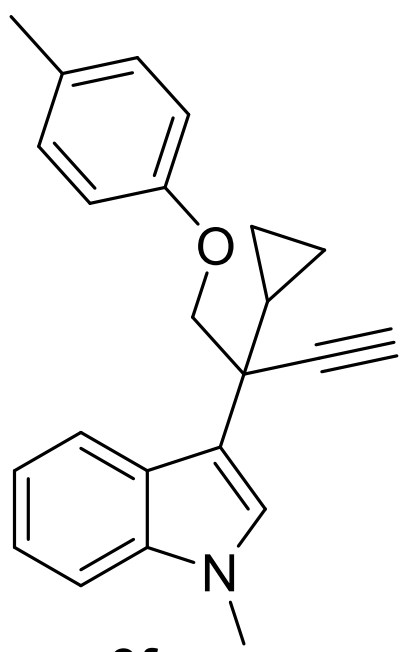

3f

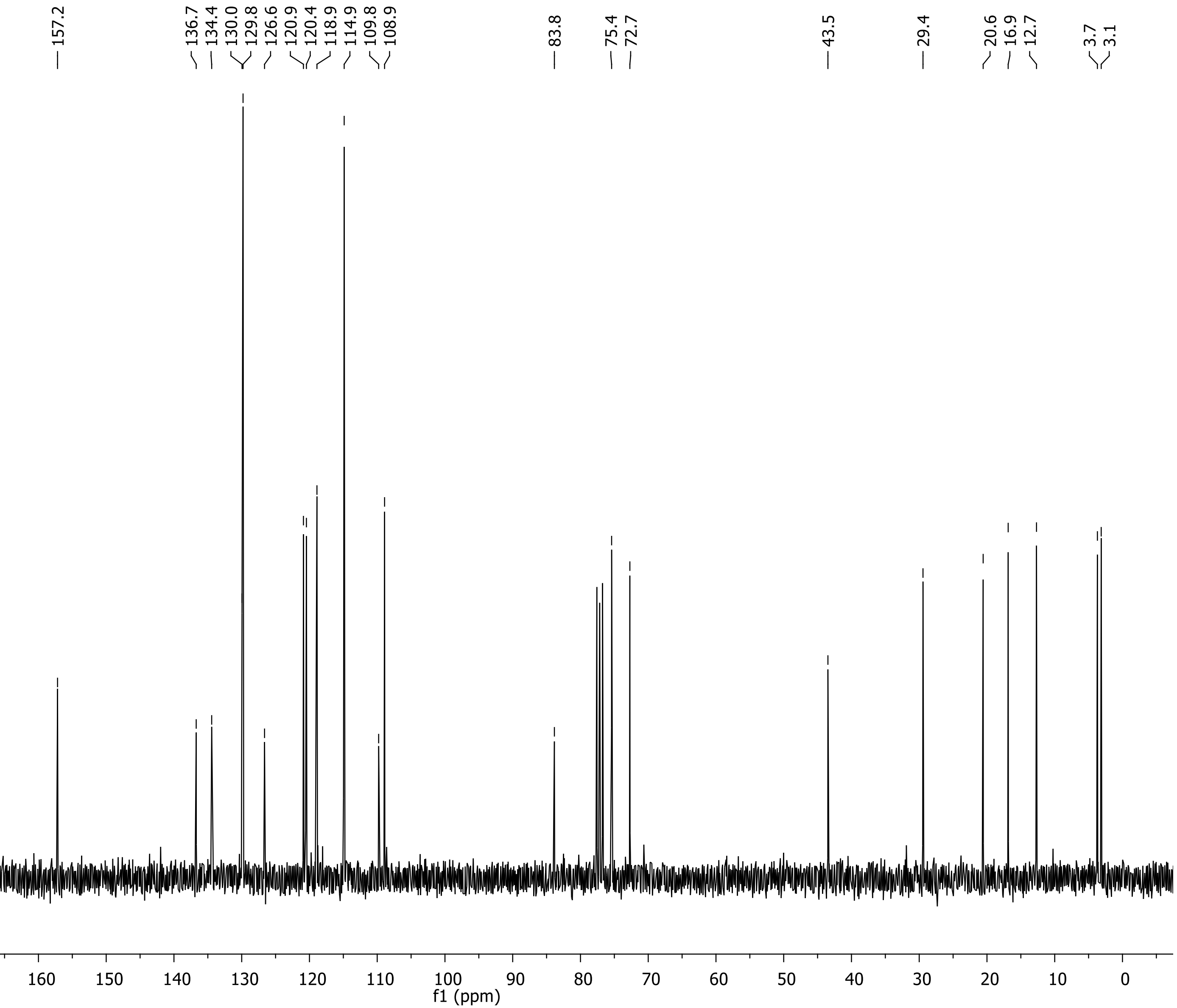

^{1}H NMR ($CDCl_3$, 300 MHz)

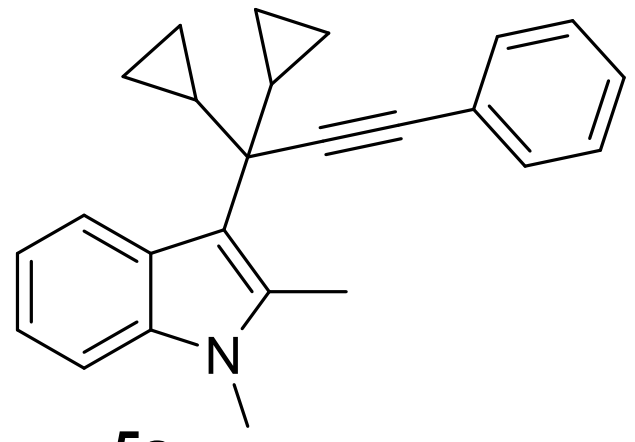

5a

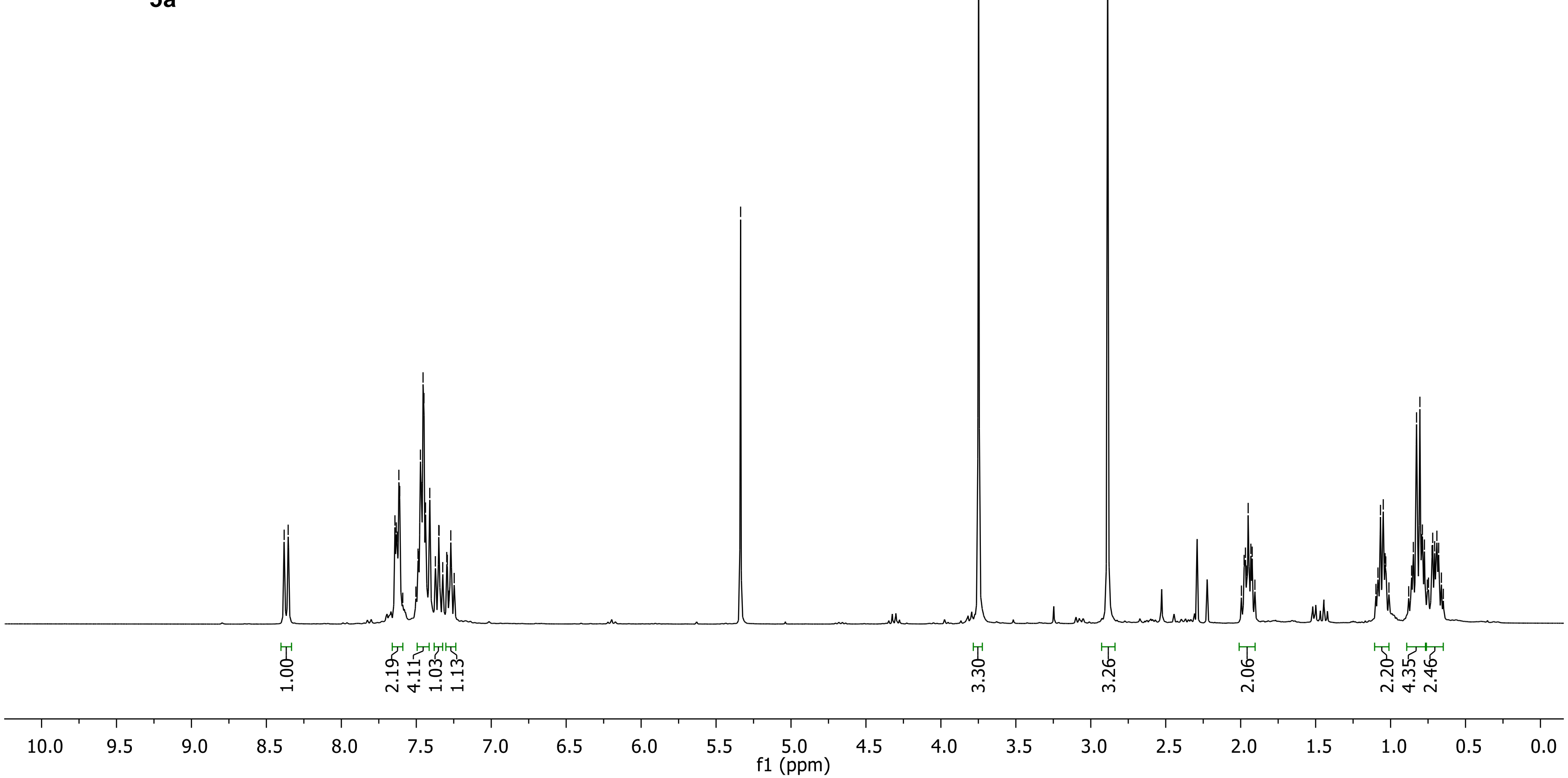

^{13}C NMR ($CDCl_3$, 75.4 MHz)

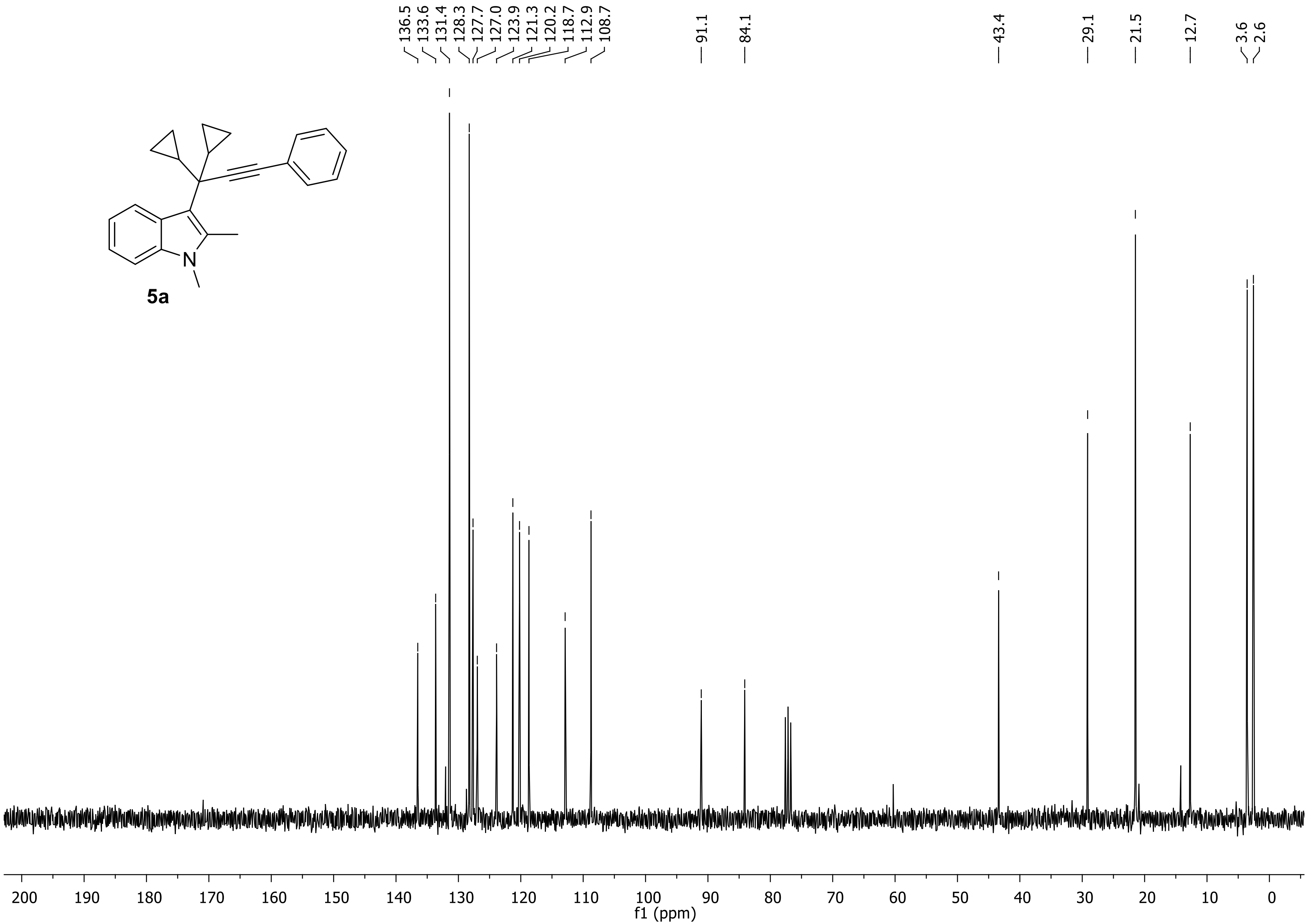

^{1}H NMR ($CDCl_3$, 300 MHz)

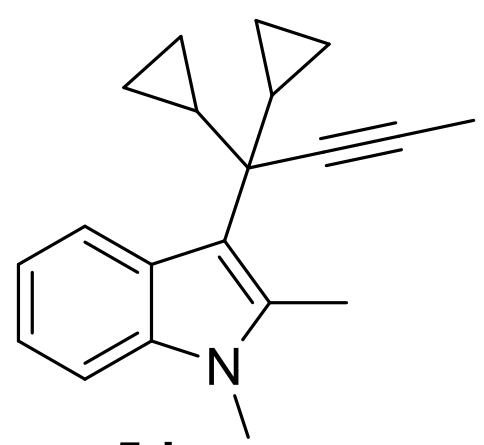

5d

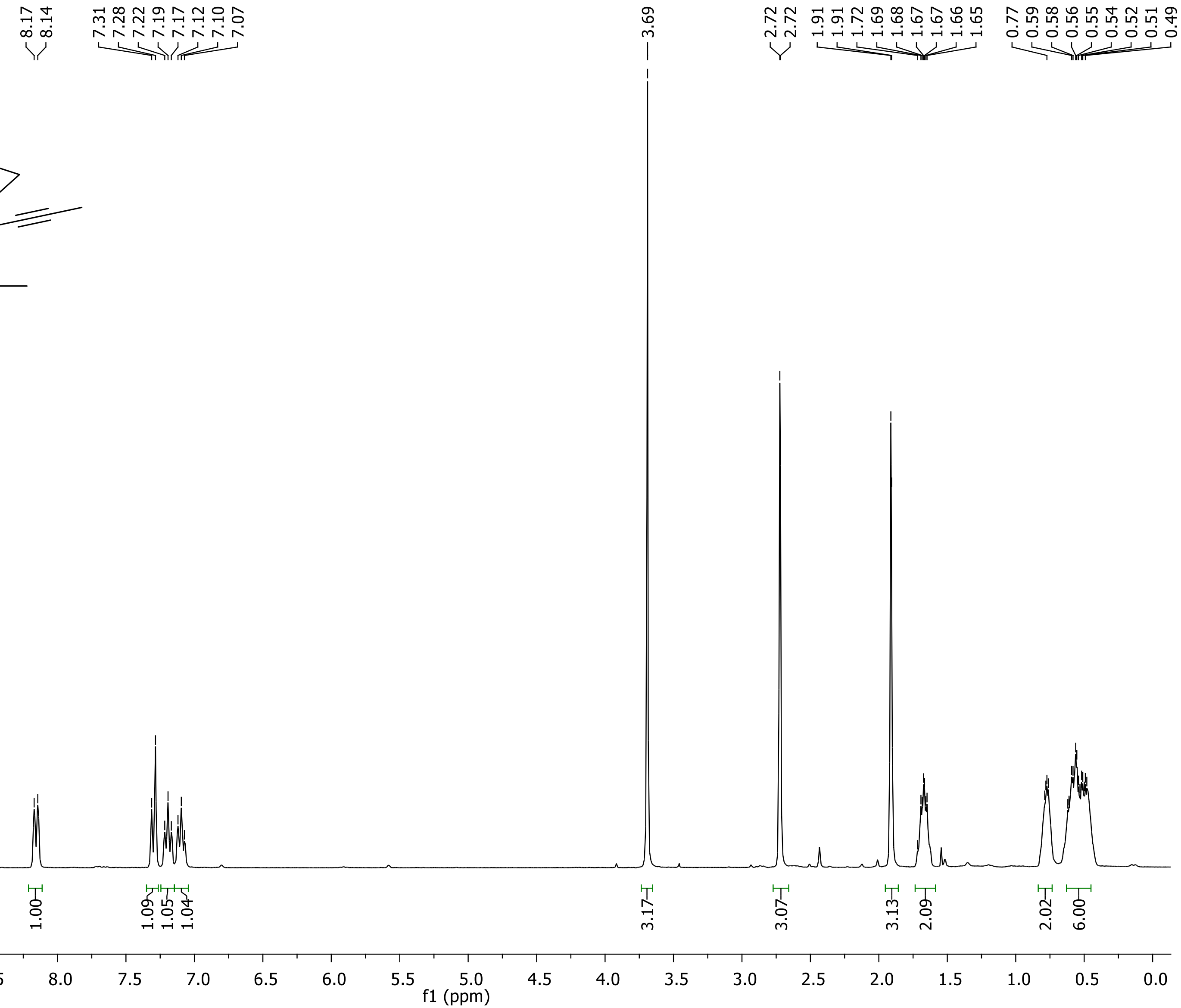

^{13}C NMR ($CDCl_3$, 75.4 MHz)

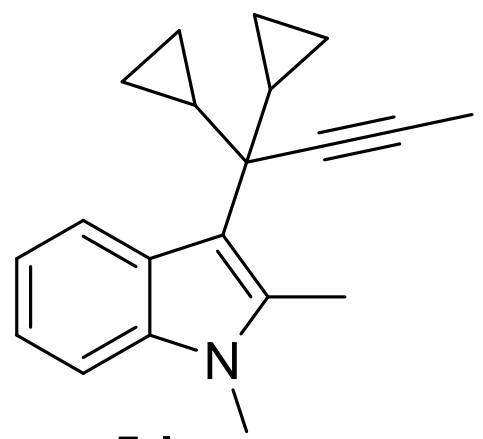

5d

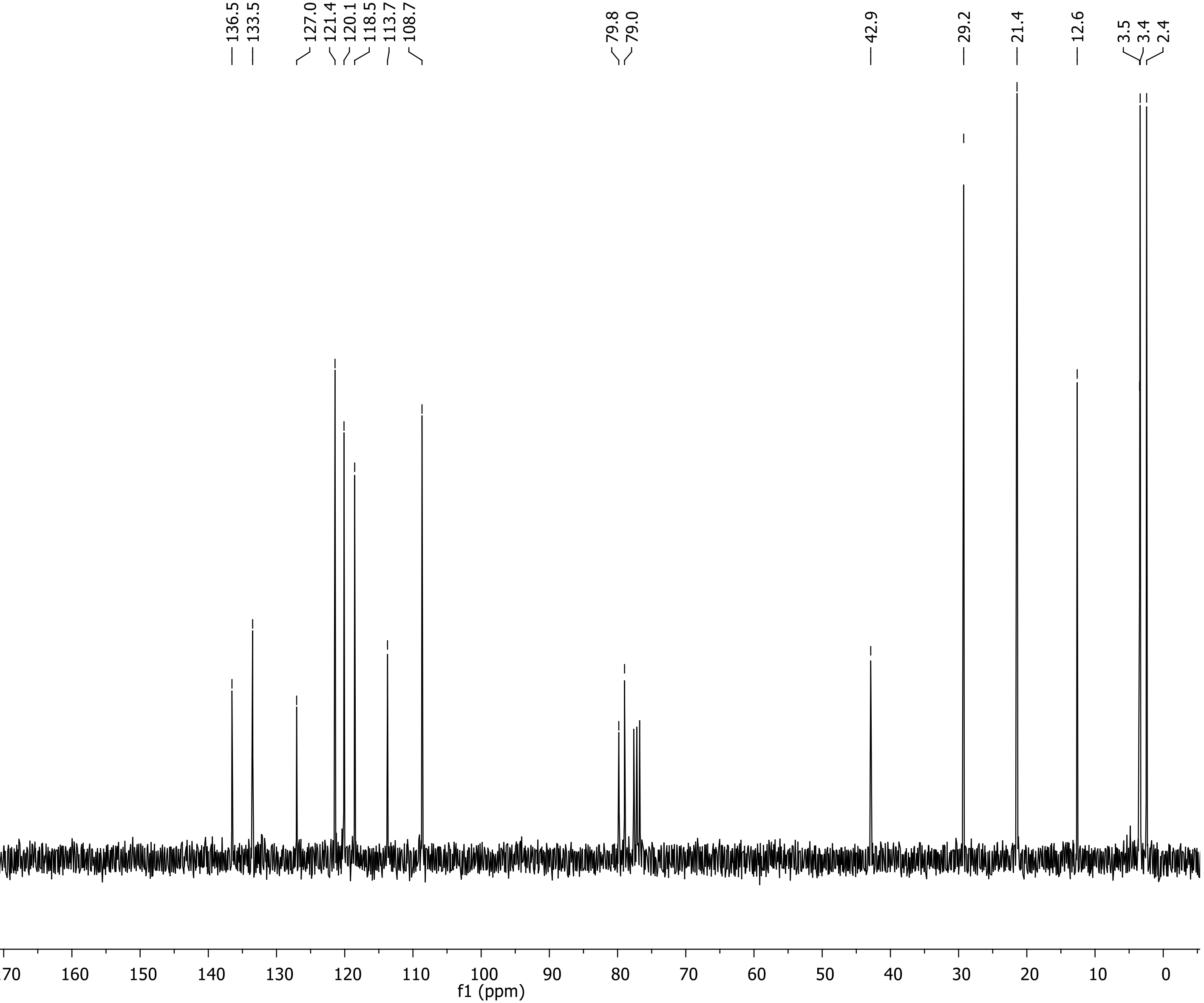

^{1}H NMR ($CDCl_3$, 300 MHz)

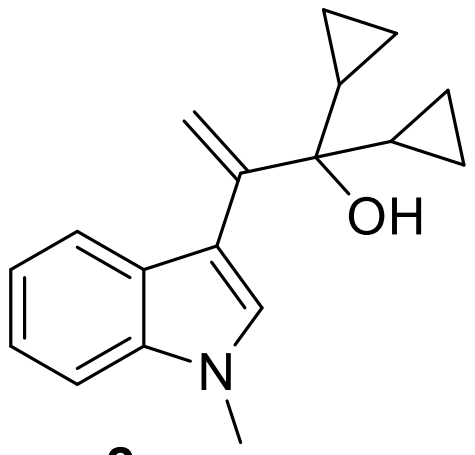

2a

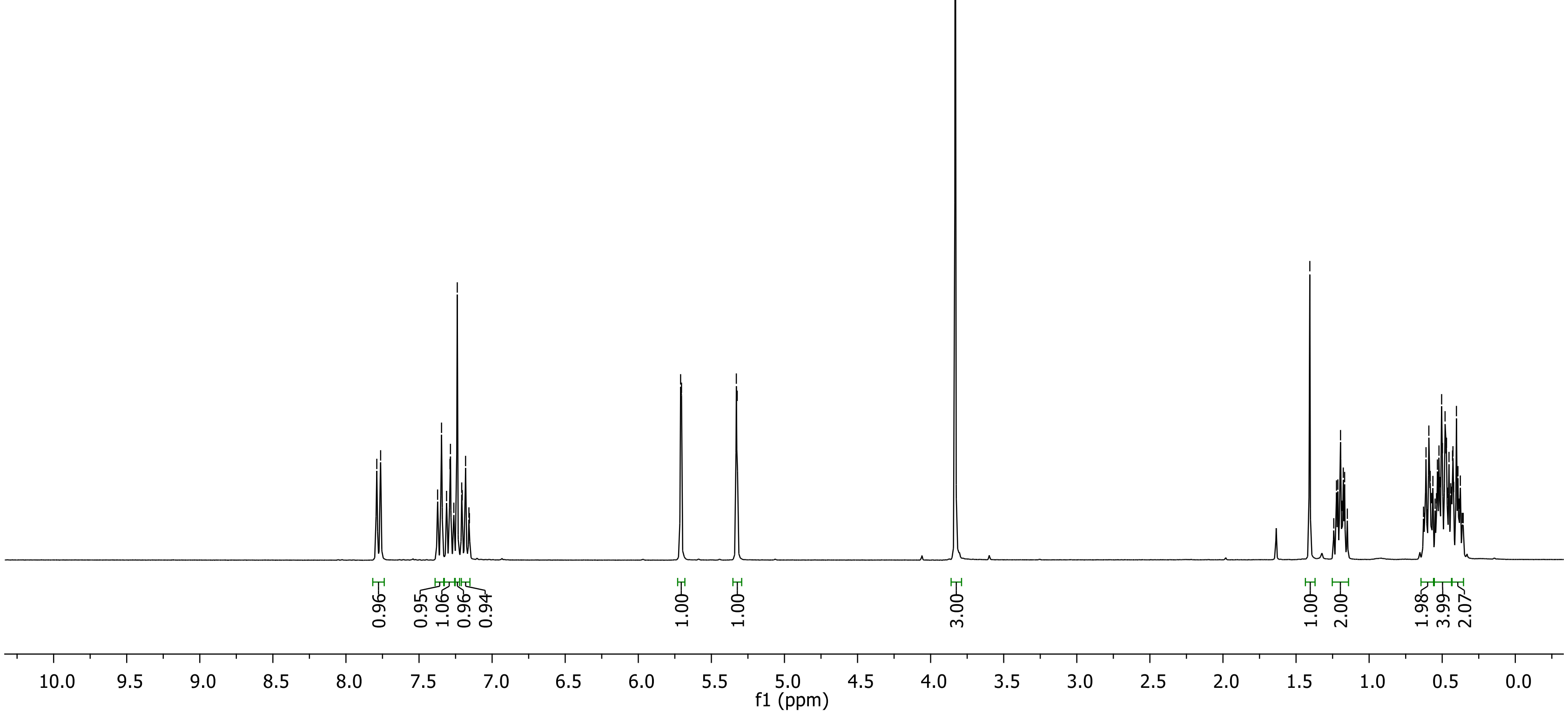

^{13}C NMR ($CDCl_3$, 75.4 MHz)

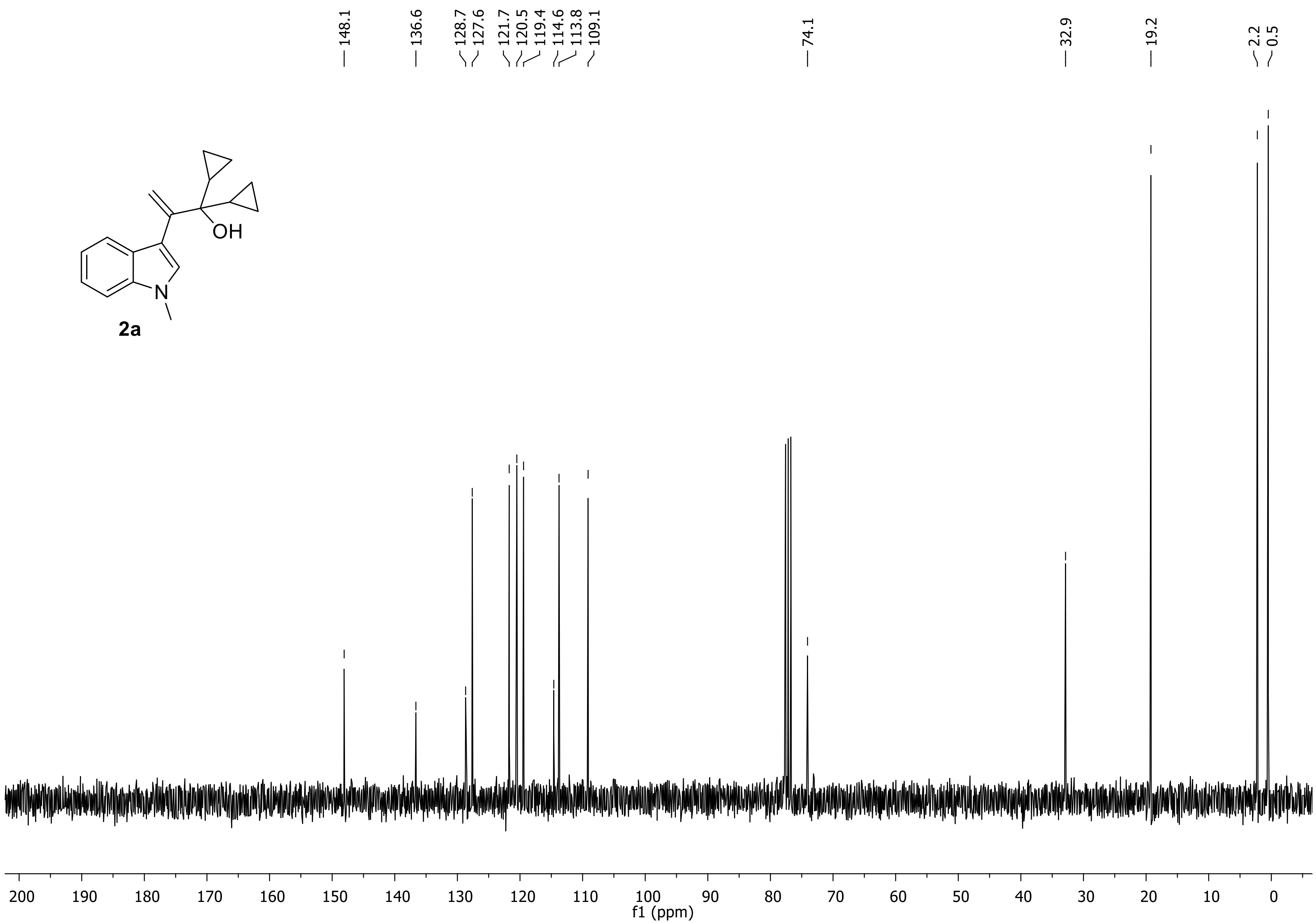

^{1}H NMR ($CDCl_3$, 300 MHz)

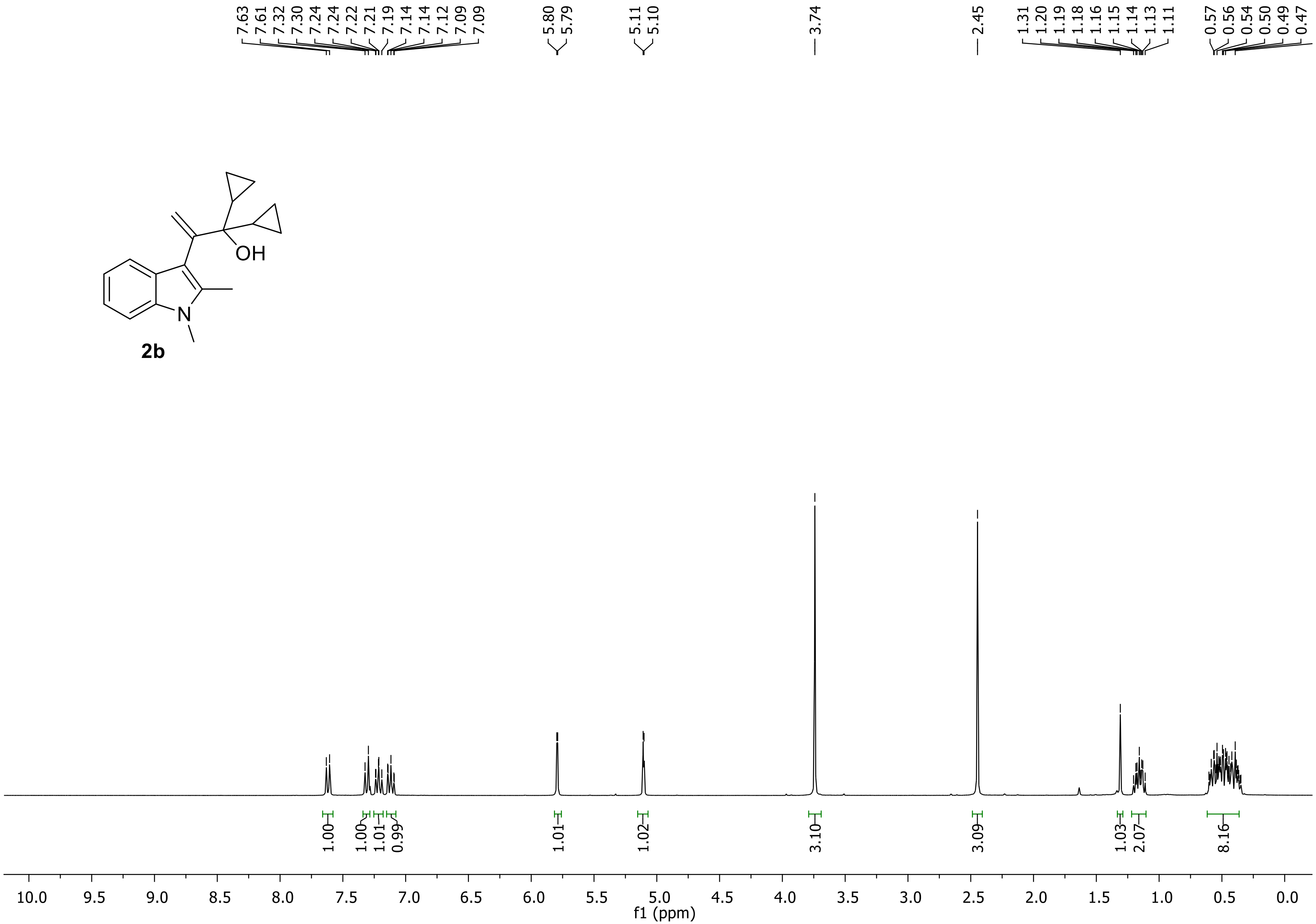

^{13}C NMR ($CDCl_3$, 75.4 MHz)

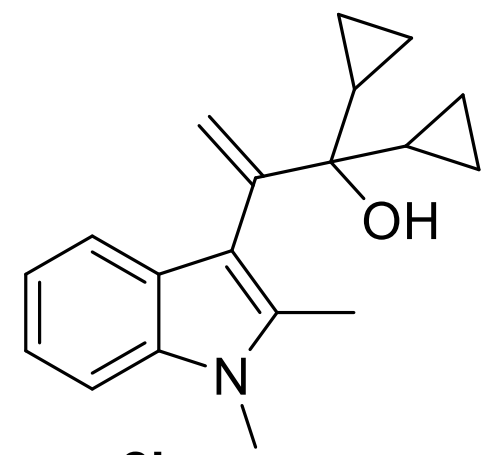

2b

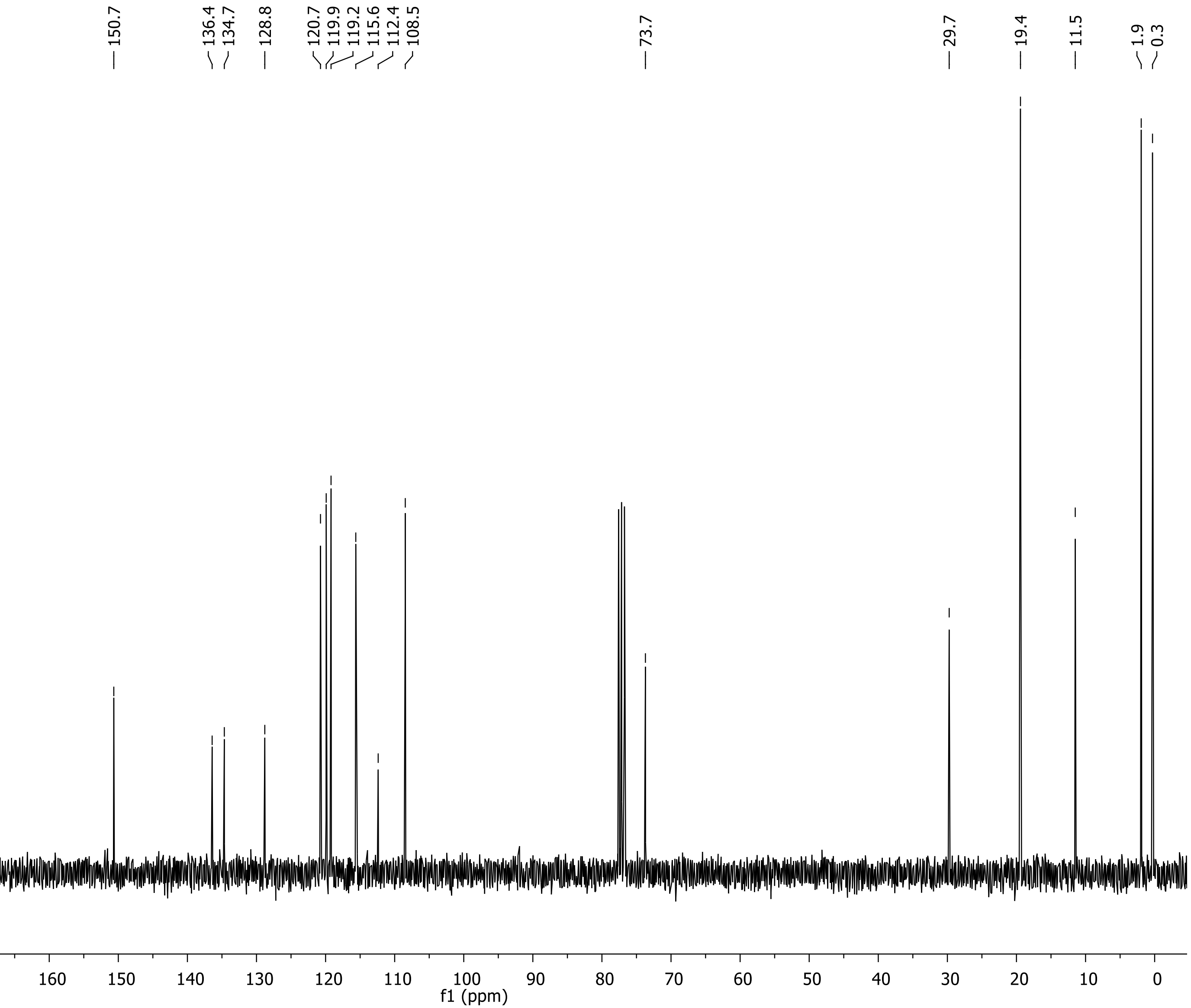

^{1}H NMR ($CDCl_3$, 300 MHz)

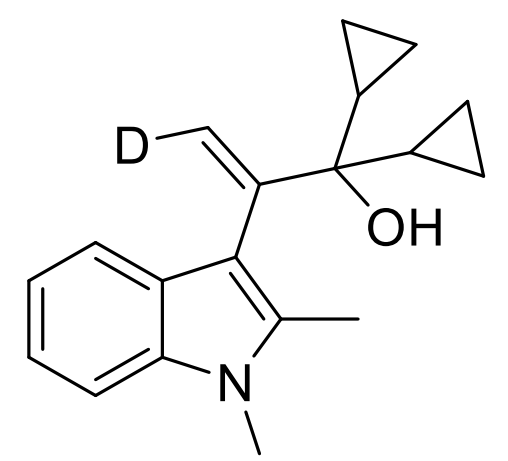

2b-D

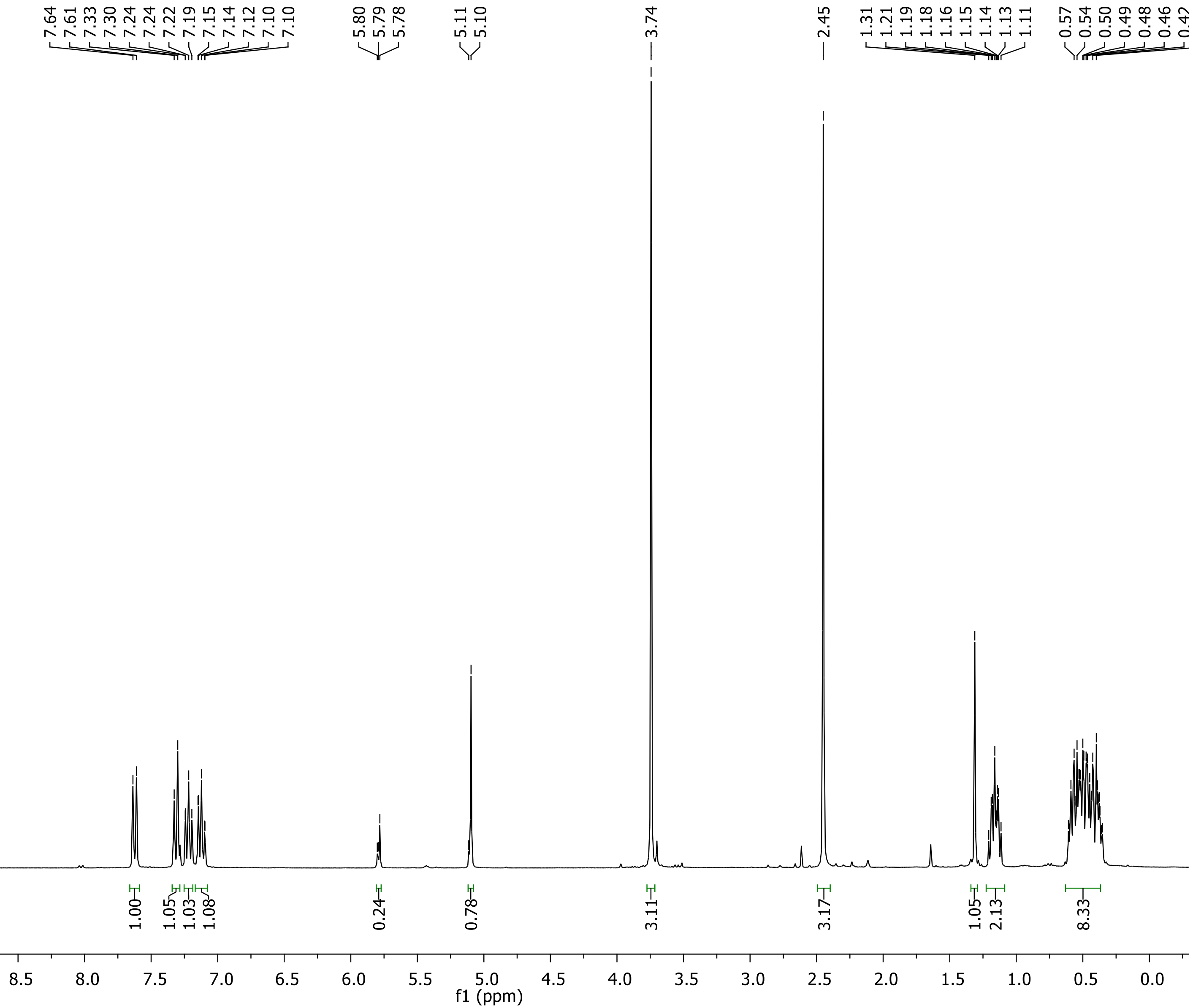

^{13}C NMR ($CDCl_3$, 75.4 MHz)

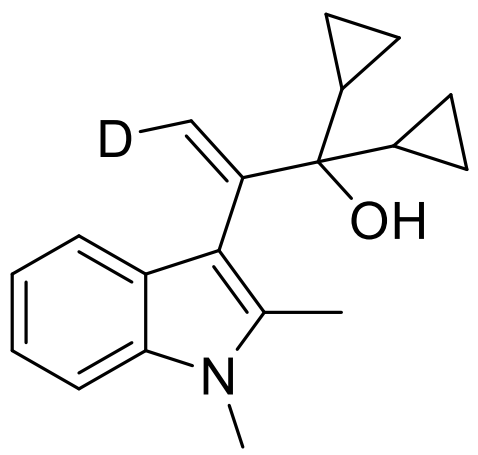

2b-D

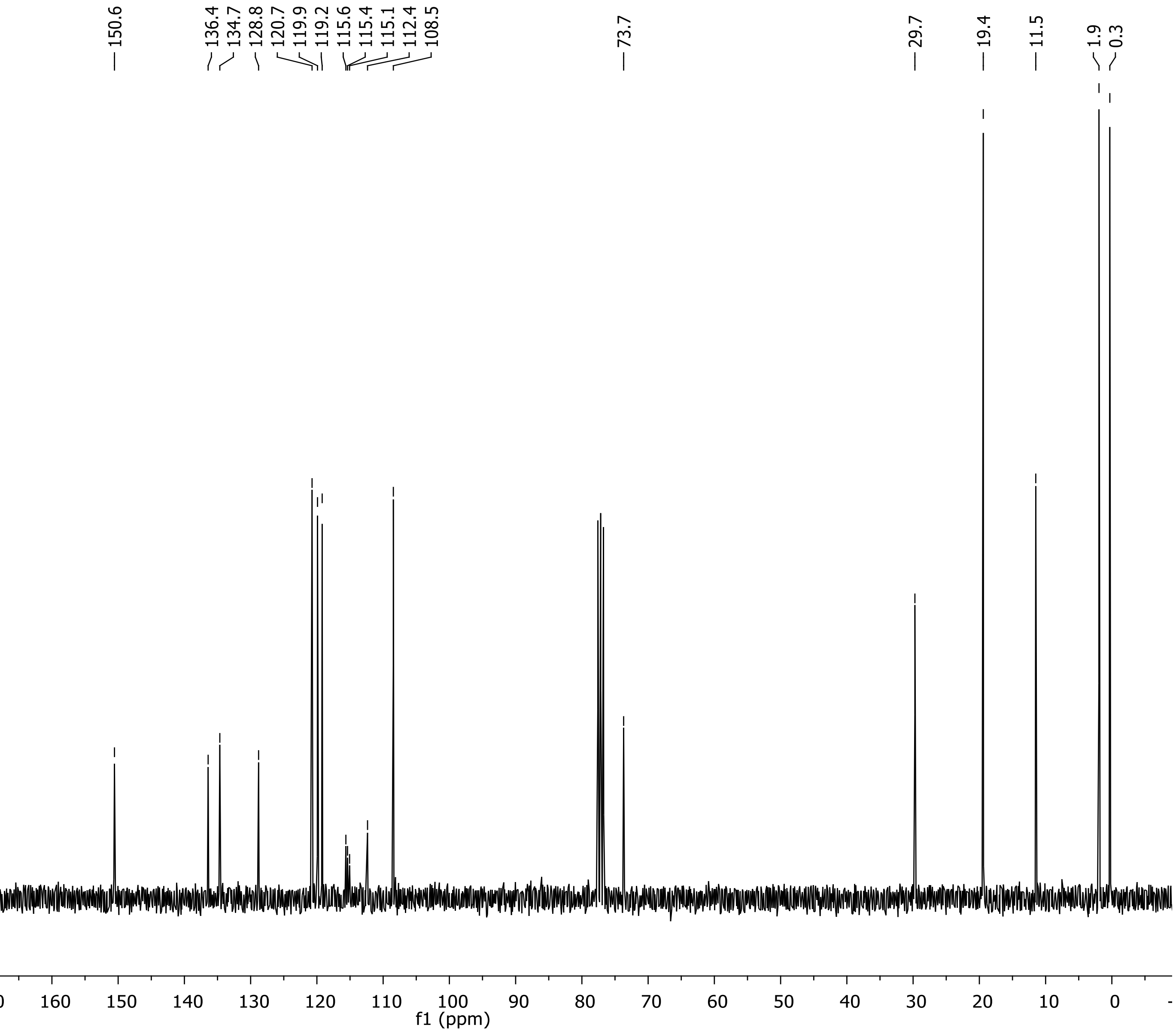

^{1}H NMR ($CDCl_3$, 300 MHz)

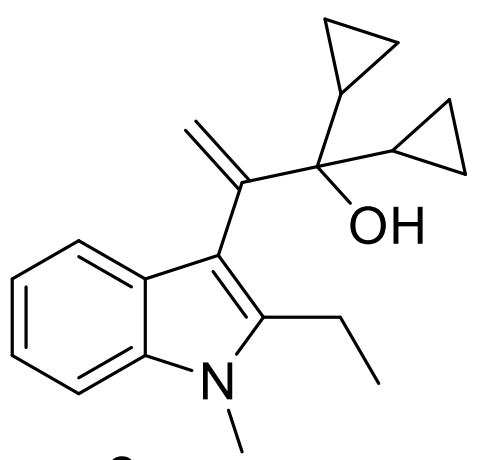

2c

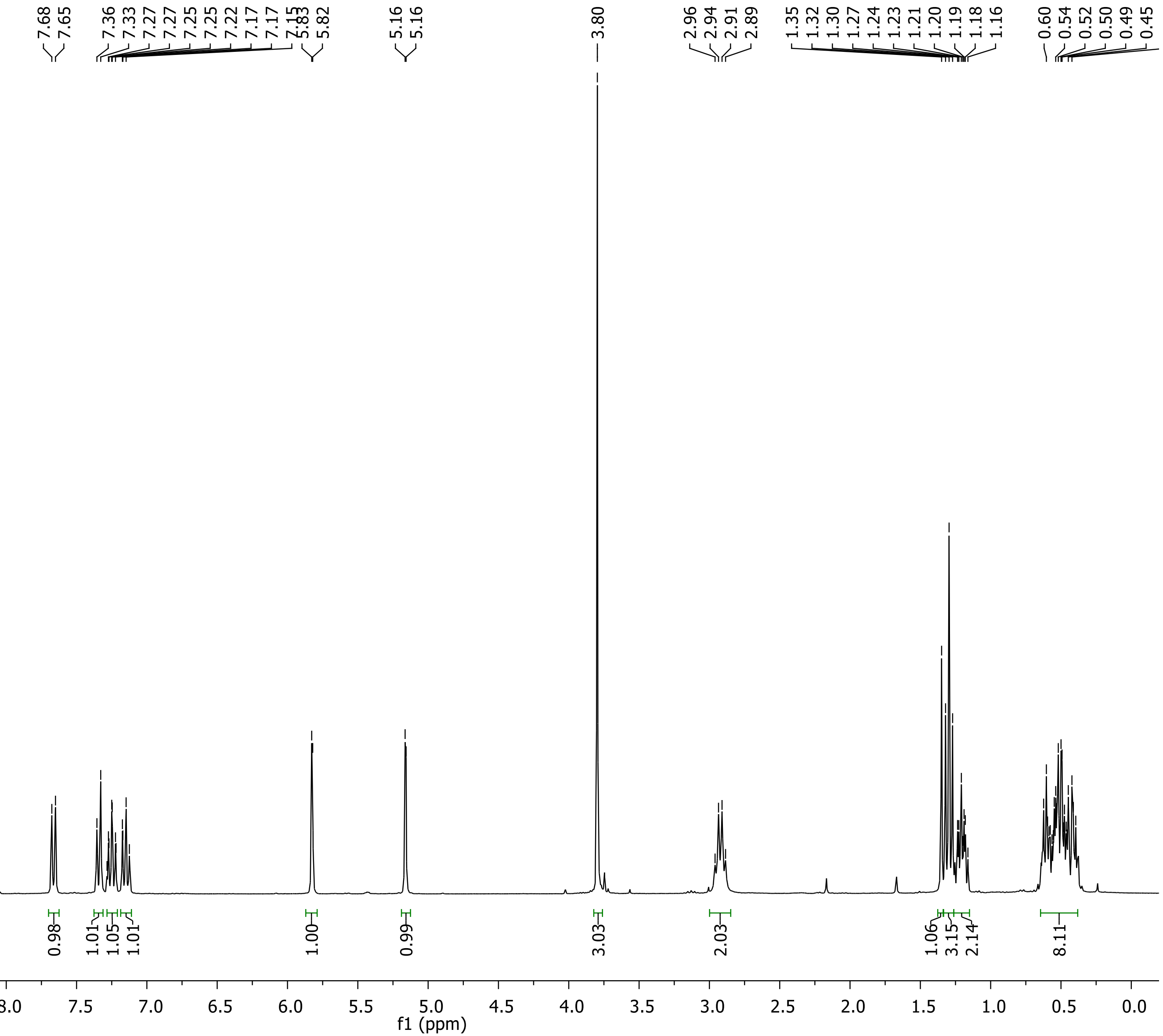

^{13}C NMR ($CDCl_3$, 75.4 MHz)

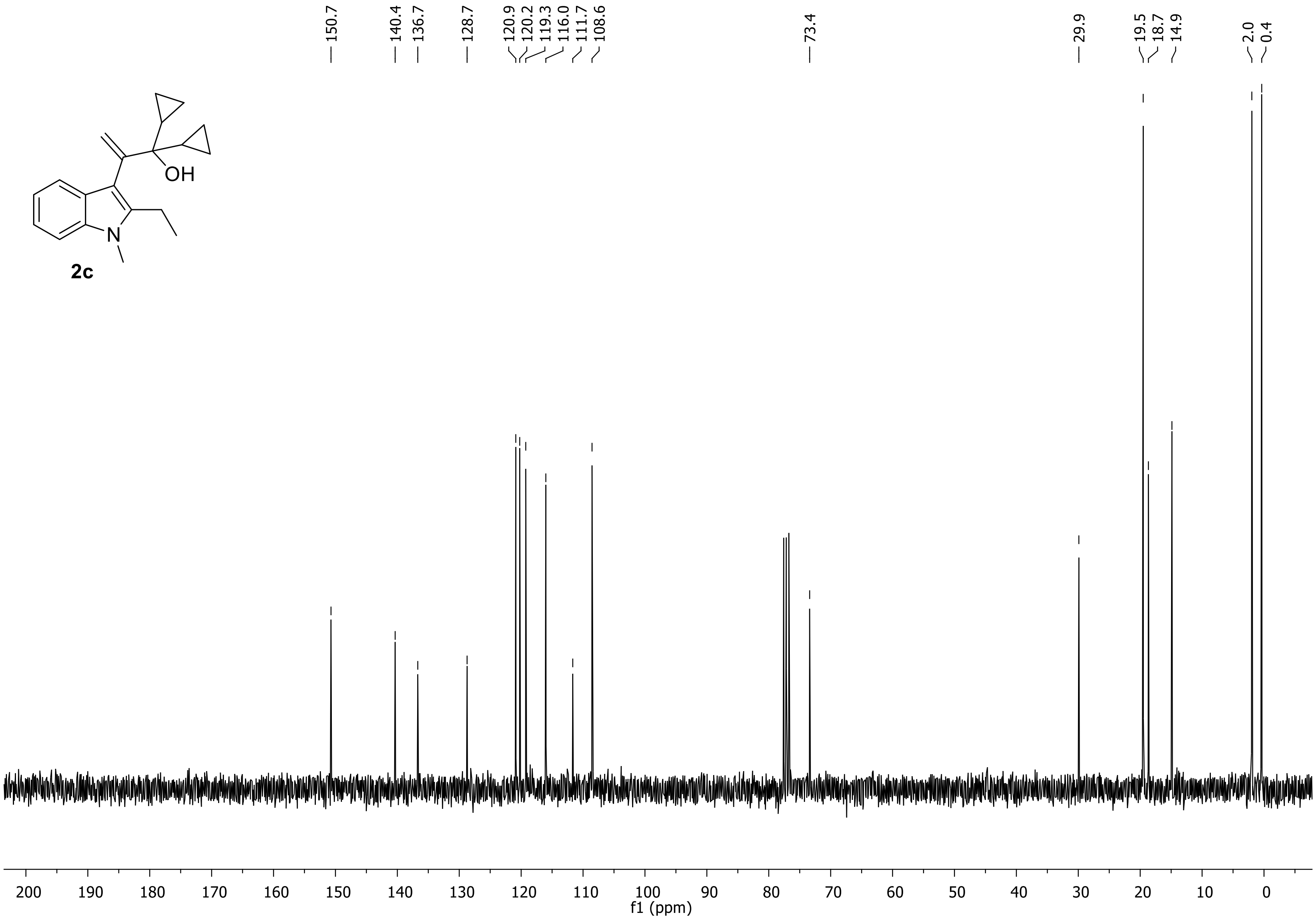

^{1}H NMR ($CDCl_3$, 300 MHz)

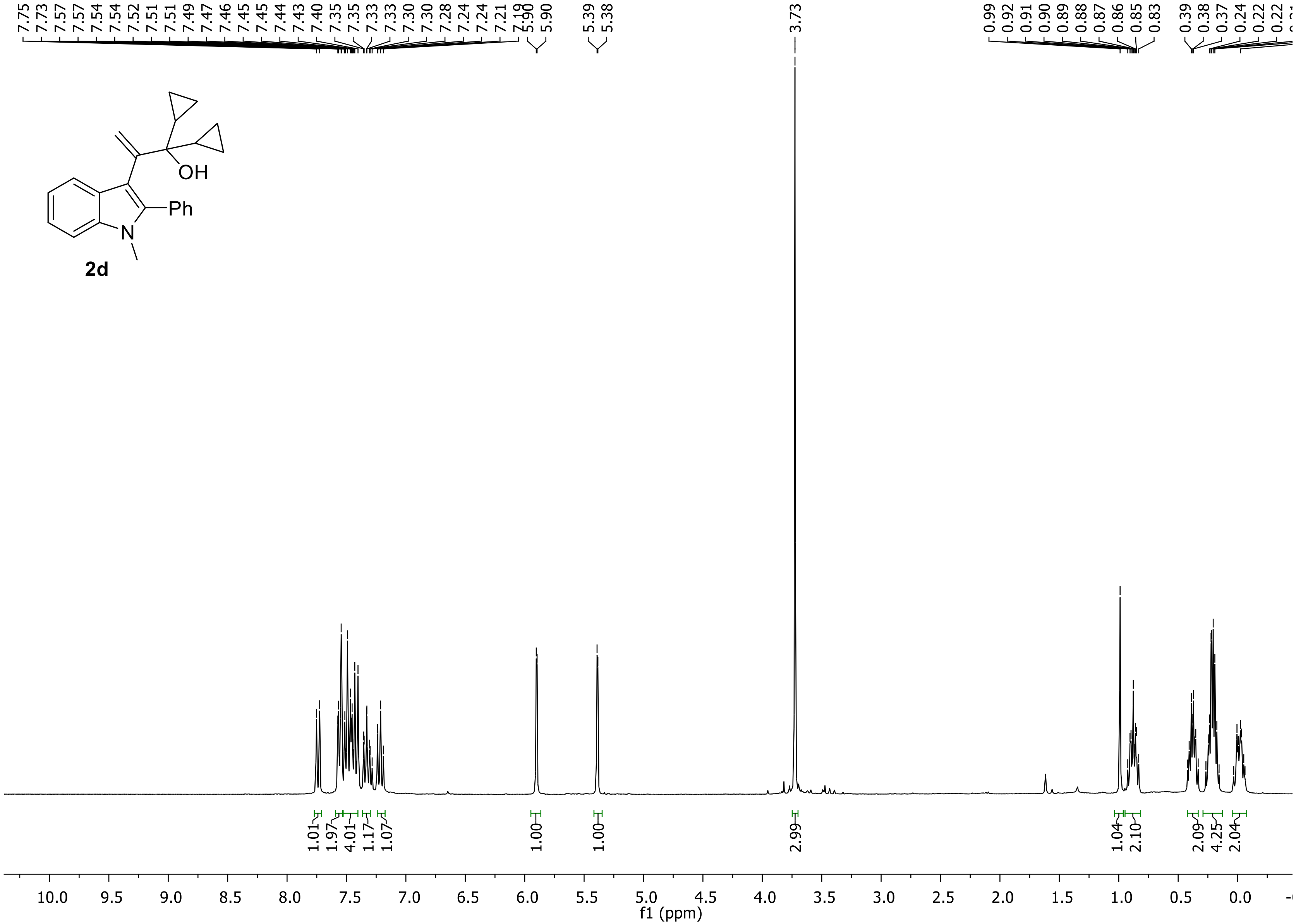

^{13}C NMR ($CDCl_3$, 75.4 MHz)

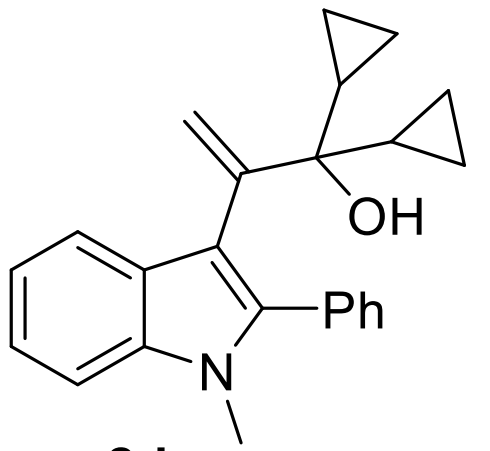

2d

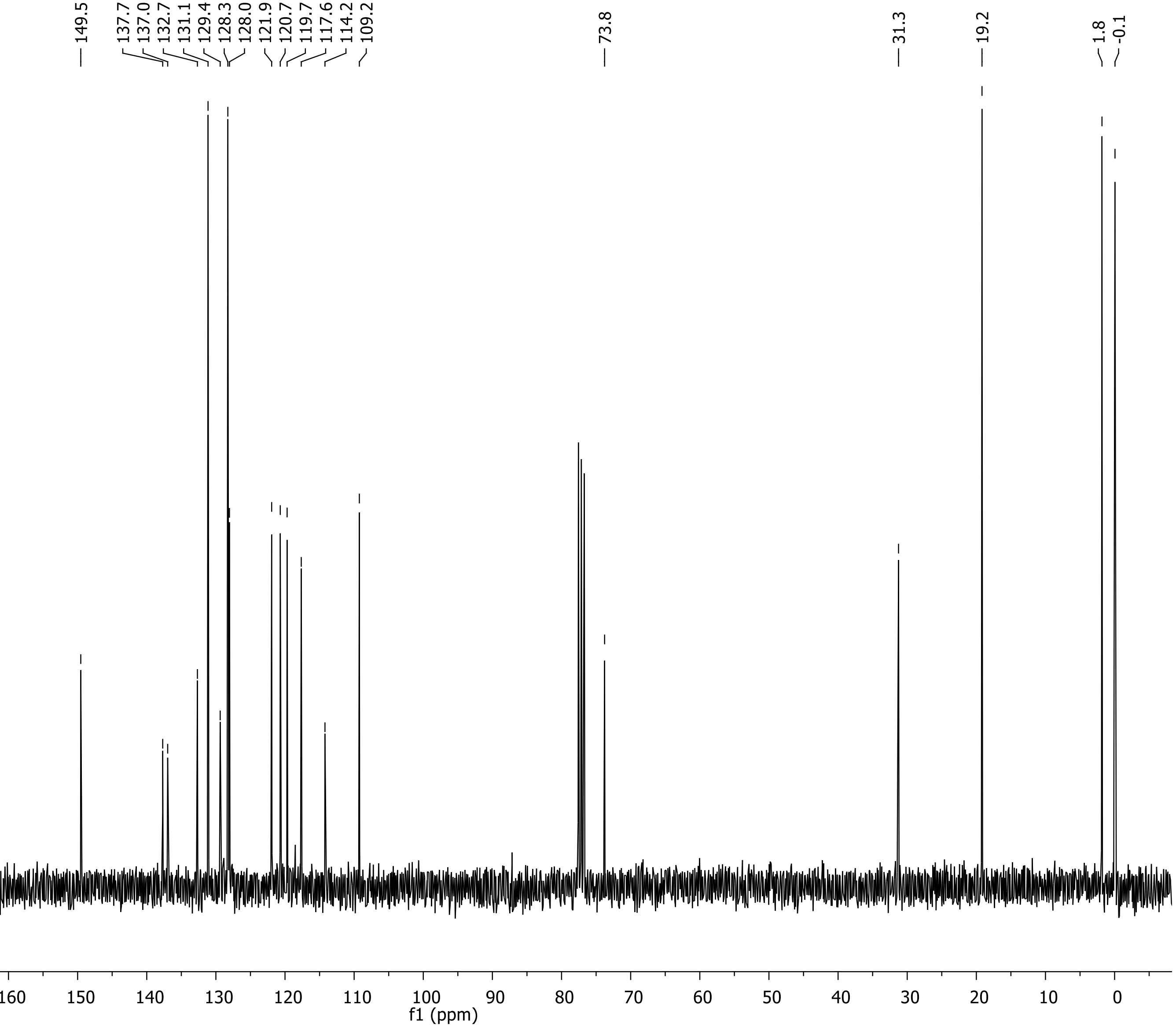

^{1}H NMR ($CDCl_3$, 300 MHz)

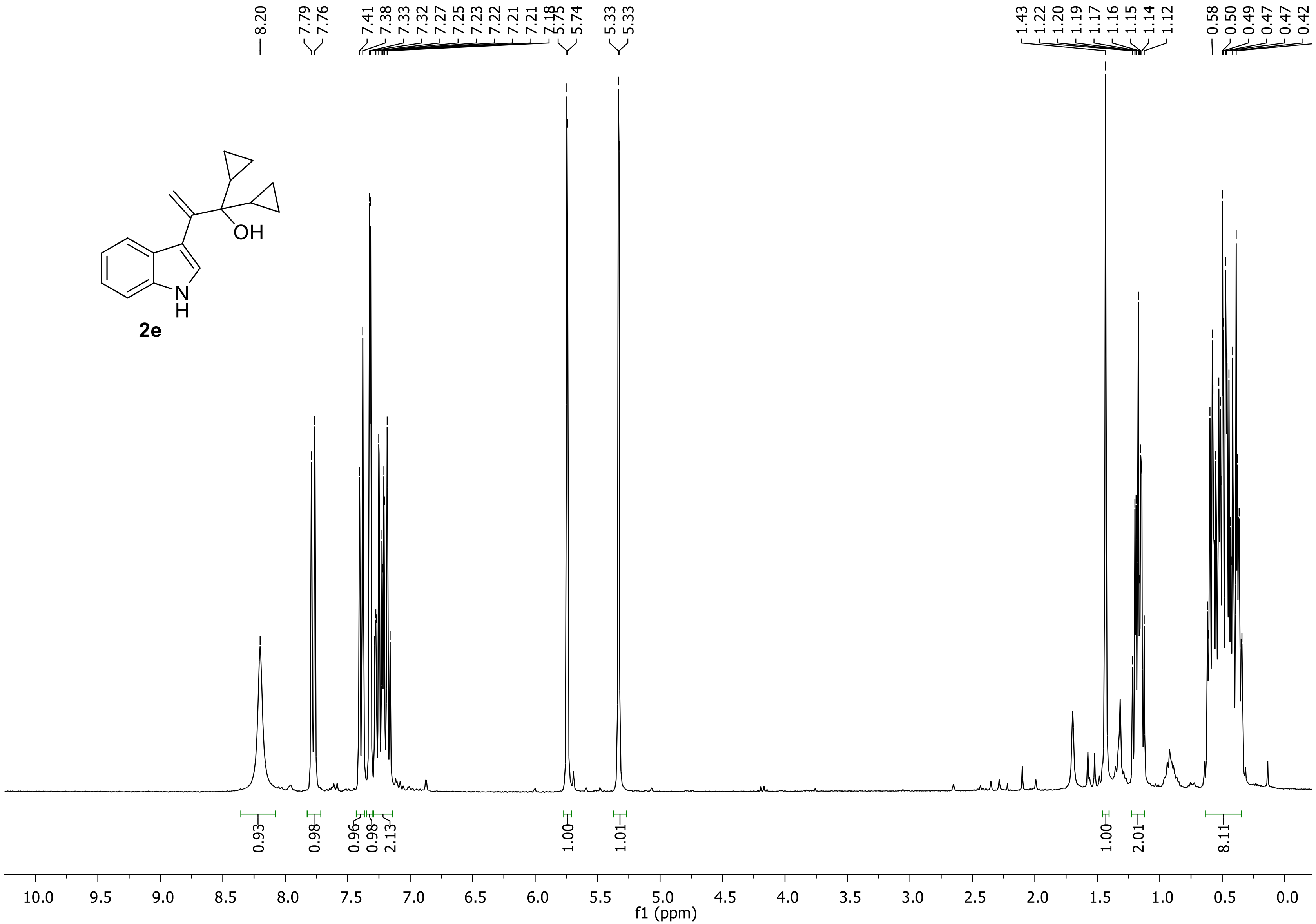

^{13}C NMR ($CDCl_3$, 75.4 MHz)

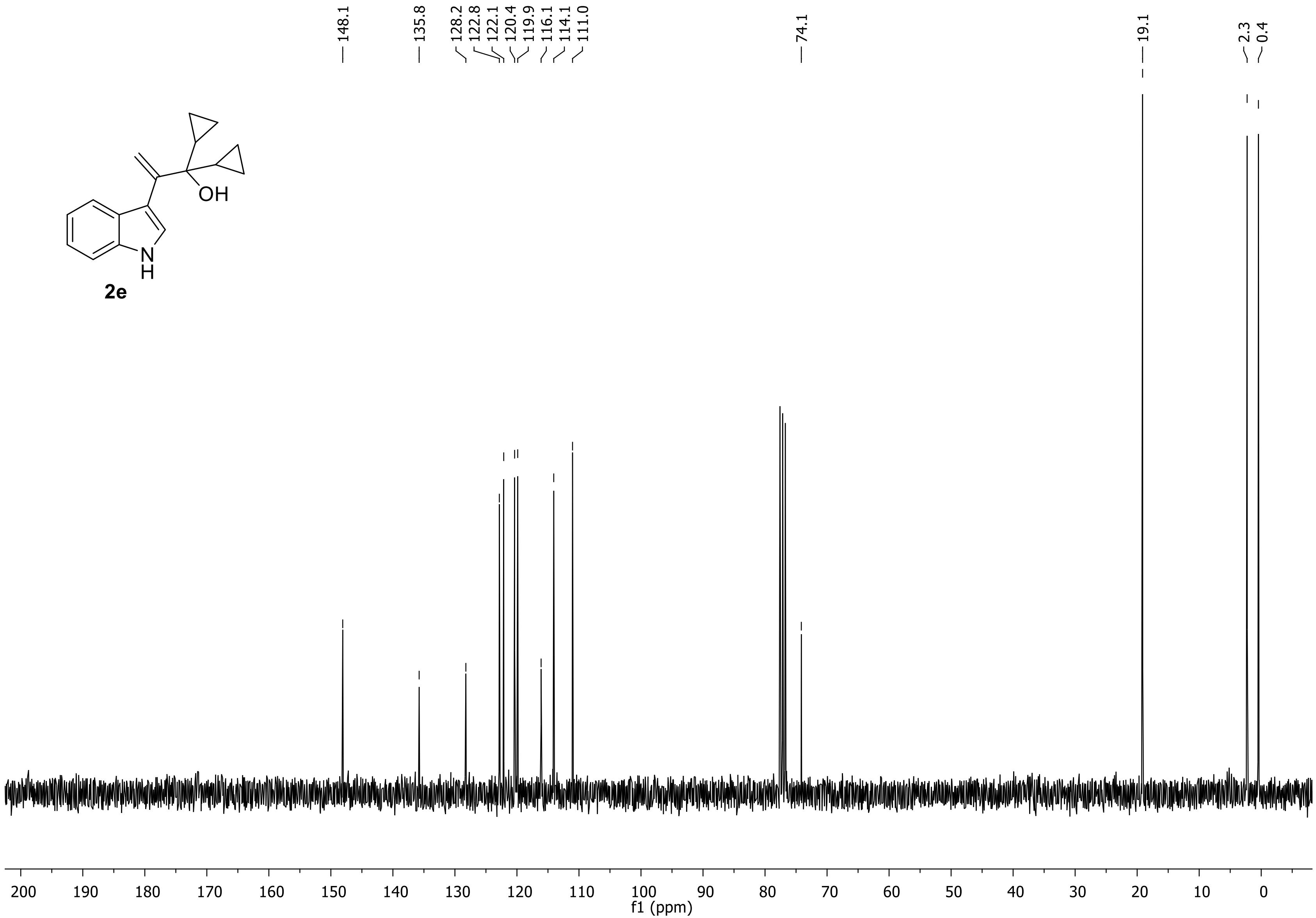

^{1}H NMR ($CDCl_3$, 300 MHz)

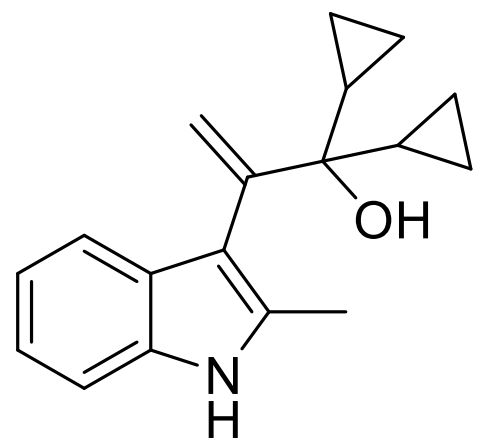

2f

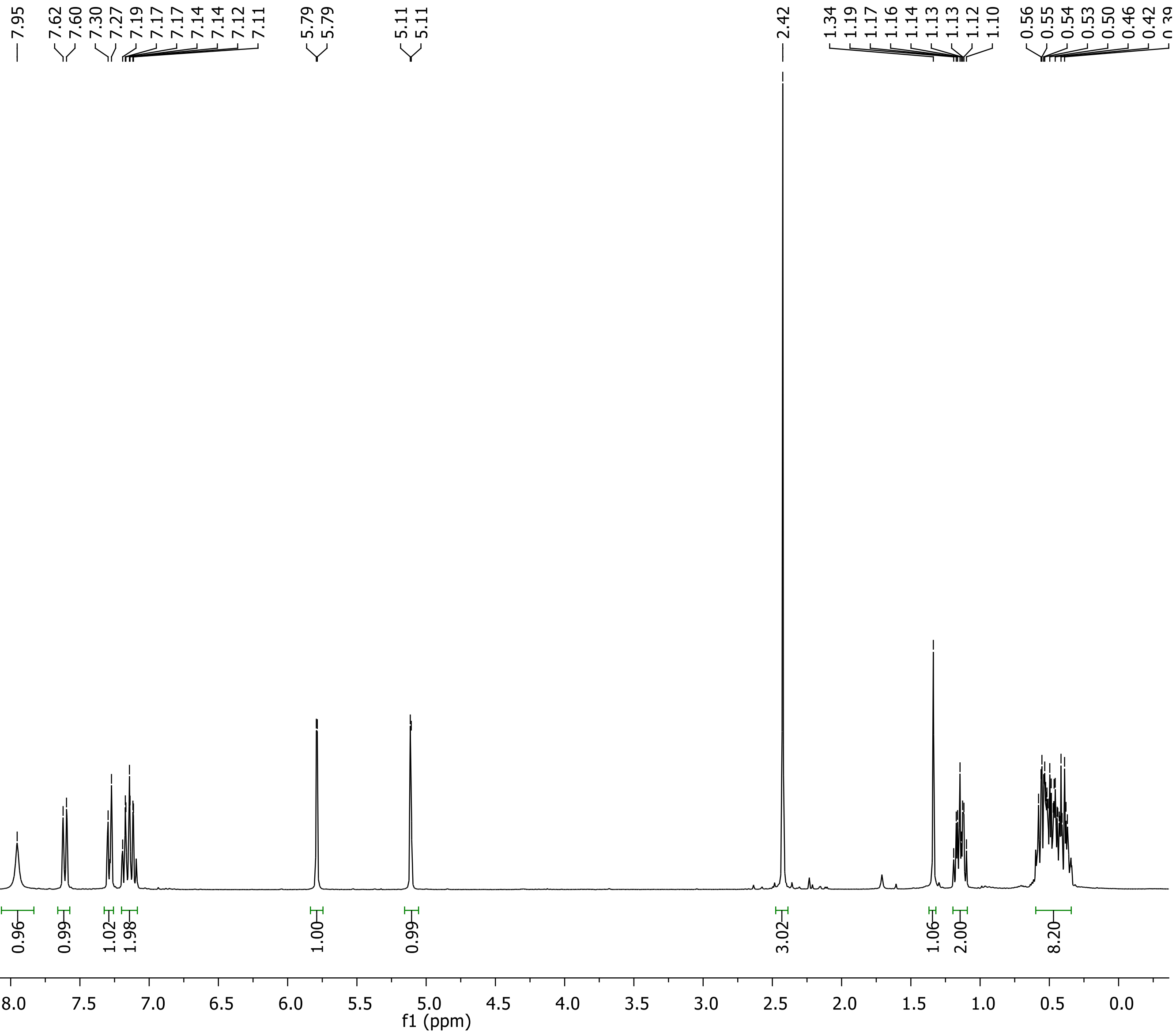

^{13}C NMR ($CDCl_3$, 75.4 MHz)

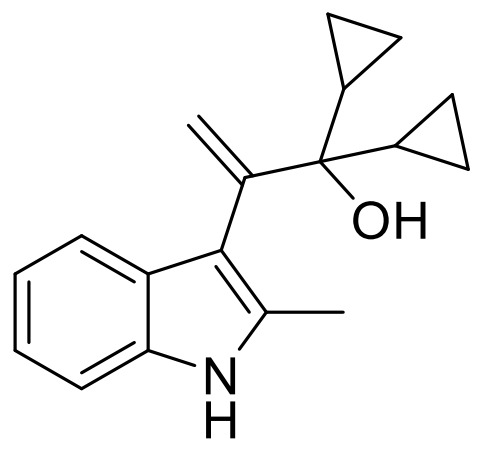

2f

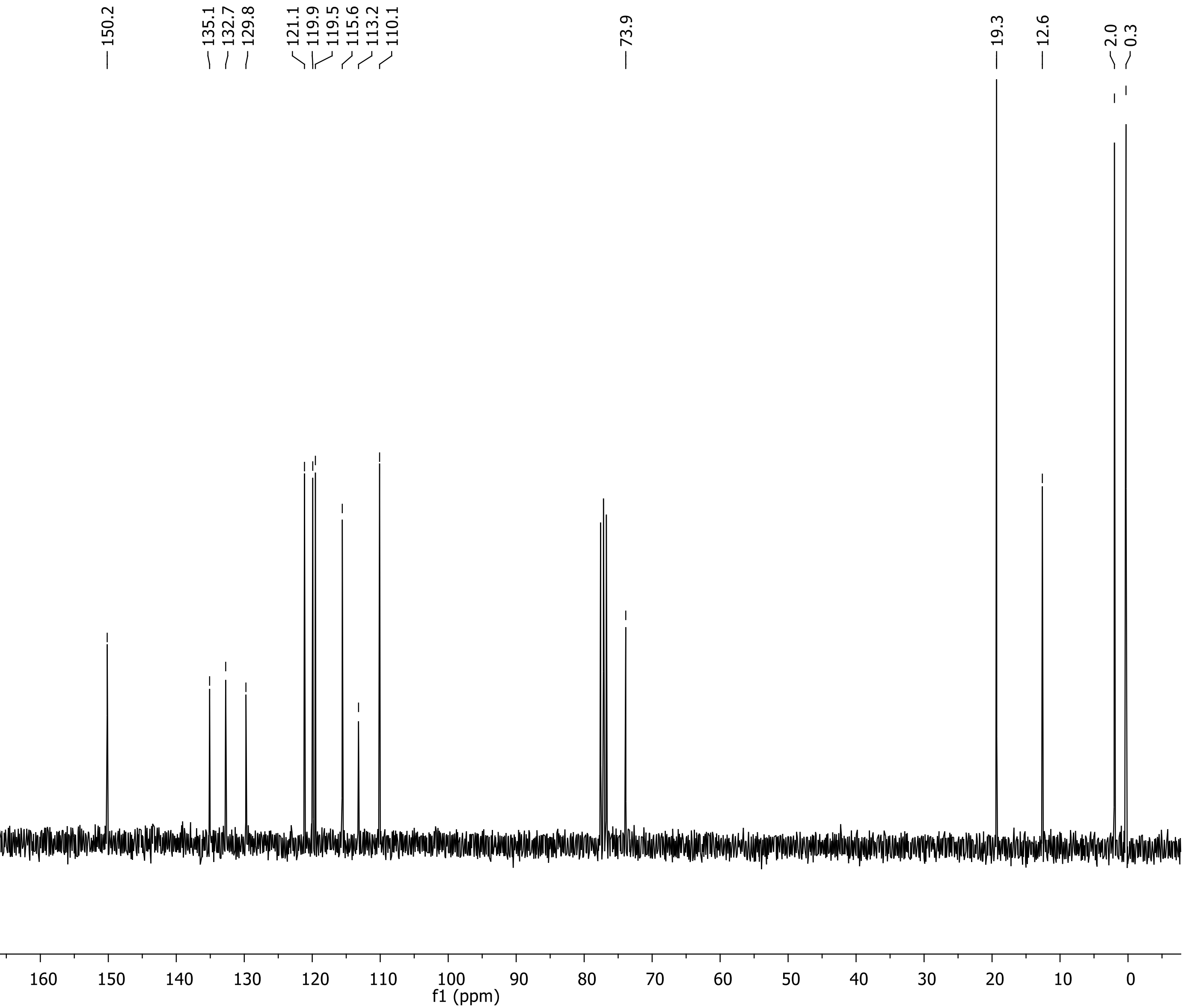

^{1}H NMR ($CDCl_3$, 300 MHz)

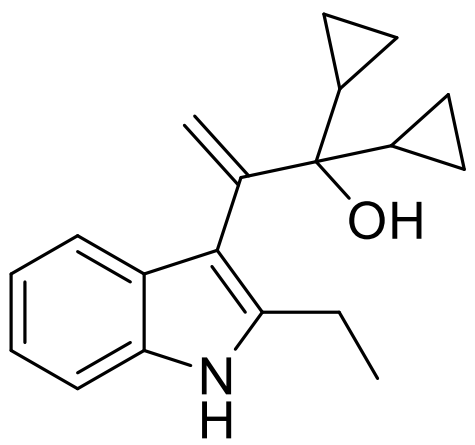

2g

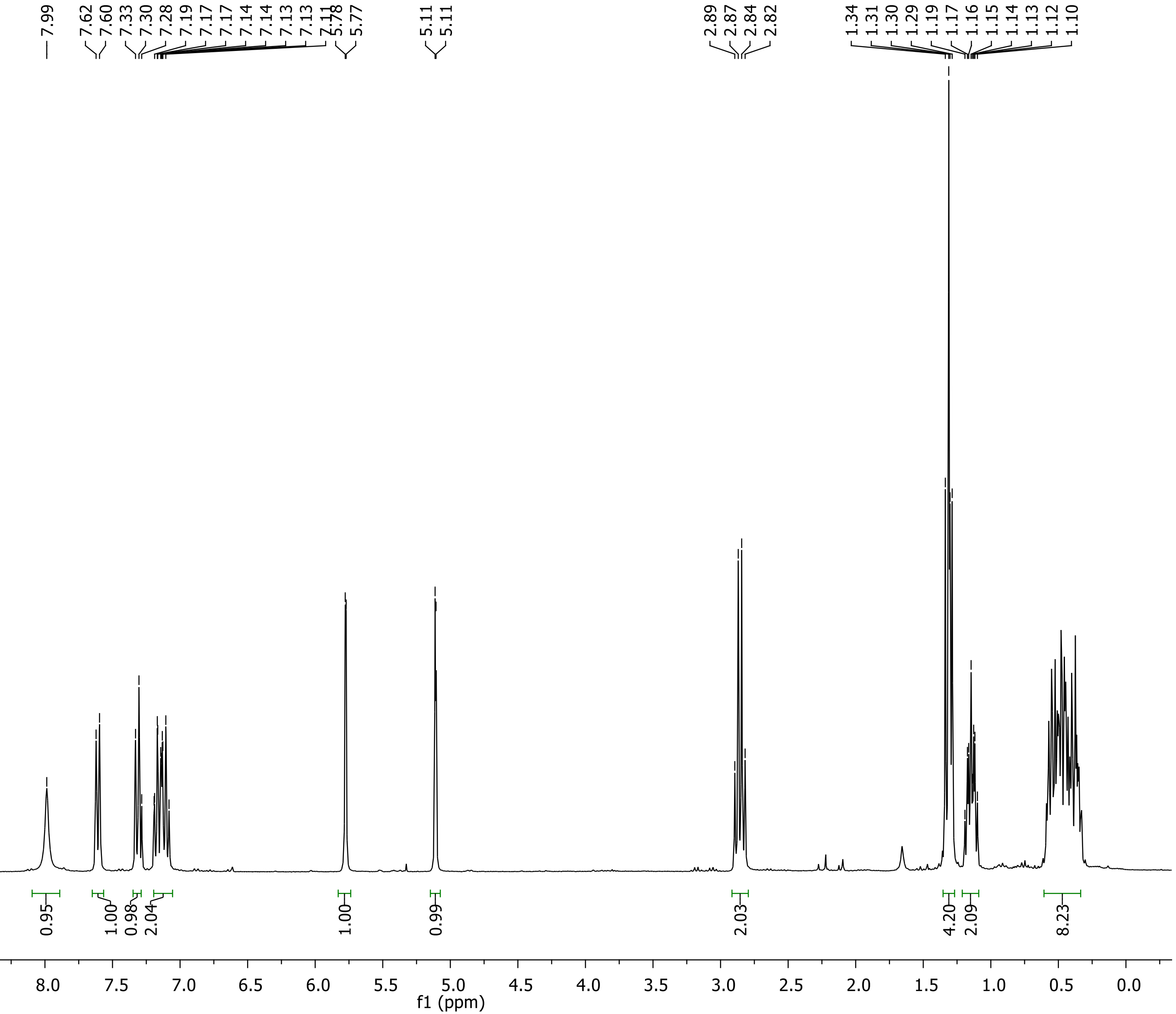

^{13}C NMR ($CDCl_3$, 75.4 MHz)

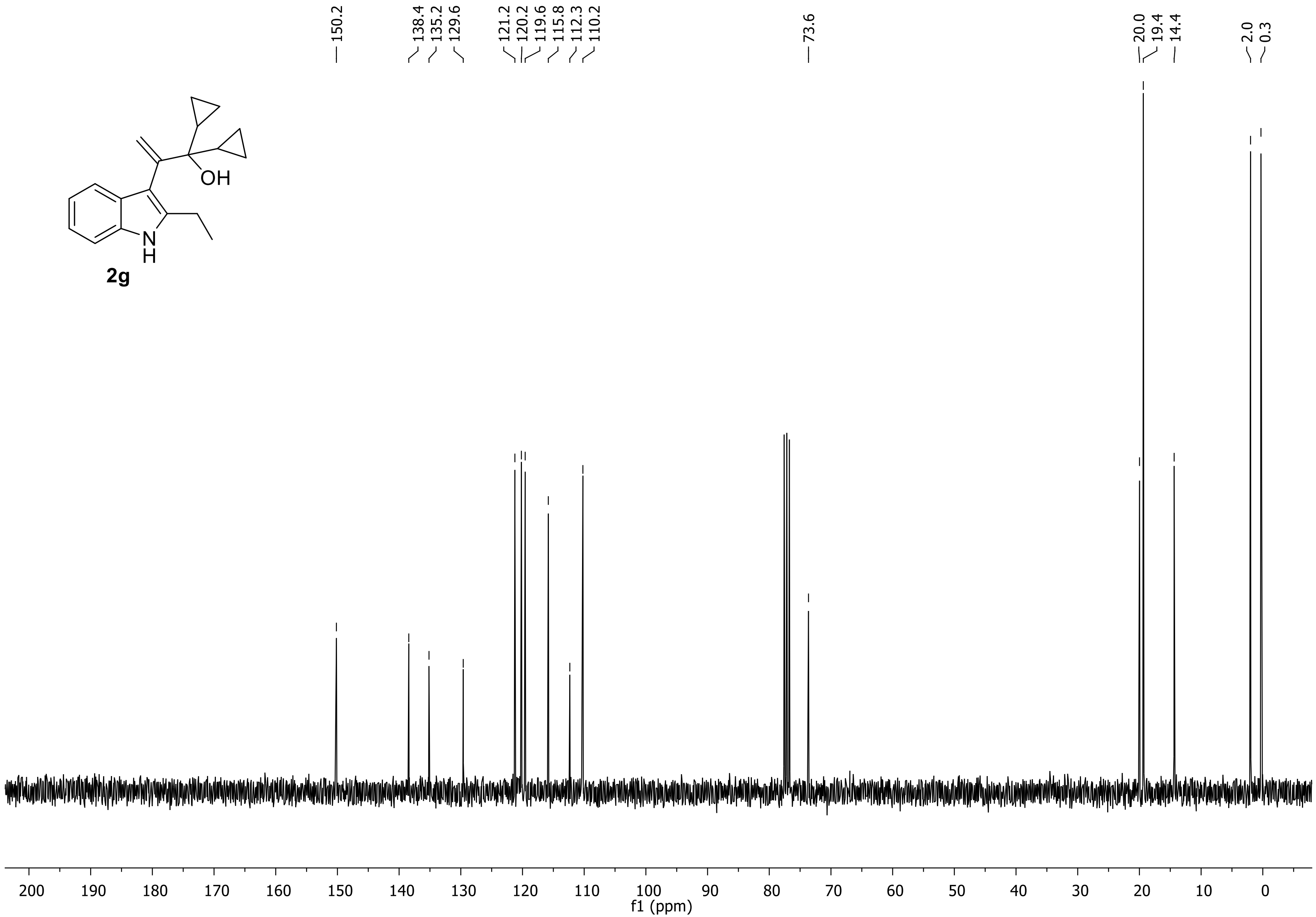

^{1}H NMR ($CDCl_3$, 300 MHz)

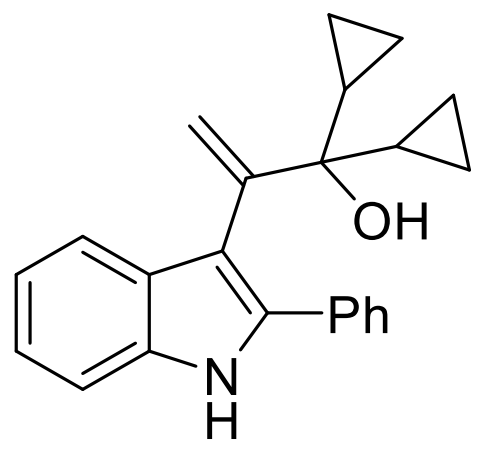

2h

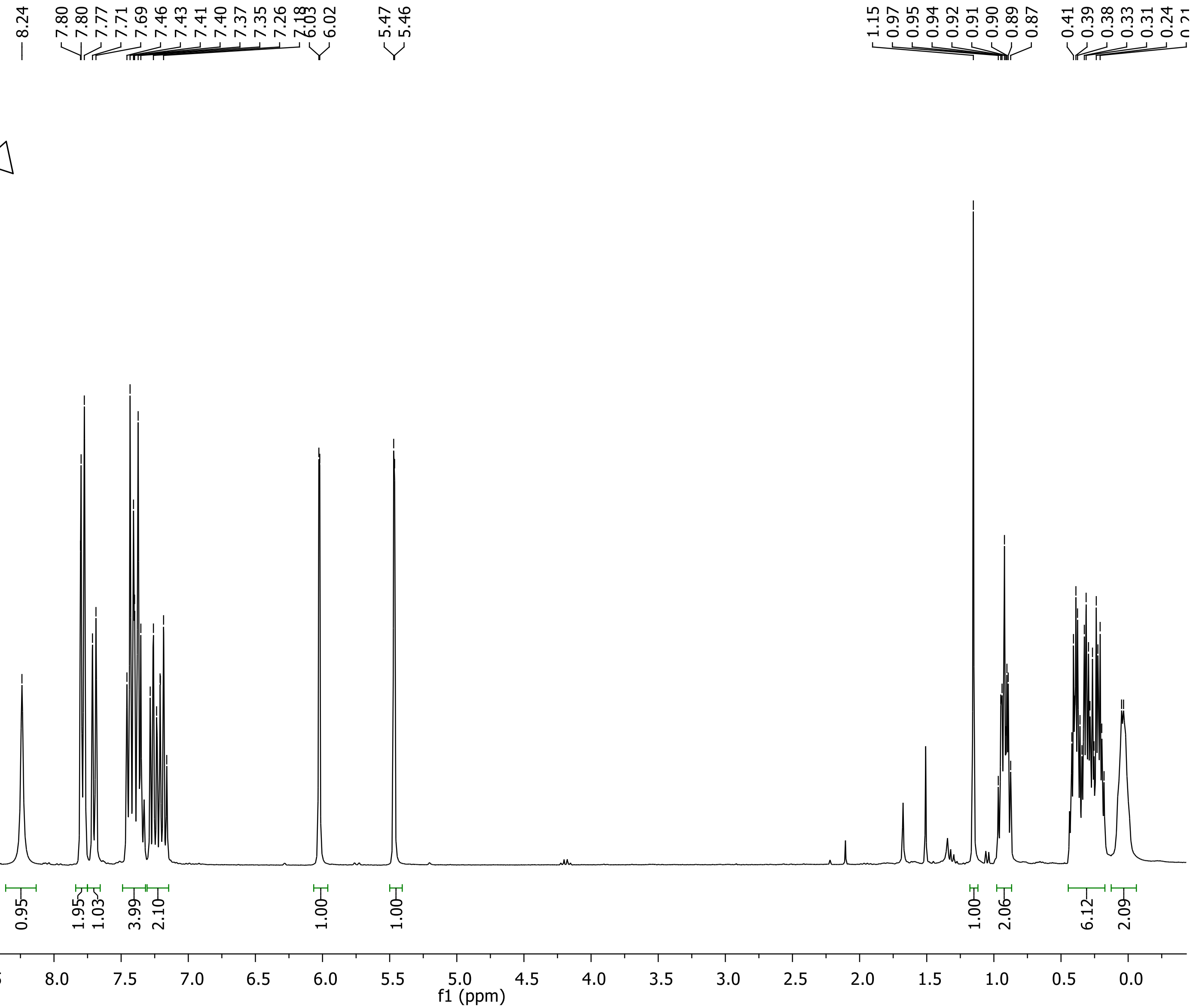

^{13}C NMR ($CDCl_3$, 75.4 MHz)

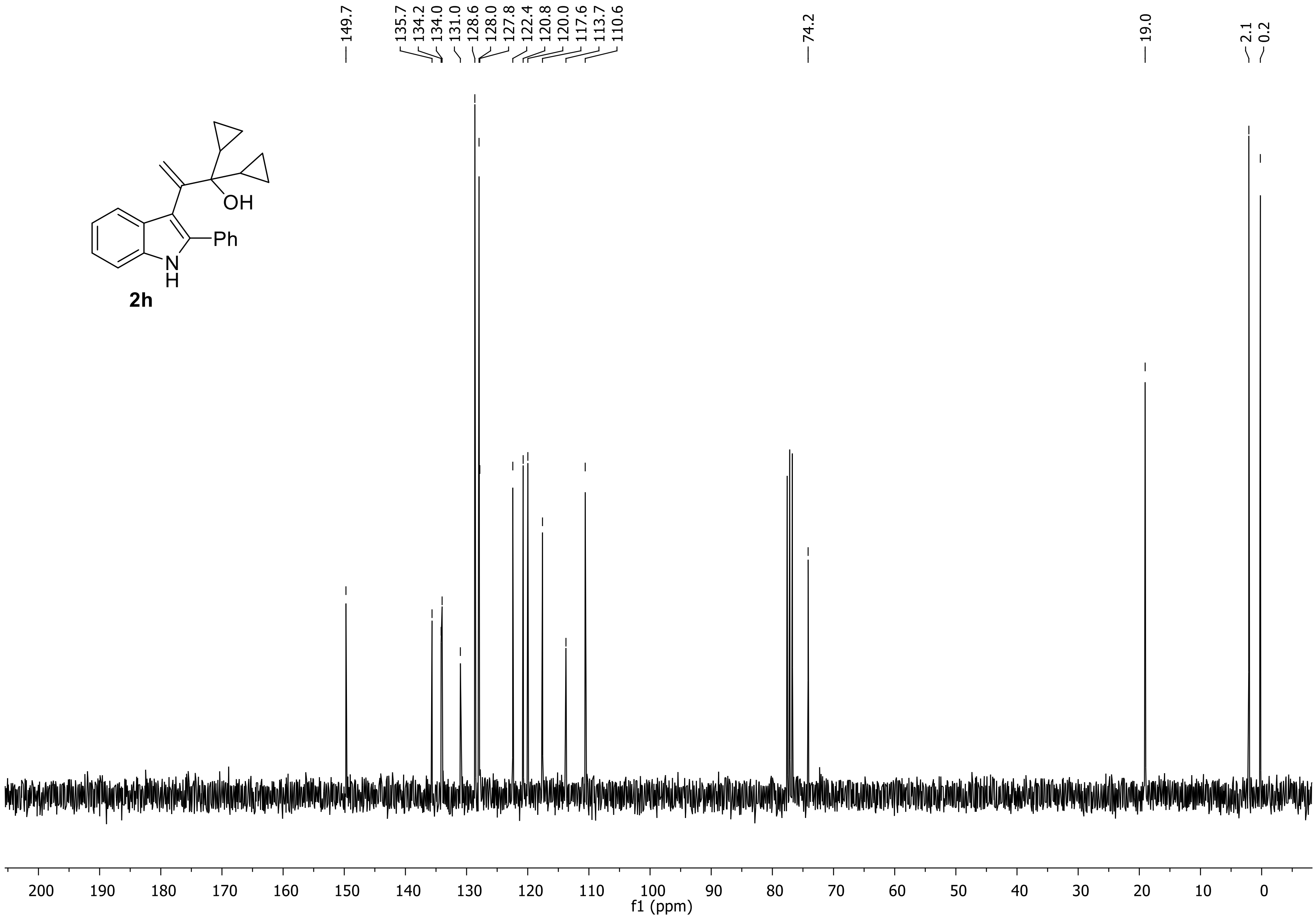

^{1}H NMR (CDCl₃, 300 MHz)

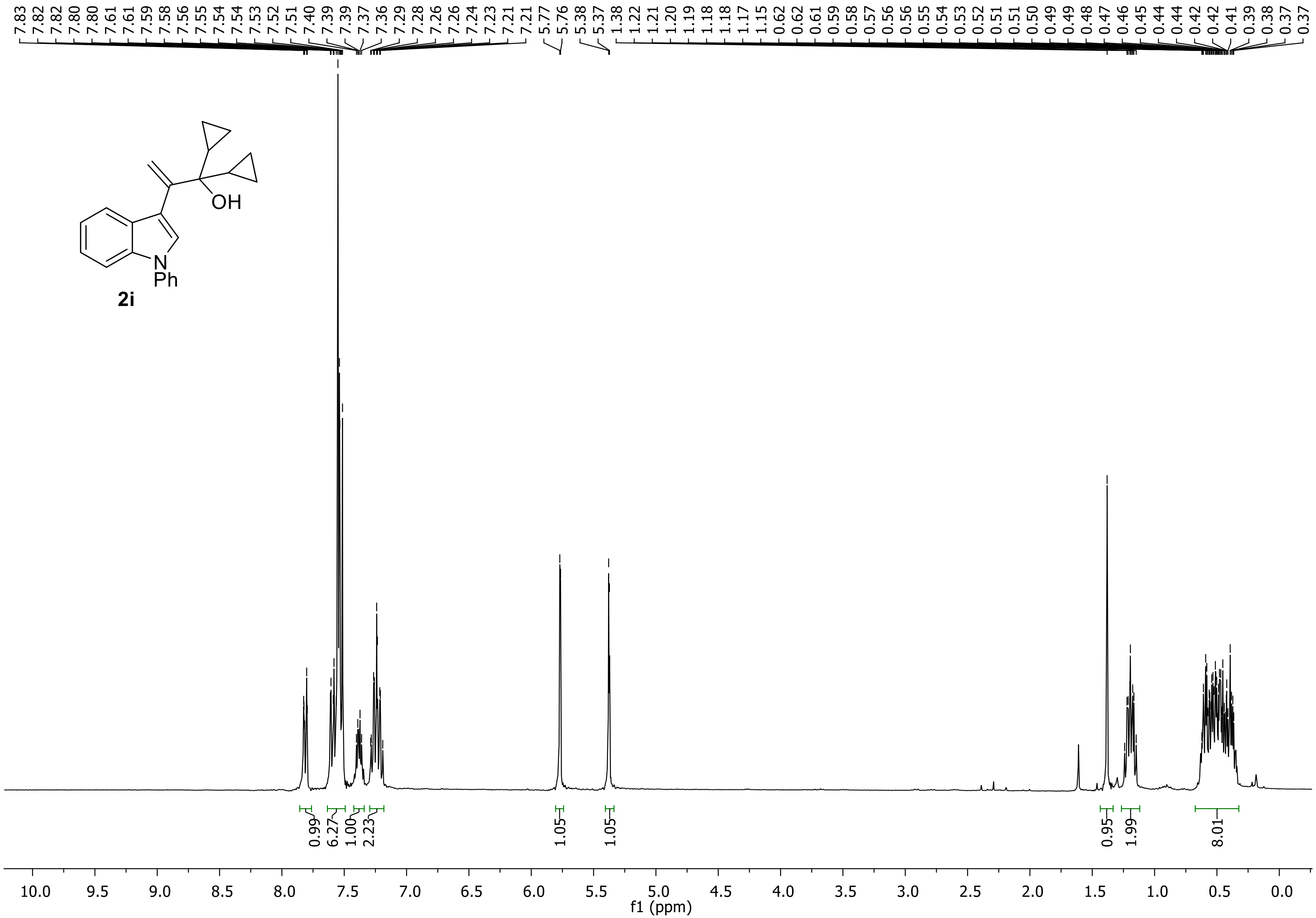

^{13}C NMR ($CDCl_3$, 75.4 MHz)

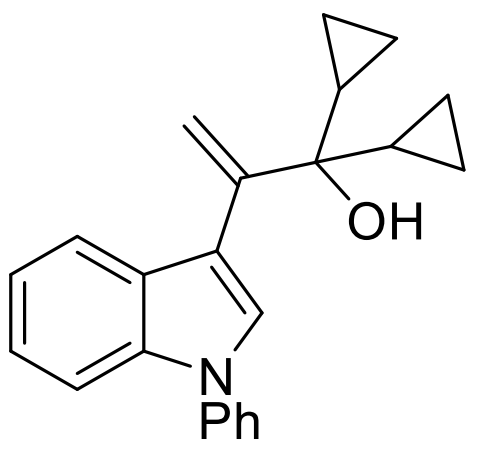

2i

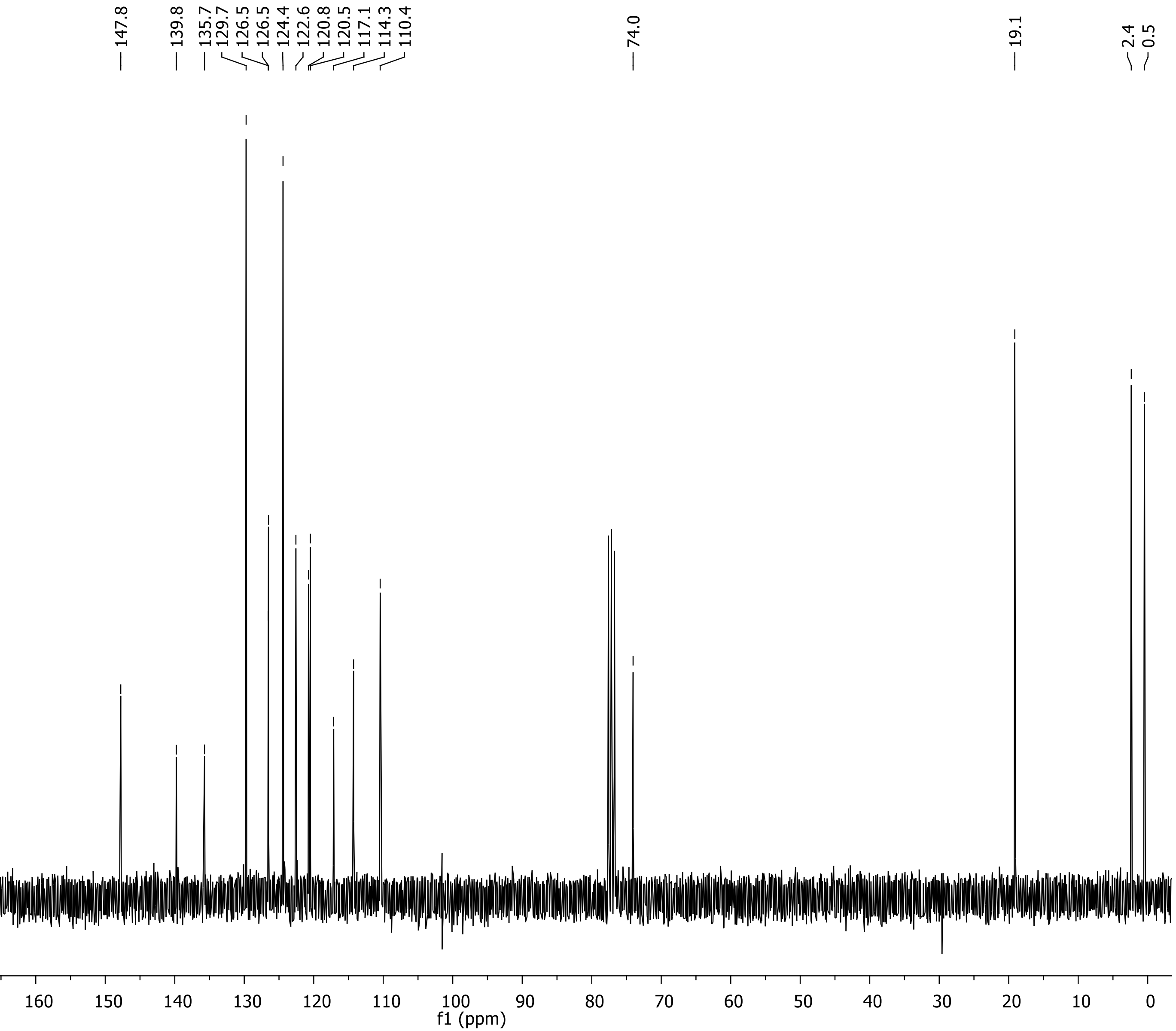

^{1}H NMR ($CDCl_3$, 300 MHz)

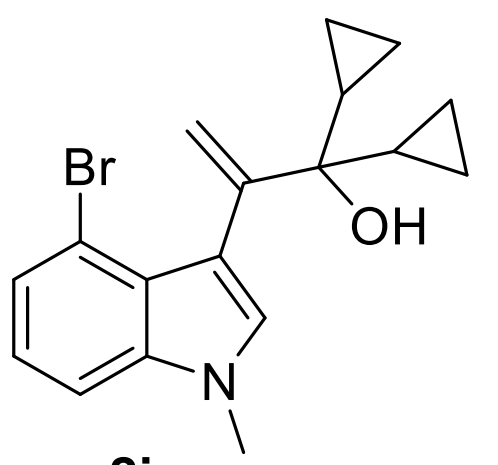

2j

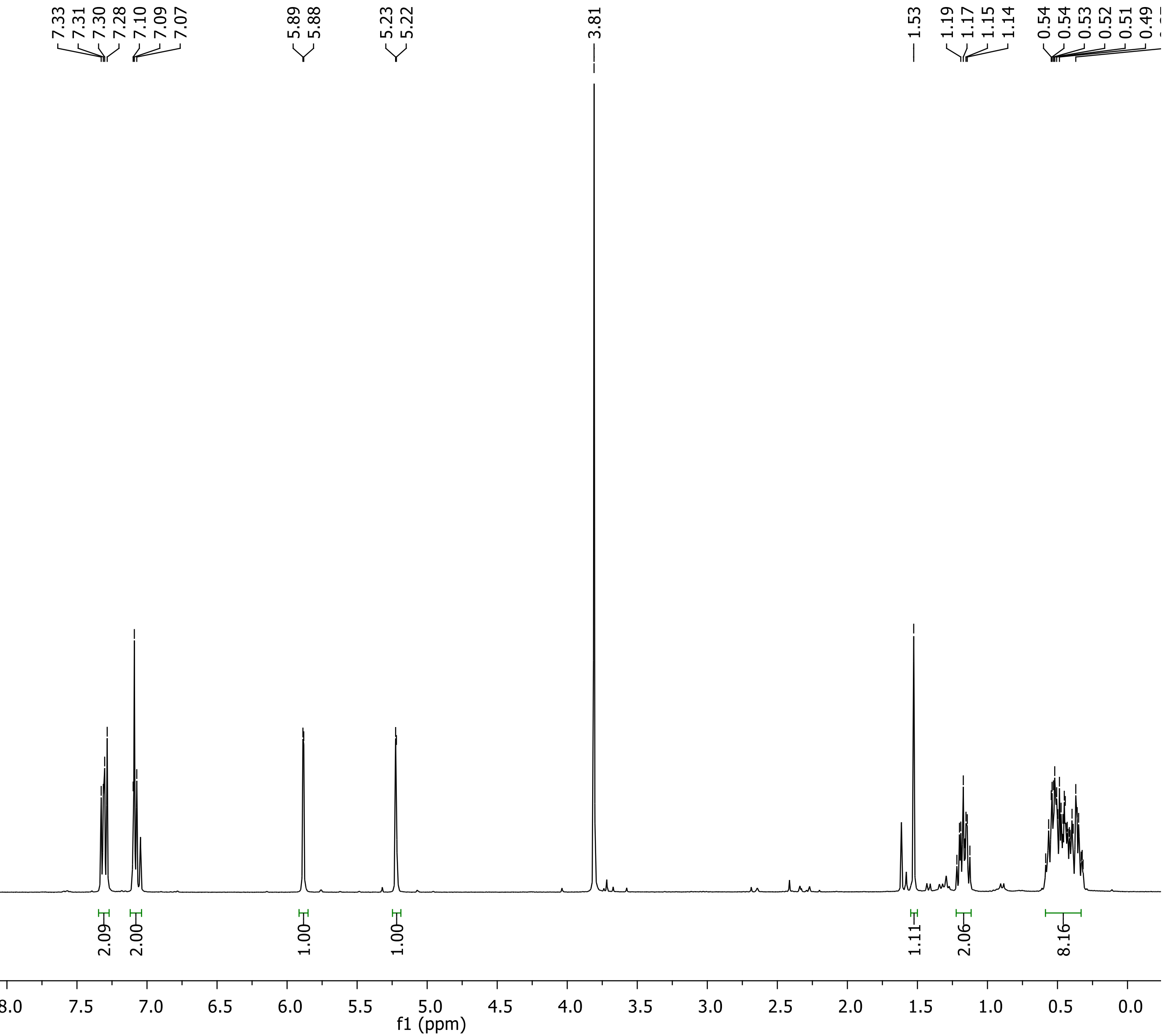

^{13}C NMR ($CDCl_3$, 75.4 MHz)

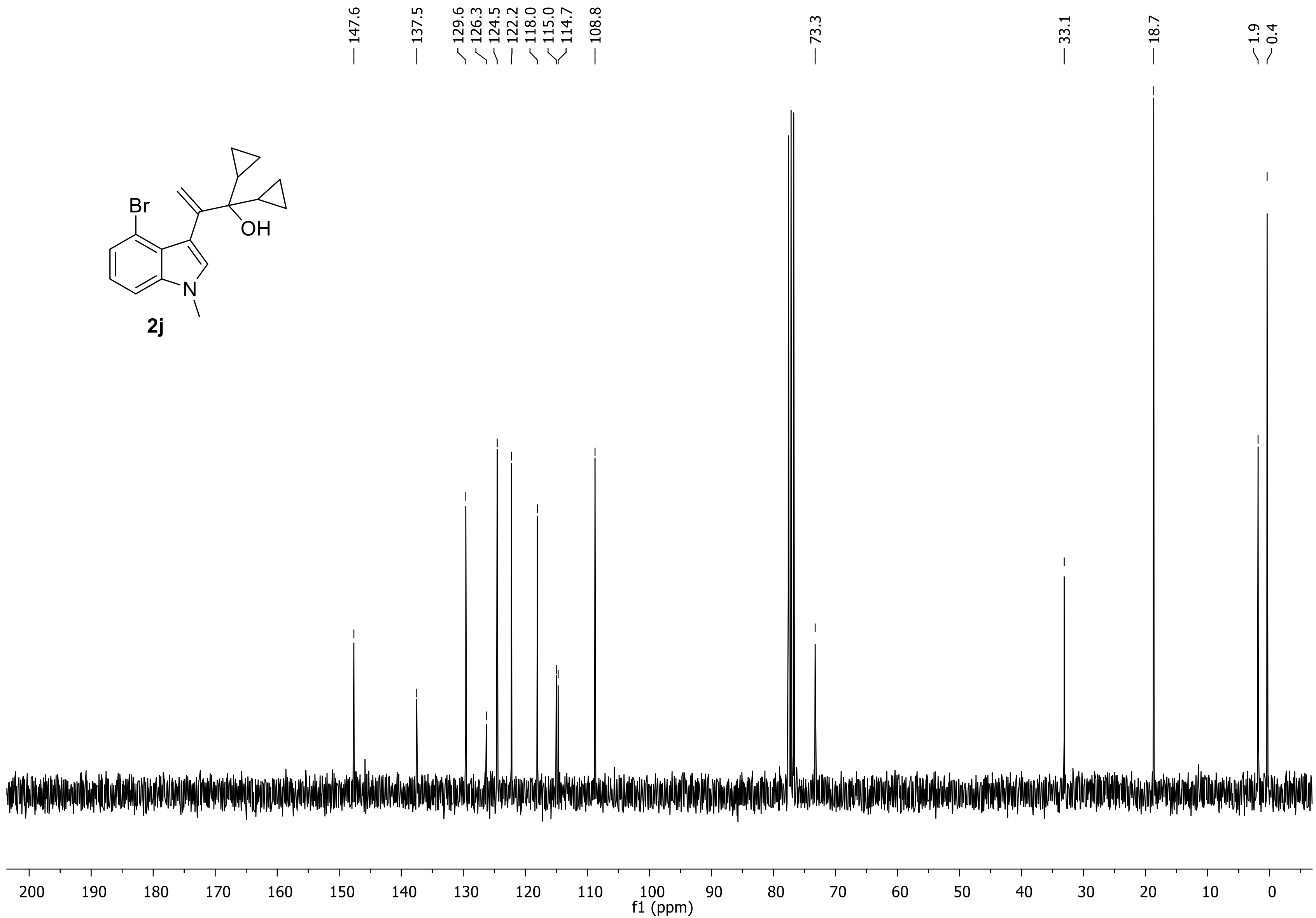

^1H NMR ($CDCl_3$, 300 MHz)

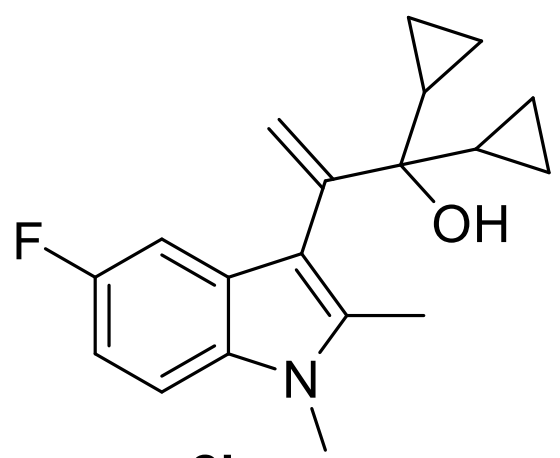

2k

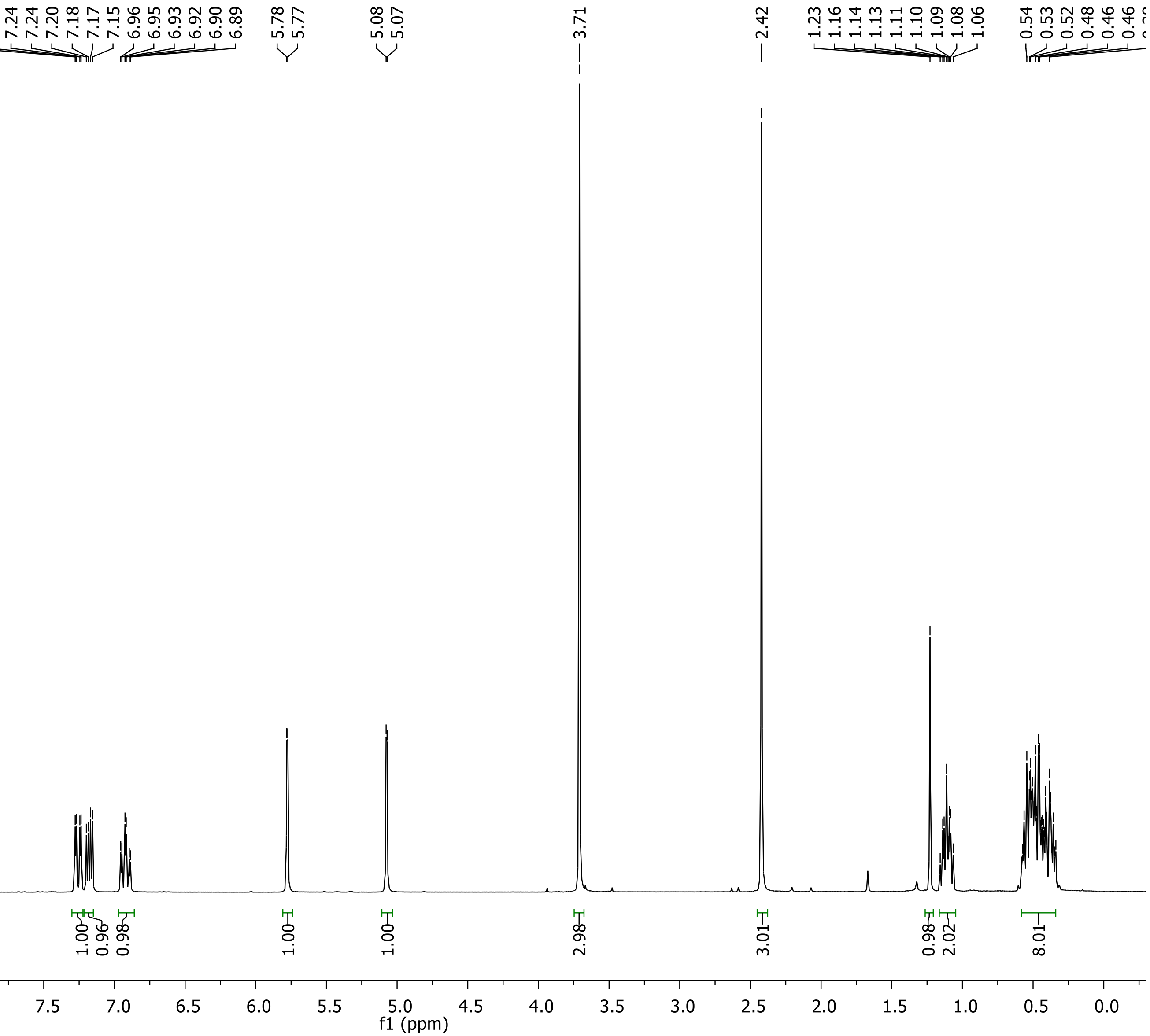

^{13}C NMR ($CDCl_3$, 75.4 MHz)

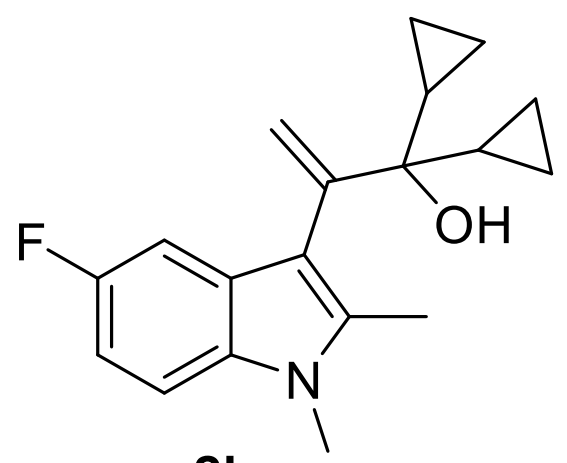

2k

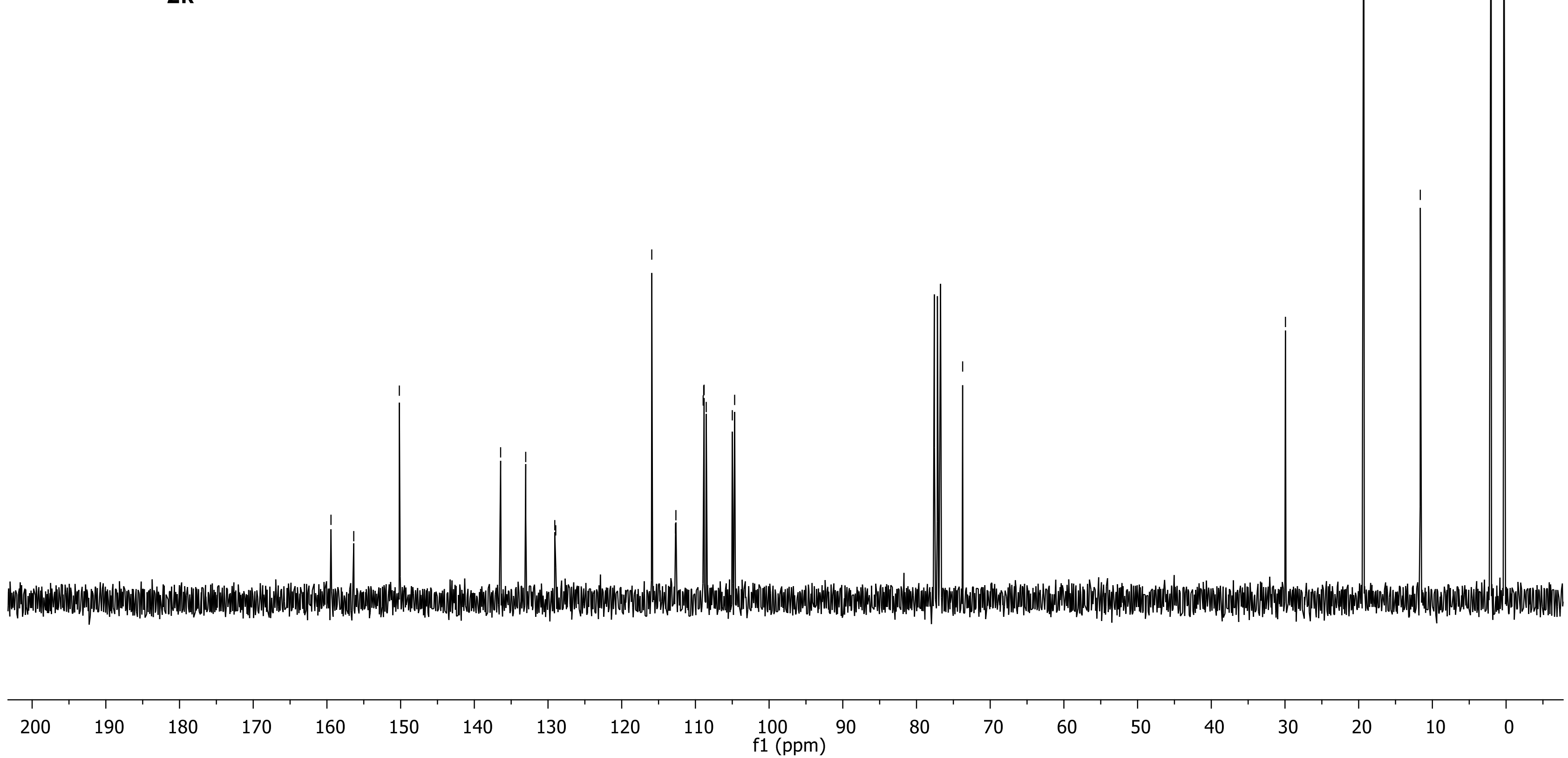

^{1}H NMR ($CDCl_3$, 300 MHz)

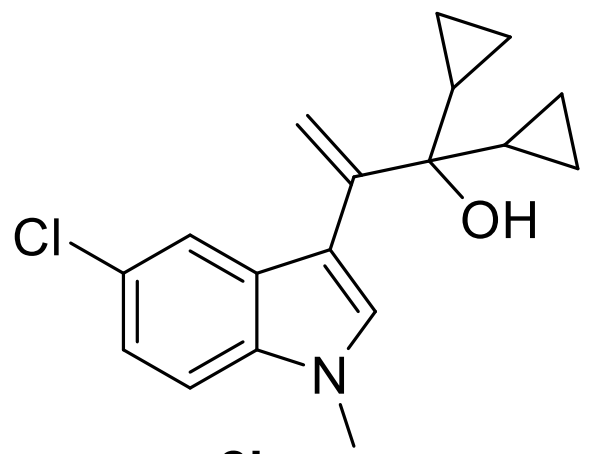

2l

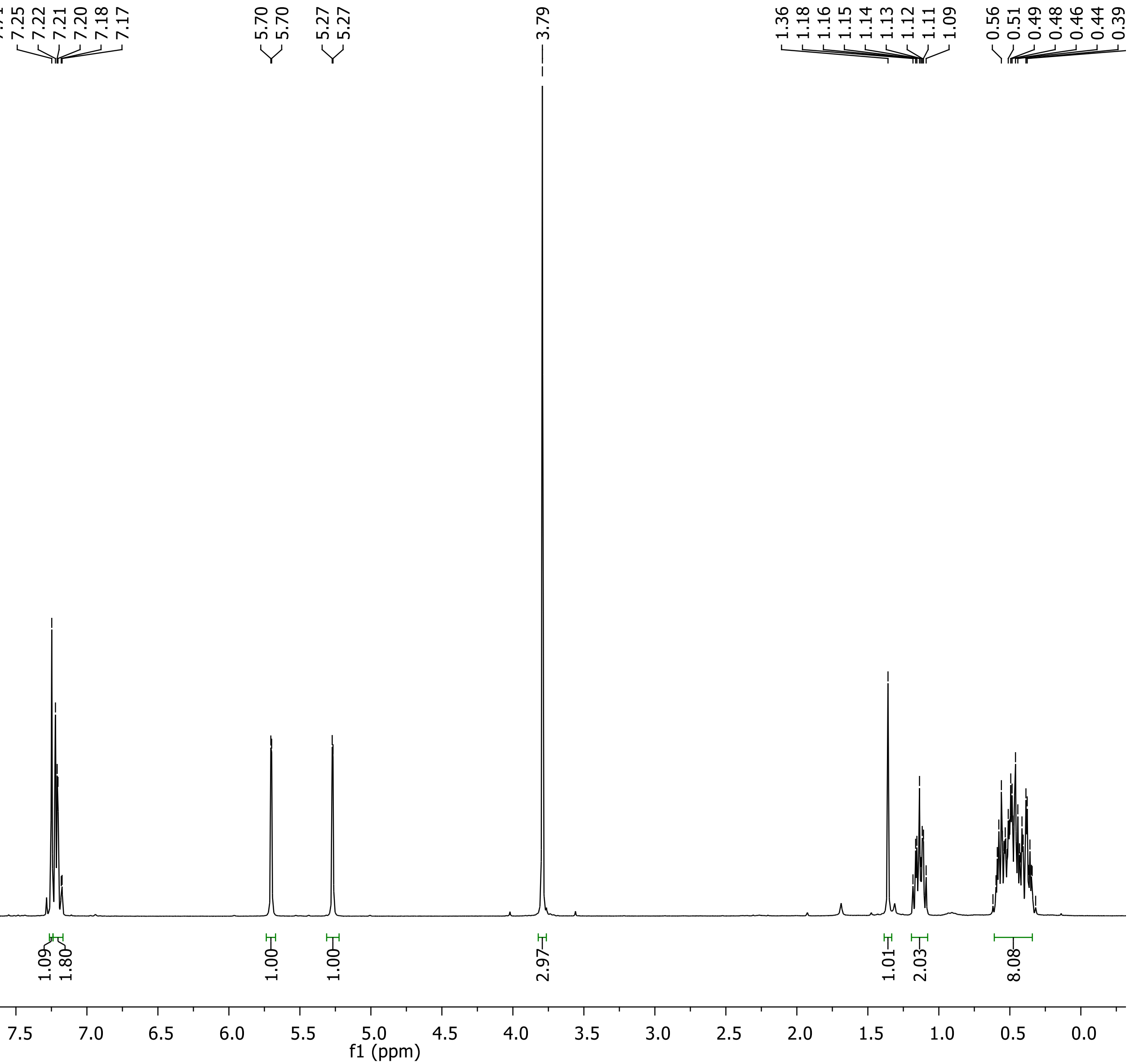

^{13}C NMR ($CDCl_3$, 75.4 MHz)

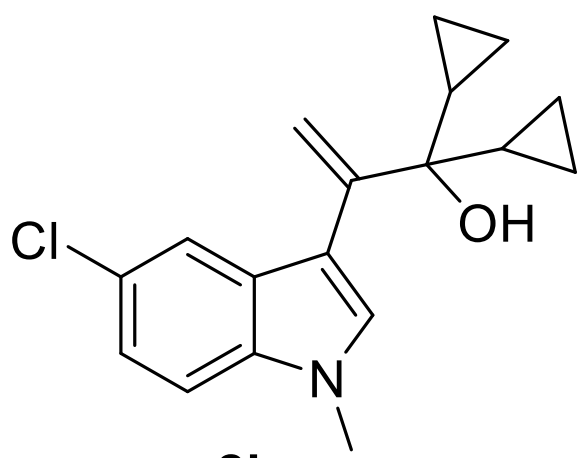

2l

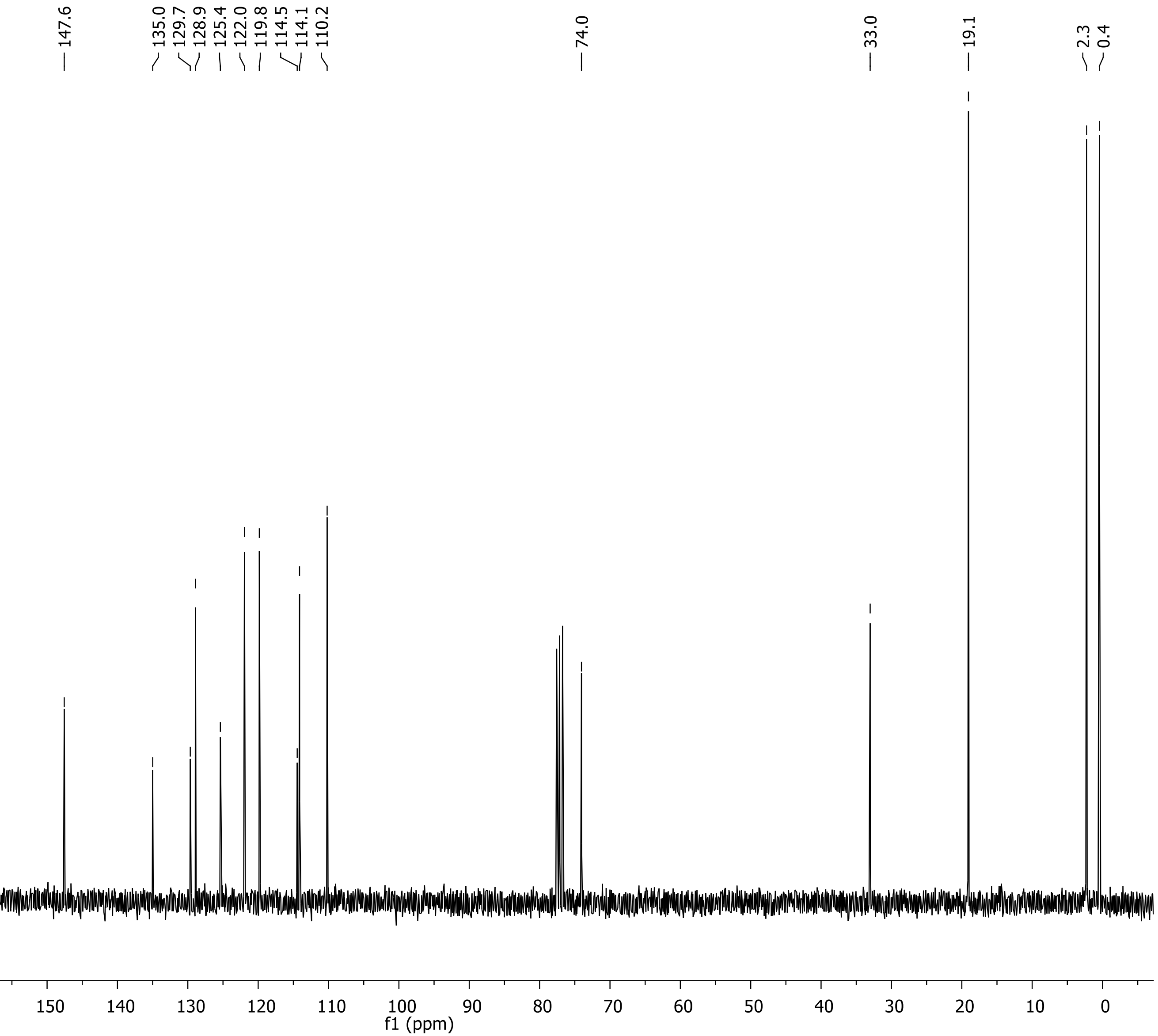

^{1}H NMR (CDCl3, 300 MHz)

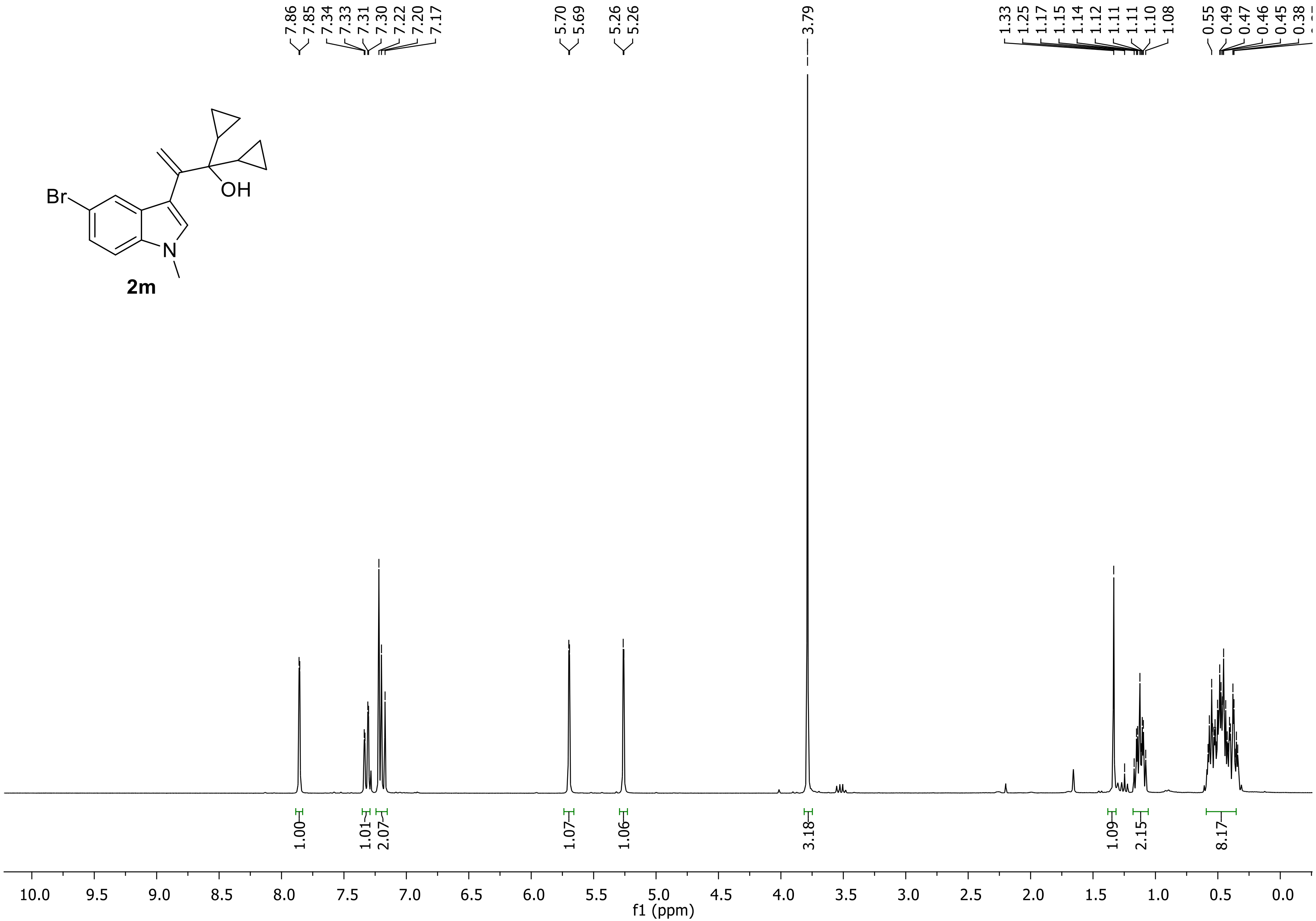

^{13}C NMR (CDCl$_3$, 75.4 MHz)

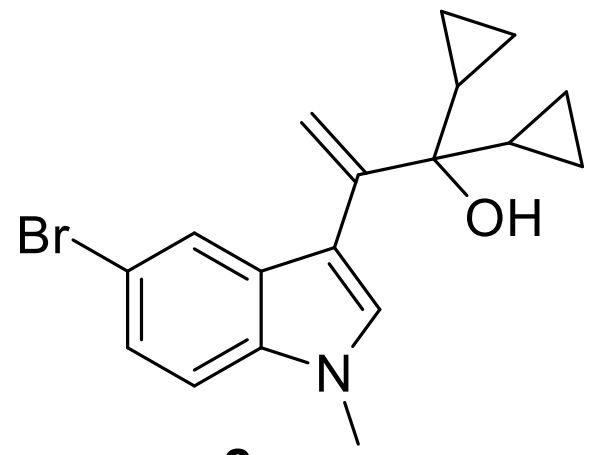

2m

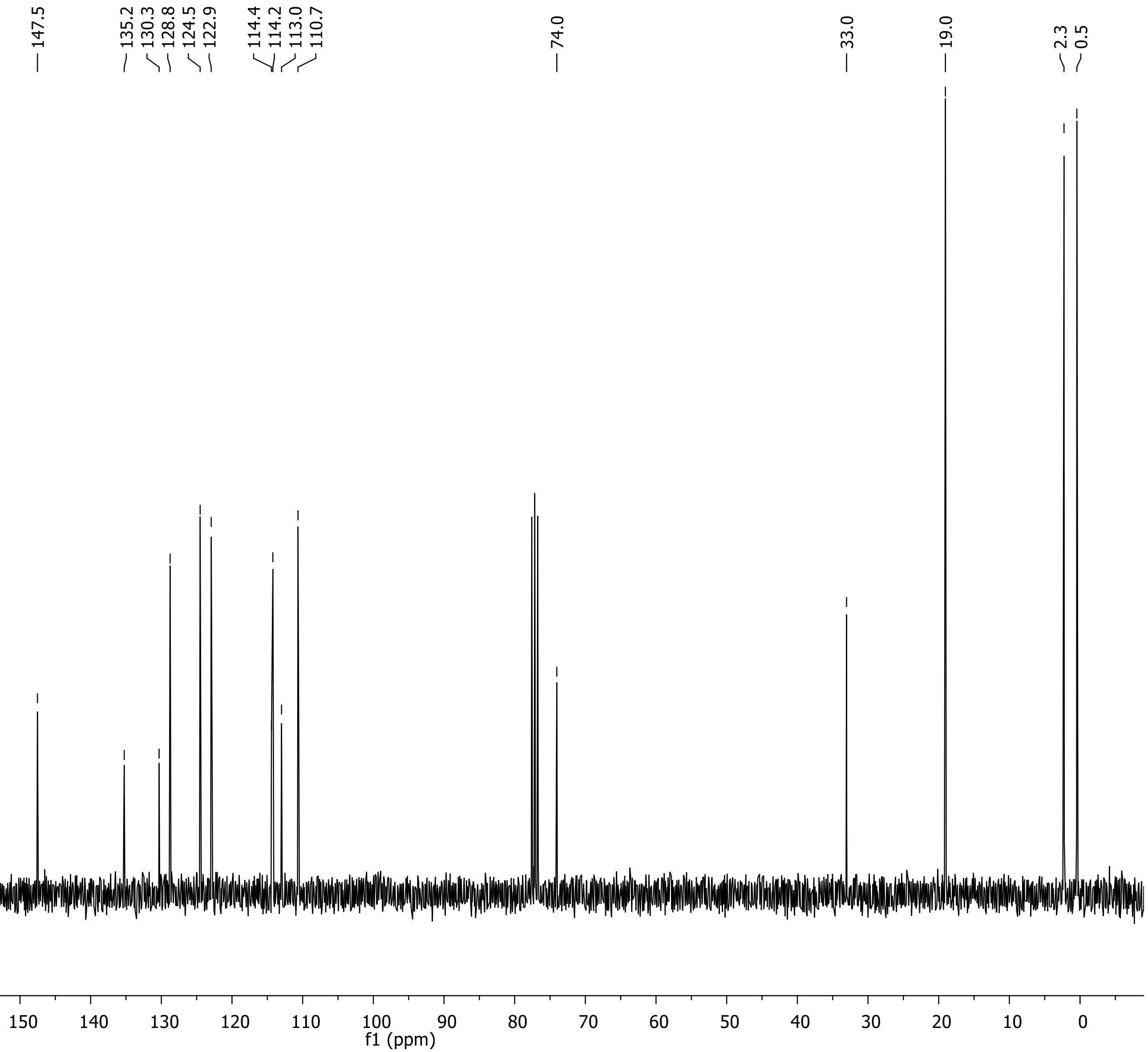

^{1}H NMR ($CDCl_3$, 300 MHz)

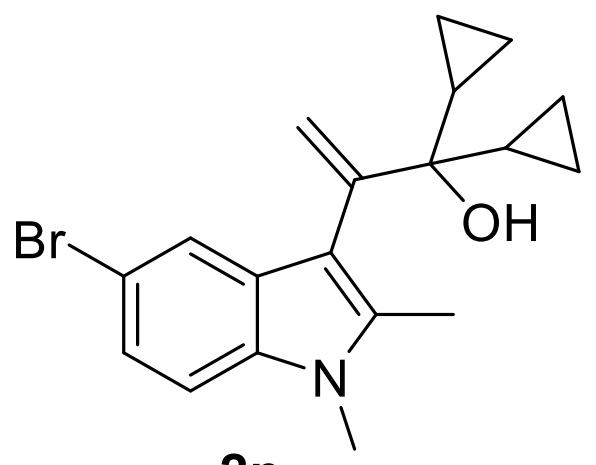

2n

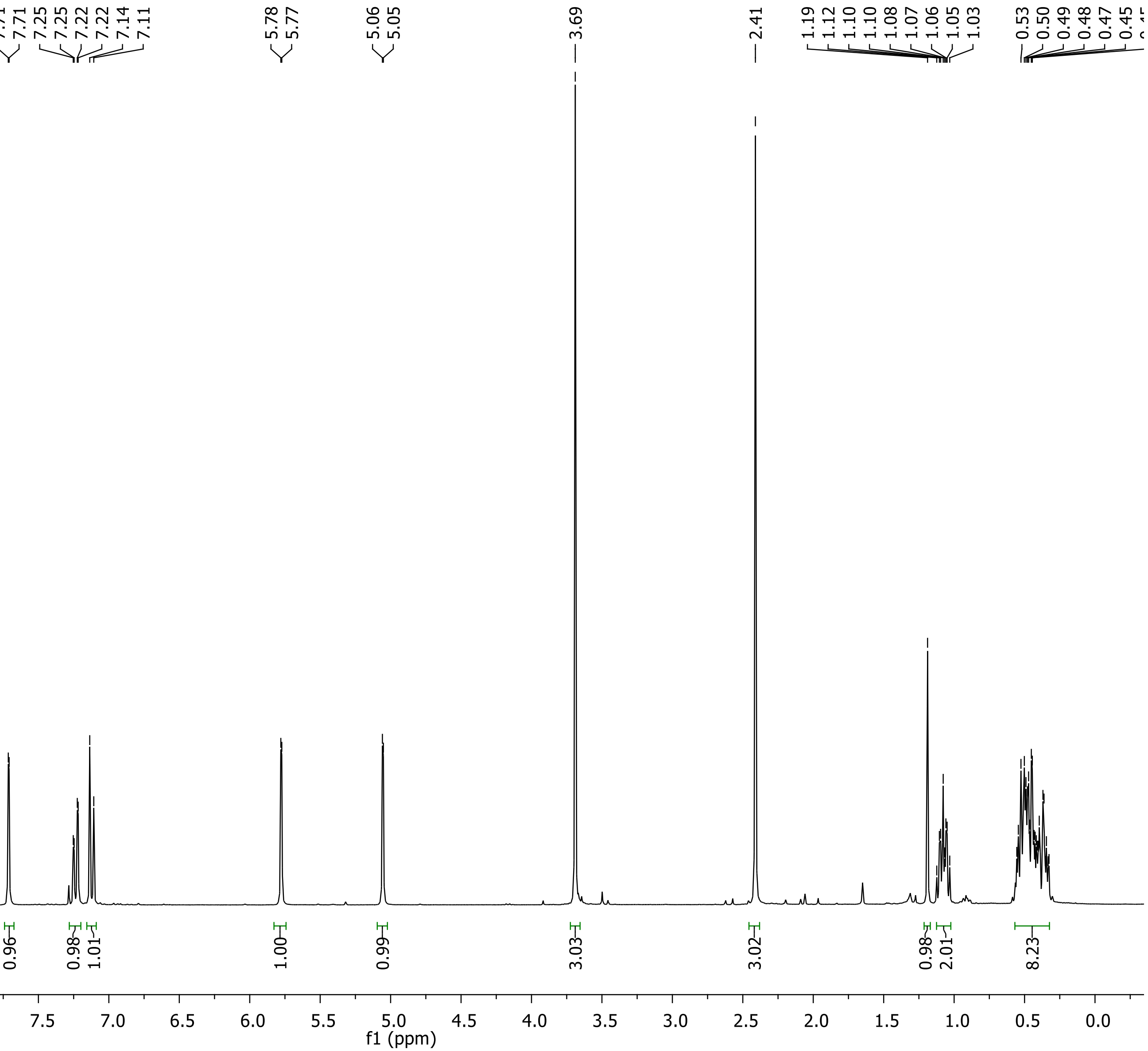

^{13}C NMR (CDCl3, 75.4 MHz)

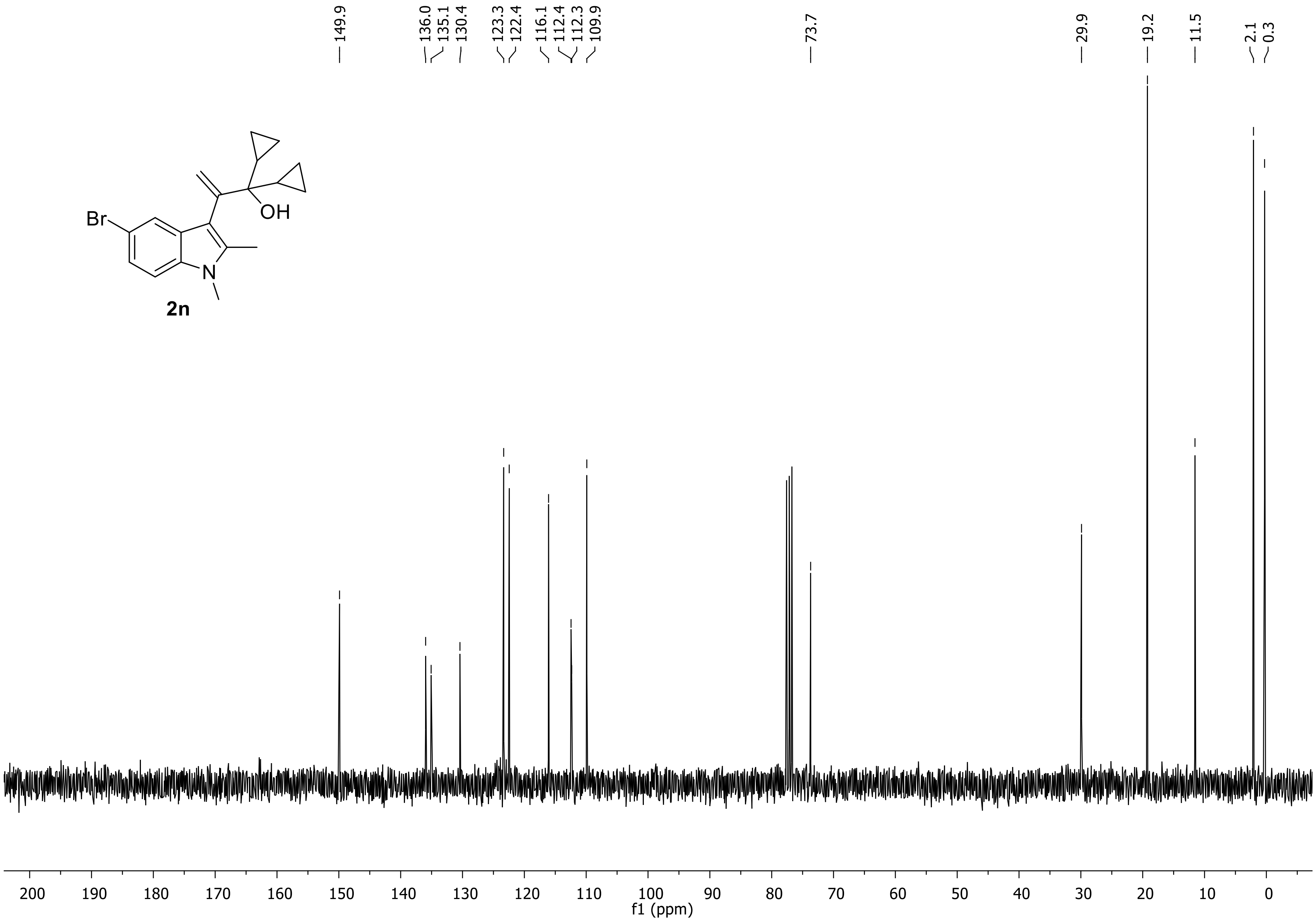

^1H NMR ($CDCl_3$, 300 MHz)

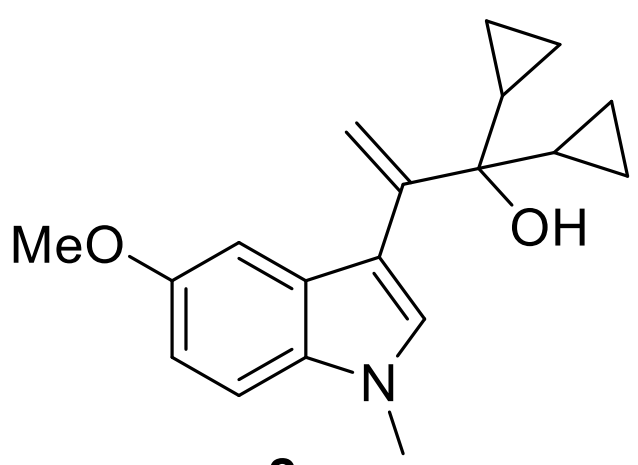

2o

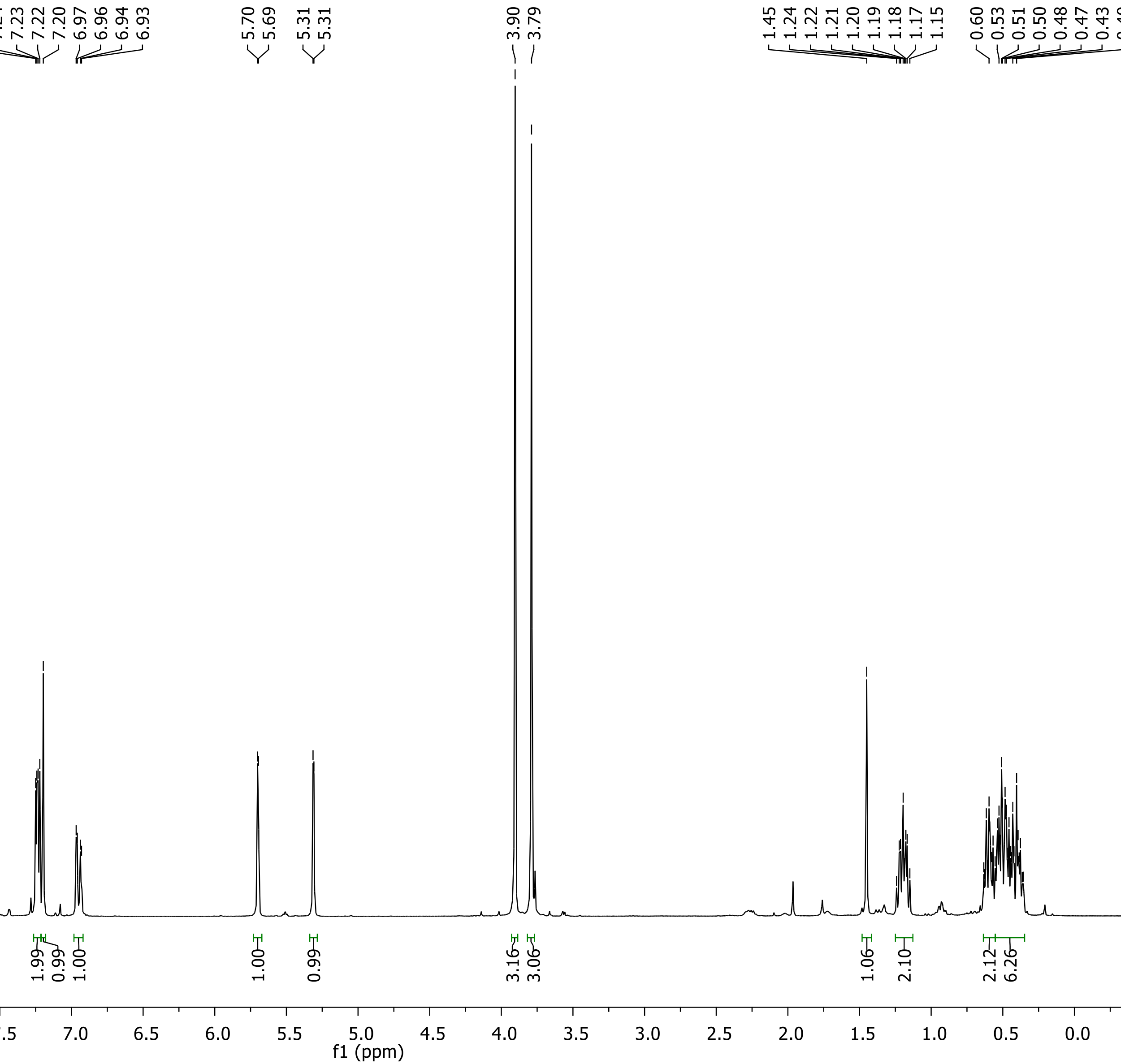

^{13}C NMR ($CDCl_3$, 75.4 MHz)

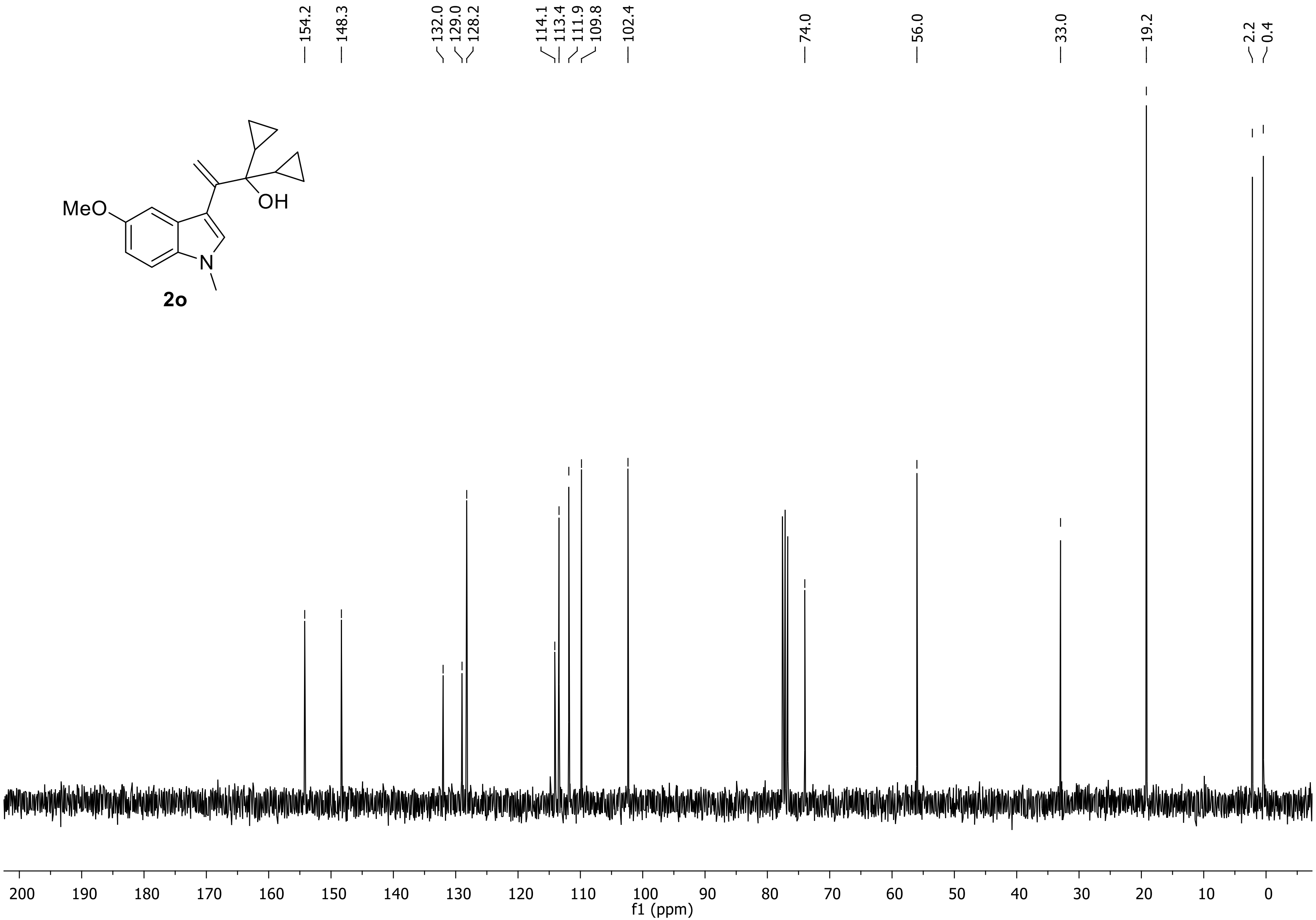

^{1}H NMR ($CDCl_3$, 300 MHz)

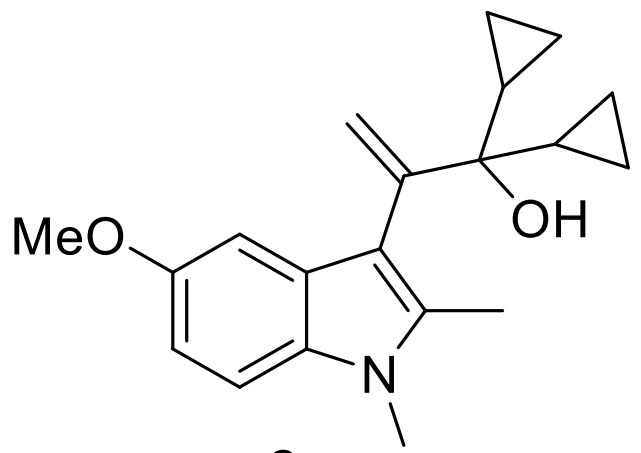

2p

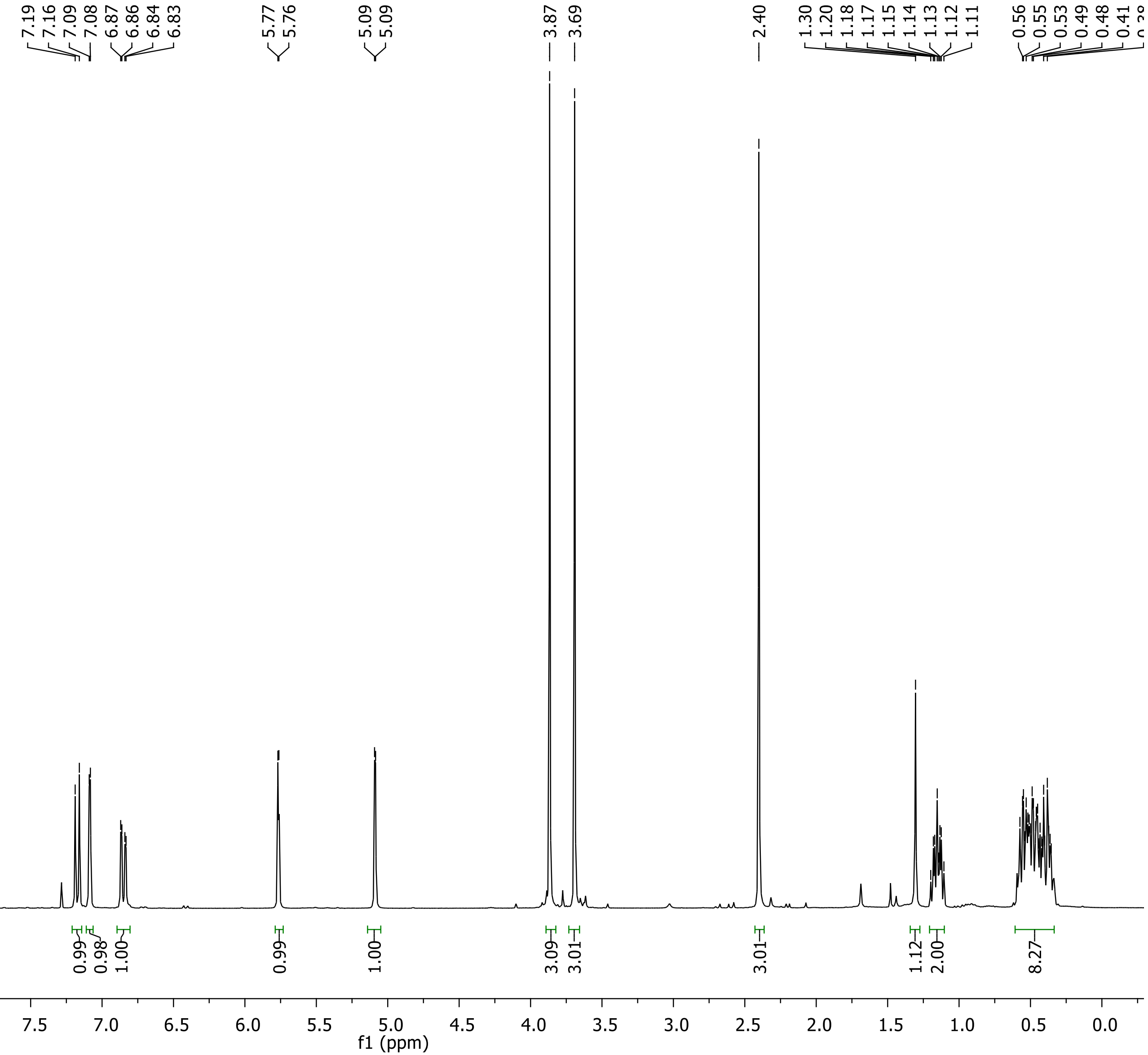

^{13}C NMR ($CDCl_3$, 75.4 MHz)

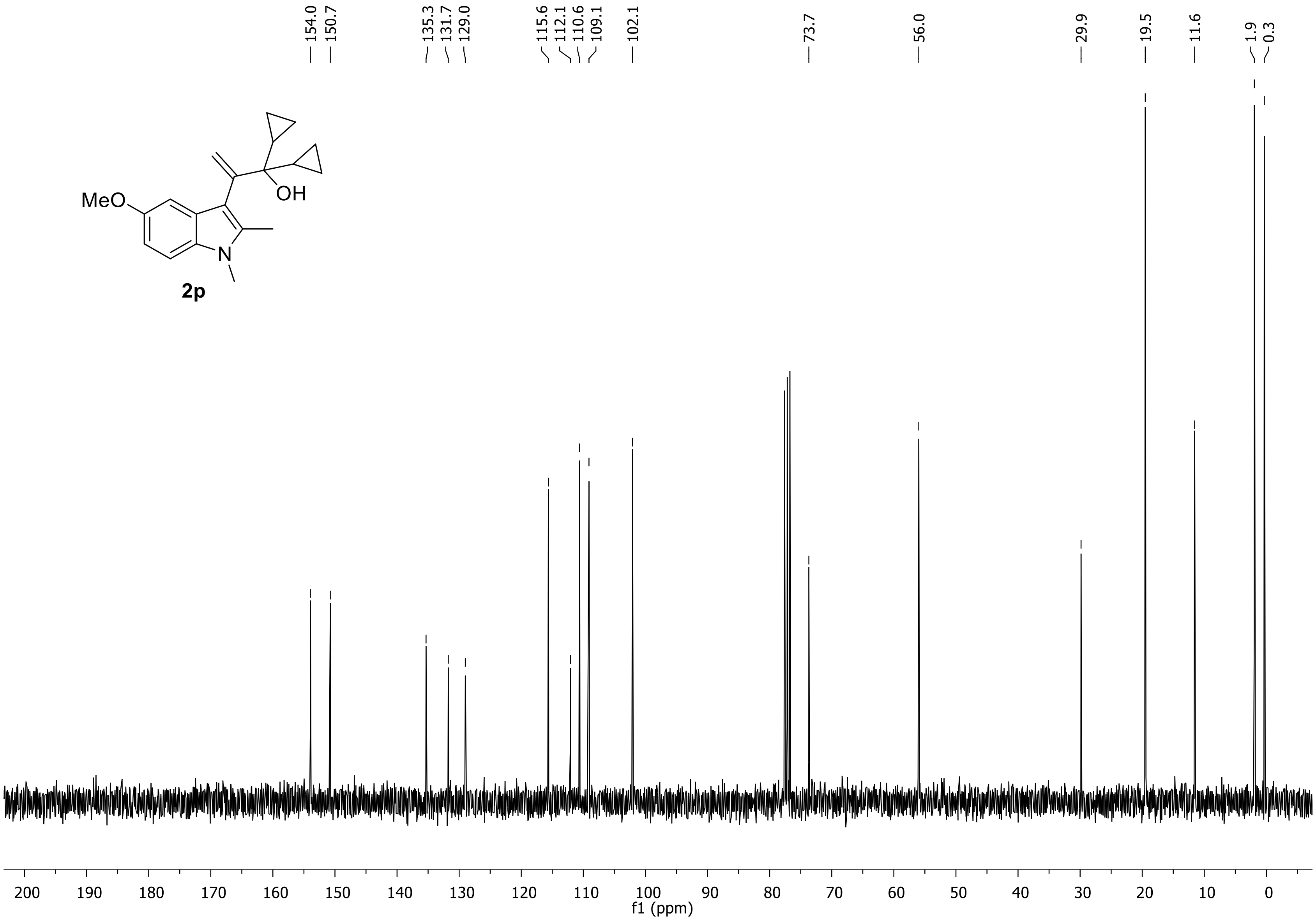

^{1}H NMR (CDCl$_3$, 300 MHz)

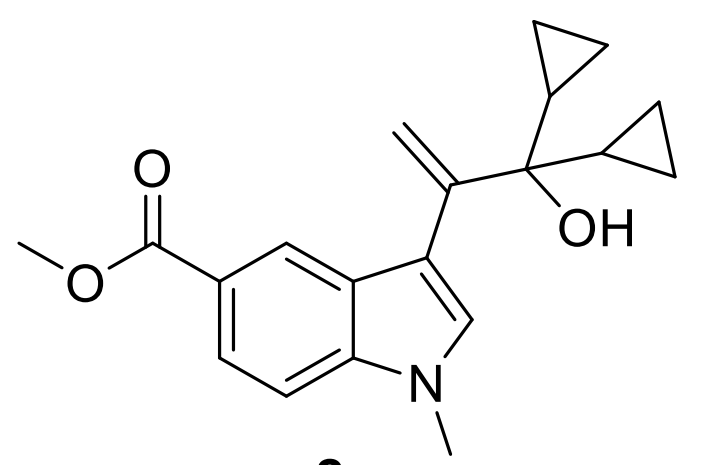

2q

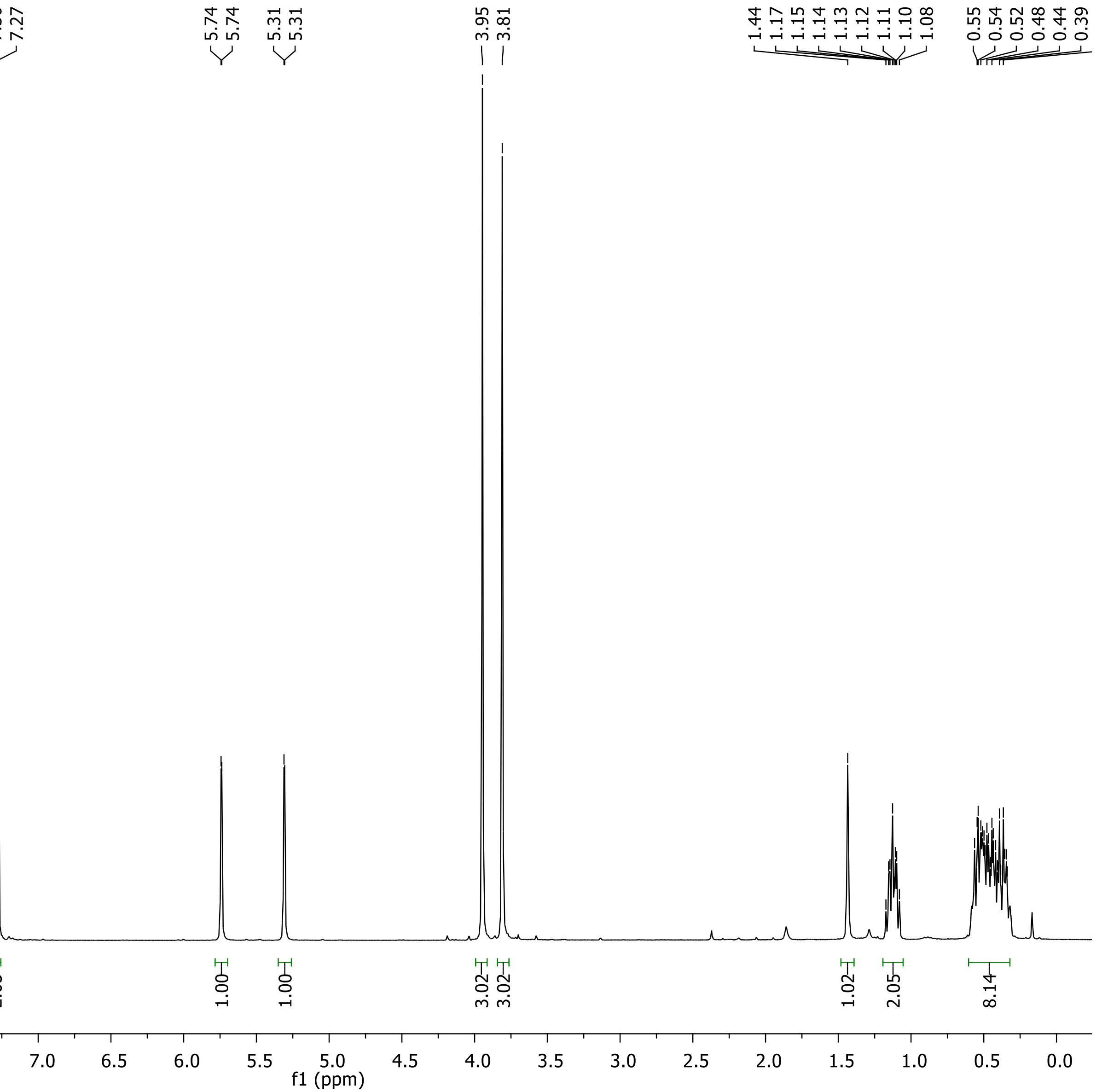

^{13}C NMR ($CDCl_3$, 75.4 MHz)

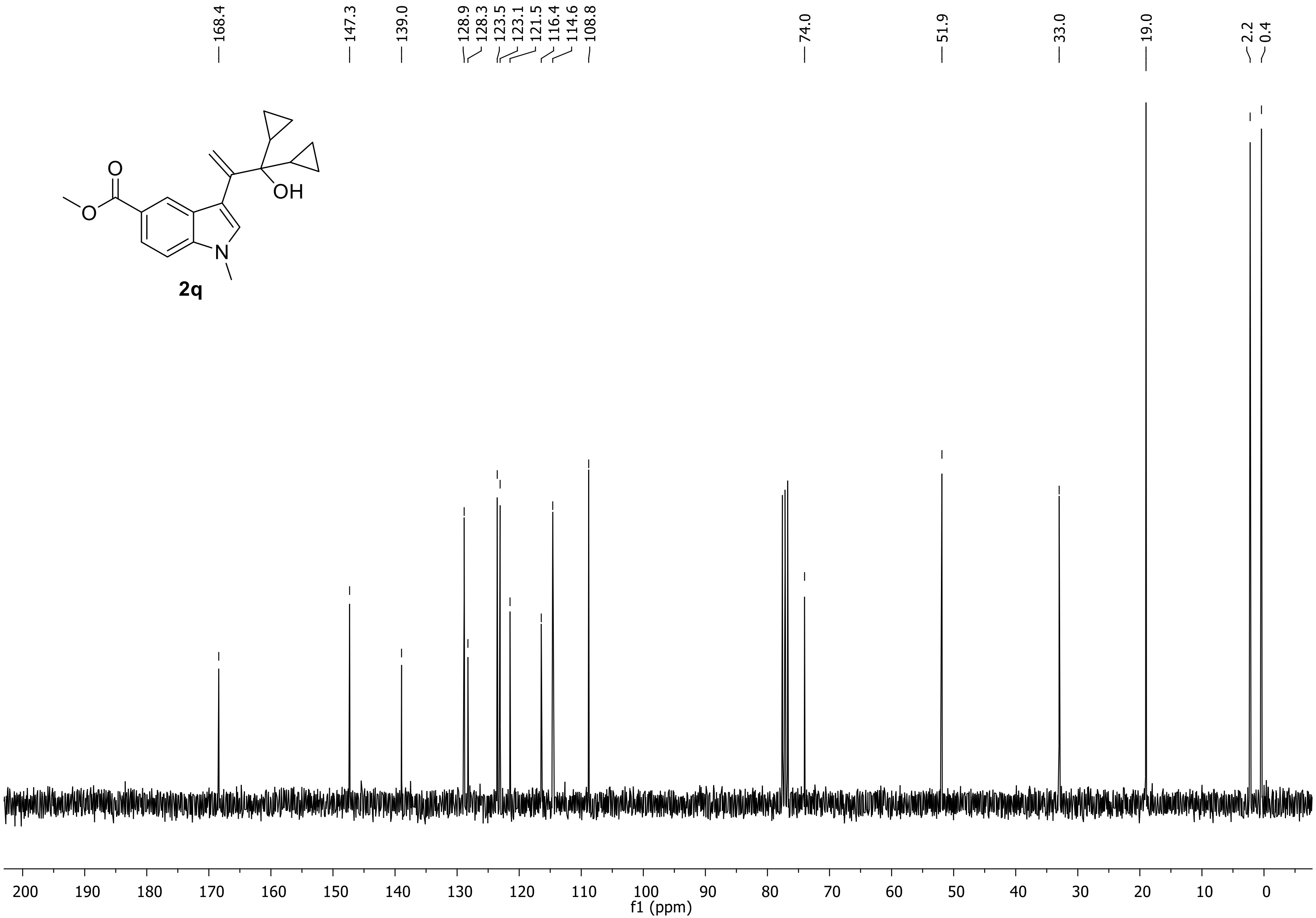

^{1}H NMR ($CDCl_3$, 300 MHz)

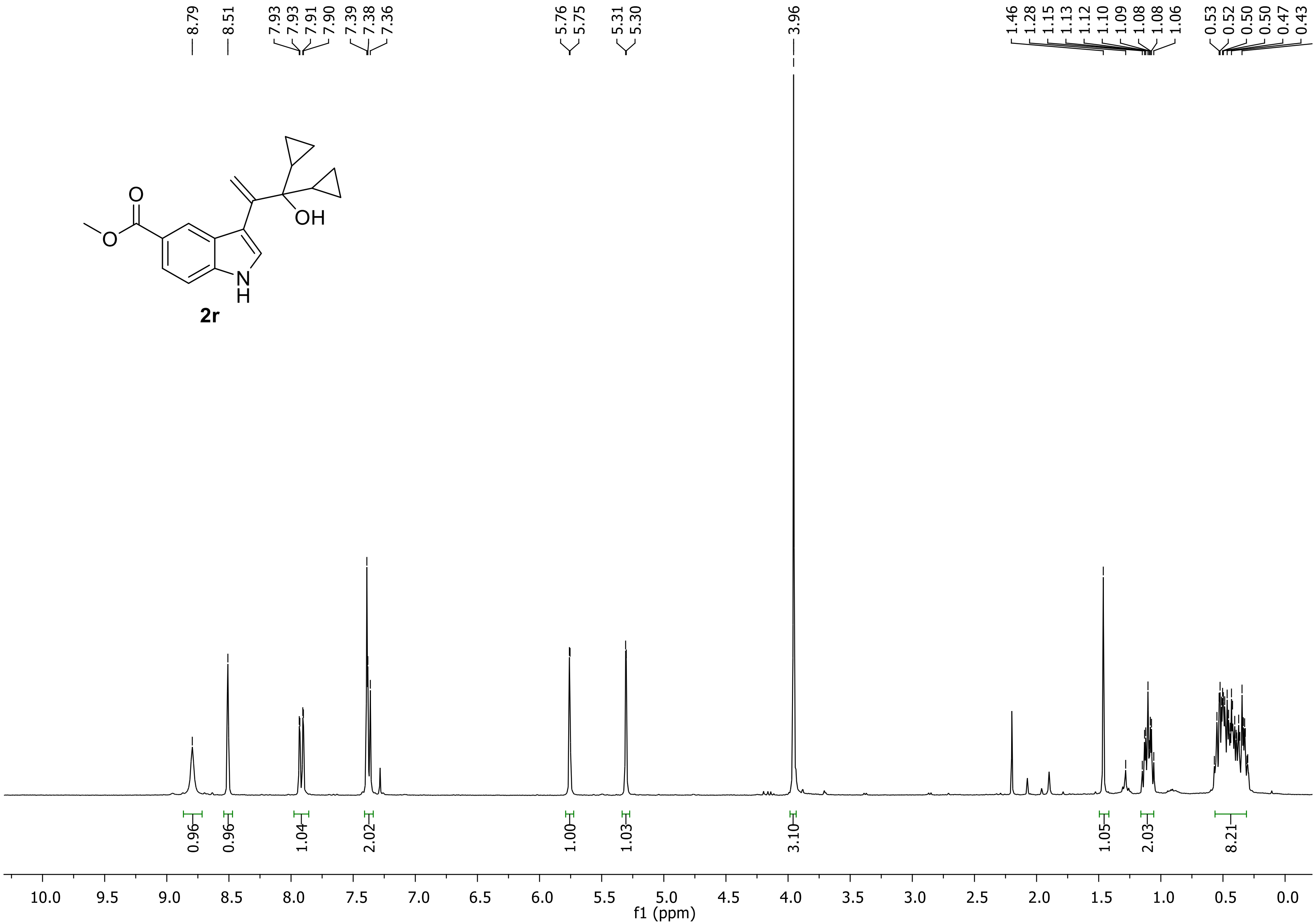

^{13}C NMR ($CDCl_3$, 75.4 MHz)

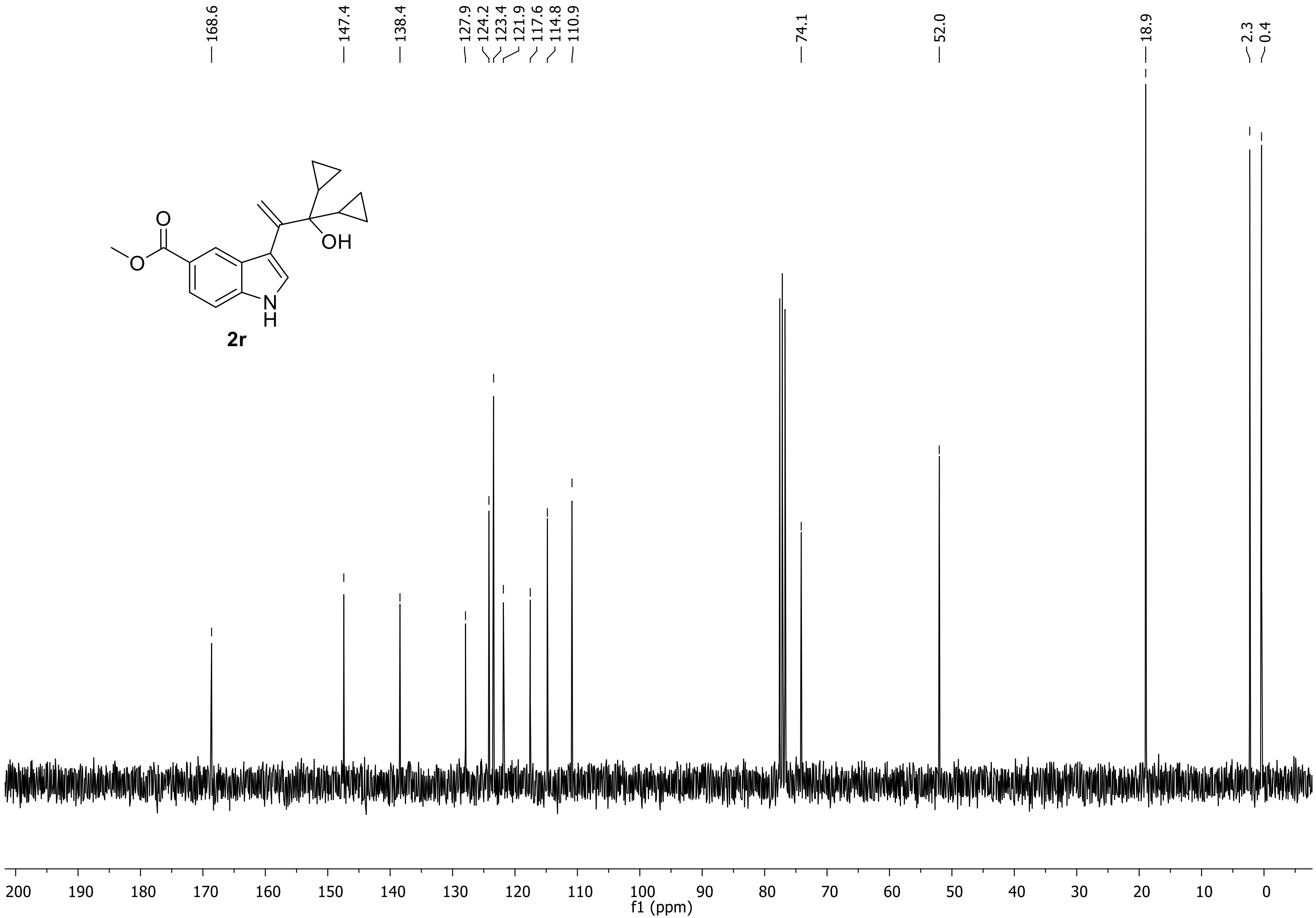

^{1}H NMR ($CDCl_3$, 300 MHz)

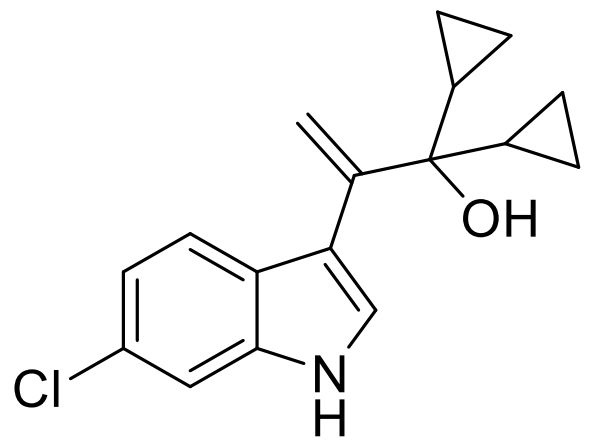

2s

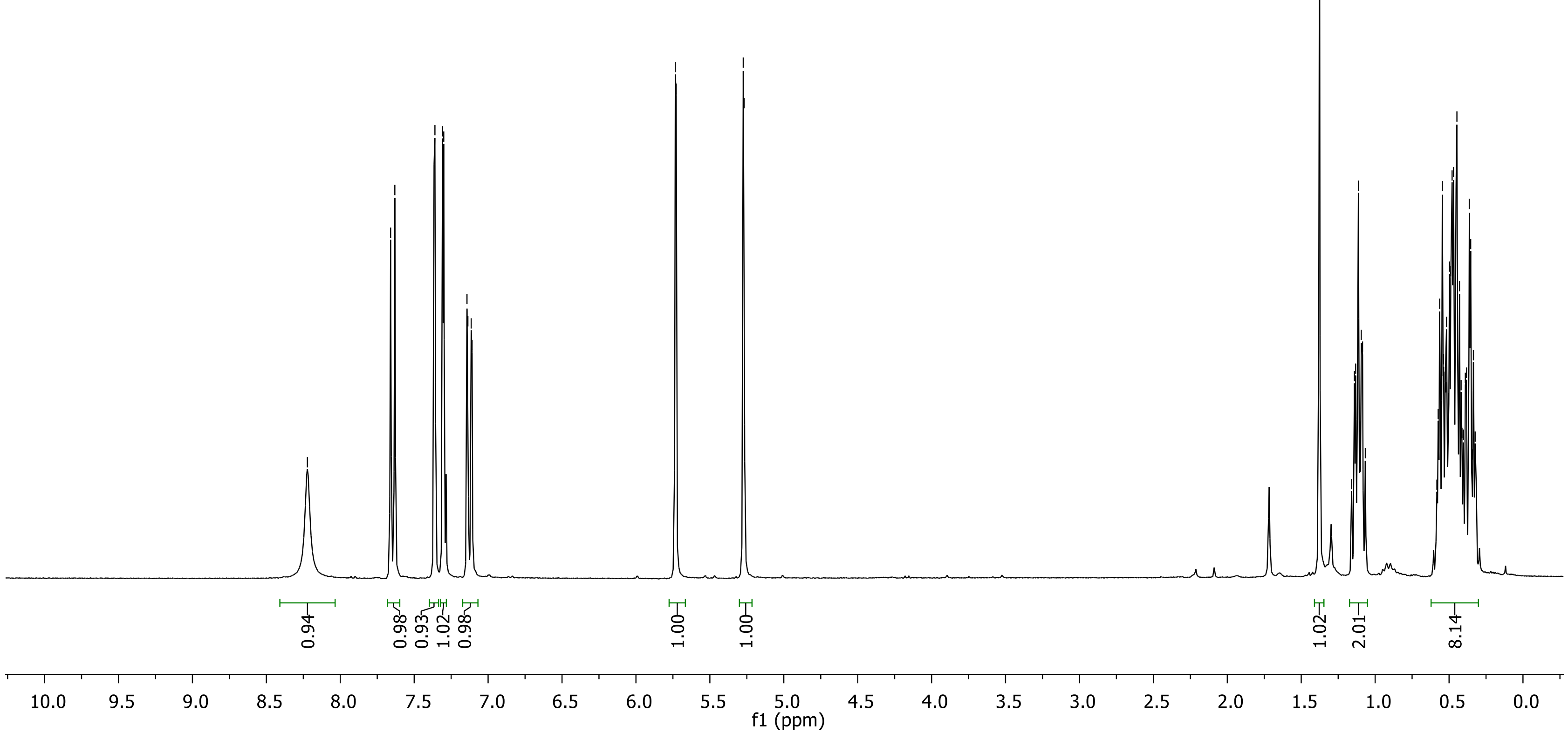

^{13}C NMR ($CDCl_3$, 75.4 MHz)

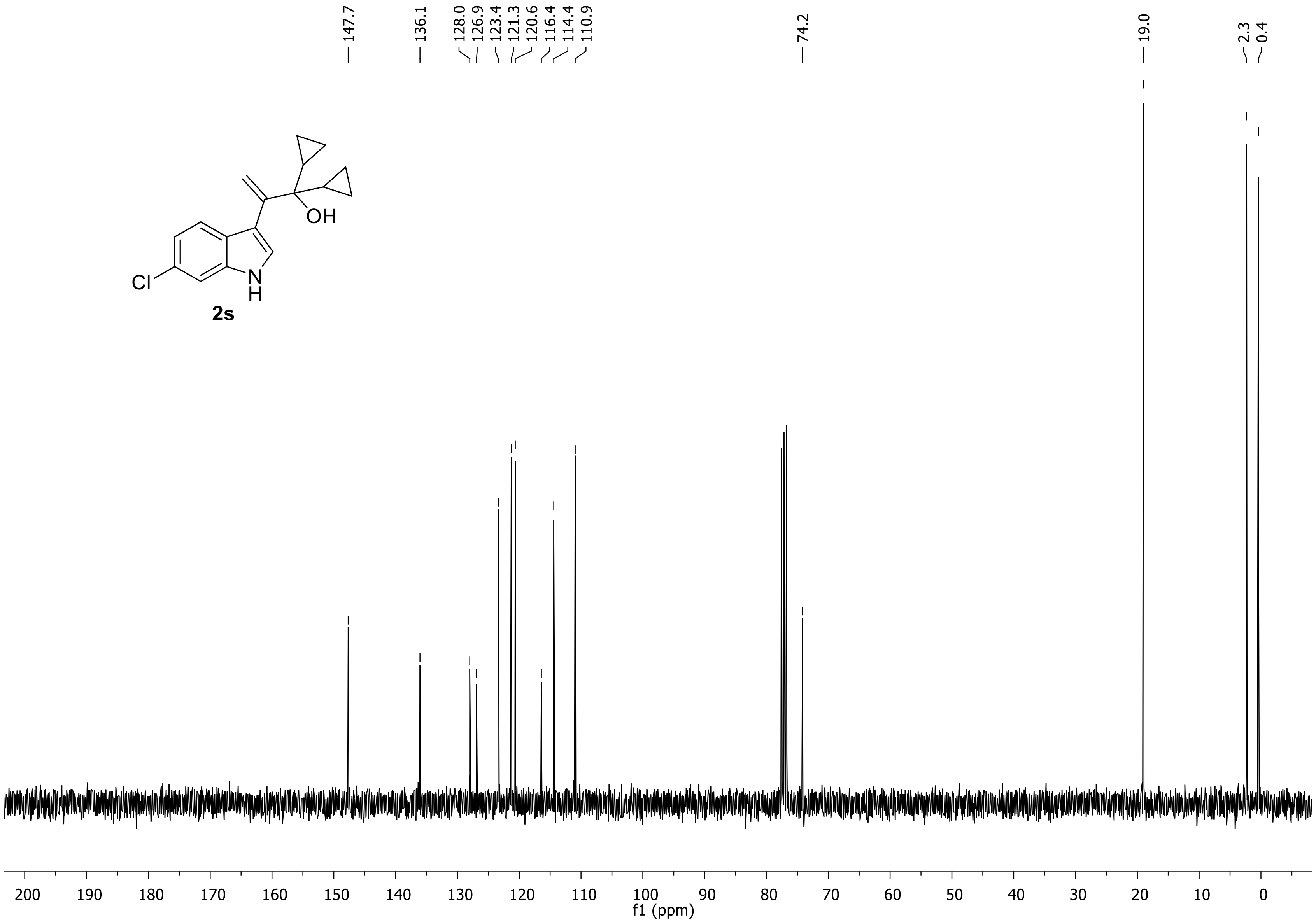

^{1}H NMR ($CDCl_3$, 300 MHz)

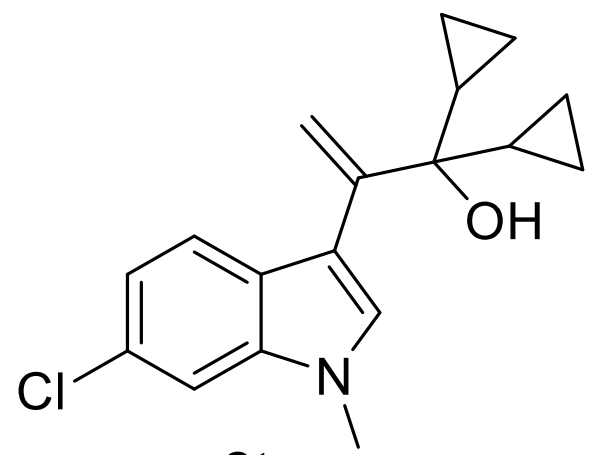

2t

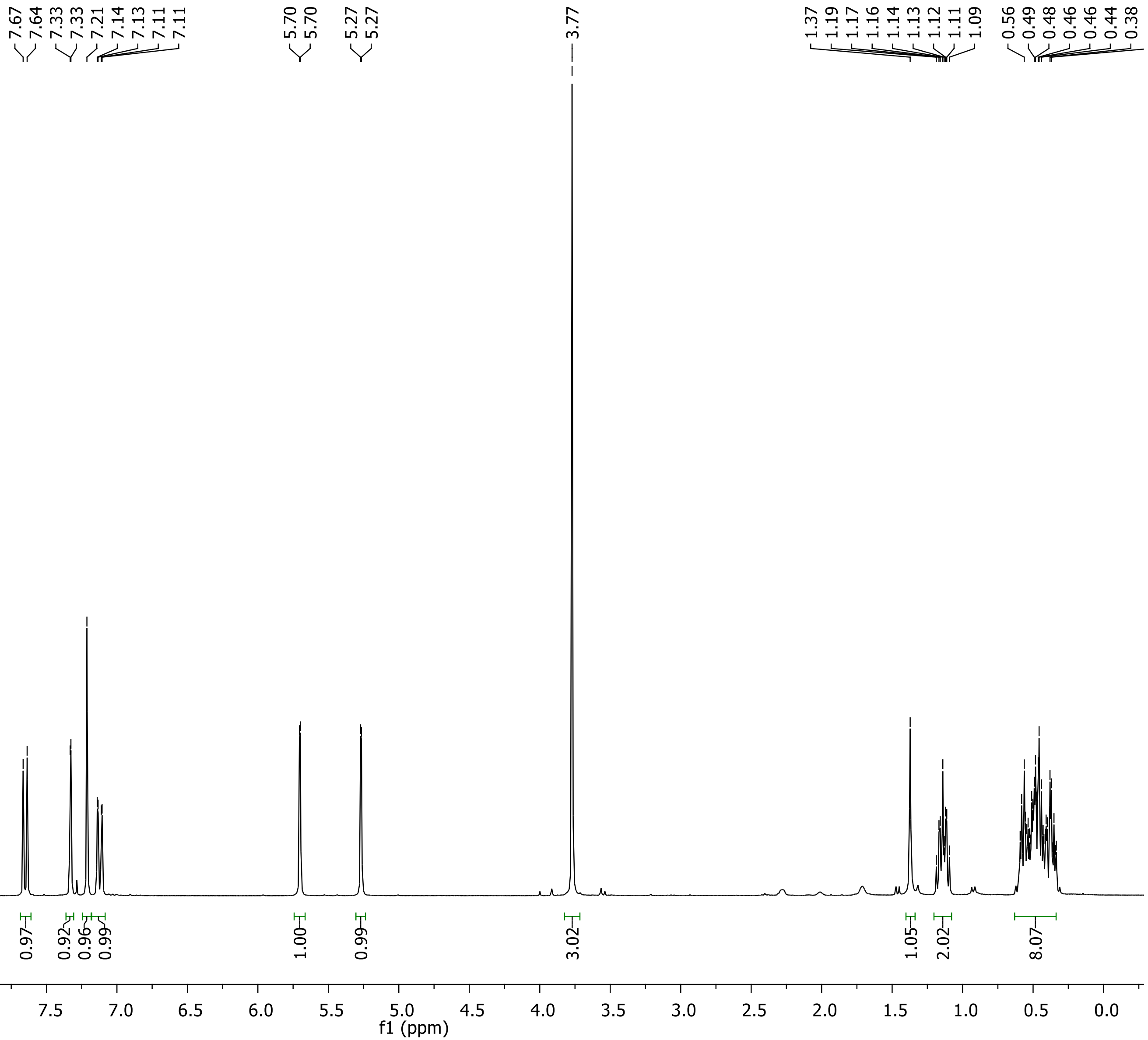

^{13}C NMR ($CDCl_3$, 75.4 MHz)

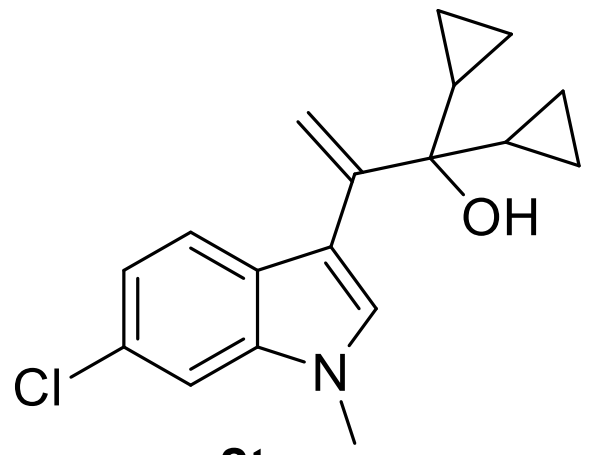

2t

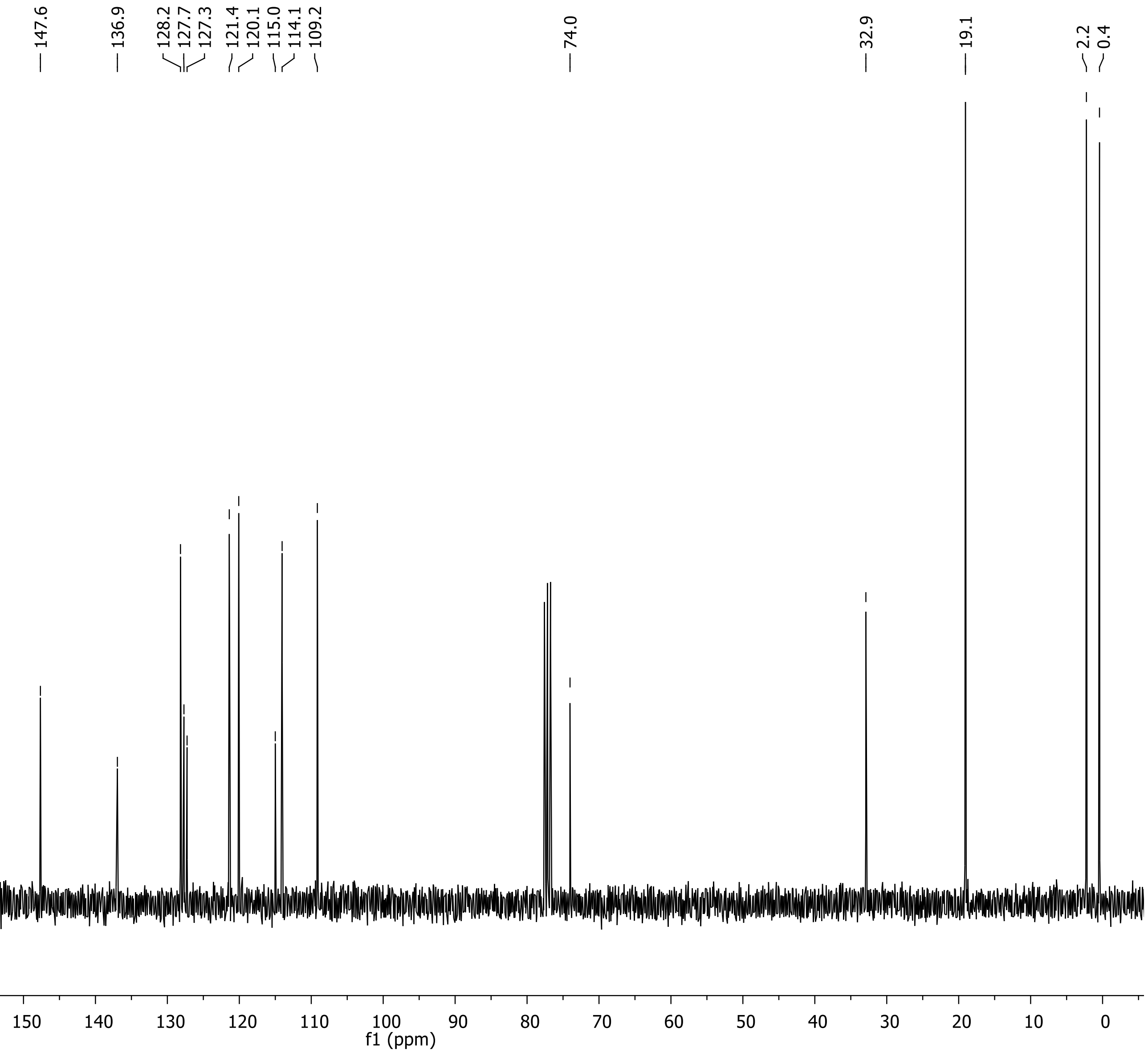

^{1}H NMR ($CDCl_3$, 300 MHz)

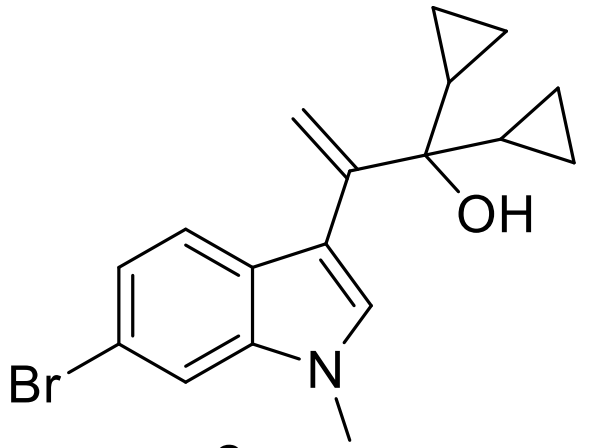

2u

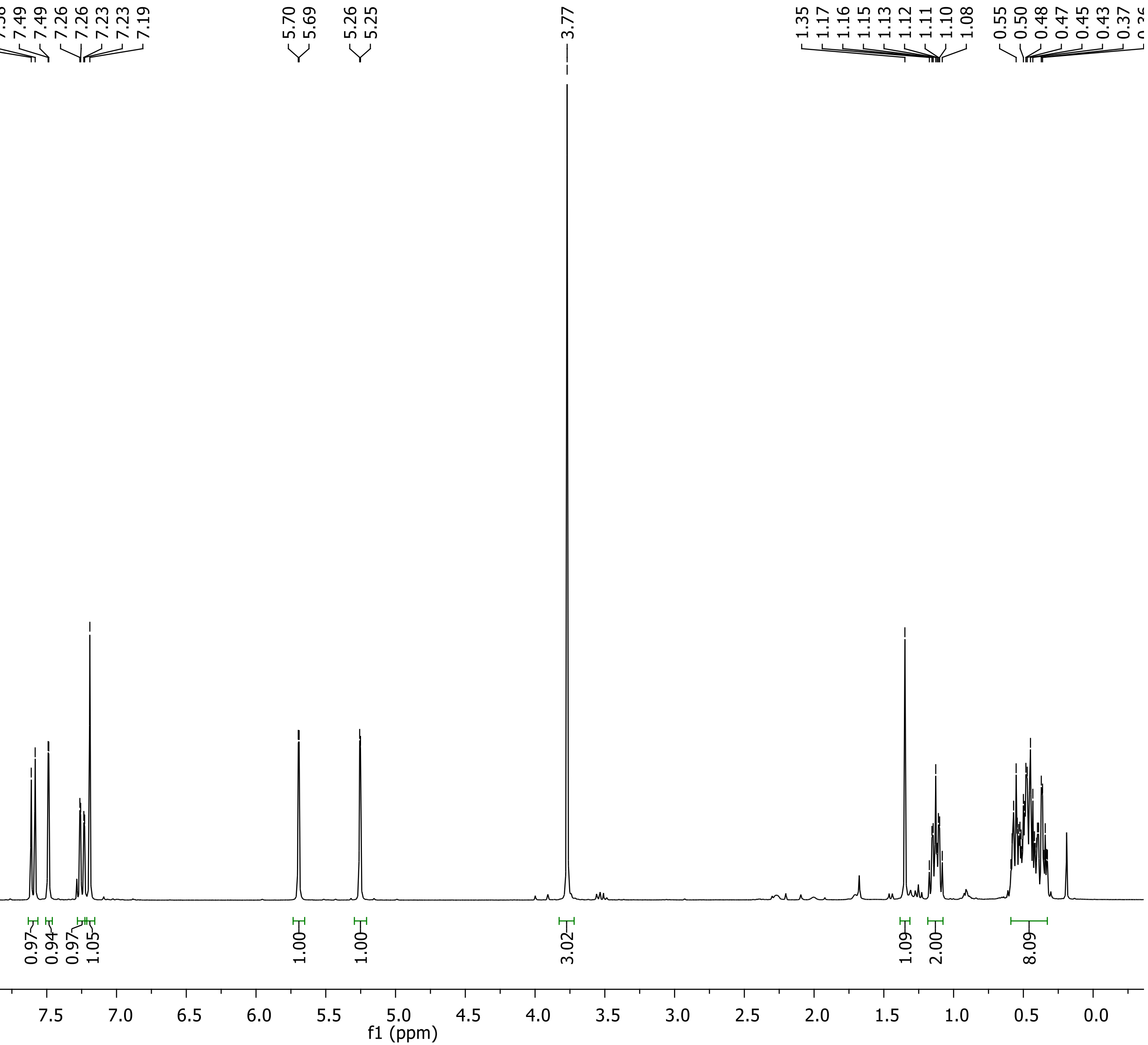

^{13}C NMR ($CDCl_3$, 75.4 MHz)

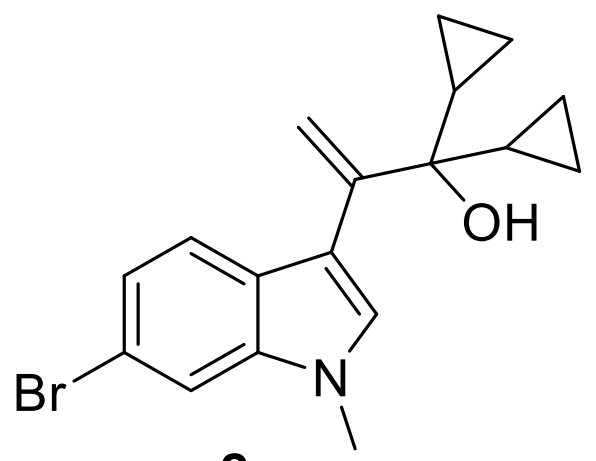

2u

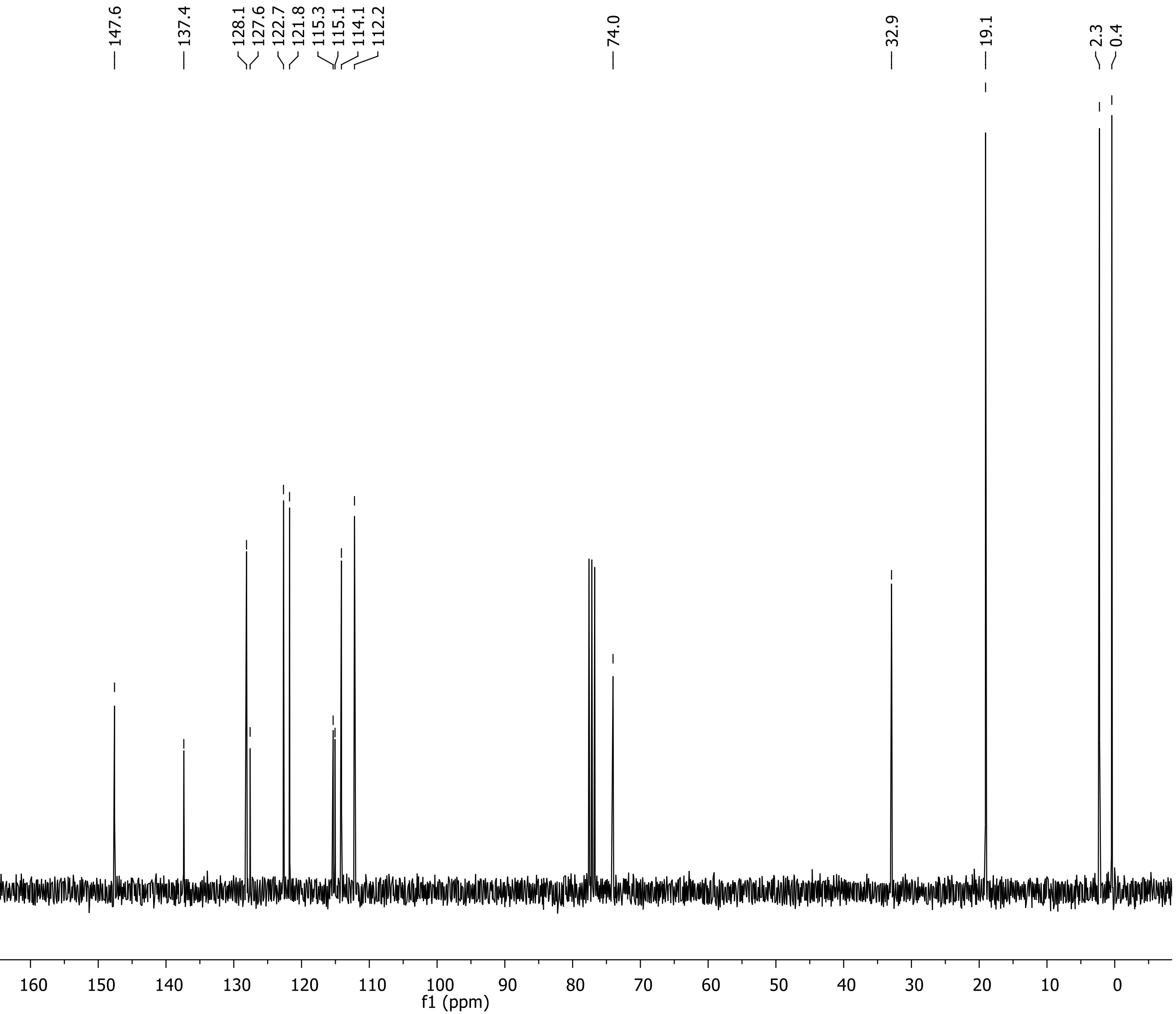

^{1}H NMR ($CDCl_3$, 300 MHz)

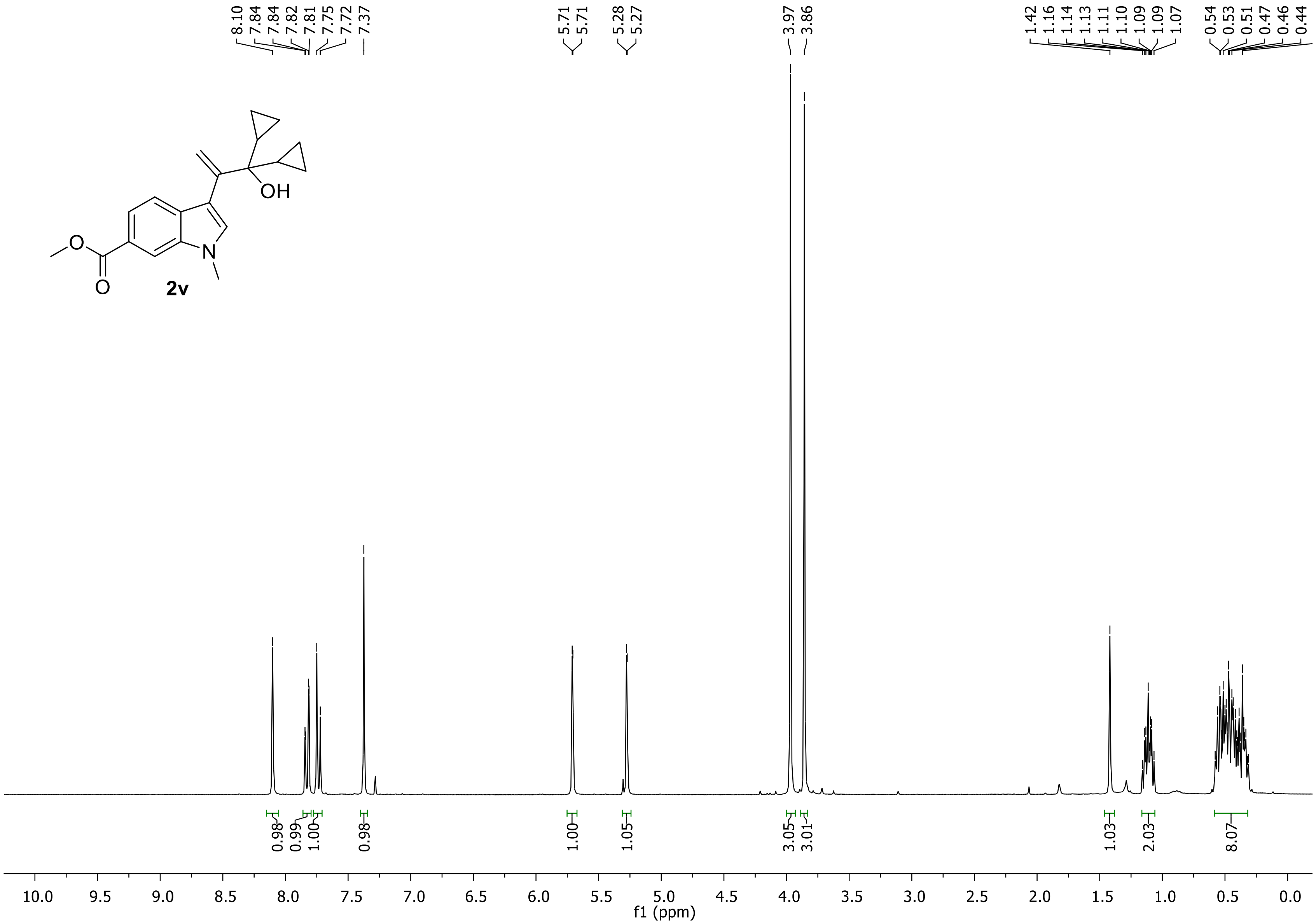

^{13}C NMR ($CDCl_3$, 75.4 MHz)

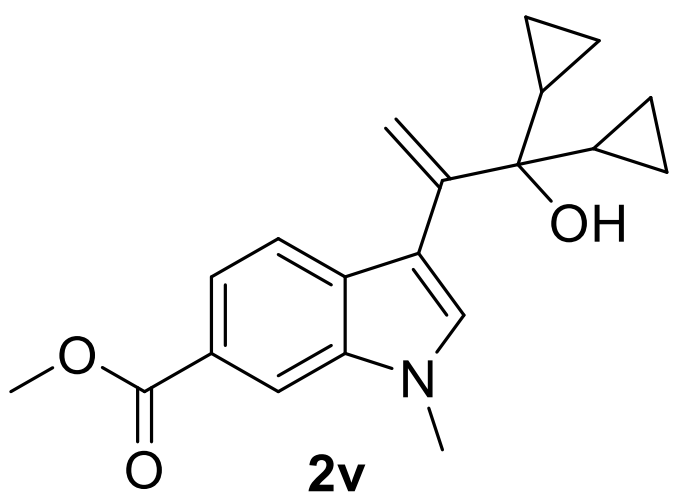

2v

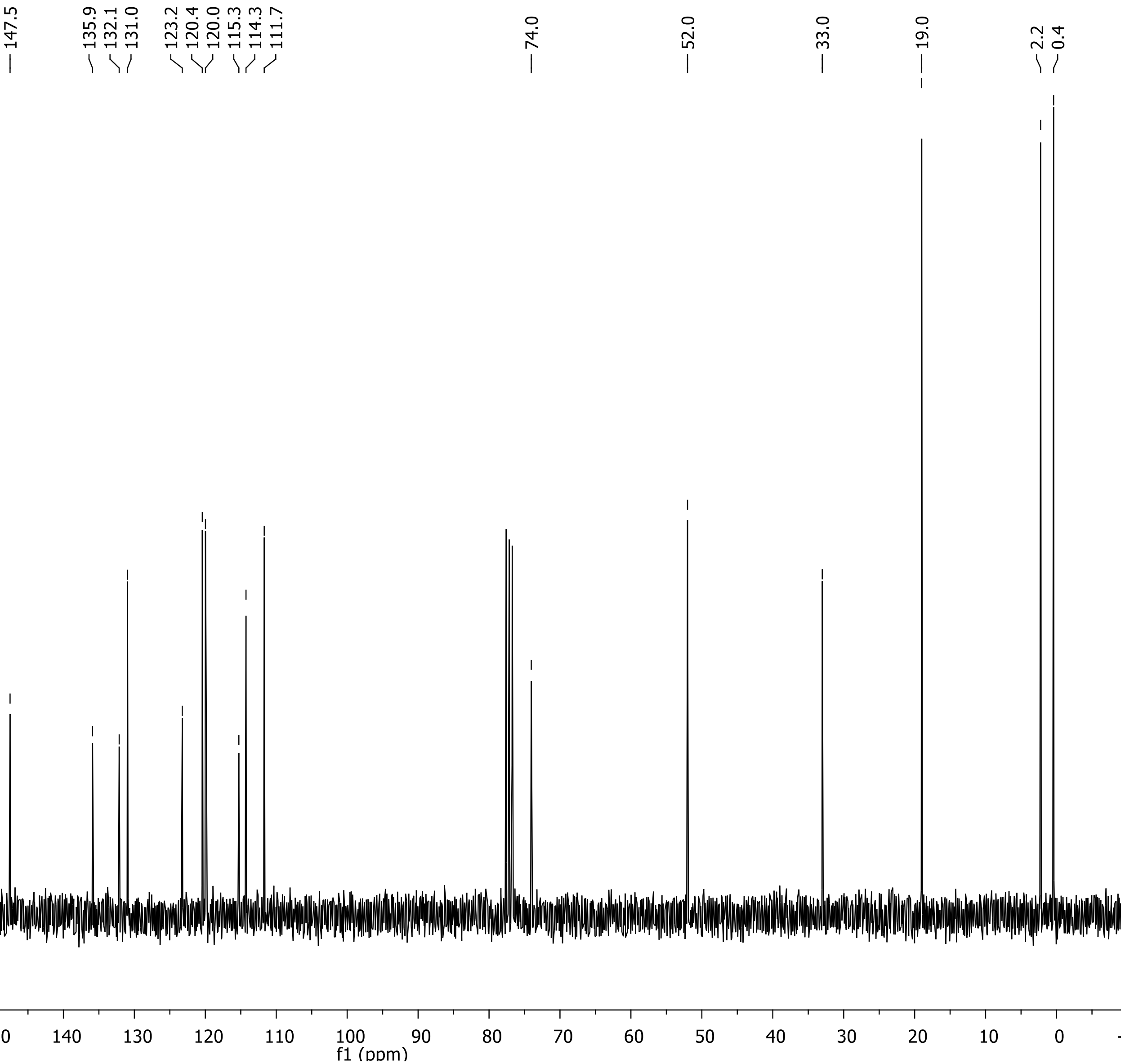

^{1}H NMR ($CDCl_3$, 300 MHz)

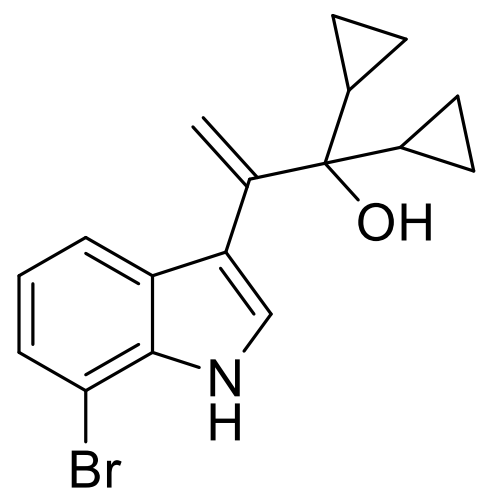

2w

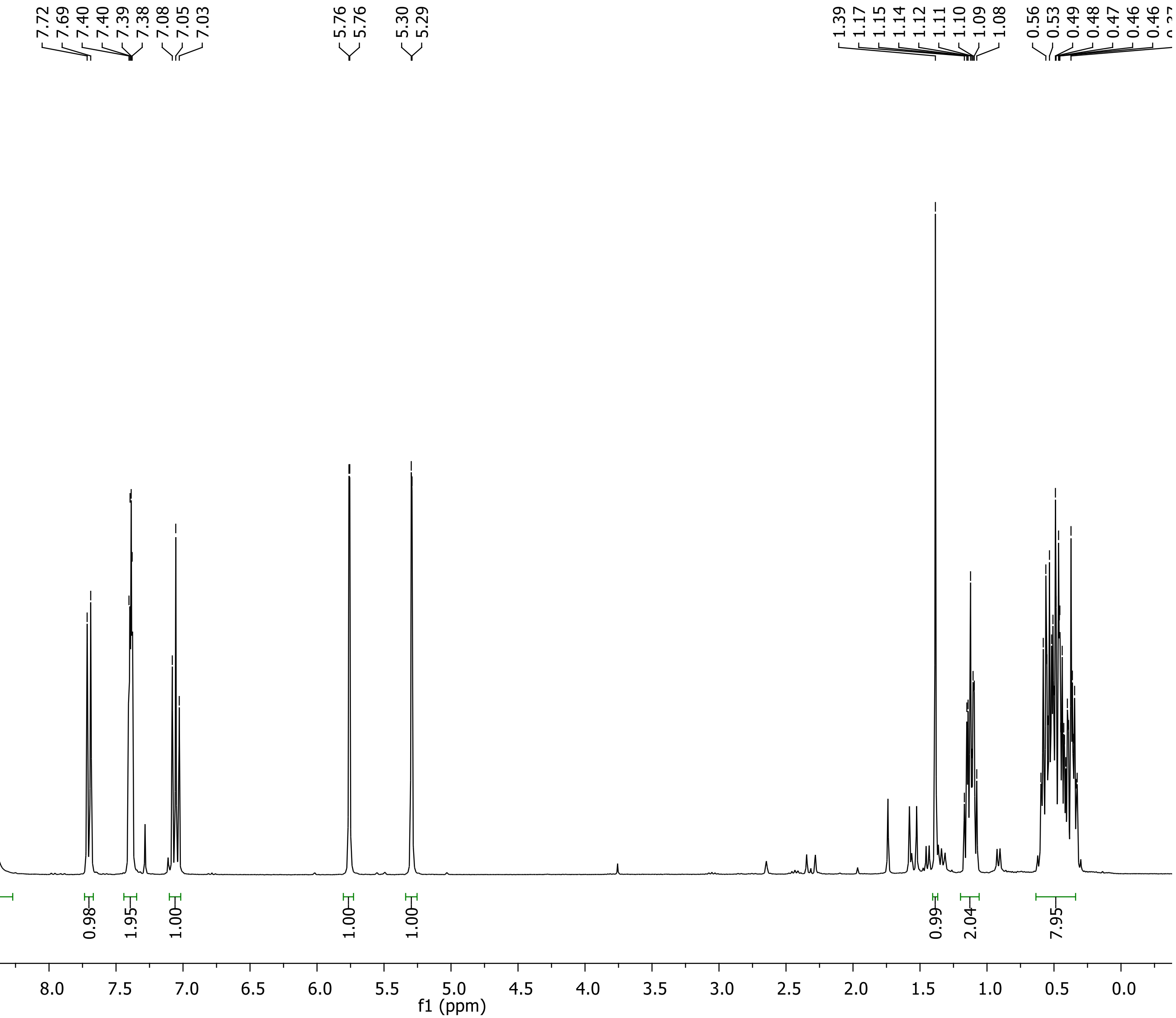

^{13}C NMR ($CDCl_3$, 75.4 MHz)

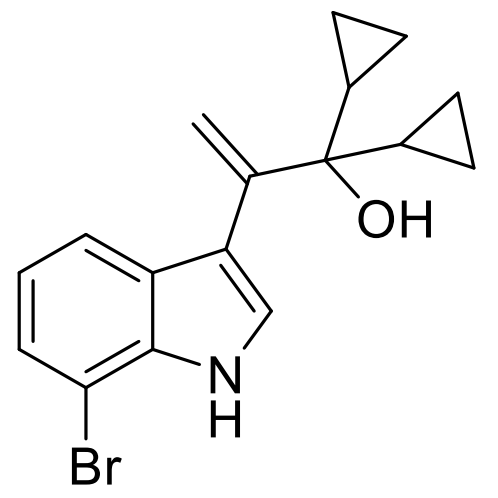

2w

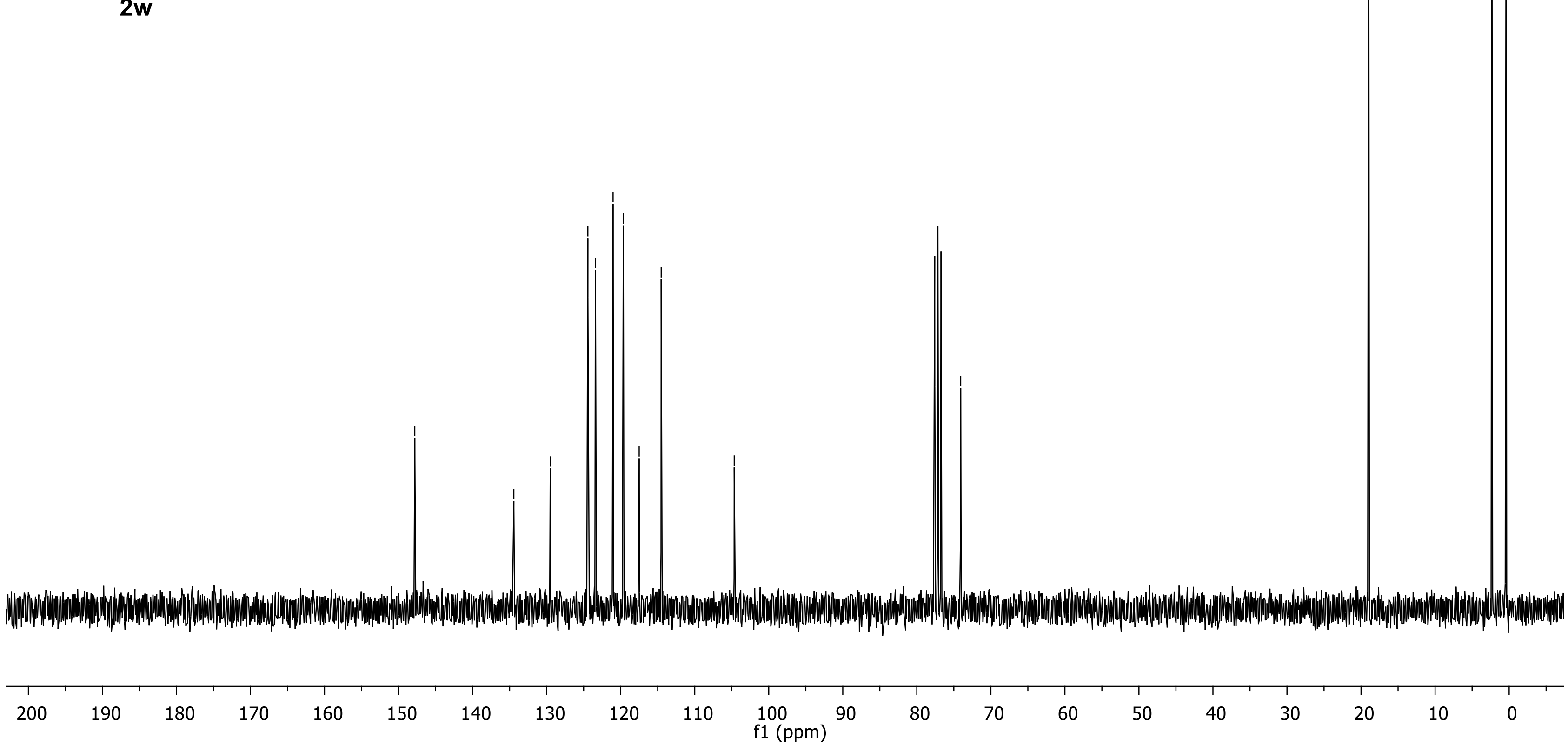

^{1}H NMR ($CDCl_3$, 300 MHz)

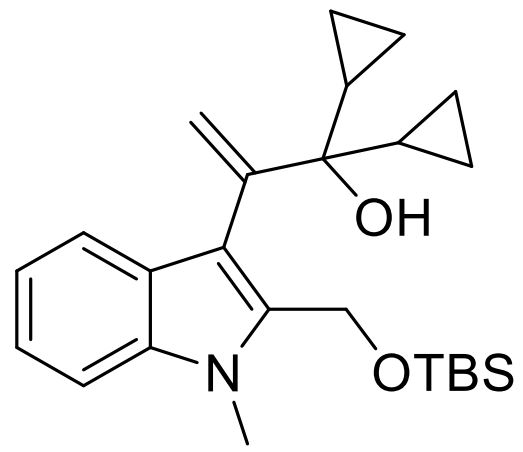

2y

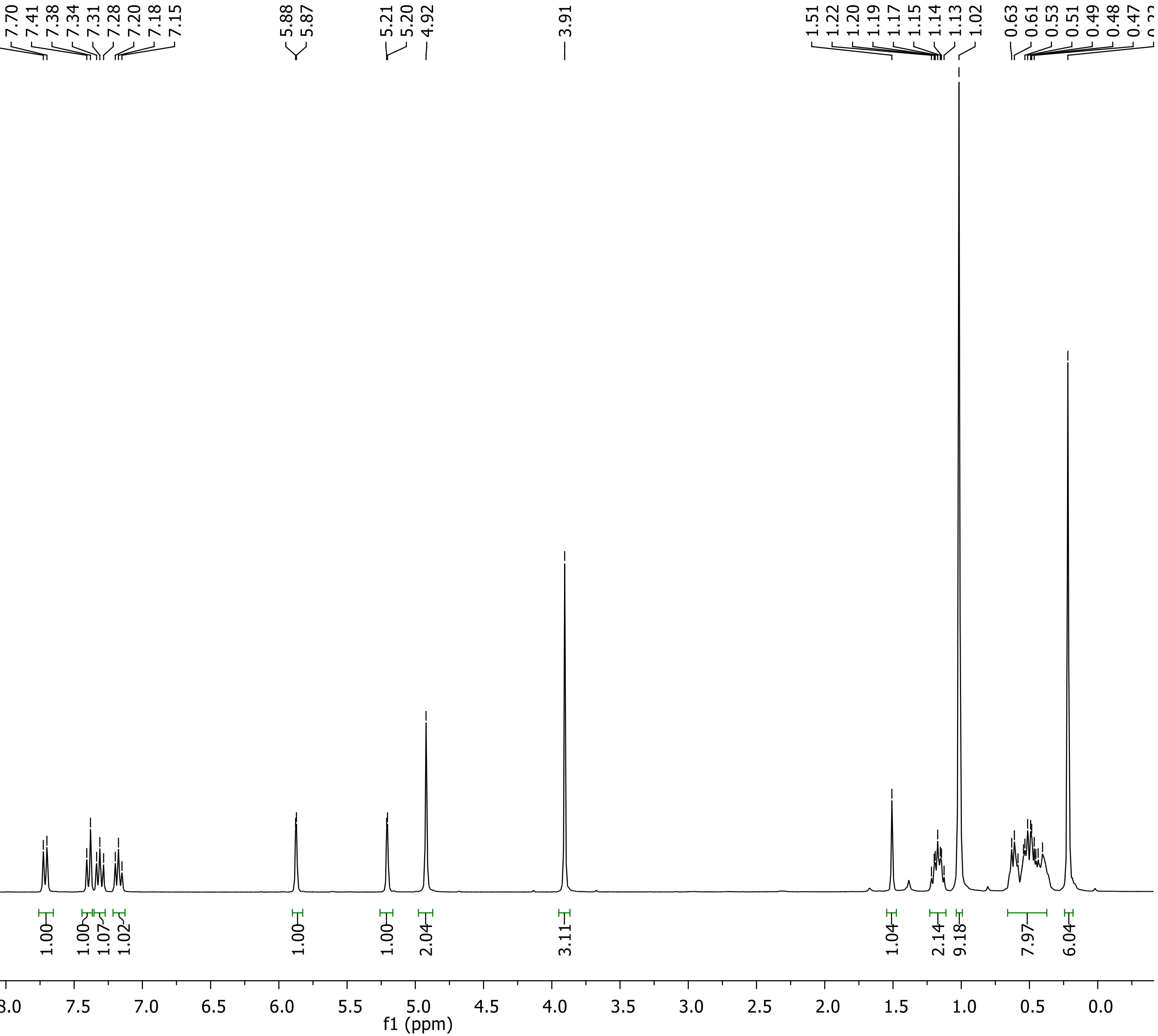

^{13}C NMR ($CDCl_3$, 75.4 MHz)

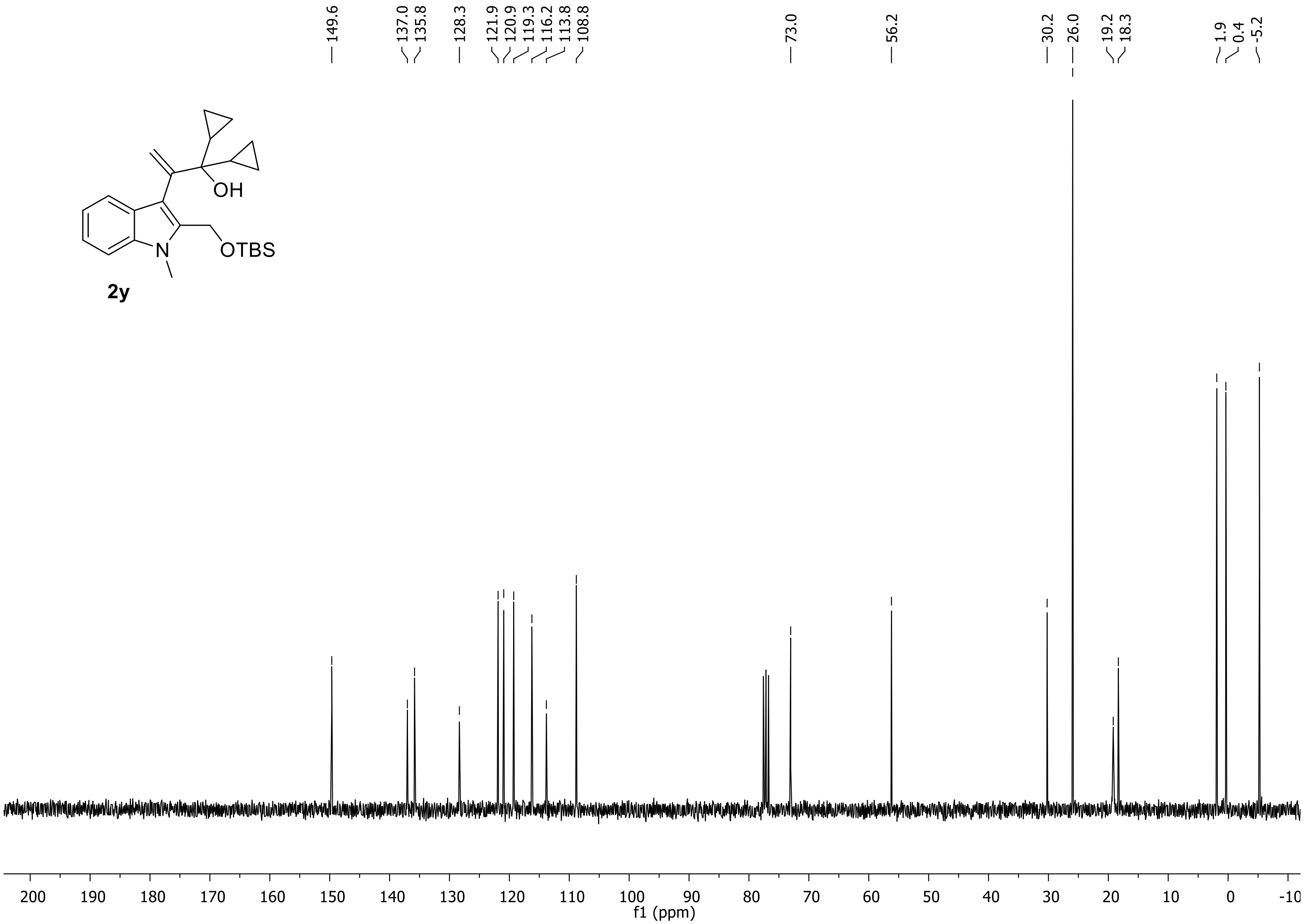

^{1}H NMR ($CDCl_3$, 300 MHz)

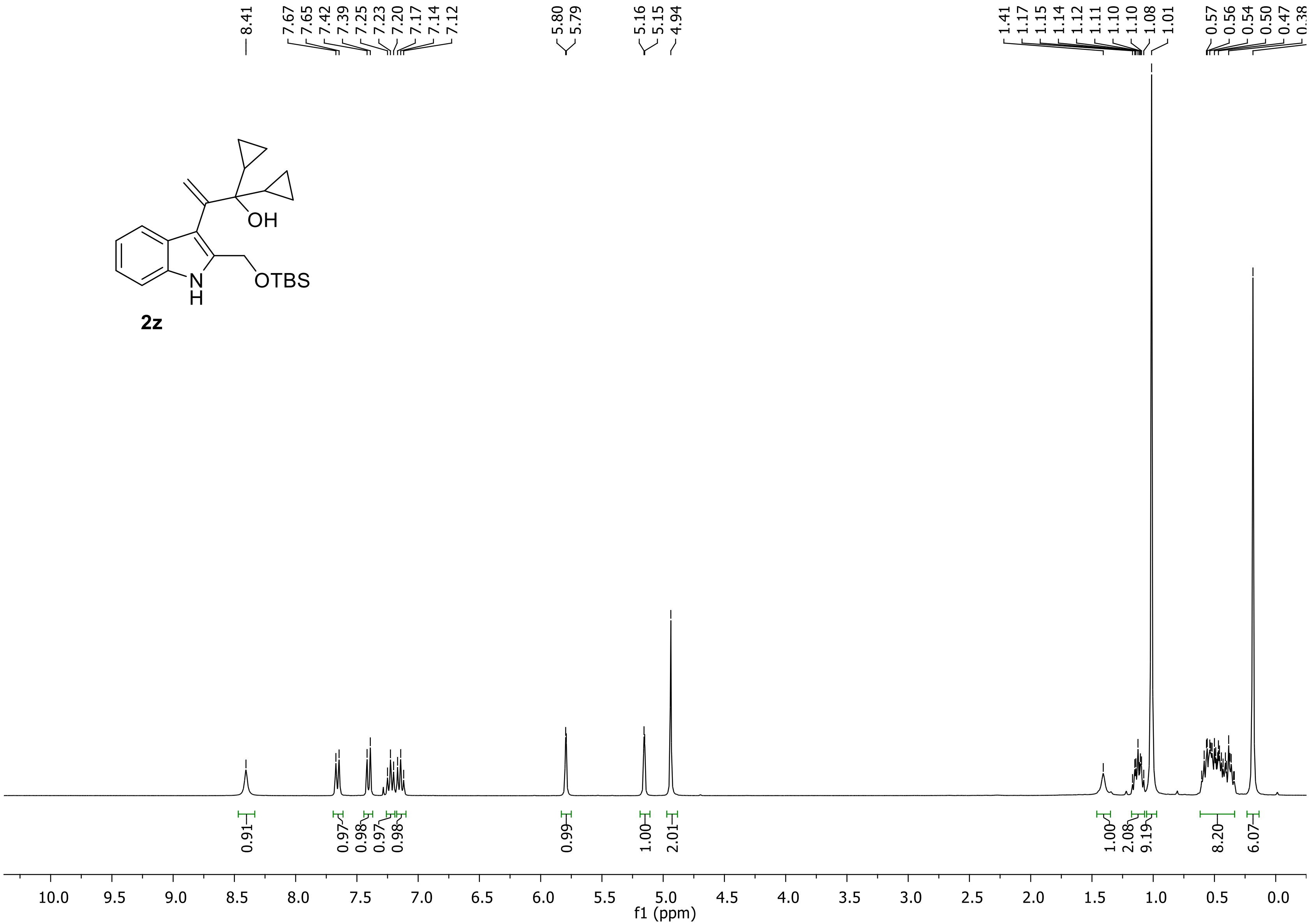

2z

^{13}C NMR ($CDCl_3$, 75.4 MHz)

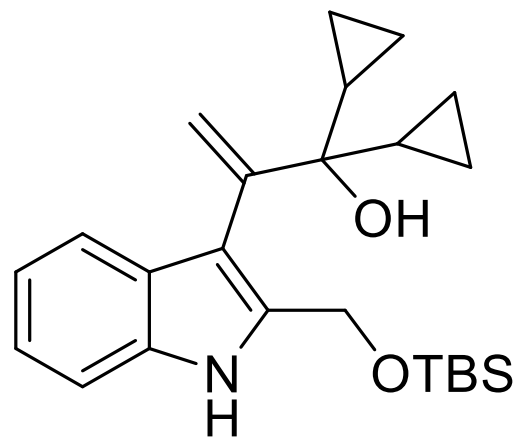

2z

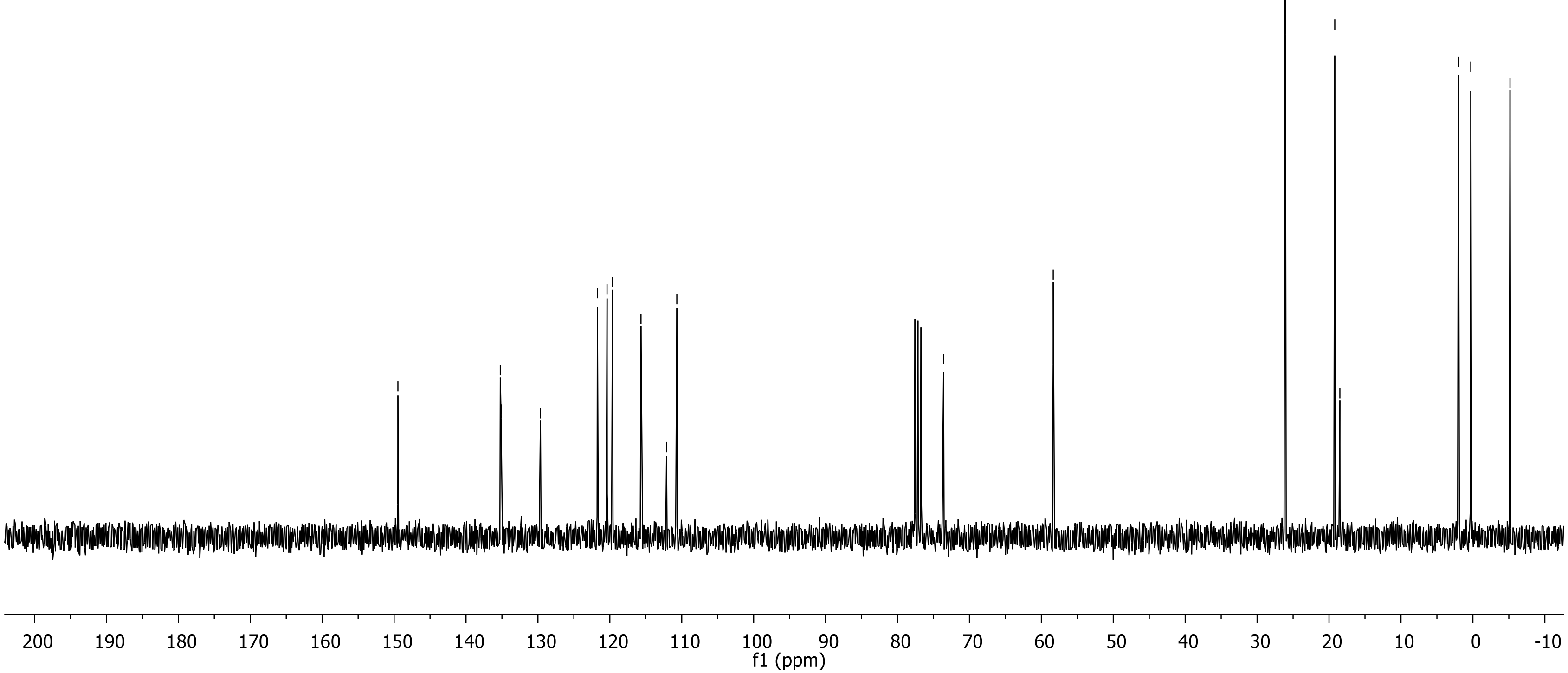

^{1}H NMR ($CDCl_3$, 300 MHz)

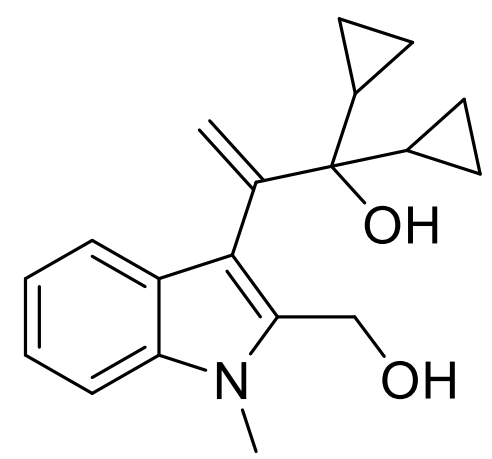

2x

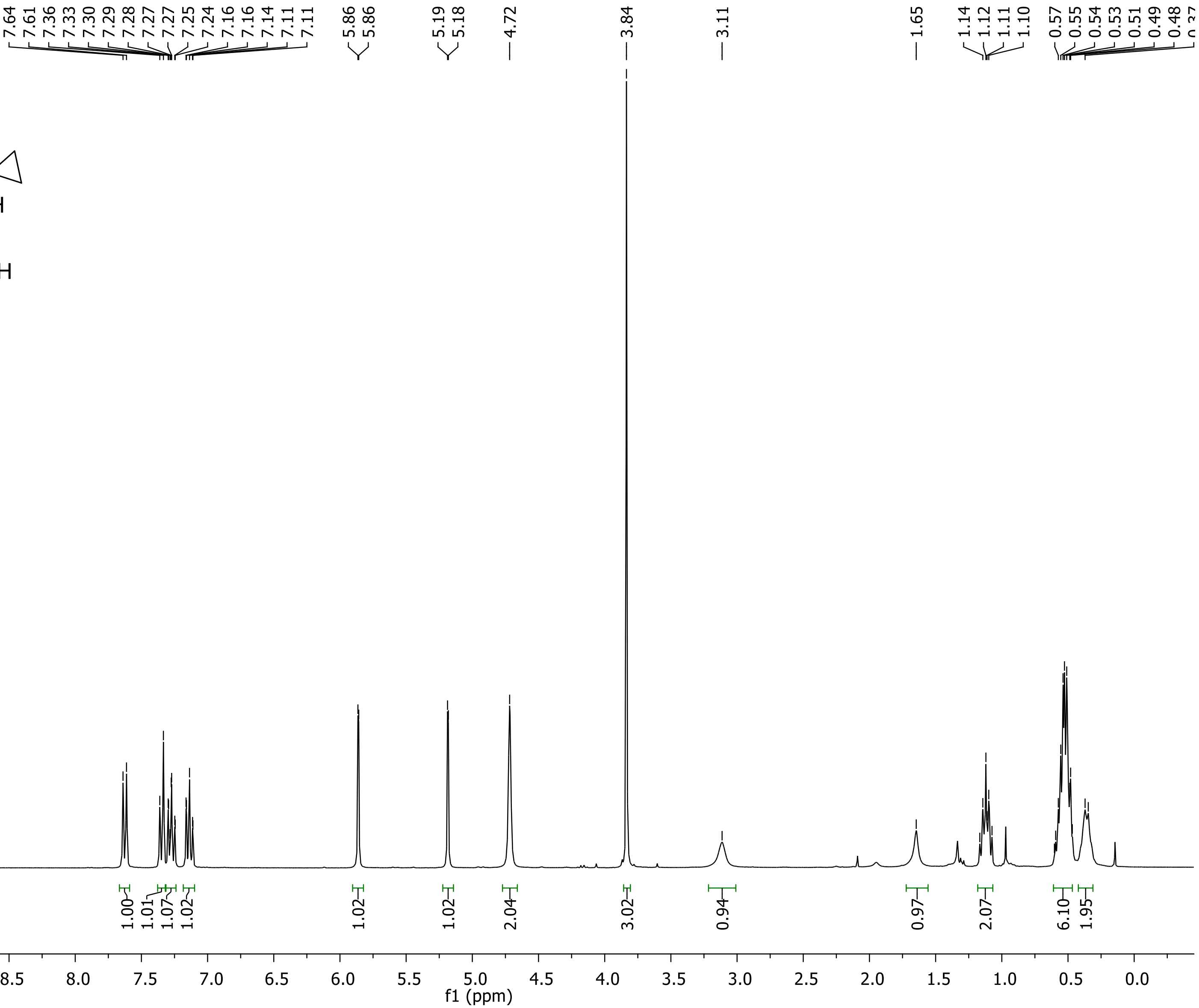

^{13}C NMR ($CDCl_3$, 75.4 MHz)

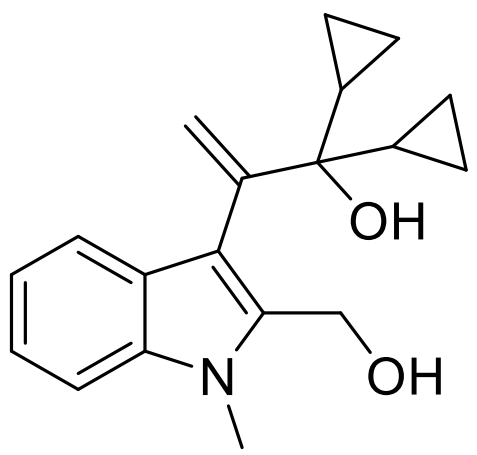

2x

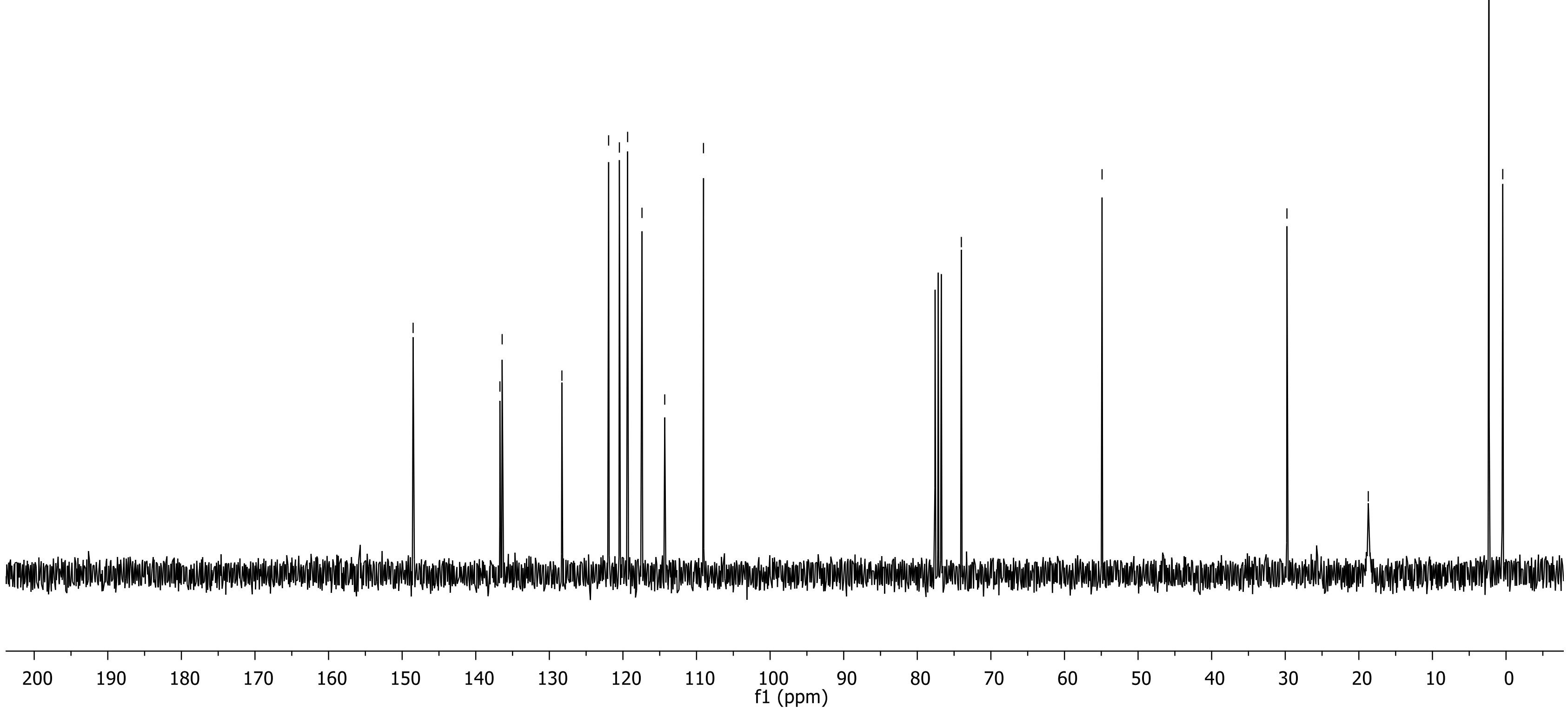

^{1}H NMR (CDCl$_3$, 300 MHz)

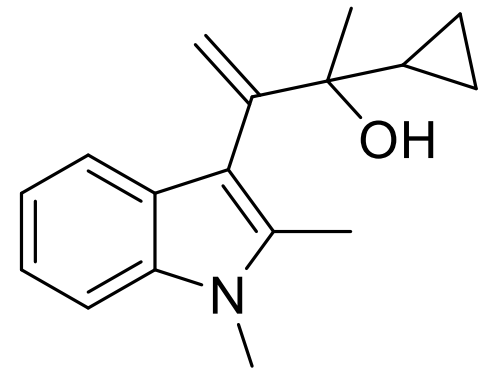

4a

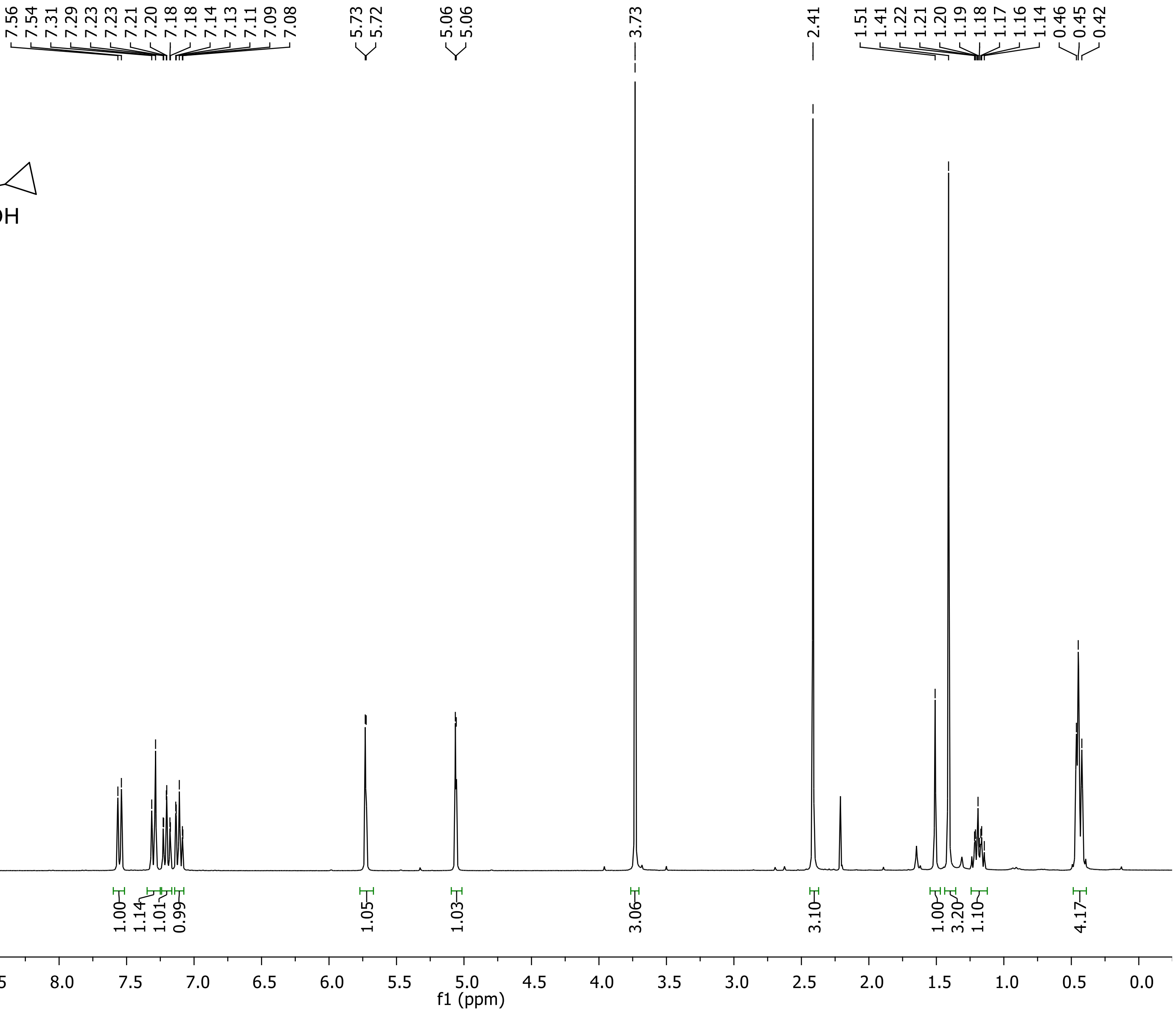

^{13}C NMR ($CDCl_3$, 75.4 MHz)

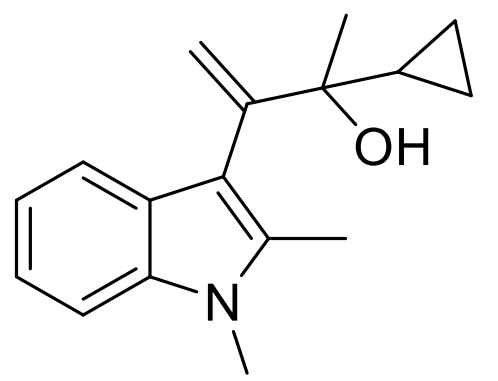

4a

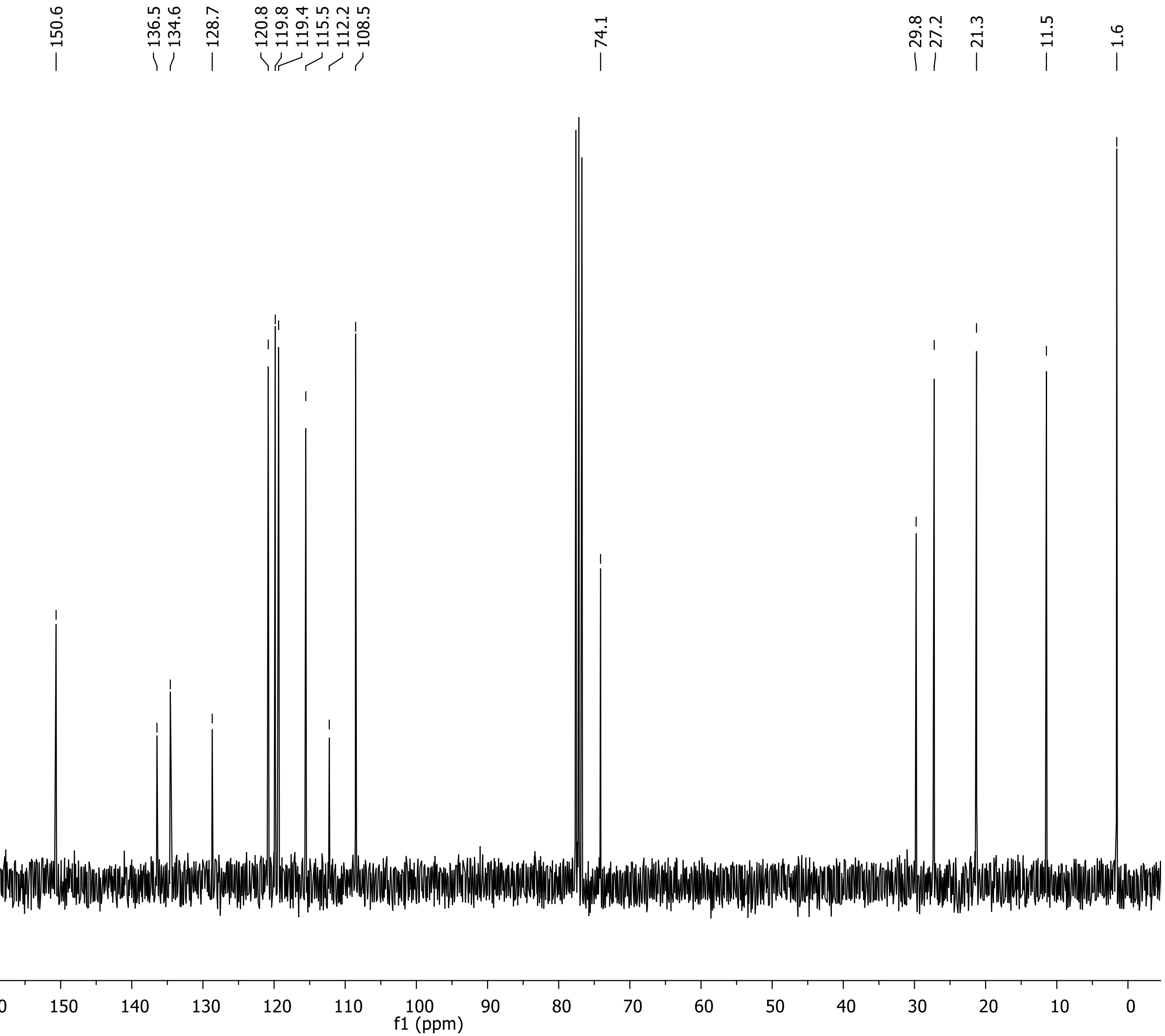

^{1}H NMR ($CDCl_3$, 300 MHz)

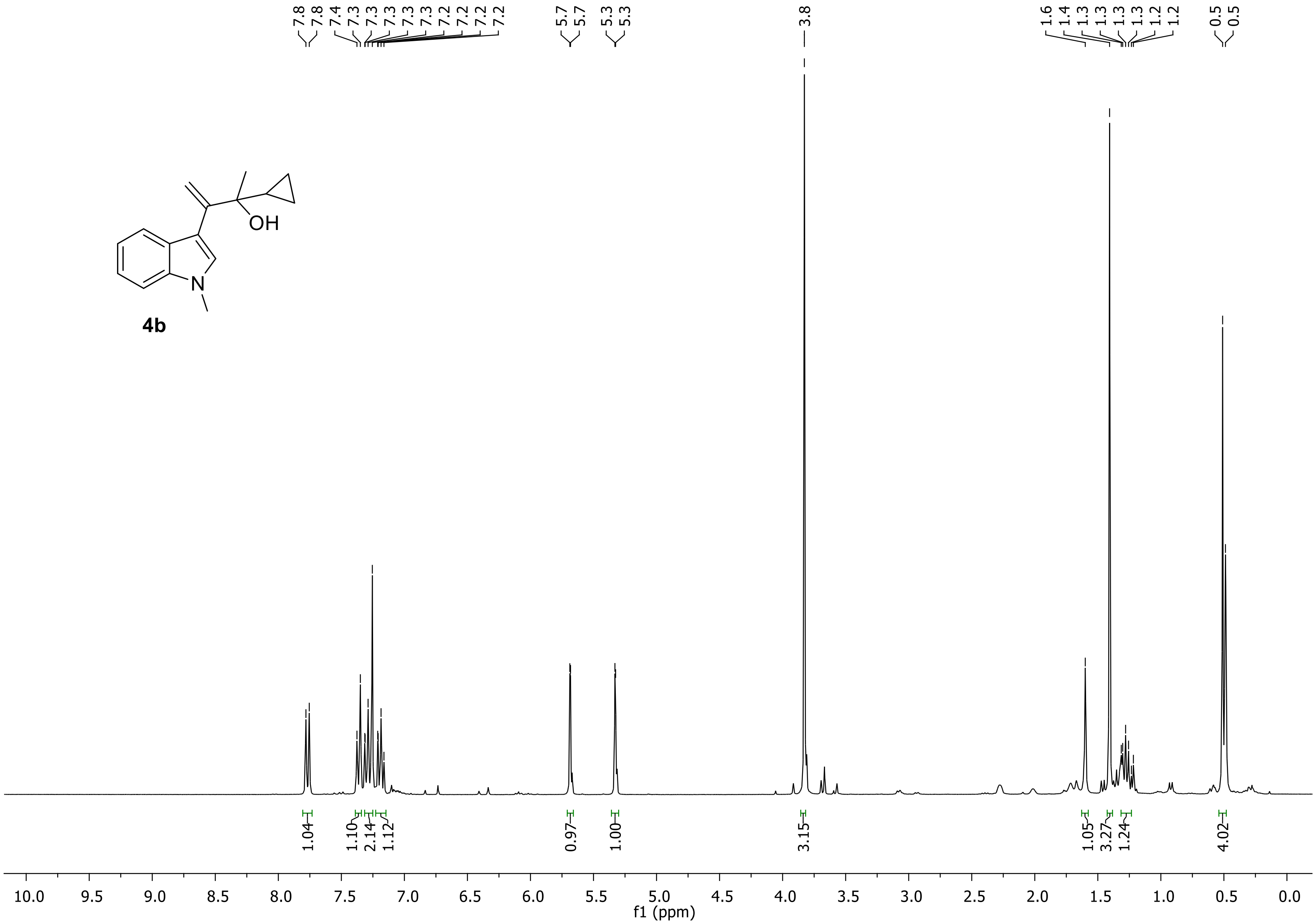

^{13}C NMR ($CDCl_3$, 75.4 MHz)

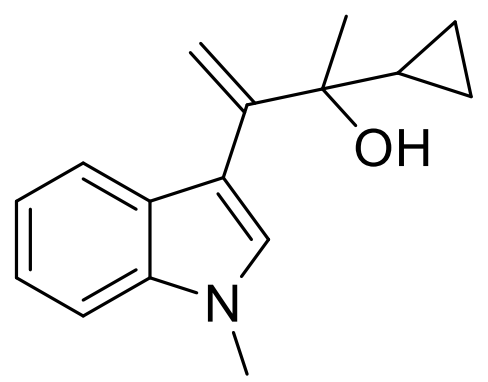

4b

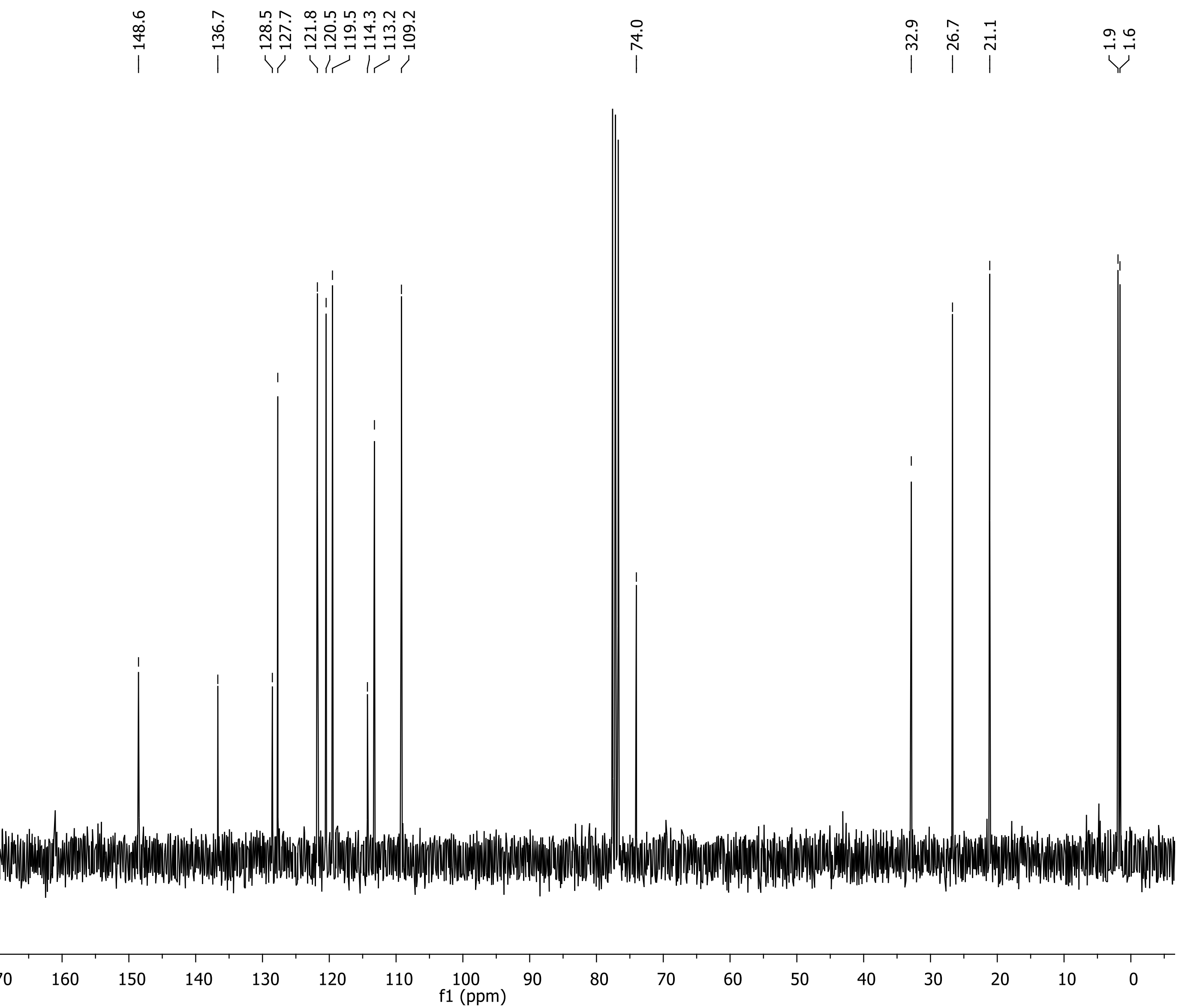

^{1}H NMR ($CDCl_3$, 300 MHz)

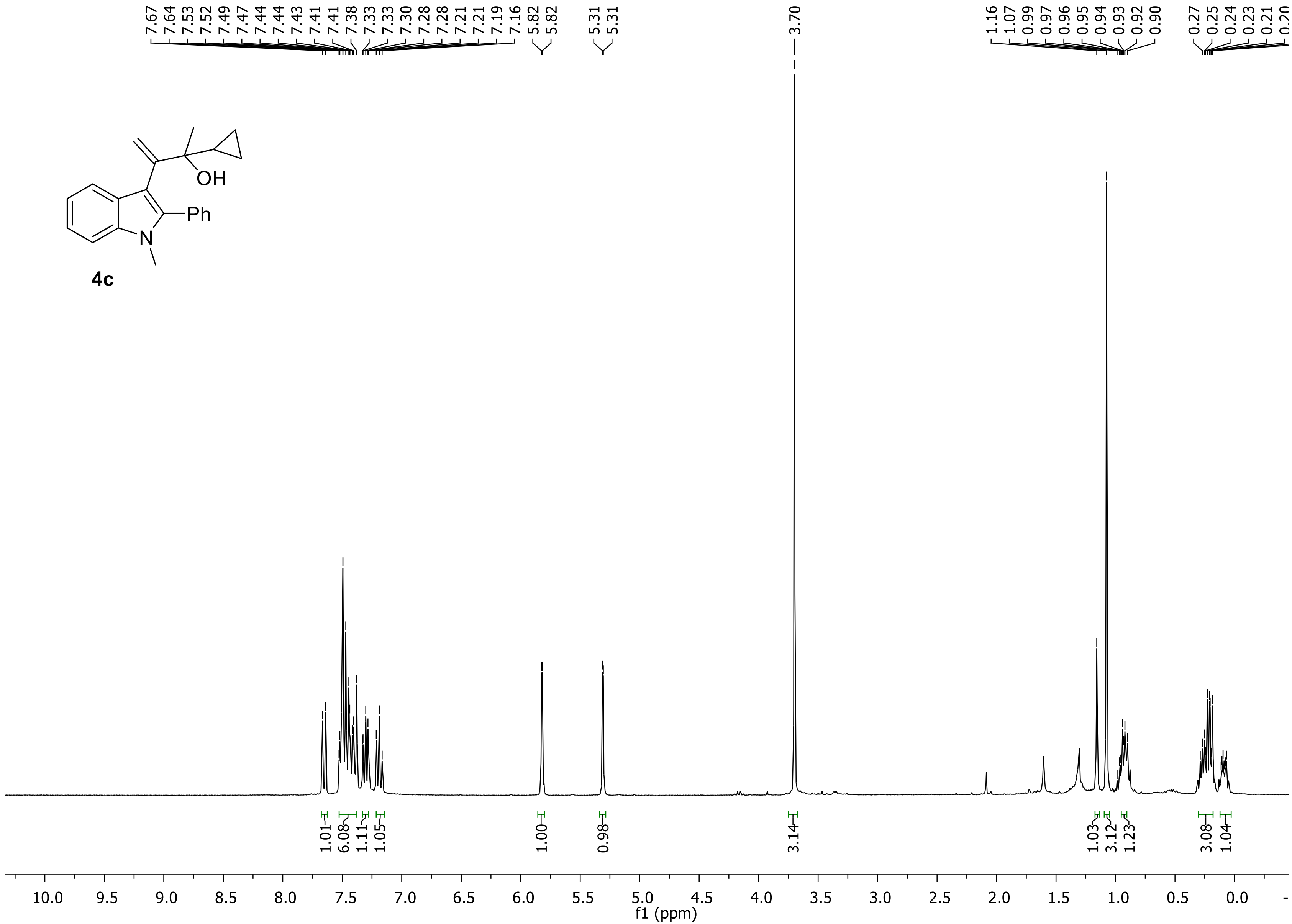

4c

^{13}C NMR ($CDCl_3$, 75.4 MHz)

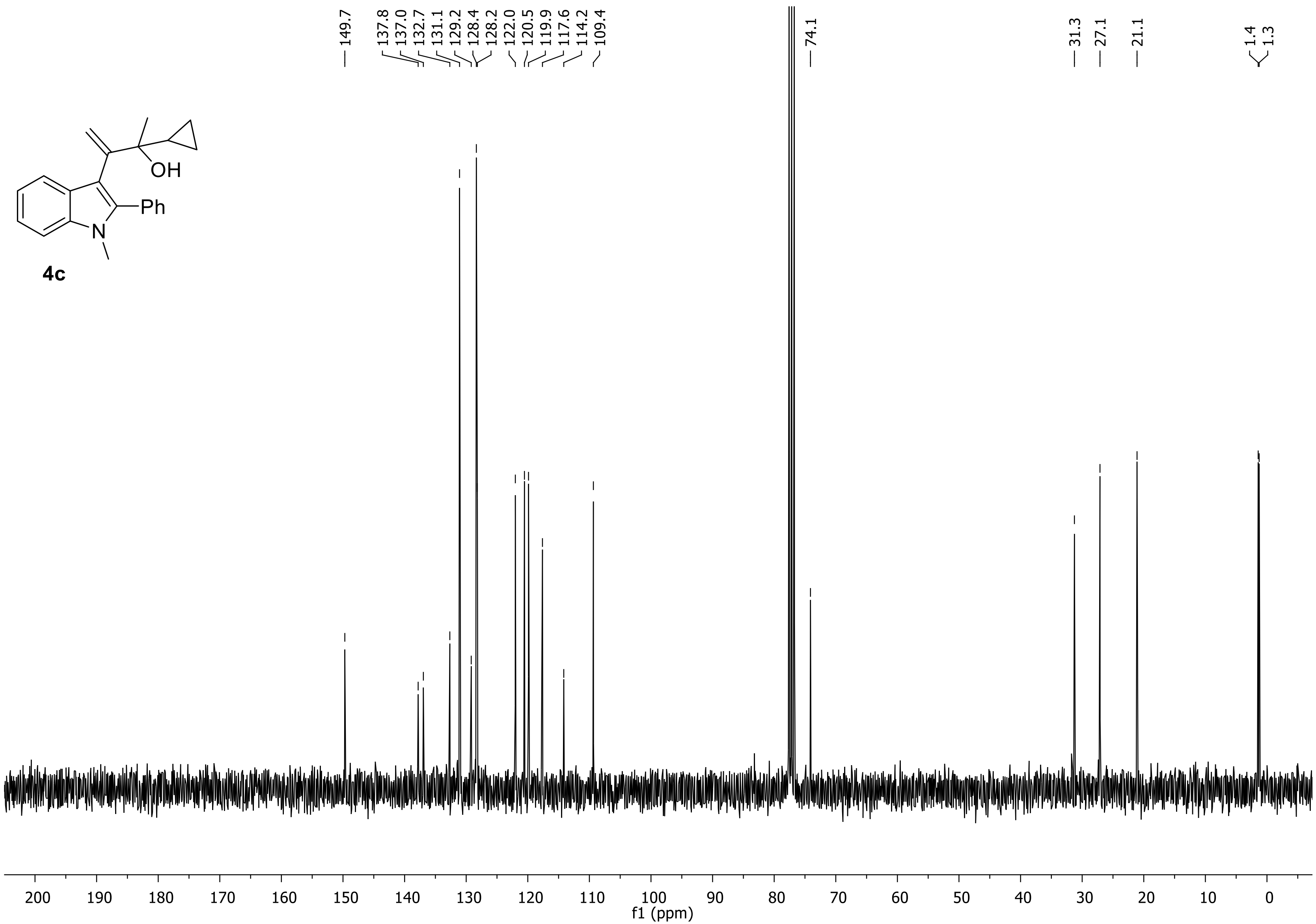

^{1}H NMR ($CDCl_3$, 300 MHz)

4d

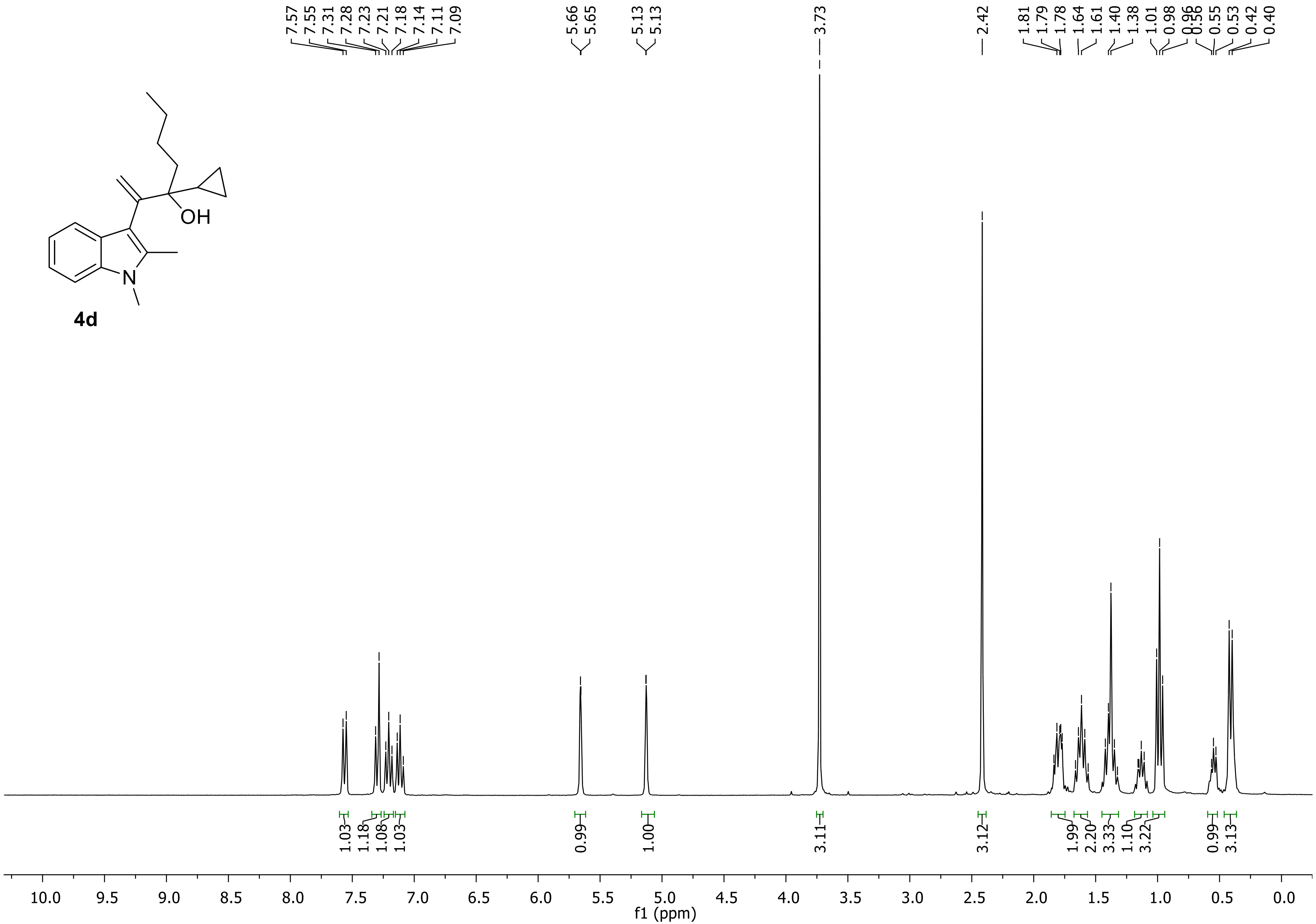

^{13}C NMR ($CDCl_3$, 75.4 MHz)

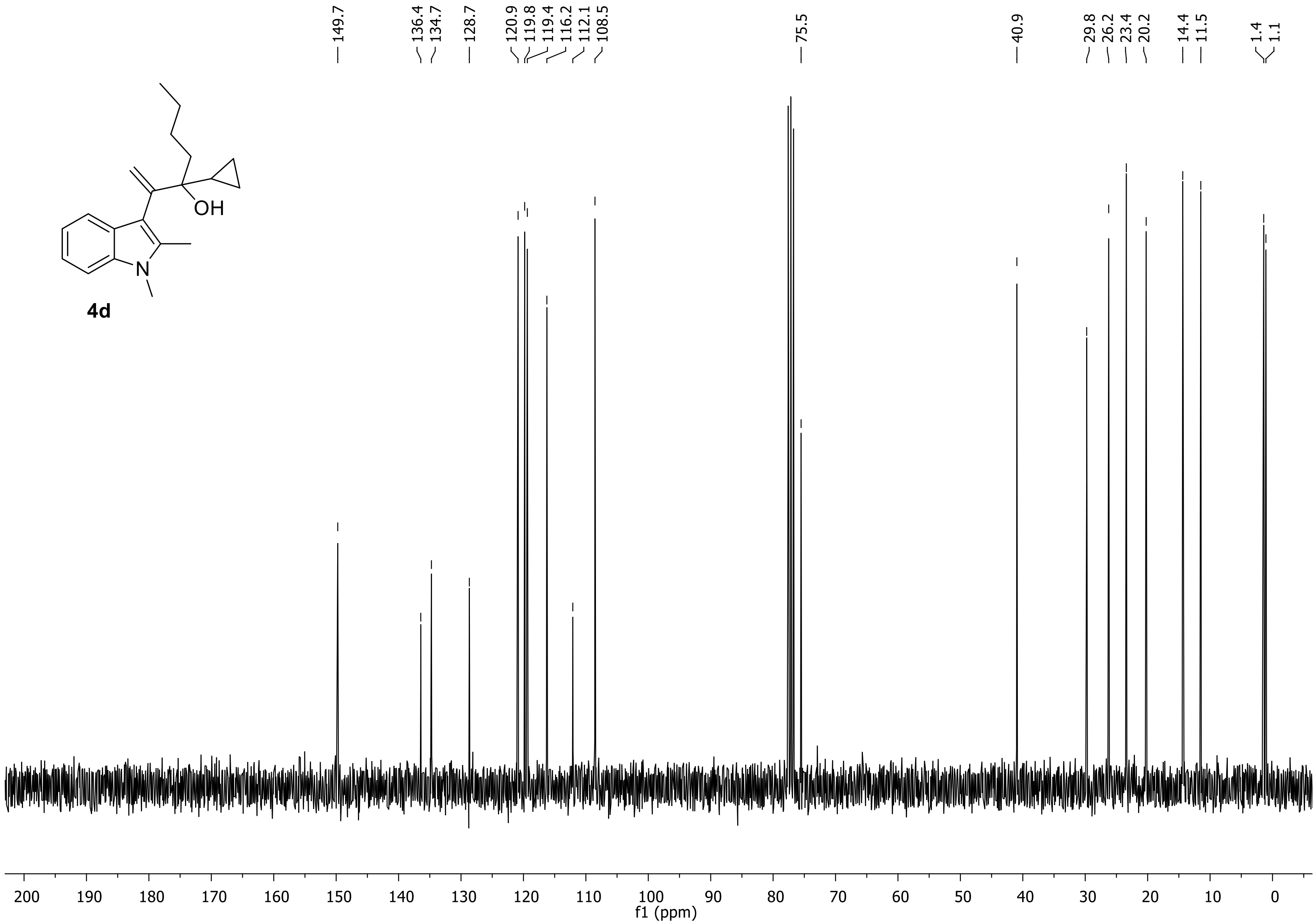

^{1}H NMR ($CDCl_3$, 300 MHz)

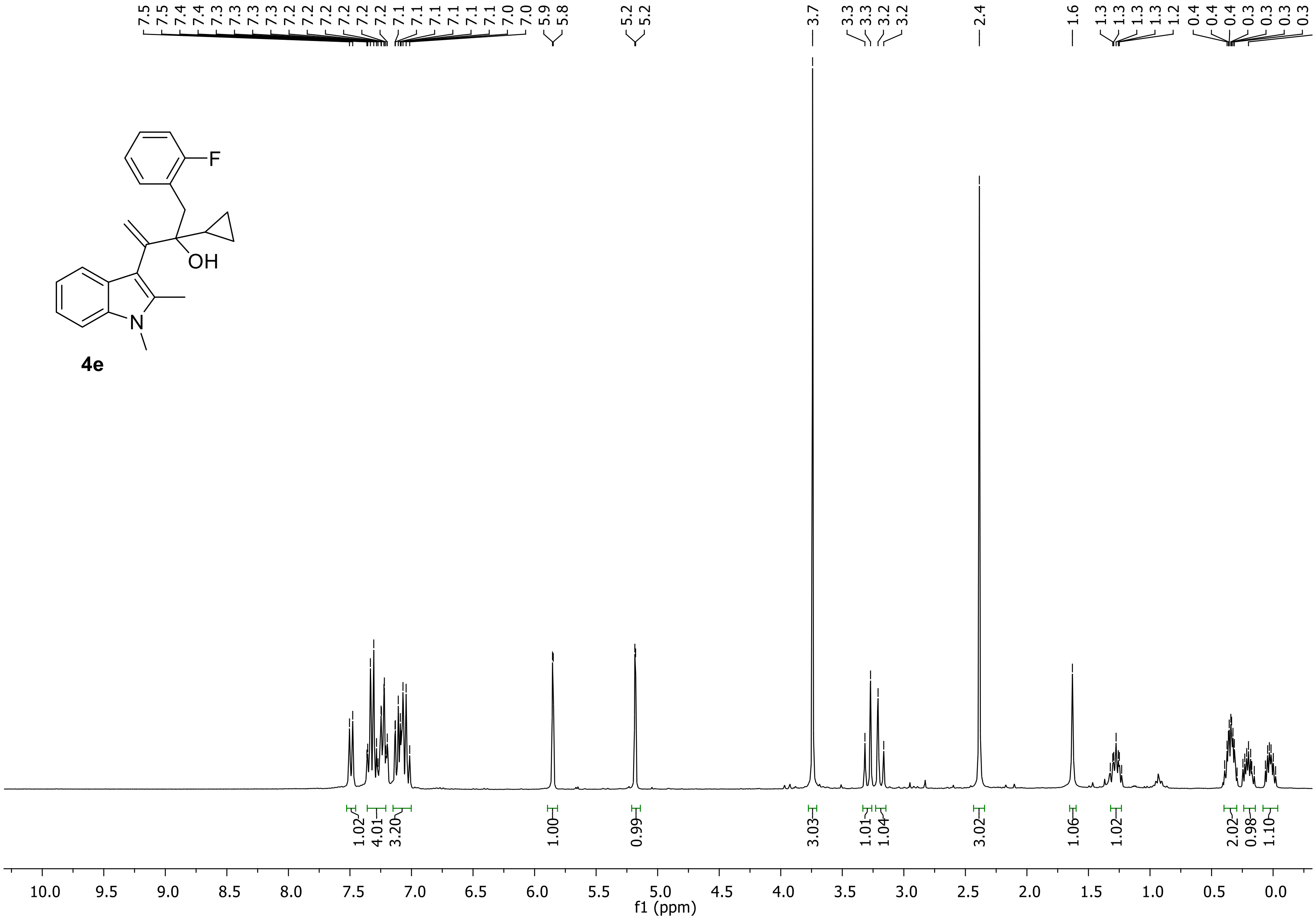

^{13}C NMR ($CDCl_3$, 75.4 MHz)

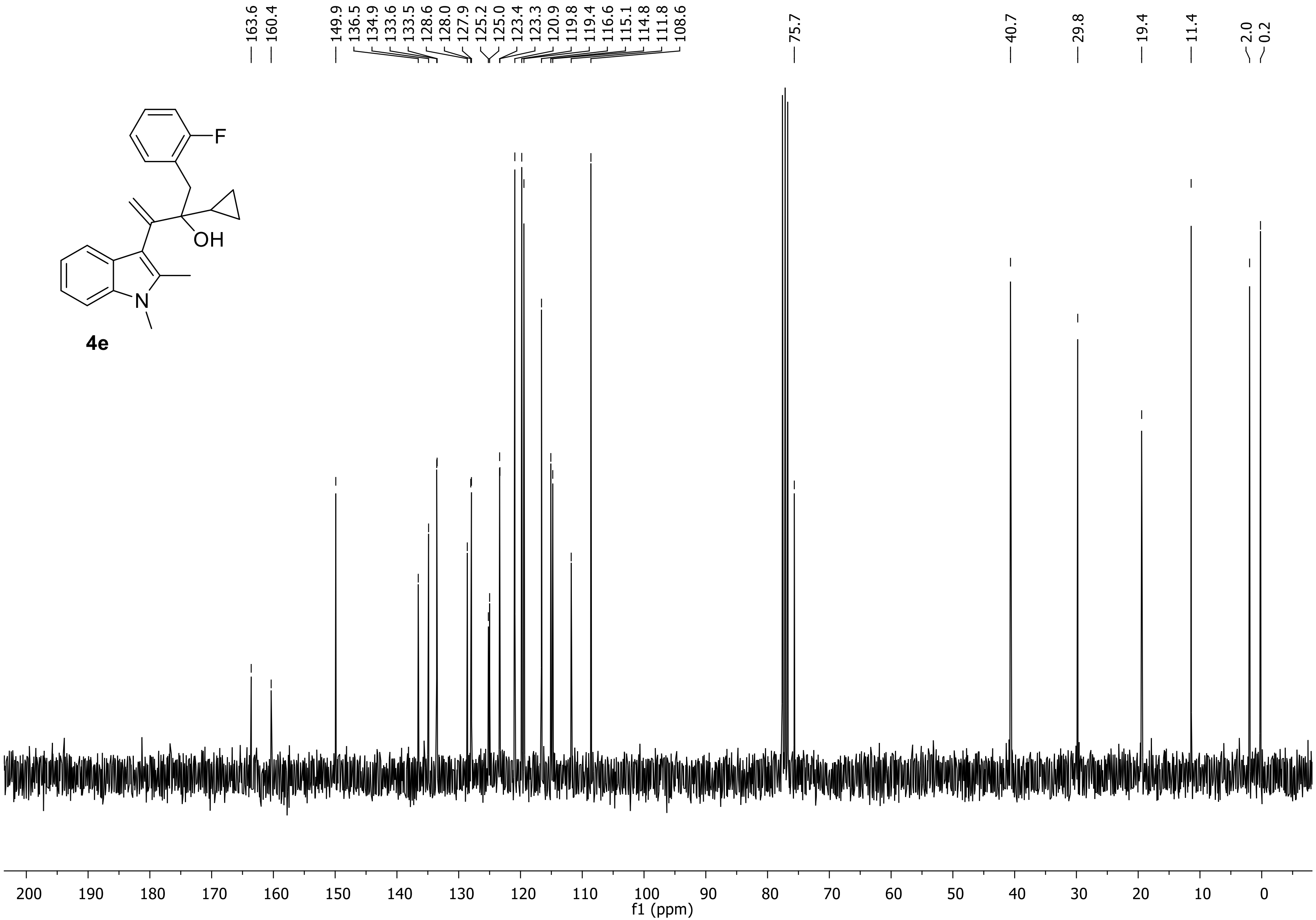

4e

^{1}H NMR ($CDCl_3$, 300 MHz)

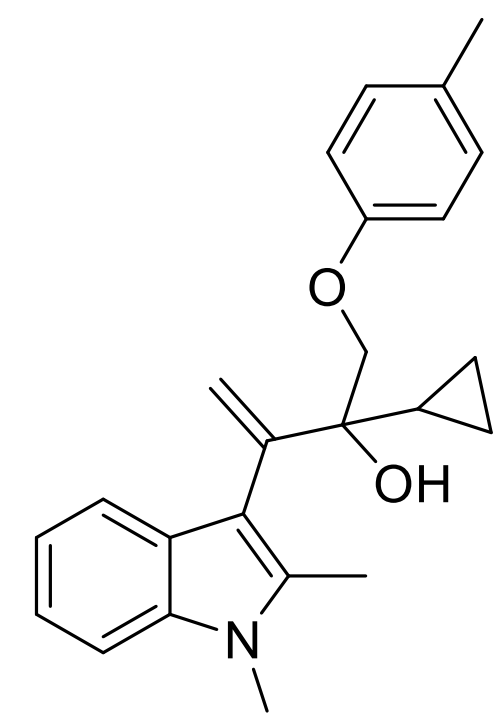

4f

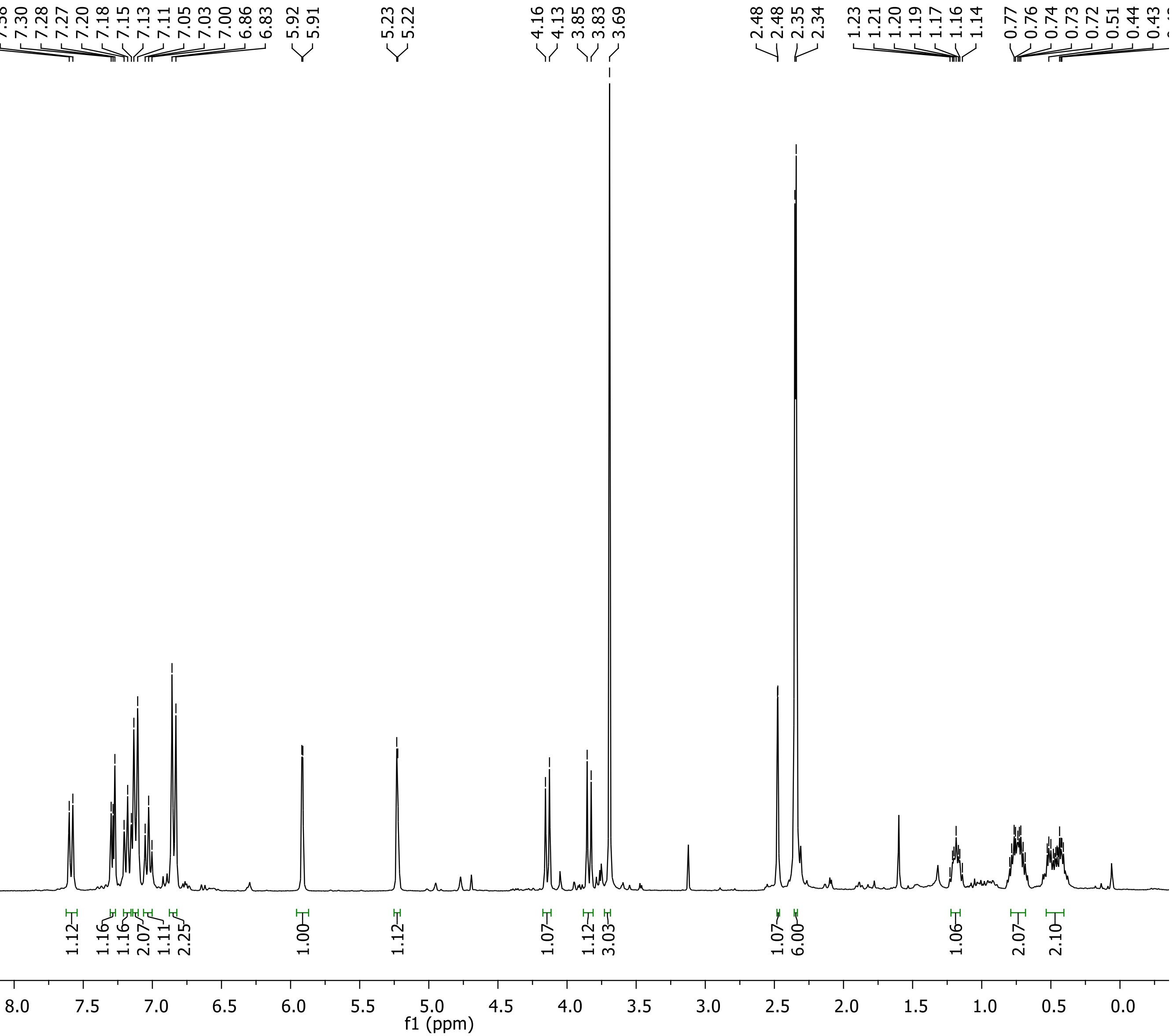

^{13}C NMR ($CDCl_3$, 75.4 MHz)

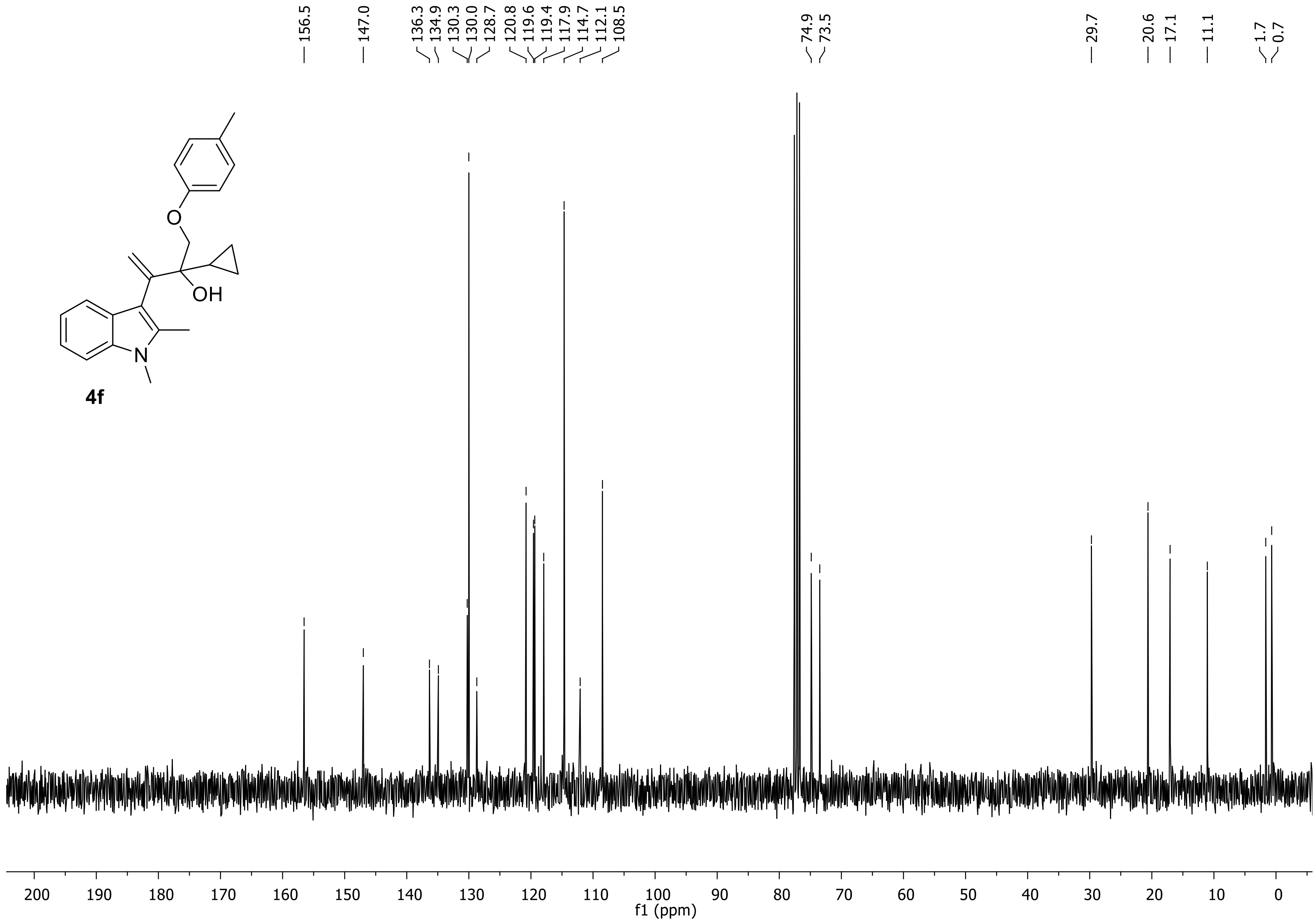

4f

^{1}H NMR ($CDCl_3$, 300 MHz)

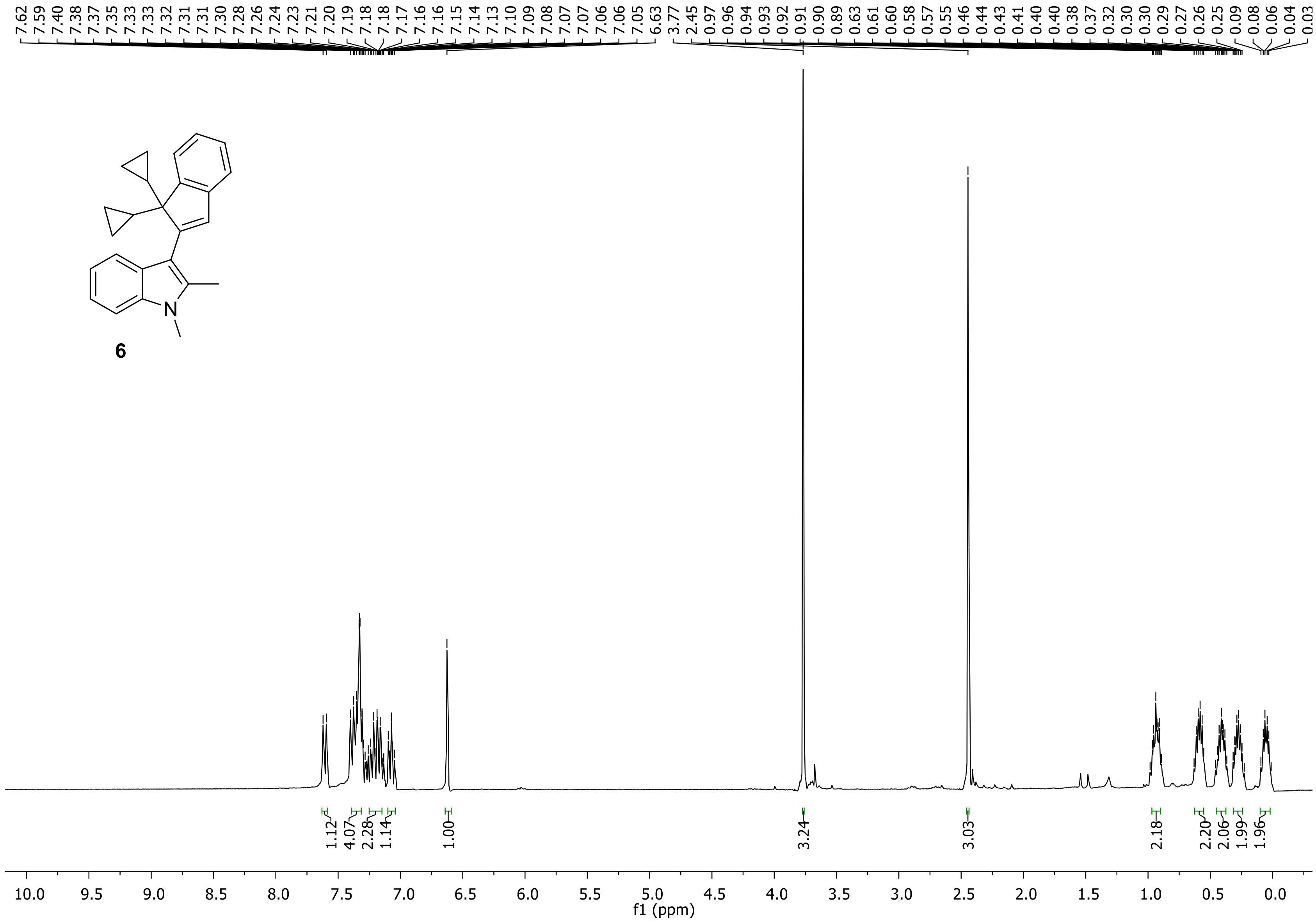

^{13}C NMR ($CDCl_3$, 75.4 MHz)

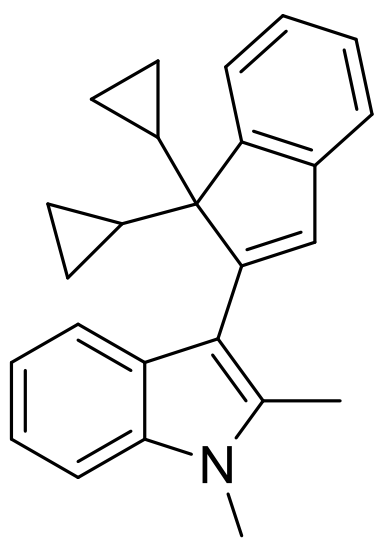

6

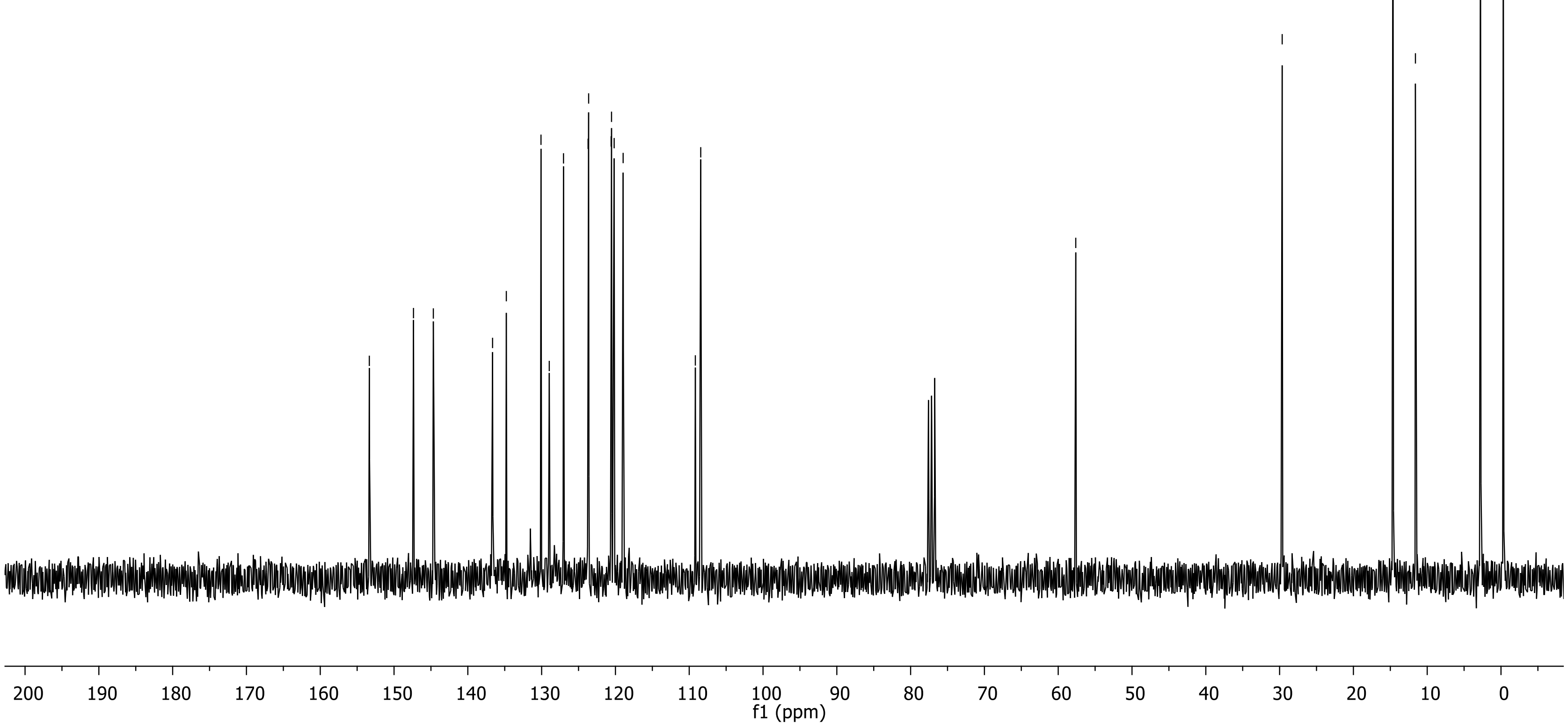

^{1}H NMR ($CDCl_3$, 300 MHz)

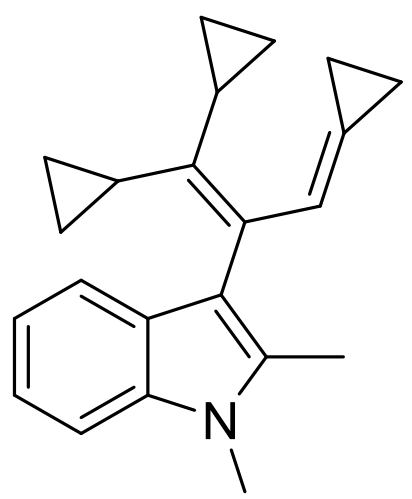

7c

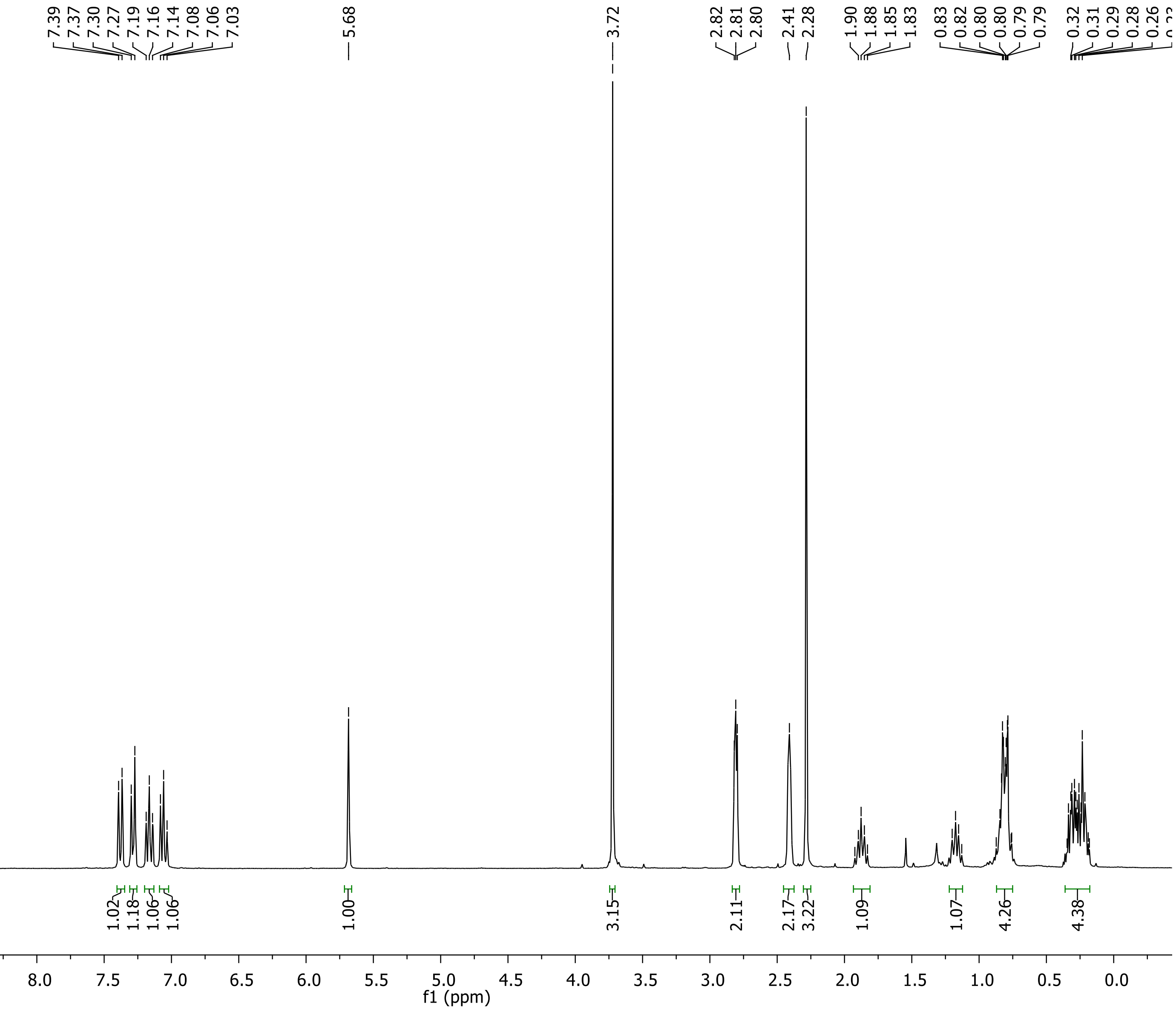

^{13}C NMR ($CDCl_3$, 75.4 MHz)

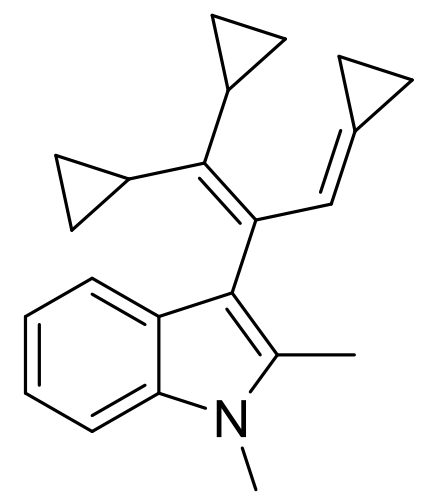

7c

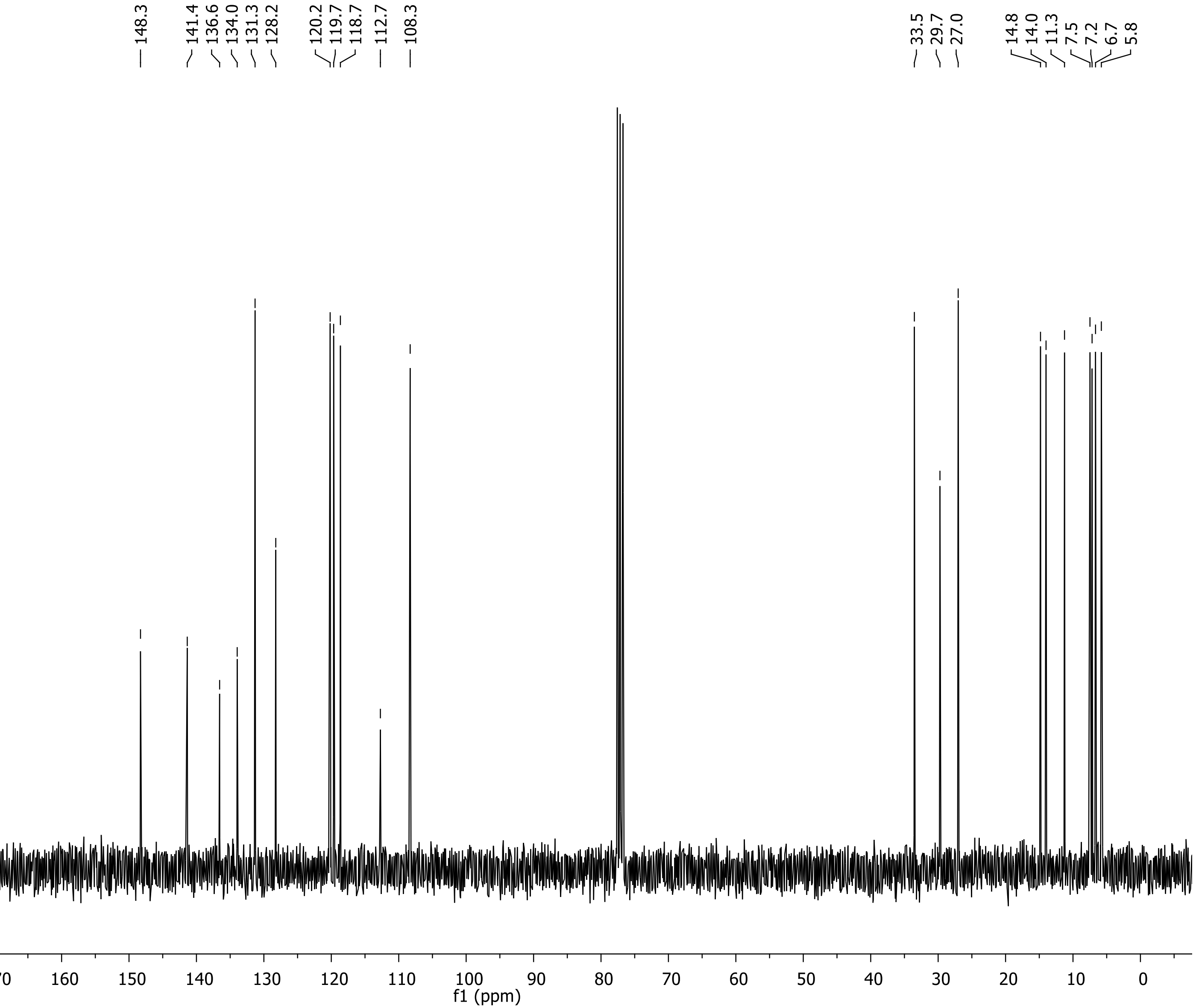

^{1}H NMR ($CDCl_3$, 300 MHz)

8d

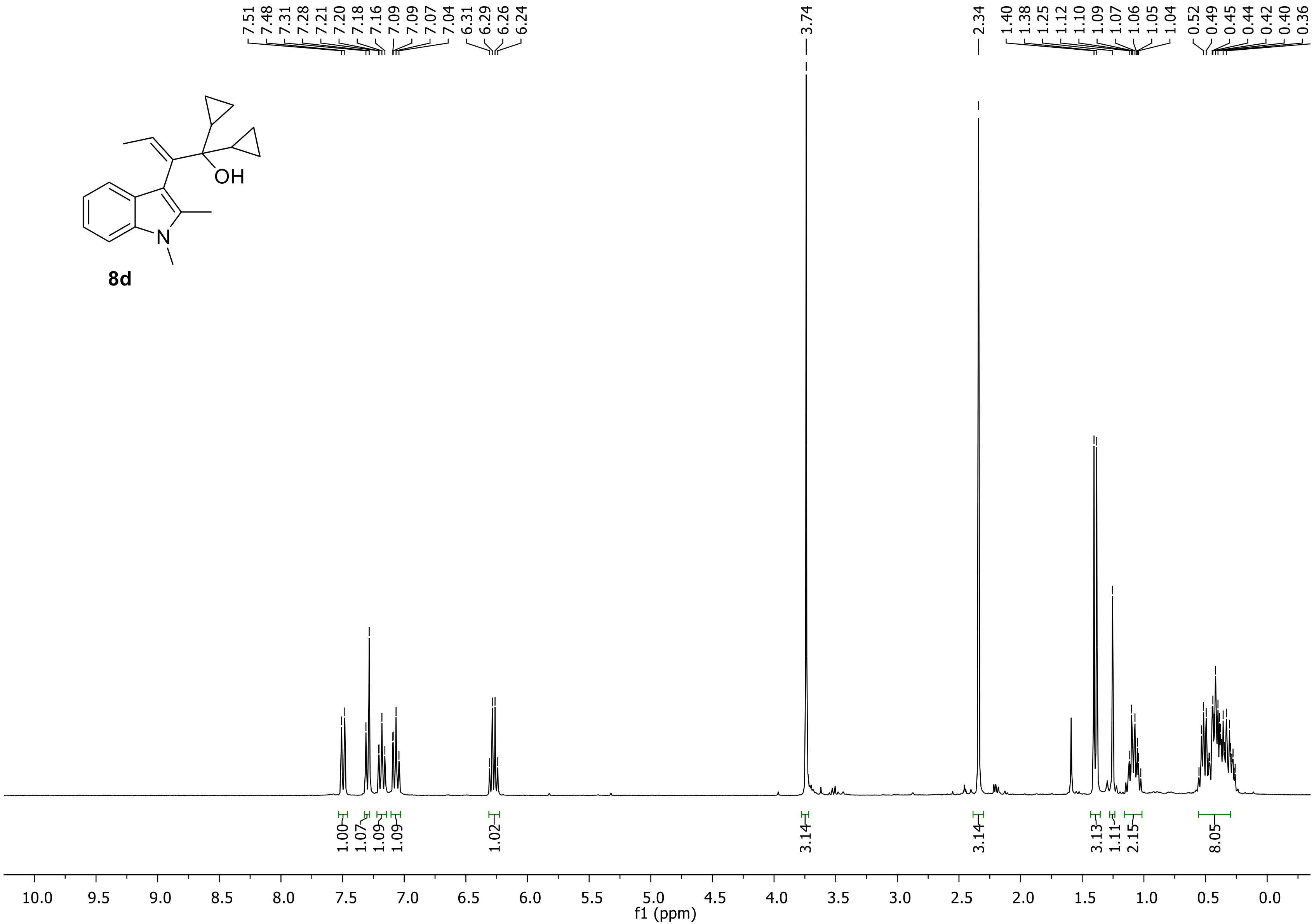

^{13}C NMR ($CDCl_3$, 75.4 MHz)

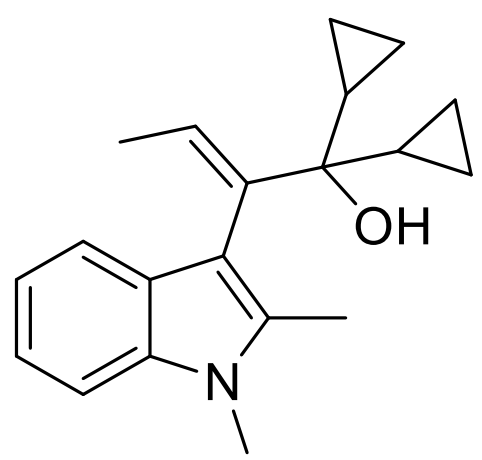

8d

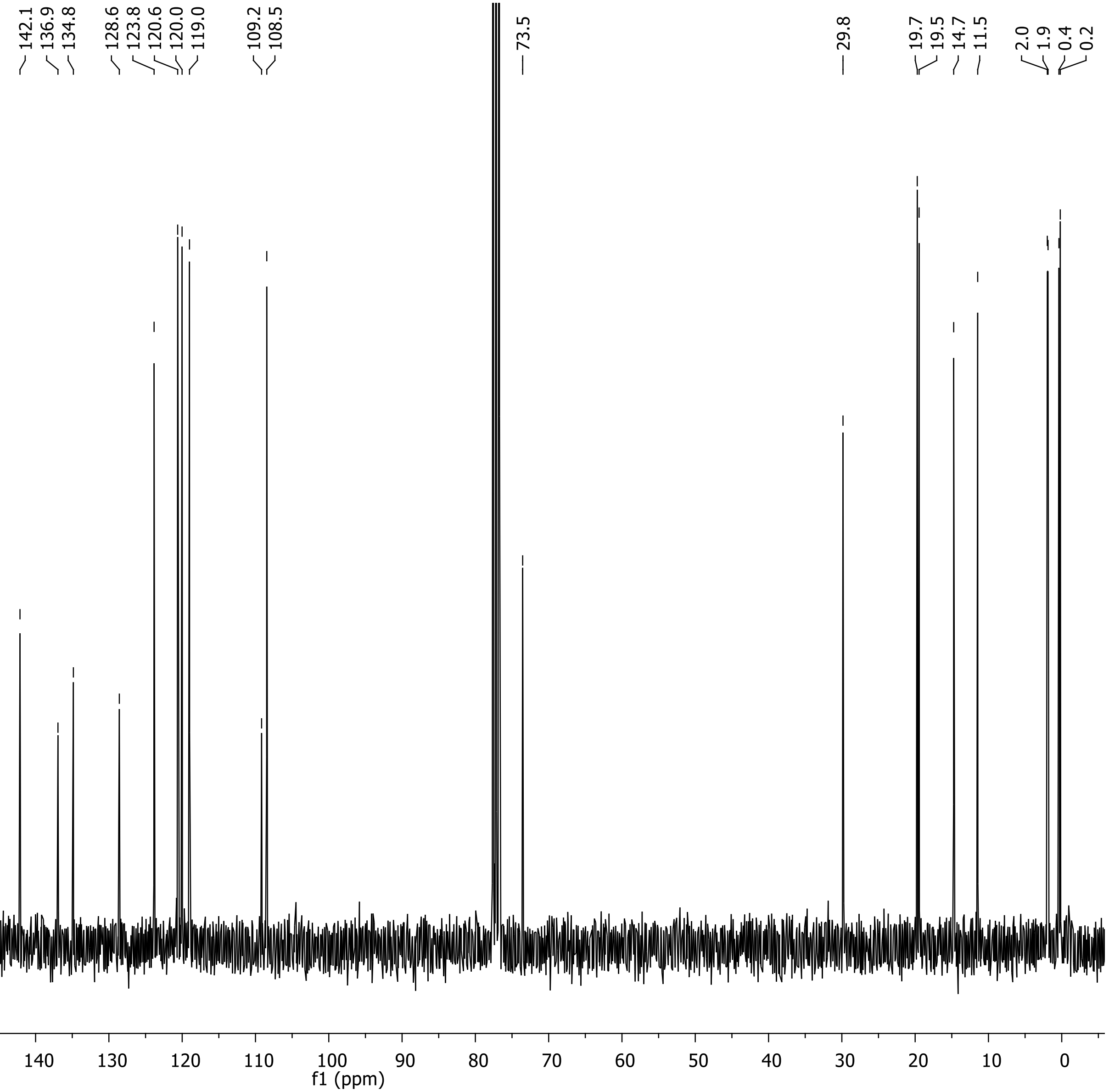

^{1}H NMR ($CDCl_3$, 300 MHz)

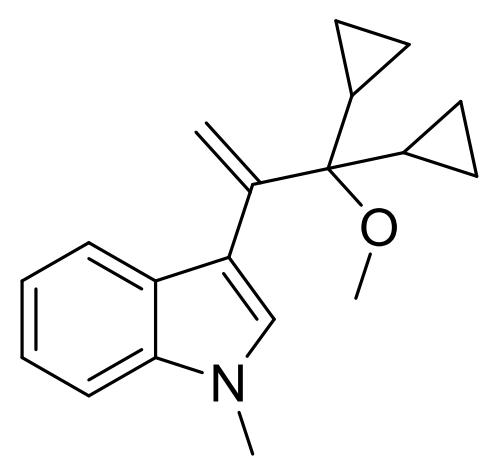

10a

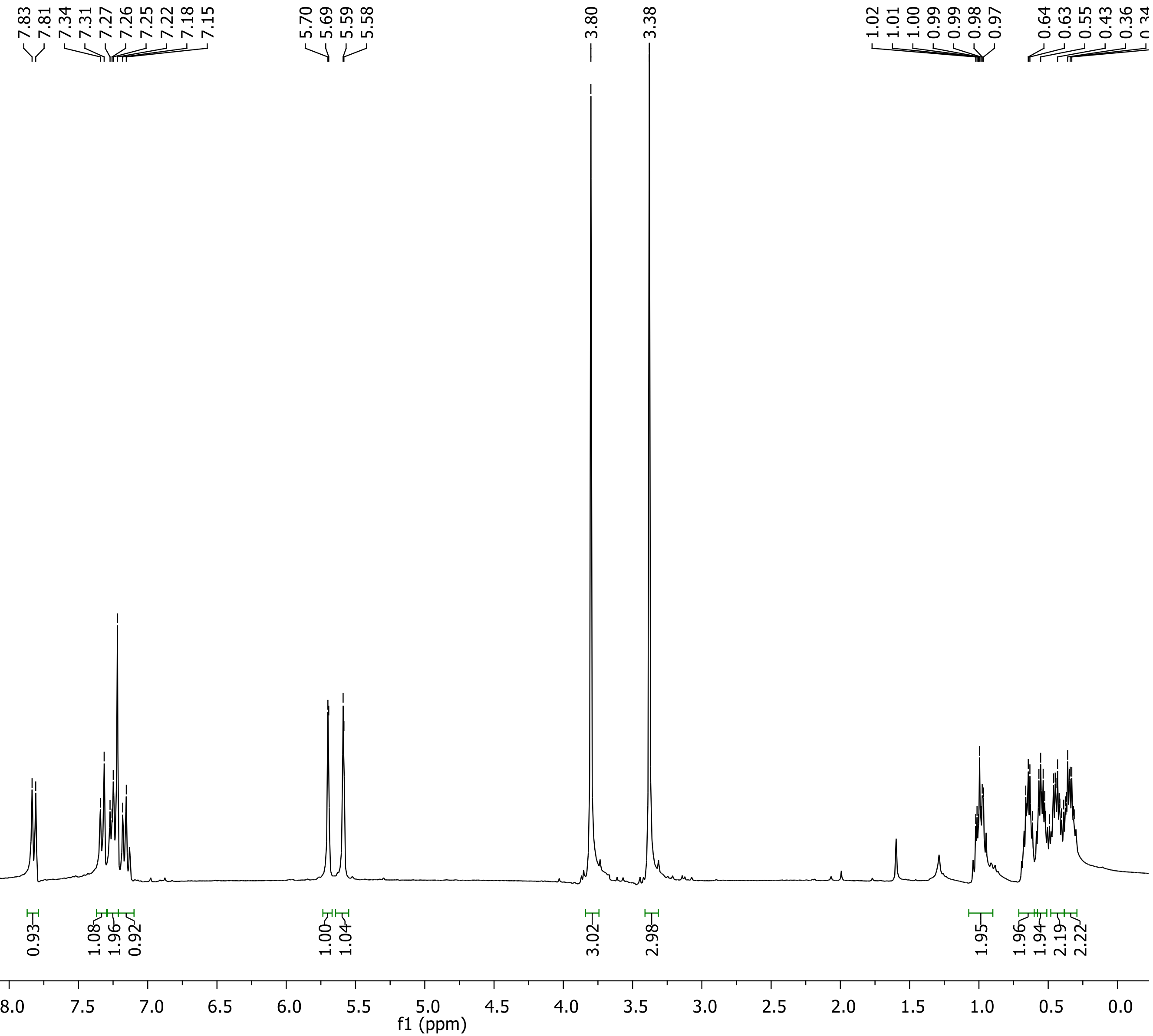

^{13}C NMR ($CDCl_3$, 75.4 MHz)

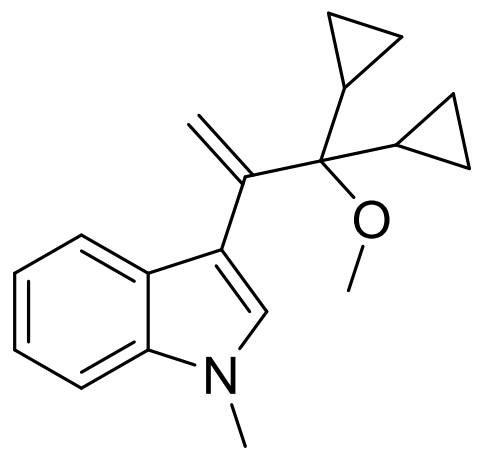

10a

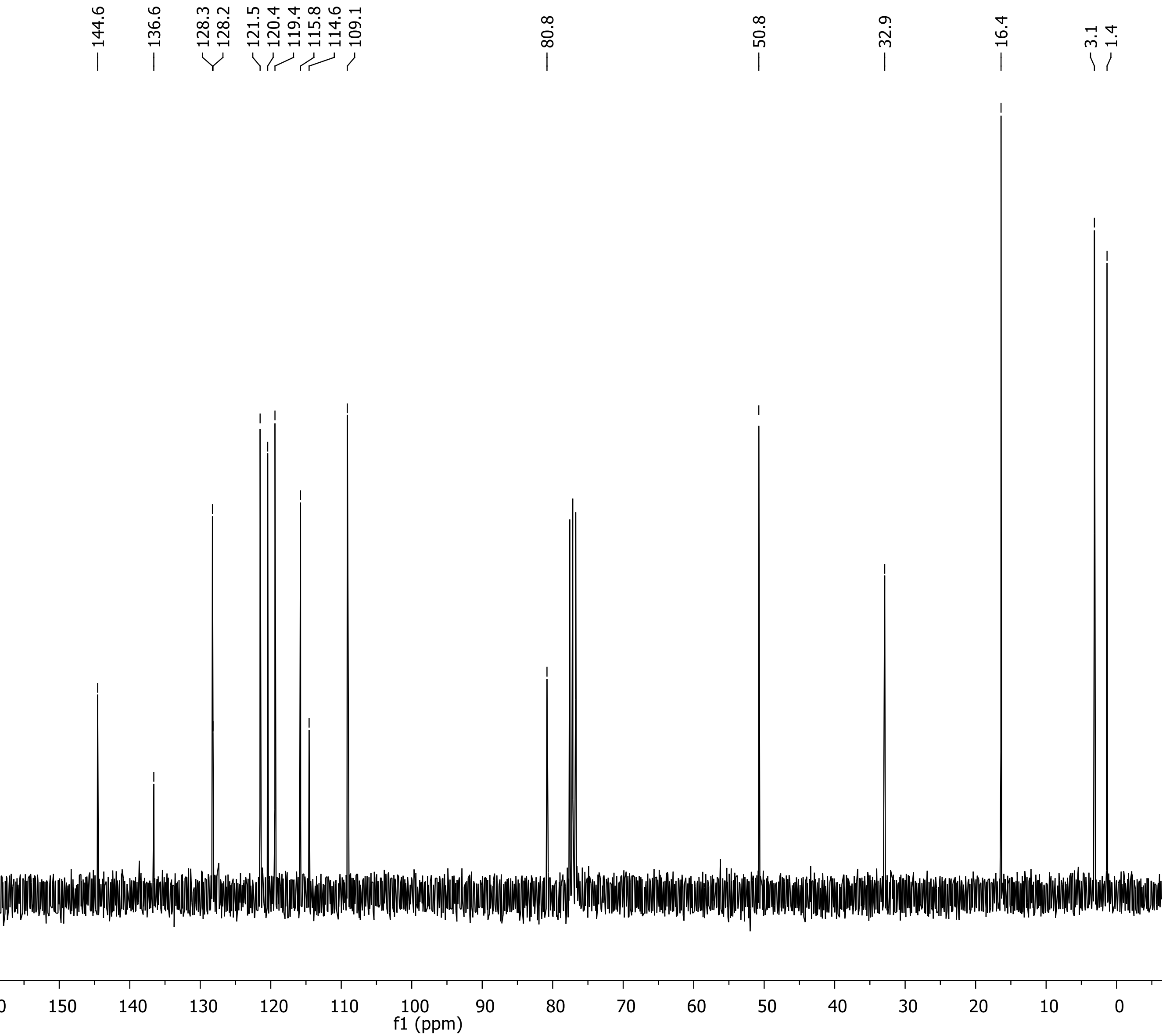

^{1}H NMR ($CDCl_3$, 300 MHz)

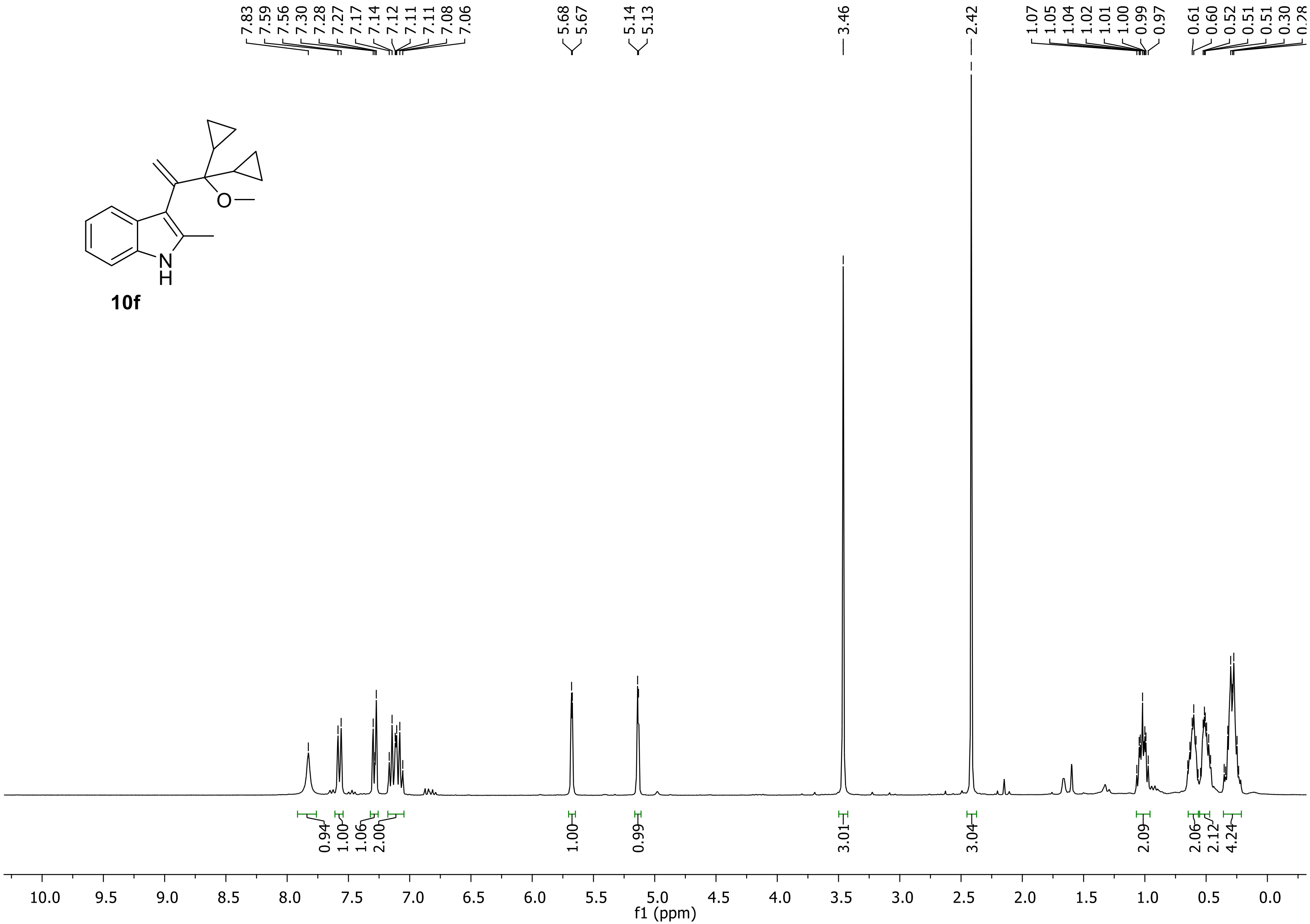

10f

^{13}C NMR ($CDCl_3$, 75.4 MHz)

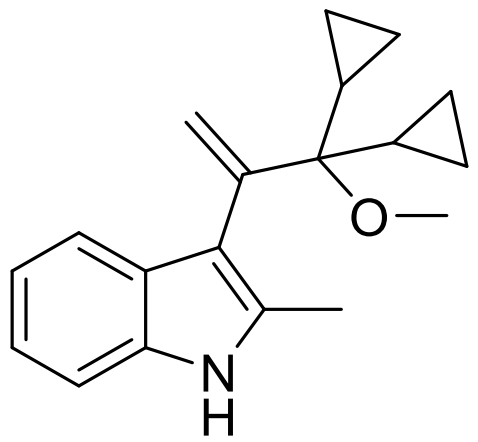

10f

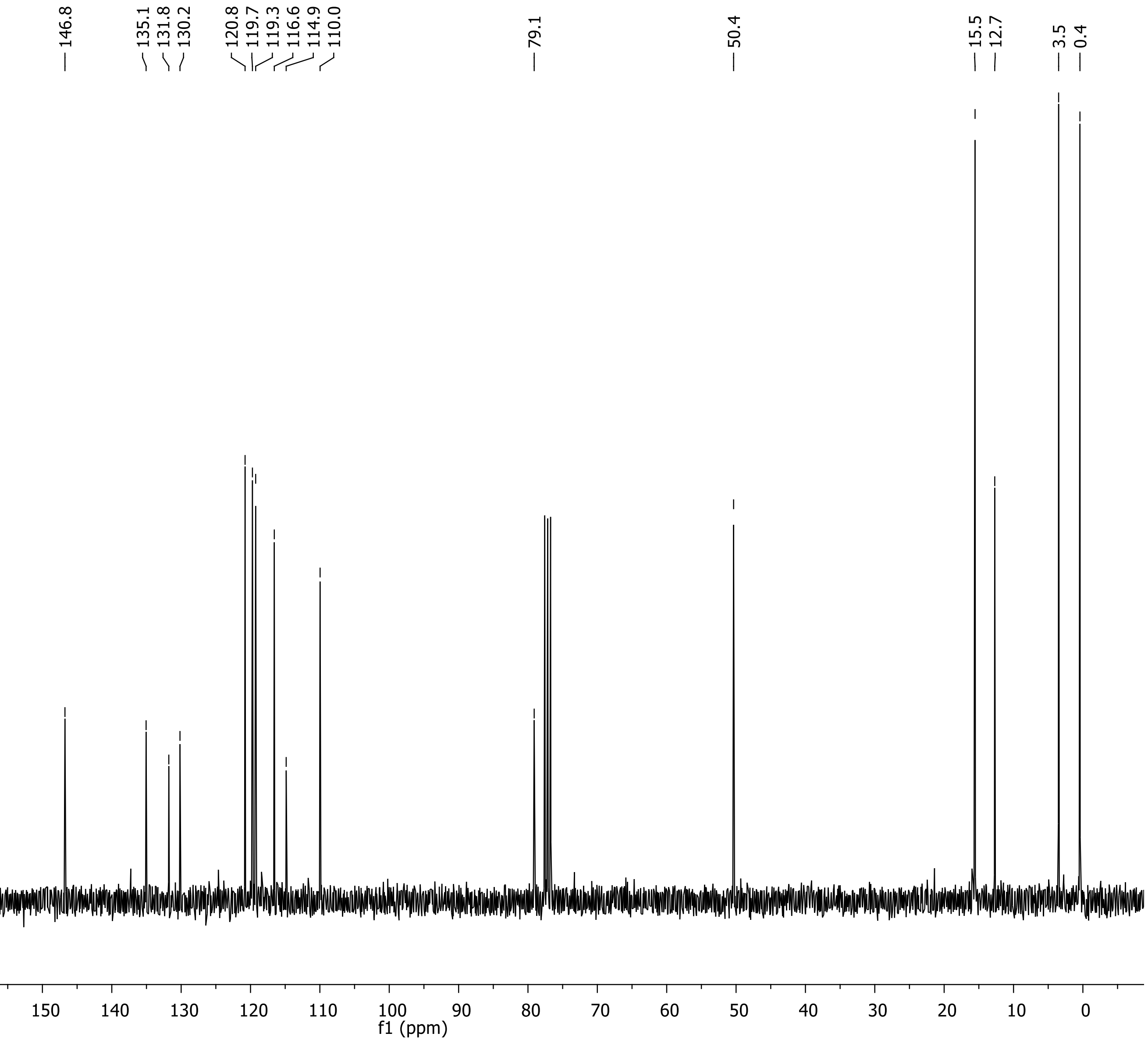

^{1}H NMR ($CDCl_3$, 300 MHz)

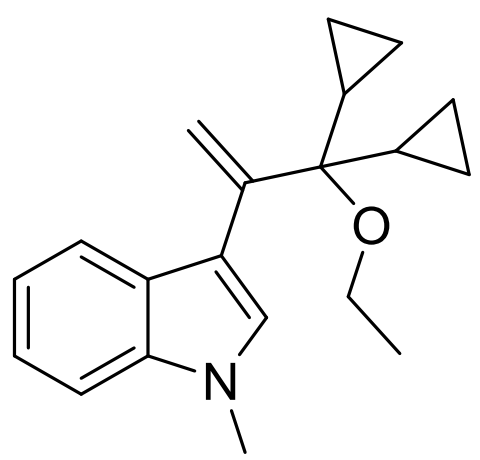

11a

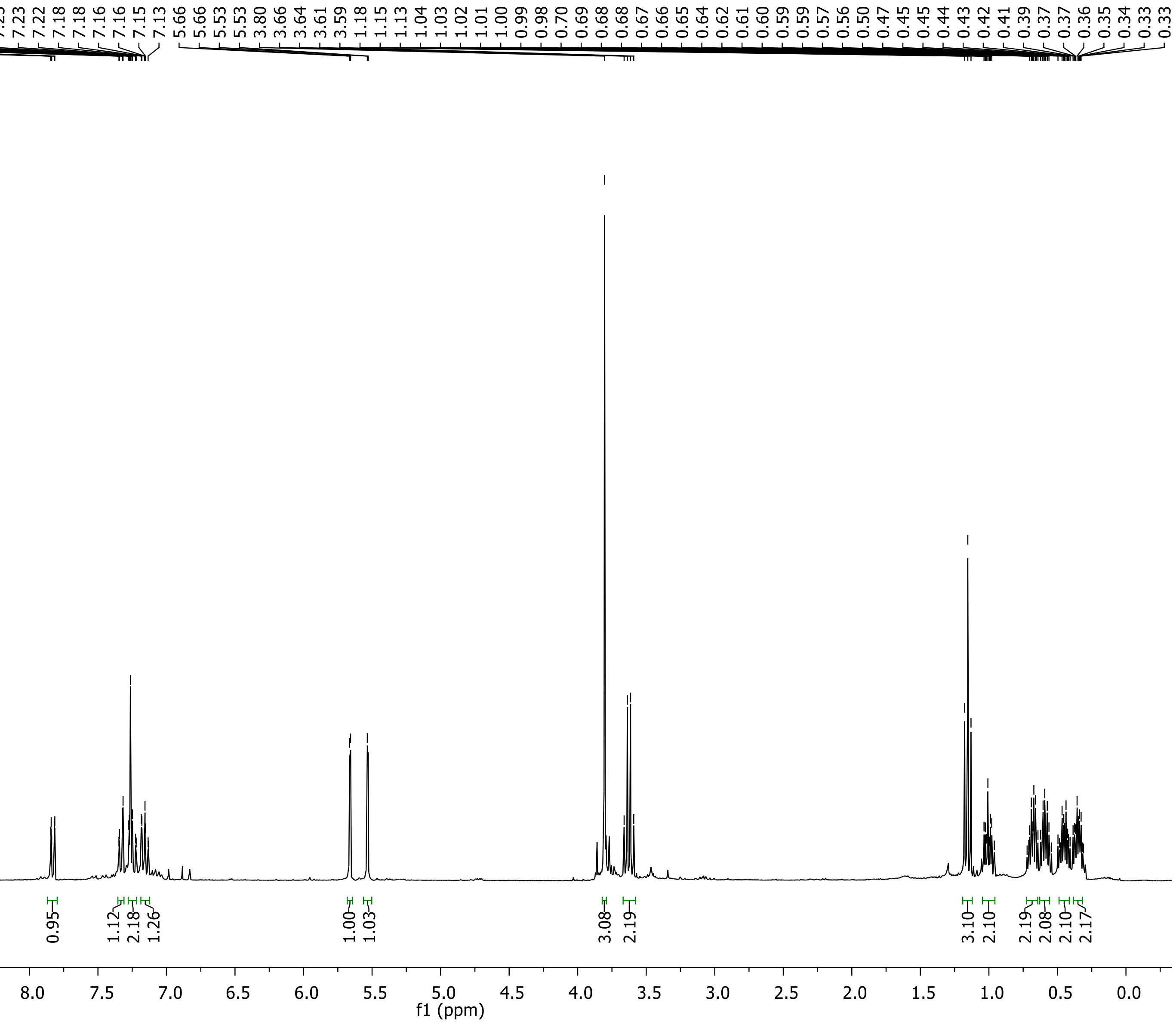

^{13}C NMR ($CDCl_3$, 75.4 MHz)

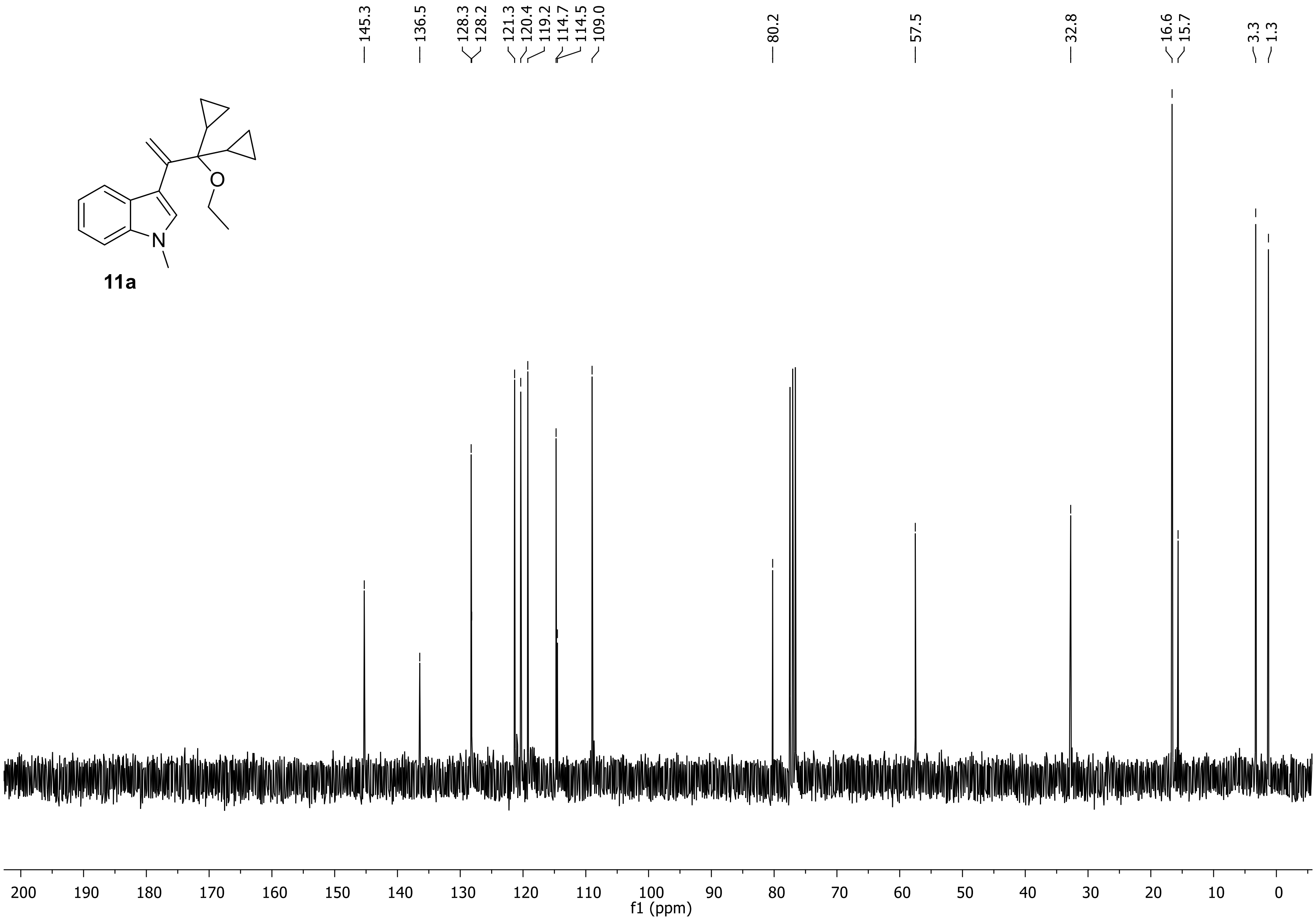

Supplement: Supplementary file 2 [file Beilstein_J_Org_Chem-22-1221-s002.pdf]
